# Randomized evaluation and cost-effectiveness of HIV and sexual and reproductive health service referral and linkage models in Zambia

**DOI:** 10.1186/s12889-016-3450-x

**Published:** 2016-08-12

**Authors:** Paul C. Hewett, Mutinta Nalubamba, Fiammetta Bozzani, Jean Digitale, Lung Vu, Eileen Yam, Mary Nambao

**Affiliations:** 1 Population Council, 4301 Connecticut Avenue, Washington, DC 20008 USA; 2 Society for Family Health, Plot 549 Ridgeway, Lusaka, 10101 Zambia; 3 London School of Hygiene and Tropical Medicine, 15-17 Tavistock Place, London, WC1H 9SH UK; 4 Population Council, One Dag Hammarskjold Plaza, New York, NY 10017 USA; 5 Ministry of Health, Zambia, Ndeke House, Lusaka, Zambia

**Keywords:** Randomized evaluation, Integration, HIV services, Sexual and reproductive health services, Family planning, Voluntary medical male circumcision, HIV testing and counseling, Cervical cancer screening

## Abstract

**Background:**

Provision of HIV prevention and sexual and reproductive health services in Zambia is largely characterized by discrete service provision with weak client referral and linkage. The literature reveals gaps in the continuity of care for HIV and sexual and reproductive health. This study assessed whether improved service delivery models increased the uptake and cost-effectiveness of HIV and sexual and reproductive health services.

**Methods:**

Adult clients 18+ years of age accessing family planning (females), HIV testing and counseling (females and males), and male circumcision services (males) were recruited, enrolled and individually randomized to one of three study arms: 1) the standard model of service provision at the entry point (*N* = 1319); 2) an enhanced counseling and referral to add-on service with follow-up (*N* = 1323); and 3) the components of study arm two, with the additional offer of an escort (*N* = 1321). Interviews were conducted with the same clients at baseline, six weeks and six months. Uptake of services for HIV, family planning, male circumcision, and cervical cancer screening at six weeks and six months were the primary endpoints. Pairwise chi-square and multivariable logistic regression statistical tests assessed differences across study arms, which were also assessed for incremental cost-efficiency and cost-effectiveness.

**Results:**

A total of 3963 clients, 1920 males and 2043 females, were enrolled; 82 % of participants at six weeks were tracked and 81 % at six months; follow-up rates did not vary significantly by study arm. The odds of clients accessing HIV testing and counseling, cervical cancer screening services among females, and circumcision services among males varied significantly by study arm at six weeks and six months; less consistent findings were observed for HIV care and treatment. Client uptake of family planning services did not vary significantly by study arm. Integrated services were found to be more efficiently provided than vertical service provision; the cost-effectiveness for HIV/AIDS and cervical cancer was high in the enhanced service models.

**Conclusions:**

Study results provide evidence for increasing the linkages and integration of a selection of HIV and sexual and reproductive health services. The study provided cost-effective service delivery models that enhanced the likelihood of clients accessing some additional needed health services.

**Trial registration:**

ISRCTN84228514 Retrospectively registered.

The study was retrospectively registered in the ISRCTN clinical trials registry on 06 October 2015. The first recruitment of participants occurred on 17 December 2013.

**Electronic supplementary material:**

The online version of this article (doi:10.1186/s12889-016-3450-x) contains supplementary material, which is available to authorized users.

## Background

Existing data indicate that the integration of sexual and reproductive health (SRH) and human immunodeficiency virus (HIV) services is most likely to be cost effective in generalized HIV epidemic settings with significant unmet need for modern contraception [1, 2] With a national endemic HIV prevalence over 13 % and roughly one out of five married women reporting an unmet need for family planning (FP) [3], conditions in Zambia are ripe for exploring effective strategies for the integration of SRH and HIV services. There is strong consensus among policymakers and cooperating partners within Zambia that better linkages between SRH and HIV services are essential to synergize health impact and greater net-cost savings for the health sector [4, 5].

In Zambia, health services are provided by Government, churches and private institutions. Government and denominational health services are delivered through a system comprising five levels, specialist hospitals, provincial or general hospitals, district hospitals, health centres and health posts. Non-governmental organizations (NGOs), mining and other industrial companies may also provide various specialized health services to address service delivery gaps.

Government and NGO client entry points to HIV testing and counseling (HTC), voluntary medical male circumcision (VMMC), and FP are dominated vertical programming and service specialization and are administered by distinct management structures, established according to historical precedent [6]. In practice, this has resulted in parallel service models, requiring that clients make multiple visits to separate locations to access comprehensive information about add-on SRH or HIV services [7, 8]. An add-on service in this context, therefore, implies a service that may be of interest to the client as it addresses a related health need, but is not readily available; for example, if a man presenting for HIV testing was also interested in VMMC. In most public health facilities, add-on SRH and HIV services are offered sporadically and in separate buildings due to inadequate infrastructure and human resources limitations. Navigating the maze of available service schedules and locations usually requires basic literacy, significant time, and persistence [9–12]. Limited referral and tracking mechanisms often fail to ensure that clients access recommended add-on services; a shortage of human resources and an overburdened and under-resourced public health sector have exacerbated these issues [13].

In Zambia, where 18 % of women aged 15–49 who have ever had sex are living with HIV [14, 15], many programs’ shared client outcome goals are unlikely to be fully met through discrete models of service provision. Strengthened cross-referrals and service linkages have the potential to increase uptake of add-on FP and HIV services among current FP, VMMC, and HTC clients and their partners. A lack of cohesive provider-initiated referral and linkage systems within the public and NGO sectors effectively limits uptake of SRH services as well as treatment and support services for HIV-positive clients, potentially curbing the population-level health benefits of existing SRH and HIV interventions [16, 17].

Not all services are equally suited for on-site integration; maternal and child health (MCH) and FP services, as well as prevention of mother-to-child transmission (PMTCT) services, have been shown to provide synergistic benefits when integrated at shared service points, while FP and sexually transmitted infection (STI) service integration efforts have shown mixed results [18, 19]. Also noteworthy is observational evidence that suggests service sites primarily serving women do not appeal to adult and adolescent males [20], Where fully integrated on-site services are not feasible, stronger provider-initiated referral and linkage systems have been shown to increase uptake of HTC services and improve client perceptions of service quality [21–27].

## Methods

### Study aims, setting and participants

The aims of this study were to contribute to the existing evidence base for best practices in SRH and HIV service linkage and integration, as well as to determine whether two interventions designed to enhance services provision, increase referrals to add-on services, and improve client follow-up, would increase the likelihood that clients would access additional services. Further, it sought to assess whether the interventions could be provided more efficiently as integrated services and would be cost-effective enough to merit implementation at scale.

The study was initiated at seven health service sites within the provincial capitals of Lusaka and Chipata districts of Zambia; Lusaka is also the capital and largest city in Zambia One additional recruitment site was added in each district midway through data collection to address low recruitment rates. The districts were purposefully selected based on the existence of Society for Family Health (SFH) service locations. SFH is a locally registered non-governmental organization that is an affiliate of Population Services International. In coordination with the Zambia Ministry of Health, SFH manages private health services centers (e.g., HTC, VMMC), as well as provides supporting services at select public health facilities.

There were two types of study sites: **entry point sites**, where clients were recruited into the study, and **referral sites,** to which clients were referred for additional services. Each district had at least one of the three entry point services: HTC, FP, and VMMC. At HTC sites, participants of either sex could be recruited, at FP sites recruitment was limited to women, and recruitment at VMMC sites was limited to men. Referral service sites included SFH-operated integrated service centers, public hospitals/clinics, and partner NGO-run service centers; all referral sites were mapped and located within walking distance of the entry point locations. Although the study referred specific sites for services, it was possible that participants enrolled in the study went to health facilities that were not participating in the study. One existing fully-integrated health facility in Lusaka operated by the Young Women’s Christian Association (YWCA) was used to make comparisons of service costs for the economic evaluation.

Participants were largely drawn from a population of urban and peri-urban residents in the two districts of Lusaka and Chipata. At the time of the study, the HIV prevalence was 19 % in and 11 % in Lusaka and Chipata districts, respectively, while the prevalence of male circumcision was 23 and 6 % respectively [3]. The two districts had a similar prevalence (41 %) of participants who had been tested for HIV and had received their test results in the previous year [3]. Among women, there was near universal knowledge of modern methods of contraption (>90 %), however only approximately 32 % of women aged 15–49 were currently using a modern method of contraception, with injectables being the most widely used method [3].

Participants recruited into the study at all of the study entry points met the following eligibility criteria: were 18 years or older; were sexually active, defined as having had sex within the past 12 months; planned to reside within the study catchment area for the next six months; and were willing and able to provide informed consent.

### Client randomization and study arms

**Fig. 1:**
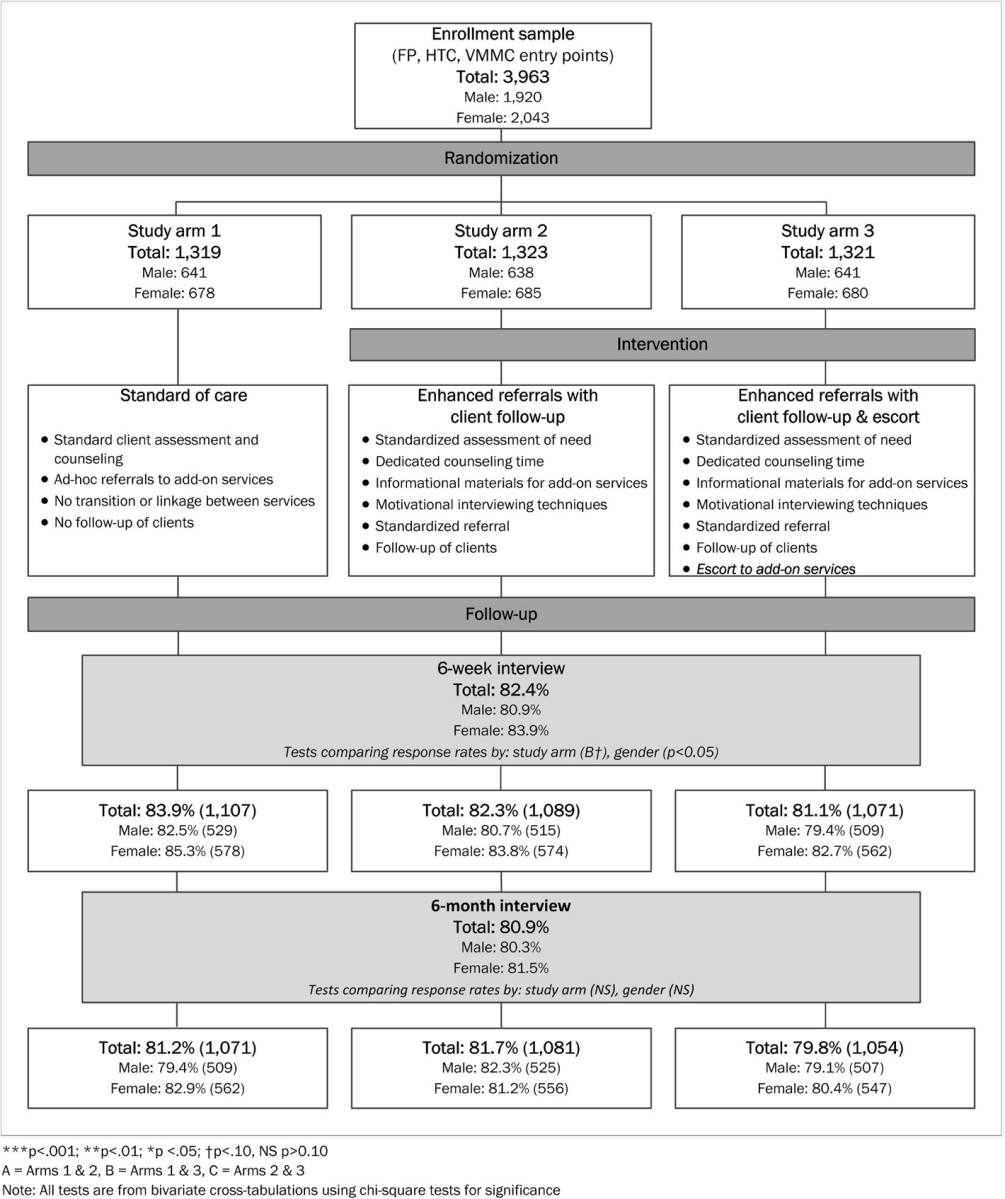
Study participant flow diagram

Clients accessing services at the recruitment sites and voluntarily enrolling in the study were individually randomized into one of three study arms that offered a different package of services (see Fig. 1): **Arm 1)** the standard model of service provision at FP, HTC, and VMMC sites (control); **Arm 2)** enhanced client counseling and referral to add-on services with client follow-up; and **Arm 3)** the components of experimental Arm 2 with an additional offer of immediate escort to the add-on service. A block randomization scheme with a block size of nine, stratified by site, was used to randomly assign participants to ensure balanced sample sizes across control and intervention arms; a small number of cases were ruled ineligible for inclusion after the data was collected. Only after a client registered for the study was his or her random assignment revealed to the research staff member.

Clients randomized to the control arm received the existing standard of care for HTC, FP, and/or VMMC services. Given the differing implementation environments, including public health facilities and NGO-managed sites (SFH), the standard of care varied by site and provider, but, as illustrated in Fig. 1, generally included client assessment and counseling for the services sought; ad-hoc referrals to additional services, usually client initiated; no direct transition or linkage between services; no follow-up of clients. For example, with existing services, female FP clients might on their own ask a nurse counselor about HTC services and be referred for HIV testing. Similarly, HIV-negative male HTC clients might be referred for VMMC services; however, clients were not otherwise linked to those services.

Clients randomized to the two intervention arms were provided enhanced client-centered counseling, a standard process for referrals to additional services, and follow-up by phone if they failed to access the referred service within seven days. The enhanced client counseling included a form-based standardized assessment of need, dedicated time to discuss referrals, readily available informational materials about the add-on services, and used client-centered motivational interviewing (MI) techniques to address barriers to service uptake. MI is a client-centered and directed approach to behavioral counseling designed to enhance a client’s readiness for change by eliciting his/her own motivations. Counsellors interactively work with clients to address potential barriers to service uptake. MI has been shown to significantly increase client engagement, intention, and self-efficacy in the adoption of new health practices, including utilization of HTC and antiretroviral therapy (ART) adherence [28–30].

Referrals for services were given for the primary client as well as for his or her spouse/partner and children when appropriate; given the small number of clients with children for which there were data, the children’s referrals are not discussed further. If clients failed to access the referral service within seven days, they were called on the phone by a psychosocial counselor who used MI techniques to address barriers to accessing services. Clients who were randomized to the third study arm were additionally offered an immediate escort at the time of referral, who would guide clients to the referral site. The escort physically walked the client to the add-on service and introduced the client to the site and processes, including registration. For practical and ethical reasons the client did not receive preferential access to services at referral sites. For relevant add-on services not used immediately (e.g., for spouses/partners), providers encouraged clients to return with the secondary beneficiary to the entry point for escort to the services; such clients, however, could proceed directly to the referral site if desired.

### Study outcomes and client observations

The referral service uptake outcomes and the eligible population included in assessments of the primary objectives of this study are listed in Table 1. The outcomes were measured for enrolled clients, as well as their spouses/partners, if applicable. Uptake was defined as clients reporting that they or their spouse/partner utilized a referred service within the six-week or six-month period after enrollment. As is clear from Table 1, whether or not an outcome could be measured was dependent upon the entry point from which the client was recruited. For instance, uptake of HTC as a referral service could only be observed among females enrolled at FP sites, since men were routinely tested for HIV at both HTC sites and VMMC sites.

**Table 1:** Study outcomes by entry point and gender eligibility

	Entry points	Gender eligible	Notes
Client outcomes			
HIV testing and counseling	FP	Female	
Family planning	HTC	Female	Among all non-pregnant women able to have children at baseline^a^
Voluntary medical male circumcision	HTC	Male	
Cervical cancer screening	FP, HTC	Female	
HIV & STI care and treatment outcomes			
HIV care and treatment	FP, VMMC, HTC	Male, Female	
STI care and treatment	FP, VMMC, HTC	Male, Female	
Psychosocial counseling	FP, VMMC, HTC	Male, Female	Among all who got HIV care and treatment
TB testing	FP, VMMC, HTC	Male, Female	Among all who got HIV care and treatment
CD4 testing	FP, VMMC, HTC	Male, Female	Among all who got HIV care and treatment
Initiated ART	FP, VMMC, HTC	Male, Female	Among those who were tested for CD4 counts and were eligible for ART
Partner outcomes			
HIV testing and counseling	FP, VMMC, HTC	Male, Female	Among those with primary sex partners
Family planning	VMMC, HTC	Male	Among those with primary sex partners
Voluntary medical male circumcision	FP, HTC	Female	Among those with primary sex partners
Cervical cancer screening	VMMC, HTC	Male	Among those with primary sex partners
HIV care and treatment	FP, VMMC, HTC	Male, Female	Among those with primary sex partners
STI care and treatment	FP, VMMC, HTC	Male, Female	Among those with primary sex partners

^a^Women self-reported that they were not able to have children in the baseline survey

The study collected information from the client at enrollment, six weeks, and six months post-enrollment. A client information registry and tracking database (CTD) collected client information, including names, national registration card number, contact information, and services sought on the day of recruitment. The CTD included site name, visit date, time of visit start, time of visit end, and services received. Based on Microsoft SQL architecture, the CTD at each individual entry point and referral site was synchronized every ten minutes with a central server using USB 3G/4G modems. The synchronization provided real-time information about clients accessing referral services and was used to generate reports of those in intervention arms who failed to access referral services within seven days.

All study clients were interviewed at baseline by a trained enumerator at the client entry point prior to service provision. Information collected at baseline included socio-demographic characteristics; residential and household information; recent health service utilization; recent sexual and HIV risk behaviors; self-assessments of health status; recent STI diagnosis, treatment and symptoms; fertility desires; and contraceptive use. All clients were tracked at six weeks and six months post-enrollment for an interview that included questions about service uptake at study and nonstudy health facilities. The data from survey interviews were electronically captured on Android™ tablets and audio computer-assisted self-interviewing (ACASI) was used for the sexual behavior questions.

### Power and statistical analysis methods

Power analysis for the experimental evaluation was performed to determine the study sample sizes required to statistically assess minimally detectable treatment effects. The sample size required for each study entry point depended on the outcome to be analyzed, how it was measured, its estimated standard deviation, an estimate of its value at baseline, and the anticipated treatment effect from the intervention(s). For the calculations performed, a standard power of 0.80 and a significance level of 0.05 were specified. The sample size was generated for an acceptable minimally detectable effect size for each indicator. Power calculations were performed such that each of the two experimental arms could be statistically evaluated against the control arm, as well as against each other.

The empirical assessment of the primary outcome indicators in Table 1 was based on client interviews conducted at approximately six weeks and six months after enrollment. The empirical assessment was based on an intent-to-treat (ITT) analysis, assessing cases according to their original randomization whether or not they actually received any or all of intervention components in their assigned arm as per protocol [31]. Because of random assignment, relatively simple statistical methods were used to assess intervention impact. Differences in the means for continuous indicators were assessed using pairwise t-tests of significance, while pairwise chi-square tests were used to assess differences between study arms for binary indicators at six weeks and six months; Fisher’s exact test was used as an alternative when cell sizes were small.

Two logistic regression multivariable models were also used to assess treatment impact. The first regression model included only indicators for the study arms, with the standard of care as reference. The second model included the study arms, service entry point site fixed effects and a limited number of covariates to determine whether the precision of the parameters was improved with more information empirically modeled. A separate generalized estimating equations (GEE) analysis was conducted to assess whether within-site clustering may have affected the parameter standard errors and statistical tests. Also, given loss-to-follow-up at six weeks and six months, a sensitivity analysis was conducted using multiple imputation methods for missing data to determine if any conclusions drawn might have changed if the outcomes were fully observed [31, 32]. As the conclusions that were drawn from the GEE and multiple imputation models did not differ in any meaningful way from the standard statistical assessments, the latter results are presented [33]. All analyses were performed using Stata 13.1 (Stata Corp, College Station Texas).

### Economic evaluation

The embedded economic evaluation comprised two components, a technical efficiency and a cost-effectiveness analysis. Technical efficiency has to do with minimizing waste in the provision of a given service and is achieved when the desired output is produced with the least inputs [34]. Interventions costs were assessed in terms of whether they varied depending on mode of delivery, either separately via vertical, stand-alone services or together via integrated services (under one roof). The urban vertical service sites in Lusaka were SFH Cairo (HTC), SFH ChaChaCha (VMMC) and Chawama Clinic (FP). The rural vertical service sites in Chipata were the SFH Platform (HTC), Chipata General Hospital (VMMC) and Kapata Clinic (FP). The integrated comparator site was SFH YWCA in Lusaka. All other referral services were costed at Chawama Clinic, a representative public health facility. All study facilities were part of existing SFH referral networks in Lusaka and in Chipata. Costs were collected using an ‘ingredients’ approach by which all resource items used in the production of the services are identified. Costs per client were then multiplied by the number of clients seen at the facility in one year to calculate annual costs used in the cost-effectiveness analysis.

The second component of the economic evaluation assessed the value-for-money of the two intervention arms in the trial compared to the standard of care. Cost-utility analysis using Disability Adjusted Life Years (DALYs) as the unit of outcome was used since the health outcomes of the study predominantly affected morbidity in the short run. DALYs are considered the lost years of a healthy life due to disease or disability [35]. Discounted years of life lost (YLLs) were calculated as the sum of expected deaths in the Zambian population over time based on the life expectancy in different age groups. Discounted years lost due to disability (YLDs) were calculated as the sum of the years lost due to disability caused by HIV infection, onset of AIDS, and cervical cancer. The number of Zambians experiencing different health states at time (t) was calculated based on the incidence and mortality estimates for the outcomes of interest as well as the probability of treatment success and mortality and morbidity reduction from the different interventions, summarized in Table 2. Incremental DALYs averted from the trial interventions were then calculated as the sum of YLLs and YLDs in each intervention arm. The disability weights used for symptomatic pre-AIDS HIV, AIDS with and without ART, and cervical cancer (diagnosis and primary therapy) were those used in the Global Burden of Disease Study 2010 [36]. Discounted DALY measures and projected lifetime treatment costs derived from these intermediate outcomes were used to calculate incremental cost-effectiveness ratios, which provide forthright cost comparisons between the experimental and control service models.

**Table 2:** Model parameter assumptions used for estimation of Disability Adjusted Life Years

Parameter	Assumption		Source
Discount rate		0.03	WHO recommendation
HIV incidence in Zambian pop.	Male	Female	UNAIDS Zambia 2014 [43]
15–24	0.45 %	0.98 %	
25–29	0.01 %	0.05 %	
30–34	0.00 %	0.00 %	
35–39	0.00 %	0.01 %	
40+	0.00 %	0.01 %	
HIV mortality (no ART)		0.0214	Lozano 2012 [44]
Incidence reduction from HTC			
Discordant couple		74.00 %	Allen 2014 [45]
M-F couple		91.00 %	Allen 2014 [45]
Mortality reduction from ART		11.40 %	UNAIDS Zambia 2014 [43]
ART coverage		90.00 %	UNAIDS Zambia 2014 [43]
Prevalence reduction from VMMC			UNAIDS Zambia 2014 [43]
15–24		1.10 %	
25–29		−7.00 %	
30–34		2.30 %	
35–39		13.70 %	
40+		22.90 %	
Cervical cancer incidence		0.09	Sankaranarayanan 2006 [46]
Cervical cancer mortality		0.04	Sankaranarayanan 2006 [46]
Mortality reduction from CCS		81.00 %	Mandelblatt 2002 [47]

## Results

As can be observed in Fig. 1, 3963 men and women were enrolled to participate in the study; 42 % enrolled from health facilities in Lusaka and 58 % from health facilities in Chipata. A total of 2043 women (representing 52 % of the sample) were enrolled from FP and HTC entry sites, while a total of 1920 men (representing 48 % of sample) were recruited from VMMC and HTC entry sites. While the number of women enrolled was nearly equally divided between FP and HTC sites, only 27 % of the enrolled men entered from VMMC sites, the remainder coming from the study’s HTC sites. The distribution of participants by study arm included 1319 in the standard of care or control arm (study arm 1), 1323 in the enhanced services with follow-up arm (study arm 2), and 1321 in the enhanced services with follow-up and escort arm (study arm 3). The baseline characteristics of the sample did not vary systematically by study arm [33].

### Participant baseline characteristics

Table 3 provides an overview of the baseline demographic characteristics of the sample by participant sex. The mean age of study participants was 26.5 (95 % CI: 26.3–26.7) years of age, with a slightly younger sample of males (26.1, 95 % CI: 25.8–26.4) than females (26.8, 95 % CI: 26.5–27.1). The majority of male participants were in the 18–24 age range (51.0 %, 95 CI: 48.7–53.2 %), with a decreasing prevalence of men across the remaining age ranges. A similar pattern was observed for females, a plurality of females seeking FP and HTC services (44.8 %, 95 % CI: 42.7–47.0 %) coming from the 18–24 age group and only 14 % (95 % CI: 12.6–15.7 %) of participants aged 35 years or older. Males were also more likely to currently attend school (28.7 %, 95 % CI: 26.7–30.8 %), relative to females (13.9 %, 95 % CI: 12.4–15.4 %). The difference in educational attendance is likely driven by the sex differences observed in schooling attainment at secondary and tertiary levels in Zambia [3]. A larger percentage of males than females in our sample were 18–24 years old, ages when they are likely to be attending school, presumably at the tertiary level. The mean grade of schooling attainment was nine (one year into secondary school in Zambia), with males on average more likely to have entered secondary.

**Table 3:** Baseline demographic characteristics of study participants by sex (percentages unless otherwise indicated)

Sample size	Males		Females		Total	
	1920		2043		3963	
	% (or mean)	95 % CI	% (or mean)	95 % CI	% (or mean)	95 % CI
Mean age in years	26.1	25.8–26.4	26.8	26.5–27.1	26.5	26.3–26.7
Age groups						
18–24	51.0	48.7–53.2	44.8	42.7–47.0	47.8	46.3–49.4
25–29	22.4	20.5–24.3	23.8	22.0–25.7	23.1	21.8–24.5
30–34	13.6	12.1–15.3	17.2	15.6–18.9	15.5	14.4–16.7
35–39	7.9	6.7–9.2	9.3	8.0–10.6	8.6	7.7–9.5
40+	5.1	4.1–6.1	4.8	4.0–5.9	4.9	4.3–5.7
Currently attending school						
No	71.3	69.2–73.3	86.1	84.6–87.6	78.9	77.6–80.2
Yes	28.7	26.7–30.8	13.9	12.4–15.4	21.1	19.8–22.4
Mean grade completed	10.4	10.2–10.5	8.0	7.8–8.2	9.2	9.0 –9.3
Marital status						
Never married	58.9	56.6–61.1	23.7	21.9–25.6	40.7	39.2–42.3
Currently married/living with partner	31.1	29.1–33.3	63.8	61.7–65.9	48.0	46.4–49.6
Divorced/separated/widowed	10.0	8.7–11.4	12.5	11.1–14.0	11.3	10.3–12.3
Among unmarried, has regular sexual partner^a^						
No	33.6	31.0–36.2	29.6	26.3–33.0	32.2	30.1–34.2
Yes	66.4	63.8–69.0	70.4	67.0–73.7	67.8	65.8–69.9
Mean number of biological children	1.0	1.0–1.1	2.5	2.4–2.6	1.8	1.7–1.8
Residence						
Urban	93.2	92.0–94.3	95.4	94.5–96.3	94.4	93.6–95.1
Rural	6.8	5.7–8.0	4.6	3.7–5.5	5.6	4.9–6.4
Tribe						
Lozi	3.2	2.5–4.1	3.5	2.7–4.4	3.4	2.8–4.0
Ngoni	28.5	26.5–30.6	32.7	30.6–34.7	30.6	29.2–32.1
Tonga	5.5	4.5–6.6	5.4	4.4–6.5	5.4	4.7–6.2
Bemba	13.5	12.0–15.1	14.2	12.7–15.7	13.8	12.8–14.9
Other	49.3	47.1–51.6	44.3	42.1–46.5	46.7	45.2–48.3
Religion						
Catholic	21.3	19.5–23.2	17.0	15.4–18.7	19.1	17.9–20.3
Christian	72.9	70.9–74.9	78.9	77.1–80.7	76.0	74.6–77.3
Other	5.8	4.8–6.9	4.1	3.3–5.1	4.9	4.3–5.6
Employment status						
Not working	43.6	41.4–45.9	63.8	61.7–65.9	54.0	52.5–55.6
Currently working	56.4	54.1–58.6	36.2	34.1–38.3	46.0	44.4–47.5
Mean number of household assets (0–15)	8.3	8.1–8.4	7.1	7.0–7.3	7.7	7.6–7.8
Household assets						
Lowest quintile	16.4	14.8–18.1	28.0	26.1–30.0	22.4	21.1–23.7
Middle quintiles	69.1	67.0–71.2	64.1	61.9–66.2	66.5	65.0–68.0
Highest quintile	14.5	12.9–16.1	7.9	6.7–9.1	11.1	10.1–12.1
Has own mobile phone						
No	12.4	11.0–14.0	21.4	19.6–23.2	17.1	15.9–18.3
Yes	87.6	86.0–89.0	78.6	76.8–80.4	82.9	81.7–84.1
Type of water source						
Piped	81.9	80.1–83.6	79.9	78.1–81.6	80.9	79.6–82.1
Well/Spring	8.9	7.7–10.3	9.5	8.3–10.9	9.2	8.3–10.2
Borehole	8.9	7.7–10.3	9.6	8.4–11.0	9.3	8.4–10.2
Other	0.3	0.1–0.7	1.0	0.6–1.5	0.7	0.4–1.0
Mean time to water source (minutes)	2.7	2.4–3.0	3.0	2.7–3.4	2.9	2.6–3.1
Type of transport to health facility						
Walking	60.6	58.4–62.8	59.7	57.5–61.8	60.1	58.6–61.7
Bicycle	5.9	4.9–7.0	2.3	1.7–3.1	4.0	3.5–4.7
Bus	30.6	28.5–32.7	37.0	34.9–39.1	33.9	32.4–35.4
Car/Taxi	2.9	2.2–3.8	1.1	0.7–1.6	2.0	1.6–2.5
Mean distance to health facility^b^	4.7	4.3–5.1	2.7	2.4–3.0	3.8	3.5–4.1

^a^Those who are living together considered “married”

^b^38 % don’t know distance, are missing on this variable

Participants were interviewed at six weeks and six months after enrollment (Fig. 1). Of the 3963 males and females enrolled, 82.4 % were interviewed at six weeks and 80.9 % at six months, averaged across the study arms. The differences in follow-up rates between males and females were statistically significant (*p* < .05) at the six-week interview, but those statistically significant differences dissipated by the six-month interview. As indicated in Fig. 1, there were no meaningful differences between the follow-up interview response rates across study arms [33]. Those in the standard of care arm were marginally more trackable than those in the other two arms at the six-week interview, with the greatest difference (3 %, *p* < .10) between the control and the enhanced referral and escort arm. Statistically significant differences in attrition between arms did not exist at six months. The finding of no statistically significant differences in follow-up across arms over time provides greater confidence in the experimental assessment of outcomes reported in the behavioral data. Multivariable logistic attrition analyses (not shown) were also conducted to assess differences between those interviewed and those not interviewed [33]. Women were significantly less likely to be lost to follow-up than were men. Characteristics that were significantly associated with attrition over time were being younger, having lower educational attainment, being divorced, separated, or widowed (at six weeks), being Catholic (at six weeks), and not owning a mobile phone (at six weeks).

### Impact assessment

Tables 4 and 5 indicate that the impact of the interventions on the study outcome indicators did not have a consistent effect across all indicators; however, meaningful results were found for a selection of outcomes. Focusing on the results at the six-month follow-up displayed in Table 5, for clients—the primary target of the interventions—a statistically significant increase in the uptake of services was found for HTC services (*p* < .10), VMMC (*p* < .001), and cervical cancer screening (CCS) services (*p* < .001), but not for the uptake of FP, HIV care and treatment, or STI care and treatment services. For clients who indicated that they had utilized HIV care and treatment services in the previous six months, there were indications that the interventions did improve uptake of tuberculosis (TB) testing services (*p* < .01), with marginally statistically significant findings for initiation of ART.

**Table 4:** Descriptive statistics of service uptake outcomes at six weeks by study arm (percentages)

Uptake of services at 6 weeks				
	Arm 1	Arm 2	Arm 3	
	Standard of care	Enhanced services	Enhanced services & escort	Tests
Females (n range)	279–577	283–574	276–562	
HIV testing and counseling	13.4	17.2	21.3	B*
Family planning	15.4	19.3	10.6	C**
Cervical cancer screening	4.2	21.3	24.6	A***, B***
Males (n)	385	363	362	
Voluntary medical male circumcision	4.7	8.8	12.2	A*, B***
Both females and males (n range)	23–1104	37–1084	32–1070	
HIV care and treatment	8.5	9.4	10.7	B†
STI care and treatment	3.8	4.6	4.2	
TB testing^a^	20.0	21.6	17.5	
CD4 testing	42.1	54.4	43.0	A†
Initiated ART^a^	95.7	86.5	87.5	
Psychosocial support	32.3	18.8	23.2	A*
Partners of clients (n range)	361–872	381–882	357–832	
HIV testing and counseling	21.0	23.2	23.0	
Family planning	16.3	17.0	17.9	
Voluntary medical male circumcision	3.9	3.7	5.5	
Cervical cancer screening	7.6	6.5	10.5	C†
HIV care and treatment	7.8	7.9	9.0	
STI care and treatment	5.2	4.8	4.8	

A = Arms 1 & 2, B = Arms 1 & 3, C = Arms 2 & 3

Note: Five cases are excluded for answering these questions for a 6-month window instead of a 6-week window

Note: Sample sizes for client outcomes vary as each outcome is restricted to certain entry sites and a few participants chose not to disclose receipt of certain services

Note: All tests are from bivariate cross-tabulations using chi-square tests for significance, unless otherwise noted

***p < .001; **p < .01; *p < .05; †*p* < .10

^a^Tested using Fisher’s exact test due to small cell sizes

**Table 5:** Descriptive statistics of service uptake outcomes at six months by study arm (percentages)

Uptake of services at 6 months				
	Arm 1	Arm 2	Arm 3	
	Standard of care	Enhanced services	Enhanced services & escort	Tests
Females (n range)	283–559	273–555	273–547	
HIV testing and counseling	28.3	34.9	33.9	A†
Family planning	23.7	19.3	22.3	
Cervical cancer screening	9.7	22.2	23.6	A***, B***
Males (n)	371	375	364	
Voluntary medical male circumcision	4.3	6.1	11.5	B***, C**
Both females and males (n range)	43–1070	57–1080	53–1054	
HIV care and treatment	8.0	9.5	10.0	
STI care and treatment	4.5	4.1	4.5	
TB testing^a^	18.8	24.5	37.3	B**, C*
CD4 testing	63.5	73.5	71.6	
Initiated ART^a^	93.0	100.0	98.1	A†
Psychosocial support	15.3	12.9	16.7	
Partners of clients (n range)	371–857	399–873	361–849	
HIV testing and counseling	33.9	35.1	39.2	B*, C†
Family planning	25.1	23.9	21.4	
Voluntary medical male circumcision	3.6	6.3	5.4	A†
Cervical cancer screening	12.6	14.9	13.7	
HIV care and treatment	7.3	8.3	8.5	
STI care and treatment	4.2	3.3	4.2	

A = Arms 1 & 2, B = Arms 1 & 3, C = Arms 2 & 3

Note: Two cases are excluded for answering these questions for a six-week window instead of a six-month window

Note: Sample sizes for client outcomes vary as each outcome is restricted to certain entry sites and a few participants chose to not disclose receipt of certain services

Note: All tests are from bivariate cross-tabulations using chi-square tests for significance, unless otherwise noted

***p < .001; **p < .01; *p < .05; †*p* < .10

^a^Tested using Fisher’s exact test due to small cell sizes

For the secondary target of the interventions—the partners of clients—only in the uptake of HTC in study arm three was there a statistically significant effect (*p* < .05), although marginally statistically significant findings (*p* < .10) were observed for VMMC. In most cases, excluding FP, the interventions increased the use of services relative to the standard of care.

The multivariable adjusted regression models for six weeks and six months are presented in Tables 6 and 7 respectively. As indicated model 2 in Table 7, female clients enrolled in the intervention arms at FP sites had 36 % (study arm 2) and 28 % (study arm 3) higher odds of accessing HTC services than clients in the control, although these results were only marginally statistically significant at *p* < .10 for clients in study arm two (AOR 1.36, 95 % CI: 0.95–1.95). The adjusted logistic regression results also indicate that men entering HTC sites in the referral-plus-escort arm (study arm 3) had nearly three times the odds of taking up VMMC services than men in the standard of care arm (AOR 2.85, 95 % CI: 1.55–5.23). This impact translated into an increase in the prevalence of uptake of VMMC within six months from approximately 4 % to around 12 % of the eligible study sample (Table 5). A similar magnitude of impact was observed in the adjusted results of Table 7 for the uptake of CCS services among women entering FP and HTC sites in both intervention arms (Arm 2: AOR 2.76, 95 % CI: 1.94–3.91; Arm 3: AOR 2.98, 95 % CI: 2.10–4.22) (Table 7). This impact translated into an increase in the prevalence of uptake within six months from approximately 10 % to around 24 % of the eligible study sample (Table 5).

**Table 6:** Multivariable logistic regression results of impact of intervention on six-week service uptake outcomes

	Uptake of HTC			Uptake of FP			Uptake of VMMC			Uptake of CCS			Uptake of HIV C&T			Uptake of STI C&T		
	OR	CI	Sign.	OR	CI	Sign.	OR	CI	Sign.	OR	CI	Sign.	OR	CI	Sign.	OR	CI	Sign.
Model 1: Study arm																		
Study arm																		
Standard of care	1			1			1			1			1			1		
Enhanced referral	1.34	0.85–2.10		1.32	0.84–2.07		1.97	1.09–3.58	*	6.22	3.95–9.80	***	1.11	0.83–1.50		1.22	0.80–1.86	
Enhanced referral & escort	1.75	1.13–2.71	*	0.65	0.39–1.10		2.82	1.60–4.98	***	7.50	4.77–11.78	***	1.28	0.96–1.71	†	1.11	0.72–1.71	
N	875			791			1110			1713			3254			3258		
Chi-square	6.41		*	7.96		*	14.13		***	118.79		***	2.95			0.89		
Degrees of freedom	2			2			2			2			2			2		
Model 2: Study Arm + Site + Demographic Covariates																		
Study Arm	AOR	CI	Sign.	AOR	CI	sign.	AOR	CI	Sign.	AOR	CI	Sign.	AOR	CI	Sign.	AOR	CI	Sign.
Standard of care	1			1			1			1			1			1		
Enhanced referral	1.34	0.85–2.11		1.33	0.84–2.12		2.05	1.11–3.79	*	6.60	4.17–10.45	***	1.16	0.85–1.58		1.28	0.84–1.96	
Enhanced referral & escort	1.73	1.11–2.69	*	0.62	0.36–1.05	†	2.99	1.66–5.36	***	7.75	4.91–12.23	***	1.30	0.96–1.75	†	1.14	0.73–1.76	
Entry Site^a^																		
SFH HTC - Cairo Road				1			1			1			1			1		
Chawama Clinic - MCH	1									0.93	0.48–1.80		0.46	0.23–0.92	*	1.80	0.63–5.17	
Chawama Clinic - Out Patient Ward (VMMC)													0.75	0.32–1.75		1.53	0.50–4.70	
Kamwala Clinic - TB, STI & HIV Clinic				0.83	0.38–1.80		0.30	0.06–1.40		0.76	0.34–1.71		1.87	1.06–3.28	*	3.38	1.37–8.35	**
Kapata Urban Clinic, MCH	1.31	0.87–1.96								1.70	0.93–3.09	†	1.02	0.58–1.81		1.92	0.73–5.08	
Kapata Urban Clinic, TB, STI & HIV				1.04	0.53–2.04		1.59	0.71–3.53		2.18	1.19–3.97	*	0.64	0.37–1.12		1.88	0.79–4.45	
Chipata Gen Hosp - OP VMMC													1.03	0.43–2.45		1.29	0.39–4.27	
SFH New Start				1.06	0.53–2.12		3.41	1.62–7.19	**	0.83	0.42–1.63		2.75	1.68–4.51	***	4.00	1.77–9.05	***
SFH VMMC													1.37	0.45–4.18		1.34	0.27–6.71	
Gender^b^																		
Male													1			1		
Female													1.70	1.22–2.36	**	0.78	0.49–1.25	
Age	1.00	0.95–1.04		0.97	0.93–1.02		1.01	0.96–1.06		1.00	0.97–1.04		1.05	1.03–1.08	***	1.03	0.99–1.06	
Highest grade completed	1.00	0.94–1.06		0.97	0.91–1.04		0.99	0.91–1.08		1.00	0.96–1.05		0.93	0.89–0.96	***	1.02	0.96–1.08	
Marital Status																		
Not currently married	1			1			1			1			1			1		
Currently married/living with partner	0.85	0.53–1.34		2.57	1.61–4.08	***	1.26	0.63–2.52		1.28	0.91–1.78		0.94	0.70–1.26		0.73	0.48–1.12	
Number of children	1.00	0.84–1.18		1.10	0.93–1.30		0.91	0.73–1.14		1.00	0.89–1.12		1.02	0.93–1.12		1.07	0.93–1.23	
Residence																		
Urban	1			1			1			1			1			1		
Rural	0.53	0.20–1.42		1.66	0.63–4.36		1.02	0.33–3.15		1.10	0.57–2.10		0.93	0.54–1.60		1.01	0.47–2.19	
Employment status																		
Not working	1			1			1			1			1			1		
Currently working	1.38	0.94–2.03		1.13	0.75–1.72		0.58	0.35–0.96	*	1.38	1.04–1.83	*	1.19	0.91–1.54		1.04	0.71–1.51	
Number of household assets	0.94	0.86–1.03		1.08	0.97–1.20		1.18	1.05–1.33	**	0.93	0.87–1.00	*	0.98	0.92–1.05		0.91	0.83–0.99	*
Distance to health facility^c^	1.00	0.91–1.10		0.99	0.94–1.04		1.02	0.98–1.06		0.99	0.94–1.04		0.98	0.94–1.02		1.00	0.97–1.04	
N	865			783			1092			1695			3218			3222		
Chi-Square	15.70			36.68		***	56.89		***	172.07		***	199.87		***	46.67		***
Degrees of Freedom	12			14			14			16			20			20		

*OR* odds ratio, *AOR* adjusted odds ratio, *CI* 95 % confidence interval, *Sign* statistical significance of *p*-value

***p < .001; **p < .01; *p < .05; †*p* < .10

^a^Entry sites included in model dependent on outcome variable

^b^Gender of outcome variable constant if omitted from model

^c^38 % of baseline sample don’t know distance, therefore a dummy was also included (1 = Yes if don’t know distance)

**Table 7:** Multivariable logistic regression results of impact of intervention on six-month service uptake outcomes

	Uptake of HTC			Uptake of FP			Uptake of VMMC			Uptake of CCS			Uptake of HIV C&T			Uptake of STI C&T		
	OR	CI	Sign.	OR	CI	Sign.	OR	CI	Sign.	OR	CI	Sign.	OR	CI	Sign.	OR	CI	Sign.
Model 1: Study arm																		
Study arm																		
Standard of care	1			1			1			1			1			1		
Enhanced referral	1.36	0.95–1.94	†	0.77	0.51–1.17		1.45	0.75–2.79		2.67	1.89–3.77	***	1.21	0.89–1.63		0.90	0.60–1.37	
Enhanced referral & escort	1.30	0.91–1.87		0.93	0.61–1.40		2.89	1.60–5.25	***	2.89	2.05–4.07	***	1.27	0.94–1.71		0.99	0.66–1.50	
N	838			772			1110			1660			3204			3204		
Chi-square	3.33			1.53			14.79		***	47.42		***	2.66			0.28		
Degrees of freedom	2			2			2			2			2			2		
Model 2: Study Arm + Site + Demographic Covariates																		
Study Arm	AOR	CI	Sign.	AOR	CI	Sign.	AOR	CI	Sign.	AOR	CI	Sign.	AOR	CI	Sign.	AOR	CI	Sign.
Standard of care	1			1			1			1			1			1		
Enhanced referral	1.36	0.95–1.95	†	0.79	0.50–1.23		1.49	0.77–2.90		2.76	1.94–3.91	***	1.26	0.92–1.72		0.89	0.59–1.36	
Enhanced referral & escort	1.28	0.89–1.85		0.87	0.56–1.35		2.85	1.55–5.23	***	2.98	2.10–4.22	***	1.30	0.95–1.77	†	0.98	0.64–1.48	
Entry Site^a^																		
SFH HTC - Cairo Road				1			1			1			1			1		
Chawama Clinic - MCH	1									1.45	0.79–2.66		2.11	1.16–3.84	*	1.58	0.57–4.36	
Chawama Clinic - Out Patient Ward (VMMC)													1.06	0.45–2.49		1.55	0.56–4.28	
Kamwala Clinic - TB, STI & HIV Clinic				1.15	0.56–2.38		0.26	0.06–1.20	†	0.88	0.41–1.88		2.17	1.21–3.90	**	2.78	1.19–6.49	*
Kapata Urban Clinic, MCH	1.38	0.99–1.93	†							2.12	1.20–3.73	**	1.77	1.00–3.13	†	2.74	1.14–6.55	*
Kapata Urban Clinic, TB, STI & HIV				1.87	1.00–3.50	†	0.90	0.40–2.01		1.39	0.78–2.48		1.02	0.59–1.76		1.89	0.86–4.13	
Chipata Gen Hosp - OP VMMC													0.81	0.29–2.24		1.40	0.47–4.17	
SFH New Start				1.02	0.52–2.02		1.82	0.87–3.79		0.73	0.38–1.41		1.64	0.97–2.78	†	2.57	1.20–5.48	*
SFH VMMC													1.22	0.34–4.34		0.55	0.06–4.72	
Gender^b^																		
Male													1			1		
Female													1.65	1.15–2.35	**	0.93	0.58–1.48	
Age	1.02	0.99–1.06		0.96	0.92–1.00	*	0.99	0.94–1.05		1.03	1.00–1.06	*	1.09	1.06–1.11	***	1.04	1.00–1.08	*
Highest grade completed	1.02	0.97–1.07		1.09	1.02–1.17	*	0.99	0.90–1.08		1.02	0.98–1.06		0.95	0.92–0.99	**	0.97	0.92–1.03	
Marital Status																		
Not currently married	1			1			1			1			1			1		
Currently married/living with partner	0.73	0.49–1.08		4.14	2.69–6.35	***	0.52	0.24–1.14		1.1	0.80–1.52		0.89	0.66–1.20		0.73	0.48–1.11	
Number of children	1.00	0.87–1.15		1.17	1.00–1.37	*	1.01	0.78–1.30		0.99	0.89–1.10		0.97	0.89–1.07		0.92	0.80–1.07	
Residence																		
Urban	1			1			1			1			1			1		
Rural	0.80	0.39–1.66		1.82	0.68–4.87		0.30	0.04–2.25		1.23	0.67–2.24		0.60	0.32–1.11		0.78	0.35–1.76	
Employment status																		
Not working	1			1			1			1			1			1		
Currently working	1.06	0.77–1.47		0.95	0.65–1.40		0.76	0.45–1.28		1.19	0.90–1.56		1.08	0.83–1.41		1.28	0.88–1.86	
Number of household assets	0.99	0.91–1.06		0.98	0.89–1.08		1.10	0.97–1.24		0.94	0.88–1.00	†	0.96	0.90–1.03		0.91	0.83–0.99	*
Distance to health facility^c^	0.98	0.90–1.05		1.01	0.97–1.06		1.00	0.96–1.05		1.00	0.95–1.05		1.01	0.99–1.04		1.03	1.00–1.05	†
N	828			764			1096			1642			3172			3172		
Chi-Square	14.84			83.28		***	47.69		***	96.20		***	170.18		***	35.30		*
Degrees of Freedom	12			14			14			16			20			20		

*OR* Odds Ratio, *AOR* Adjusted Odds Ratio, *CI* 95 % Confidence Interval, *Sign* Statistical Significance of *p*-value

***p < .001; **p < .01; *p < .05; †*p* < .10

^a^Entry sites included in model dependent on outcome variable

^b^Gender of outcome variable constant if omitted from model

^c^38 % of baseline sample don’t know distance, therefore a dummy was also included (1 = Yes if don’t know distance)

The adjusted results in Table 7 also revealed significant differences in referral completions at the different study sites among clients with different baseline demographic characteristics; yet, the primary impact results of the intervention remained largely the same when site-fixed effects and covariates were introduced. For instance, the adjusted results in Table 6 show that men who were employed at baseline and referred for VMMC services had 42 % lower odds (AOR 0.58, 95 % CI: 0.35–0.96) of accessing services within six weeks. The impact of employment on VMMC uptake did reduce over time, to 24 % lower odds at six months (Table 7 lower panel) and lost statistical significance, suggesting that employed men were ultimately able to adjust their schedules to accommodate their circumcision plans. The importance of employment status on VMMC uptake has been demonstrated in previous research [37] and been the focus of studies that have addressed the opportunity and other costs associated with the uptake of circumcision services [38, 39]. The adjusted results in Table 7 also indicated that females were significantly more likely to report accessing HIV care and treatment than were their male counterparts and this effect was persistent over time. Women had 65 % greater odds within six months of reporting access to HIV services than were males (AOR 1.65, 95 % CI: 1.15–2.35). Older participants were also significantly more likely to take up HIV care and treatment (AOR 1.09, 95 % CI: 1.06–1.11), as were participants with a lower number of completed years of education (AOR 0.95, 95 % CI: 0.92–0.99).

### Economic evaluation

Table 8 summarizes costs per client at the vertical and integrated service sites in the two study provinces. The fully integrated comparator site (column 3) appeared to operate with lower unit costs than the vertical SFH-operated urban and rural sites for HTC and VMMC. Differences between the comparator site and the SFH-operated vertical sites were driven by the largely fixed costs of human resources, followed by the costs of administration and day-to-day operations (overhead) and general supplies [33]. These costs could potentially be spread over a larger number of clients by integrating services, thus lowering the average costs of service delivery and achieving economies of scale in the long run. For family planning services, in contrast to the others, the comparator site did not operate with lower unit costs than the vertical urban site, although it was significantly more technically efficient than the vertical rural site. The low estimated unit costs for the vertical urban site were likely a signal of human and material resource shortages and drug stock outs at government facilities rather than of higher technical efficiency at similar client volumes.

**Table 8:** Cost per client of vertical services compared to integrated provision, 2013 USD

	Cost per client		
	Vertical urban site	Vertical rural site	Integrated comparator site
HIV testing and counseling	$121	$118	$112
Voluntary medical male circumcision (VMMC)	$76	$31	$25
IUD insertion	$9	$142	$16
Implant insertion	$8	$134	$14
Post-partum IUD	$8	$135	$15
Other referral services^a^			
Cervical cancer screening	$18		
CD4 count	$7		
Tuberculosis testing	$8		
Antiretroviral therapy	$10		
STI testing	$8		

^a^Cost per consultation, excluding any patient-specific, variable costs such as drugs and medical supplies as the trial did not provide access to client medical records. These costs were not considered variable for HTC, VMMC and FP services as all clients receive the same service with standard quantities of medical consumables. Hence, other referral services were thus not included in the technical efficiency analysis

Table 9 provides a summary of the cost-effectiveness of the study intervention. For HIV/AIDS, only the third study arm showed a reduction in the projected number of deaths, while both intervention arms showed a reduction in the years lost to disability from HIV/AIDS. For instance, it is expected that if the intervention arm three were scaled, there would be 452 deaths averted from HIV/AIDS per 1000 clients if an escort were provided to facilitate linkages between services. Also, it would be expected that 12,826 and 31,718 DALYs would be averted per 1000 clients for each of the study arms respectively. A reduction in the projected number of deaths per 1000 clients was also evident for CCS and the number of DALYs averted by CCS was between 5000 and 6000 per 1000 clients. In both cases, estimates of the impact of the interventions within the population indicated a significant reduction in mortality and morbidity due to HIV/AIDS and cervical cancer.

**Table 9:** Summary of cost-effectiveness results, by study arm

	Effectiveness, per 1000 clients				Costs per DALY averted, US$			
	HIV/AIDS		Cervical cancer					
	Deaths	DALYs	Deaths	DALYs	HTC	VMMC	HIV C&T	CCS
Study arm								
SOC	4160	96796	3765	83966	—	—	—	
Arm 2	4240	83970	3350	78808	$ 7890	$ 377	$ 81	$ 607
Arm 3	3708	65078	3254	77741	$ 3180	$ 162	$ 33	$ 106
Difference	Deaths averted	DALYs	Deaths averted	DALYs				
SOC – Arm2	−80	12826	415	5157				
SOC – Arm 3	452	31718	511	6224				

Note: Family planning not included in the incremental cost-effectiveness calculations as the intervention did not increase uptake of services

Based on the World Health Organization (WHO), a health intervention is considered cost-effective if its costs per DALY averted is less than three times the country’s GDP per capita [40]; for Zambia, that would amount to $1845 × 3 = $5535 based on 2014 GDP per capita estimates [41]. Given that formula, intervention arm three was cost-effective for HTC, with a cost of $3180 per DALY averted. Intervention arm two was not considered cost-effective for HTC based on the WHO criteria. The results for VMMC, HIV care and treatment and cervical cancer screening indicate that both intervention arms were highly cost-effective. For CCS in particular, this result was driven both by the magnitude of impact of the intervention and the relatively low total cost of implementing CCS services.

## Discussion

The study was an implementation science randomized evaluation of interventions to improve the uptake of many critical HIV and sexual and reproductive health services in the Zambian context. The study’s rationale was that providing enhanced client health services directed toward lowering the barriers of access and improving quality would be a cost-effective way to improve the uptake of FP/HIV services. The study’s strongest findings were that the interventions improved the uptake of VMMC and CCS services among clients. Given the invasiveness of the circumcision procedure and that increasing the demand for VMMC services is difficult due to a multiplicity of barriers, the study’s results suggest a promising opportunity to enhance uptake. The provision of an escort to the VMMC site appeared to be an important ingredient in increasing the odds of clients accessing these services. These findings reinforce conclusions found elsewhere that men need more information about what the circumcision procedure involves to overcome psychological barriers to uptake [37].

Integrating CCS as an add-on service to other sexual and reproductive service provision is shown to be a highly cost-effective method of increasing the uptake of screening and in reducing disability and deaths from cervical cancer. The study results indicated that women receiving an enhanced package of counseling, referral and follow-up services had approximately three times higher odds of getting screened for cervical cancer than women receiving standard services. Given the fact that there was little meaningful difference between the two intervention arms in increasing uptake of screening suggests that provision of high-quality counseling and information services was the core driver in increasing screening uptake.

While the impact of the interventions was less consistent with regard to HIV care and treatment options, there were indications at the six-month interview that clients in the intervention arm had significantly higher uptake of important HIV care and treatment outcomes, specifically TB testing and the initiation of ART. Further research that allows for a more focused recruitment and larger sample sizes is warranted to investigate the impact of similar interventions on HIV-positive clients.

Finally, the economic evaluation’s technical efficiency analysis showed that the integrated comparator site was able to provide HTC and VMMC services at a lower cost per client than the segmented, vertical sites. These results lend further support to the argument for increasing integration of HIV services. Integrated sites might not only increase uptake of VMMC, but also provide it at a lower cost per client. Additionally, although the intervention did not significantly increase HTC uptake in the long-term, results showed that there were potential cost savings at integrated sites. This conclusion is in line with a study in Kenya and Swaziland by Obure and colleagues [42], who found cost complementarities between HIV and sexual and reproductive health services and concluded that efficiency gains are most likely achievable in settings of low-scale service delivery, with high levels of fixed costs. This may be an argument for the integration of HIV prevention services and consideration of their integration with SRH services.

### Strengths and limitations

The study presented here benefitted from a design in which clients were randomly assigned to the study intervention arms. This design allowed for greater confidence in the ITT estimates of the intervention’s impact on study outcomes and inferences drawn from the results. The study used existing government and nongovernment clinics, setting the evaluation in a real-world context. Furthermore, the study benefited from an embedded economic evaluation that provided an assessment of how services could be provided efficiently, as well as the expected cost-effectiveness of the intervention.

One significant limitation of the study and the results was that little could be said about which of the common components of the intervention contributed to the overall impact. The study would have been more informative if a treatment-on-the-treated (TOT) analysis had been possible focused on clients who had actually received a referral, received a follow-up call, or taken up the offer of an escort. This limitation derived not from an issue of design, but was due to data quality issues with the standard-of-care arm. The client tracking data for referrals and uptake of services was not properly recorded for the standard-of-care arm, discounting possible comparisons with the intervention arms [33]. These issues had no bearing on the behavioral survey data which were collected separately.

Caution is also required in interpreting the results of the economic evaluation as the technical efficiency assessment was carried out on existing SFH referral networks and is therefore specific to the way these are structured. The study service sites were not randomly selected and the comparison necessarily included facilities with different ownership structures. Although we did consistently observe lower costs of operation at the integrated service site, the extent to which our findings can be extrapolated to other contexts is limited by the nature of the program evaluation.

## Conclusions

This study was a randomized evaluation of two intervention approaches to improve the linkage of clients to additional health services as compared to the standard of care in Zambia. The study’s findings indicated that enhanced client add-on service referral and follow-up, with and without an escort to the add-on service, improved the uptake of many, but not all, of the services targeted by the study. The results indicated that the interventions had the greatest impact on improving the uptake of VMMC and CCS services among clients, while revealing more limited effects on HIV care and treatment outcomes. There were no effects found for increasing the uptake of family planning. The embedded economic evaluation found the intervention to be highly cost-effective for HTC (study arm 3 only), VMMC, HIV care and treatment and for cervical cancer screening. The study’s impact and cost-effectiveness results suggest that the enhanced service models evaluated are worthy of strong consideration when adding or integrating health services across platforms.

## Abbreviations

ACASI, audio computer-assisted self-interview; ART, antiretroviral therapy; C&T, care and treatment; CCS, cervical cancer screening; CI, confidence interval; CTD, client tracking database; DALYs, disability-adjusted life years; FP, family planning; GEE, generalized estimating equations; HTC, HIV testing and counseling; ITT, intent-to-treat; MCH, maternal and child health; MI, motivational interviewing; NGO, Non-Governmental Organization; SFH, society for family health; SRH, sexual and reproductive health; STI, sexually transmitted infection; TB, tuberculosis; TOT, treatment-on-the-treated; USAID, United States Agency for International Development; VMMC, voluntary medical male circumcision; WHO, World Health Organization

## Additional files

Additional file 1: De-identified REACH Study Data. This file contains study data, including de-identified data from the client information and tracking registry, the baseline interview, the six-week interview and the six-month interview. (CSV 8081 kb) Additional file 2: REACH Study Data Codebook. This file contains the codebook of the data, including the variable type, values, frequency distribution and missing values. (PDF 1392 kb)

## References

[1] USAID (2003). Family planning/HIV integration: technical guidance for usaid-supported field programs.

[2] WHO (2009). Strengthening linkages between sexual and reproductive health and HIV. Bull World Health Organ.

[3] Central Statistical Office (CSO) [Zambia], Ministry of Health (MOH) [Zambia], ICF International: Zambia demographic and health survey 2013-14. Rockville, MD: Central Statistical Office, Ministry of Health, and ICF International; 2014.

[4] Ministry of Health of Zambia (2011). National health strategic plan 2012–2015.

[5] Ministry of Health of Zambia (2011). National HIV/AIDS prevention strategy 2012–2015.

[6] Strachan M, Kwateng-Addo A, Harde K, Subramaniam S, Judice N, Agarwal K. An analysis of family planning content in HIV/AIDS, VCT, and PMTCT policies in 16 countries. Washington, D.C.: Futures Group International; 2004.

[7] Rutenberg N, Biddlecom A, Kaona F (2000). Reproductive decision-making in the context of HIV and AIDS: a qualitative study in Ndola, Zambia. Int Fam Plan Perspect.

[8] Chanda E, Masaninga F, Coleman M, Sikaala C, Katebe C, MacDonald M (2008). Integrated vector management: the Zambian experience. Malar J.

[9] Stekelenburg JKS, Mukelabai M, Wolffers I, van Roosmalen J (2004). Waiting too long: low use of maternal health services in Kalabo, Zambia. Trop Med Int Health.

[10] MacKeith N, Chinganya O, Ahmed Y, Murray S (2003). Zambian women’s experiences of urban maternity care: results from a community survey in Lusaka. Afr J Reprod Health.

[11] Gabrysch S, Campbell O (2009). Still too far to walk: literature review of the determinants of delivery service use. BMC Pregnancy Childbirth.

[12] Mayhew S, Lush L, Cleland J, Walt G (2001). Implementing the integration of component services for reproductive health. Stud Fam Plann.

[13] Ministry of Health of Zambia (2009). Workforce optimization analysis: optimal healthcare worker allocation for healthcare facilities across Zambia.

[14] Central Statistical Office (CSO), Ministry of Health (MOH), Tropical Diseases Research Centre (TDRC), University of Zambia, Macro International Inc. Zambian demographic and health survey 2007. Calverton, MD: CSO and Macro International; 2009.

[15] ZNAC. Zambia HIV prevention response and modes of transmission analysis. Epi-synthesis final report July 2009. Lusaka: National HIV/AIDS/TB Council of Zambia, CDC Global HIV/AIDS Program, World Bank, and UNAIDS; 2009.

[16] WHO, UNFPA, UNAIDS, IPPF. Sexual and reproductive health and HIV/AIDS: A framework for priority linkages. WHO/HIV/2005.05. Geneva: WHO; 2005.

[17] Chikamata D, Chinganya O, Jones H, Ramarao S (2002). Dual needs: contraceptive and sexually transmitted infection protection in Lusaka, Zambia. Int Fam Plan Perspect.

[18] Foreit K, Hardee K, Agarwal K (2002). When does it make sense to consider integrating STI and HIV services with family planning services? Issues in perspective: integrating HIV/STI and family planning services. Int Fam Plan Perspect.

[19] Lush L, Walt G, Cleland J, Mayhew S (2001). The role of MCH and family planning services in HIV/STD control: Is integration the answer?. African Journal of Reproductive Health / La Revue Africaine de la Santé Reproductive.

[20] Shelton J, Fuchs N (2004). Opportunities and pitfalls in integration of family planning and HIV prevention efforts in developing countries. Public Health Rep.

[21] Mullick S, Khoza D, Askew I, Maluka T, Menziwa M. Integrating counseling and testing into family planning services: What happens to the existing quality of family planning when HIV services are integrated in South Africa? Presentation at Linking Reproductive Health, Family Planning, and HIV/AIDS in Africa Conference. Addis Ababa: Organized by Johns Hopkins University Bloomberg School of Public Health, Bill and Melinda Gates Institute for Population and Reproductive Health, and Department of Community Health at Addis Ababa University; 2006.

[22] Scott P, Becker J (1995). HIV prevention and family planning: integration improves client services in Jamaica. Aidscaptions.

[23] Stover J, Fuchs N, Halperin D, Gibbons A, Gillespie D (2003). Adding family planning to PMTCT sites increases the benefits of PMTCT. Population and reproductive health issue brief.

[24] Peck R, Fitzgerald D, Liautaud B, Deschamps M, Verdier R, Beaulieu M (2003). The feasibility, demand, and effect of integrating primary care services with HIV voluntary counseling and testing: evaluation of a 15-year experience in Haiti, 1985–2000. J Acquir Immune Defic Syndr.

[25] Boonstra H (2006). The role of reproductive health providers in preventing HIV.

[26] Rasch V, Yambesi F, Massawe S (2006). Post-abortion care and voluntary HIV counselling and testing: an example of integrating HIV prevention into reproductive health services. Trop Med Int Health.

[27] Coyne KM, Hawkins F, Desmond N (2007). Sexual and reproductive health in HIV-positive women: a dedicated clinic improves service. Int J STD AIDS.

[28] Dilorio C, McCarty F, Resnicow K, McDonnell HM, Soet J, Yeager K (2008). Using motivational interviewing to promote adherence to antiretroviral medications: a randomized controlled study. AIDS Care.

[29] Lundahl B, Kunz C, Brownell C, Tollefson D, Burke B (2010). A meta-analysis of motivational interviewing: twenty-five years of empirical studies. Research on Social Work Practice.

[30] Golin C, Earp J, Tien H, Stewart P, Porter C, Howie L (2006). A 2-arm, randomized, controlled trial of a motivational interviewing-based intervention to improve adherence to antiretroviral therapy (ART) among patients failing or initiating ART. J Acquir Immune Defic Syndr.

[31] Gupta SK (2011). Intention-to-treat concept: a review. Perspect Clin Res.

[32] StataCorp (2015). Stata: Release 14. Statistical software.

[33] Hewett PC, Mutinta N, Bozzanni F, Dennis M, Digitale J, Vu L (2015). REacH: randomized evaluation of HIV/FP service models final study report.

[34] Guinness L, Wiseman V (2011). Introduction to health economics (understanding public health).

[35] Murray CJL, Lopez AD (1996). Evidence-based health policy: lessons from the Global Burden of Disease Study. Science.

[36] Salmon JATV, Hogan DR (2010). Common values in assessing health outcomes from disease and injury: disability weights measurement study for the Global Burden of Disease Study. Lancet 2012.

[37] Price JE, Phiri L, Mulenga D, Hewett PC, Topp SM, Shiliya N, et al. Behavior change pathways to voluntary medical male circumcision: Narrative interviews with circumcision clients in Zambia. PLoS ONE 9(11): e111602. 10.1371/journal.pone.0111602 PMC4222873 25375790

[38] Thirumurthy H, Masters SH, Rao S, Bronson MA, Lanham M, Omanga E (2014). Effect of providing conditional economic compensation on uptake of voluntary medical male circumcision in Kenya. JAMA.

[39] Thirumurthy H, Masters SH, Rao S, Murray K, Omanga E, Agot K. The effect of conditional economic compensation and lottery-based rewards on uptake of medical male circumcision in Kenya: A randomized trial. Paper presented at 8th International AIDS Society Conference on HIV Pathogenesis, Treatment & Prevention: July 19–22, 2015. Vancouver; 2015.

[40] WHO. Cost-effectiveness and strategic planning (WHO-CHOICE). Table: Threshold values for intervention cost-effectiveness by region. Geneva. http://www.who.int/choice/costs/CER_levels/en. Accessed 8 Aug 2016.

[41] World Bank. World development indicators - Zambia. Washington DC. http://data.worldbank.org/country/zambia. Accessed 21 Aug 2015.

[42] Obure CD, Guinness L, Sweeney S, Vassall A, Integra Initiative (2015). Does integration of HIV and SRH services achieve economies of scale and scope in practice? A cost function analysis of the Integra Initiative. Sex Transm Infect.

[43] UNAIDS (2014). Zambia country report monitoring the declaration of commitment on HIV and AIDS and the universal access.

[44] Lozano R, Naghavi M, Foreman K, Lim S, Shibuya K, Aboyans V (2012). Global and regional mortality from 235 causes of death for 20 age groups in 1990 and 2010: a systematic analysis for the Global Burden of Disease Study 2010. Lancet.

[45] Allen S, Kilembe W, Inambao M (2014). Couples’ voluntary HIV counseling and testing (CVCT) followed by treatment as prevention (TasP) for discordant couples: The impact of each step. AIDS Res Hum Retroviruses.

[46] Sankaranarayanan R, Ferlay J (2006). Worldwide burden of gynaecological cancer: the size of the problem. Best Pract Res Clin Obstet Gynaecol.

[47] Mandelblatt JS, Lawrence WF, Womack SM, Jacobson D, Yi B, Hwang YT (2002). Benefits and costs of using HPV testing to screen for cervical cancer. JAMA.

