# Supplementary material for: Randomized evaluation and cost-effectiveness of HIV and sexual and reproductive health service referral and linkage models in Zambia
Source: BMC Public Health. 2016 Aug 12;16:785. doi: 10.1186/s12889-016-3450-x (PMC4983050; doi:10.1186/s12889-016-3450-x)
Supplement: Additional file 2: — REACH Study Data Codebook. This file contains the codebook of the data, including the variable type, values, frequency distribution and missing values. (PDF 1392 kb) [file 12889_2016_3450_MOESM2_ESM.pdf]

```
Dataset:  */REacH/Datasets/REacH_StudyData_Deidentified_2015-10-28.dta
            Last saved:  28 Oct 2015 16:23

                 Label:  [none]
   Number of variables:  931
Number of observations:  4,168
                  Size:  13,275,080 bytes ignoring labels, etc.

_dta:
  1.  This data file was generated from REacH_ClientData_2015-10-28 and
      REacH_SurveyData_Merged_2015-10-28 on 2015-10-28. The data were generated from
      REacH_8_ClientSurvey_Merge.do.

-----------------------------------------------------------------------------------------------
sid                                                                                         sid
-----------------------------------------------------------------------------------------------

                  type:  string (str9)

         unique values:  4168                     missing "":  0/4168

              examples:  "02-0303"
                         "06-0019"
                         "07-0310"
                         "11-0197"

-----------------------------------------------------------------------------------------------
armID                                                                                 Study Arm
-----------------------------------------------------------------------------------------------

                  type:  numeric (byte)
                 label:  armID

                 range:  [1,3]                        units:  1
         unique values:  3                        missing .:  0/4168
       unique mv codes:  1                       missing .*:  205/4168

            tabulation:  Freq.   Numeric  Label
                          1319         1  1. Standard of Care
                          1323         2  2. Comprehensive Referrals with
                                          Follow-Up
                          1321         3  3. Integrated Services Model
                           205        .j  .j. Ineligible: External site
                                          client

-----------------------------------------------------------------------------------------------
siteID                                                                        Registration Site
-----------------------------------------------------------------------------------------------

                  type:  numeric (byte)
                 label:  site

                 range:  [1,13]                       units:  1
         unique values:  9                        missing .:  0/4168
       unique mv codes:  1                       missing .*:  205/4168

            tabulation:  Freq.   Numeric  Label
                           557         1  1. SFH HCT - Cairo Road
                           489         2  2. Chawama Clinic - MCH
                           245         3  3. Chawama Clinic - Out Patient
                                          Ward (VMMC)
                           362         5  5. Kamwala Clinic - TB, STI &
                                          HIV Clinic
                           545         6  6. Kapata Urban Clinic, MCH
                           775         7  7. Kapata Urban Clinic, TB, STI
                                          & HIV
                           189         8  8. Chipata Gen Hosp - OP VMMC
                           713        11  11. SFH New Start
                            88        13  13. SFH VMMC
                           205        .j  .j. Ineligible: External site
```

```
                                    client

-------------------------------------------------------------------------------
SiteProv                                           Registration Site Province
-------------------------------------------------------------------------------

                  type:  numeric (byte)
                 label:  province

                 range:  [3,5]                        units:  1
         unique values:  2                        missing .:  0/4168
       unique mv codes:  1                       missing .*:  205/4168

            tabulation:  Freq.   Numeric  Label
                          2310         3  3. EASTERN
                          1653         5  5. LUSAKA
                           205        .j  .j. Ineligible: External site
                                          client

-------------------------------------------------------------------------------
Gender                                                                   Gender
-------------------------------------------------------------------------------

                  type:  numeric (byte)
                 label:  Gender

                 range:  [0,1]                        units:  1
         unique values:  2                        missing .:  0/4168

            tabulation:  Freq.   Numeric  Label
                          2033         0  0. Male
                          2135         1  1. Female

-------------------------------------------------------------------------------
MedRecordType                                               Medical Record Type
-------------------------------------------------------------------------------

                  type:  numeric (byte)
                 label:  MedRecordType

                 range:  [0,1]                        units:  1
         unique values:  2                        missing .:  2/4168
       unique mv codes:  2                       missing .*:  205/4168

            tabulation:  Freq.   Numeric  Label
                          2603         0  0. GOV
                          1358         1  1. SFH
                             2         .
                           205        .j  .j. Ineligible: External site
                                          client

-------------------------------------------------------------------------------
RegDate_sys                                            System Registration Date
-------------------------------------------------------------------------------

                  type:  numeric (double)

                 range:  [1.703e+12,1.731e+12]        units:  1000
         unique values:  3959                     missing .:  3/4168
       unique mv codes:  2                       missing .*:  205/4168

                  mean:   1.7e+12
              std. dev:   6.5e+09

           percentiles:        10%       25%       50%       75%       90%
                           1.7e+12   1.7e+12   1.7e+12   1.7e+12   1.7e+12

-------------------------------------------------------------------------------
RegDate                                                       Registration Date
-------------------------------------------------------------------------------
```

```
                 type:  numeric daily date (int)

                range:  [19709,20037]                units:  1
       or equivalently:  [17dec2013,10nov2014]        units:  days
         unique values:  242                      missing .:  0/4168
       unique mv codes:  1                       missing .*:  205/4168

                 mean:   19866.5 = 23may2014 (+ 12 hours)
             std. dev:   75.2411

          percentiles:        10%       25%       50%       75%       90%
                            19771     19807     19862     19922     19974
                        17feb2014 25mar2014 19may2014 18jul2014 08sep2014

-----------------------------------------------------------------------------------------------
RegTime                                                                       Registration Time
-----------------------------------------------------------------------------------------------

                 type:  string (str5)

         unique values:  463                      missing "":  206/4168

             examples:  "09-42"
                        "10-33"
                        "11-27"
                        "12-37"

-----------------------------------------------------------------------------------------------
Province                                                                  Province of residence
-----------------------------------------------------------------------------------------------

                 type:  numeric (byte)
                label:  province

                range:  [3,10]                       units:  1
         unique values:  6                        missing .:  0/4168
       unique mv codes:  1                       missing .*:  205/4168

           tabulation:  Freq.   Numeric  Label
                         2305         3  3. EASTERN
                         1646         5  5. LUSAKA
                            6         6  6. LUAPULA
                            2         8  8. MACHINGA
                            3         9  9. CENTRAL
                            1        10  10. COPPERBELT
                          205        .j  .j. Ineligible: External site
                                         client

-----------------------------------------------------------------------------------------------
District                                                                  District of residence
-----------------------------------------------------------------------------------------------

                 type:  numeric (byte)
                label:  district

                range:  [1,9]                        units:  1
         unique values:  8                        missing .:  0/4168
       unique mv codes:  1                       missing .*:  205/4168

           tabulation:  Freq.   Numeric  Label
                            1         1  1. CHADIZA
                            5         2  2. CHAMA
                         2303         3  3. CHIPATA
                            1         4  4. CHONGWE
                            1         5  5. KAFUE
                            1         7  7. LUANGWA
                            6         8  8. LUNDAZI
                         1645         9  9. LUSAKA
                          205        .j  .j. Ineligible: External site
```

```
                                    client

-----------------------------------------------------------------------------------------------
ConsentCompleted                                                              Consent Completed
-----------------------------------------------------------------------------------------------

                  type:  numeric (byte)
                 label:  yesno

                 range:  [1,1]                        units:  1
         unique values:  1                        missing .:  0/4168
       unique mv codes:  1                       missing .*:  205/4168

            tabulation:  Freq.   Numeric  Label
                          3963         1  1. Yes
                           205        .j  .j. Ineligible: External site
                                          client

-----------------------------------------------------------------------------------------------
BaseLineIntrCompleted                                              Baseline Interview Completed
-----------------------------------------------------------------------------------------------

                  type:  numeric (byte)
                 label:  yesno

                 range:  [0,1]                        units:  1
         unique values:  2                        missing .:  0/4168
       unique mv codes:  1                       missing .*:  205/4168

            tabulation:  Freq.   Numeric  Label
                             5         0  0. No
                          3958         1  1. Yes
                           205        .j  .j. Ineligible: External site
                                          client

-----------------------------------------------------------------------------------------------
Notes                                                                                     Notes
-----------------------------------------------------------------------------------------------

                  type:  string (str244)

         unique values:  178                      missing "":  3936/4168

              examples:  ""
                         ""
                         ""
                         ""

               warning:  variable has leading, embedded, and trailing blanks

-----------------------------------------------------------------------------------------------
Assigned                                                                  Assigned to Study Arm
-----------------------------------------------------------------------------------------------

                  type:  numeric (byte)
                 label:  yesno

                 range:  [0,1]                        units:  1
         unique values:  2                        missing .:  0/4168
       unique mv codes:  1                       missing .*:  205/4168

            tabulation:  Freq.   Numeric  Label
                             1         0  0. No
                          3962         1  1. Yes
                           205        .j  .j. Ineligible: External site
                                          client

-----------------------------------------------------------------------------------------------
AssignedDate_sys                                                     System Randomization Date
-----------------------------------------------------------------------------------------------
```

```
                  type:  numeric (double)

                 range:  [1.703e+12,1.731e+12]        units:  1000
         unique values:  3959                     missing .:  3/4168
       unique mv codes:  2                       missing .*:  205/4168

                  mean:   1.7e+12
              std. dev:   6.5e+09

           percentiles:        10%       25%       50%       75%       90%
                           1.7e+12   1.7e+12   1.7e+12   1.7e+12   1.7e+12

------------------------------------------------------------------------------------------------
OtherPeople                                                    # Accompanying People Registered
------------------------------------------------------------------------------------------------

                  type:  numeric (byte)

                 range:  [0,1]                        units:  1
         unique values:  2                        missing .:  0/4168
       unique mv codes:  1                       missing .*:  205/4168

            tabulation:  Freq.  Value
                          3947  0
                            16  1
                           205  .j

------------------------------------------------------------------------------------------------
personID1                                                      ID of Person Accompanying Client
------------------------------------------------------------------------------------------------

                  type:  numeric (long)

                 range:  [1020,85004]                 units:  1
         unique values:  16                       missing .:  3947/4168
       unique mv codes:  2                       missing .*:  205/4168

            tabulation:  Freq.  Value
                             1  1020
                             1  53008
                             1  61001
                             1  61002
                             1  61003
                             1  61004
                             1  62001
                             1  62002
                             1  62003
                             1  64001
                             1  73001
                             1  73002
                             1  85001
                             1  85002
                             1  85003
                             1  85004
                          3947  .
                           205  .j

------------------------------------------------------------------------------------------------
PersonGender1                                              Gender of Person Accompanying Client
------------------------------------------------------------------------------------------------

                  type:  numeric (byte)
                 label:  Gender

                 range:  [0,1]                        units:  1
         unique values:  2                        missing .:  3947/4168
       unique mv codes:  2                       missing .*:  205/4168

            tabulation:  Freq.   Numeric  Label
```

```
                         7         0  0. Male
                         9         1  1. Female
                      3947         .
                       205        .j  .j. Ineligible: External site
                                      client

-----------------------------------------------------------------------------------------------
PersHaveNRC1                                               Person Accompanying Client has NRC
-----------------------------------------------------------------------------------------------

                  type:  numeric (byte)
                 label:  yesno

                 range:  [0,1]                        units:  1
         unique values:  2                        missing .:  3947/4168
       unique mv codes:  2                       missing .*:  205/4168

            tabulation:  Freq.   Numeric  Label
                            13         0  0. No
                             3         1  1. Yes
                          3947         .
                           205        .j  .j. Ineligible: External site
                                          client

-----------------------------------------------------------------------------------------------
PersRealship1                                     Client's Relationship to Person Accompanying
-----------------------------------------------------------------------------------------------

                  type:  numeric (byte)
                 label:  relation

                 range:  [1,12]                       units:  1
         unique values:  3                        missing .:  3947/4168
       unique mv codes:  2                       missing .*:  205/4168

            tabulation:  Freq.   Numeric  Label
                            13         1  1. SPOUSE
                             1         3  3. DAUGHTER
                             2        12  12. OTHER NON-RELATIVE
                          3947         .
                           205        .j  .j. Ineligible: External site
                                          client

-----------------------------------------------------------------------------------------------
Reg1_ID                                                                      Registration 1 ID
-----------------------------------------------------------------------------------------------

                  type:  numeric (float)

                 range:  [265,86024]                  units:  1
         unique values:  3963                     missing .:  0/4168
       unique mv codes:  1                       missing .*:  205/4168

                  mean:   55653.2
              std. dev:   16198.1

           percentiles:        10%       25%       50%       75%       90%
                             44367     47666     56042     67121     75012

-----------------------------------------------------------------------------------------------
Reg1_PersID                                Registration 1: Respondent or Person Accompanying
-----------------------------------------------------------------------------------------------

                  type:  numeric (byte)
                 label:  RegPersID

                 range:  [0,0]                        units:  1
         unique values:  1                        missing .:  0/4168
       unique mv codes:  1                       missing .*:  205/4168
```

```
          tabulation:  Freq.   Numeric  Label
                        3963         0  0. Respondent
                         205        .j  .j. Ineligible: External site
                                        client

-------------------------------------------------------------------------------------------------
Reg1_Counseling                                           Registration 1: Counseling Received
-------------------------------------------------------------------------------------------------

                type:  numeric (byte)
               label:  Counseling

               range:  [1,4]                        units:  1
       unique values:  4                        missing .:  107/4168
     unique mv codes:  2                       missing .*:  205/4168

          tabulation:  Freq.   Numeric  Label
                         118         1  1. GROUP
                        3728         2  2. ONE-ON-ONE
                           4         3  3. BOTH
                           6         4  4. NONE
                         107         .
                         205        .j  .j. Ineligible: External site
                                        client

-------------------------------------------------------------------------------------------------
Reg1_Escort                                                  Registration 1: Escort Provided
-------------------------------------------------------------------------------------------------

                type:  numeric (byte)
               label:  Reg1_Escort

               range:  [0,1]                        units:  1
       unique values:  2                        missing .:  0/4168
     unique mv codes:  3                       missing .*:  3184/4168

          tabulation:  Freq.   Numeric  Label
                         290         0  0. No
                         694         1  1. Yes
                        2642        .a  .a. Not in integrated arm
                         337        .b  .b. Integrated arm, no referrals
                         205        .j  .j. Ineligible: External site
                                        client

-------------------------------------------------------------------------------------------------
Reg1_EscortTime                                               Registration 1: Time of Escort
-------------------------------------------------------------------------------------------------

                type:  string (str5)

       unique values:  318                      missing "":  3474/4168

            examples:  ""
                       ""
                       ""
                       ""

-------------------------------------------------------------------------------------------------
Reg1_ServCameFor1                                          Registration 1: Service 1 Came For
-------------------------------------------------------------------------------------------------

                type:  numeric (int)
               label:  Service

               range:  [3,1000]                     units:  1
       unique values:  10                       missing .:  0/4168
     unique mv codes:  1                       missing .*:  205/4168

          tabulation:  Freq.   Numeric  Label
                           4         3  3. FP: INDIVIDUAL COUNSELING
```

```
                    1028         4  4. FP: LONG TERM METHODS
                                    PROVIDED
                       3         5  5. FP: SHORT TERM METHODS
                                    PROVIDED
                      21         6  6. HIV: HTC - COUPLES
                    2378         7  7. HIV: HTC - INDIVIDUAL
                       1         8  8. HIV: ARV PROVISION
                       1         9  9. HIV: CD4 TESTING
                       1        14  14. VMMC: COUNSELING ONLY
                     520        15  15. VMMC: PROCEDURE
                       6      1000  1000. NO ADDITIONAL SERVICE
                     205        .j  .j. Ineligible: External site
                                    client

-----------------------------------------------------------------------------------------------
Reg1_ServRecv1                                          Registration 1: Service 1 Received
-----------------------------------------------------------------------------------------------

                  type:  numeric (int)
                 label:  Service

                 range:  [3,1000]                     units:  1
         unique values:  10                       missing .:  105/4168
       unique mv codes:  2                       missing .*:  205/4168

            tabulation:  Freq.   Numeric  Label
                             4         3  3. FP: INDIVIDUAL COUNSELING
                           998         4  4. FP: LONG TERM METHODS
                                          PROVIDED
                             4         5  5. FP: SHORT TERM METHODS
                                          PROVIDED
                            30         6  6. HIV: HTC - COUPLES
                          2317         7  7. HIV: HTC - INDIVIDUAL
                             2         8  8. HIV: ARV PROVISION
                             1         9  9. HIV: CD4 TESTING
                            19        14  14. VMMC: COUNSELING ONLY
                           474        15  15. VMMC: PROCEDURE
                             9      1000  1000. NO ADDITIONAL SERVICE
                           105         .
                           205        .j  .j. Ineligible: External site
                                          client

-----------------------------------------------------------------------------------------------
Reg1_DateServRecv1                                 Registration 1: Date Service 1 Received
-----------------------------------------------------------------------------------------------

                  type:  numeric daily date (int)

                 range:  [19709,20037]                units:  1
       or equivalently:  [17dec2013,10nov2014]        units:  days
         unique values:  241                      missing .:  105/4168
       unique mv codes:  2                       missing .*:  205/4168

                  mean:   19865.2 = 22may2014 (+ 5 hours)
             std. dev:    74.7905

           percentiles:        10%       25%       50%       75%       90%
                             19771     19806     19862     19921     19970
                         17feb2014 24mar2014 19may2014 17jul2014 04sep2014

-----------------------------------------------------------------------------------------------
Reg1_ServDate1_sys                          Registration 1: System Date Service 1 Received
-----------------------------------------------------------------------------------------------

                  type:  numeric (float)

                 range:  [1.703e+12,1.731e+12]        units:  100000
         unique values:  3734                     missing .:  1/4168
       unique mv codes:  2                       missing .*:  205/4168
```

```
                 mean:   1.7e+12
             std. dev:   6.5e+09

          percentiles:        10%       25%       50%       75%       90%
                          1.7e+12   1.7e+12   1.7e+12   1.7e+12   1.7e+12

-------------------------------------------------------------------------------------------
Reg1_ServCameFor2                                        Registration 1: Service 2 Came For
-------------------------------------------------------------------------------------------

                 type:  numeric (int)
                label:  Service

                range:  [11,1000]                    units:  1
        unique values:  3                        missing .:  0/4168
      unique mv codes:  1                       missing .*:  205/4168

           tabulation:  Freq.   Numeric  Label
                            1        11  11. HIV: TB TESTING
                            2        13  13. STI: TREATMENT
                         3960      1000  1000. NO ADDITIONAL SERVICE
                          205        .j  .j. Ineligible: External site
                                         client

-------------------------------------------------------------------------------------------
Reg1_ServRecv2                                           Registration 1: Service 2 Received
-------------------------------------------------------------------------------------------

                 type:  numeric (int)
                label:  Service

                range:  [5,1000]                     units:  1
        unique values:  6                        missing .:  106/4168
      unique mv codes:  2                       missing .*:  205/4168

           tabulation:  Freq.   Numeric  Label
                            1         5  5. FP: SHORT TERM METHODS
                                         PROVIDED
                            1        11  11. HIV: TB TESTING
                           17        12  12. STI: ASSESSMENT
                            2        13  13. STI: TREATMENT
                            1        14  14. VMMC: COUNSELING ONLY
                         3835      1000  1000. NO ADDITIONAL SERVICE
                          106         .
                          205        .j  .j. Ineligible: External site
                                         client

-------------------------------------------------------------------------------------------
Reg1_DateServRecv2                                  Registration 1: Date Service 2 Received
-------------------------------------------------------------------------------------------

                 type:  numeric daily date (int)

                range:  [19709,20037]                units:  1
      or equivalently:  [17dec2013,10nov2014]        units:  days
        unique values:  241                      missing .:  106/4168
      unique mv codes:  2                       missing .*:  205/4168

                 mean:   19865.2 = 22may2014 (+ 6 hours)
             std. dev:   74.7751

          percentiles:        10%       25%       50%       75%       90%
                            19771     19806     19862     19921     19970
                        17feb2014 24mar2014 19may2014 17jul2014 04sep2014

-------------------------------------------------------------------------------------------
Reg1_ServDate2_sys                           Registration 1: System Date Service 2 Received
-------------------------------------------------------------------------------------------

                 type:  numeric (float)
```

```
              range:  [1.703e+12,1.731e+12]        units:  100000
      unique values:  3736                     missing .:  1/4168
    unique mv codes:  2                       missing .*:  205/4168

               mean:   1.7e+12
           std. dev:   6.5e+09

        percentiles:        10%       25%       50%       75%       90%
                        1.7e+12   1.7e+12   1.7e+12   1.7e+12   1.7e+12

-------------------------------------------------------------------------------
Reg1_ServCameFor3                             Registration 1: Service 3 Came For
-------------------------------------------------------------------------------

               type:  numeric (int)
              label:  Service

              range:  [5,1000]                     units:  1
      unique values:  3                        missing .:  0/4168
    unique mv codes:  1                       missing .*:  205/4168

         tabulation:  Freq.   Numeric  Label
                          1         5  5. FP: SHORT TERM METHODS
                                       PROVIDED
                          2        14  14. VMMC: COUNSELING ONLY
                       3960      1000  1000. NO ADDITIONAL SERVICE
                        205        .j  .j. Ineligible: External site
                                       client

-------------------------------------------------------------------------------
Reg1_ServRecv3                                Registration 1: Service 3 Received
-------------------------------------------------------------------------------

               type:  numeric (int)
              label:  Service

              range:  [5,1000]                     units:  1
      unique values:  2                        missing .:  106/4168
    unique mv codes:  2                       missing .*:  205/4168

         tabulation:  Freq.   Numeric  Label
                          1         5  5. FP: SHORT TERM METHODS
                                       PROVIDED
                       3856      1000  1000. NO ADDITIONAL SERVICE
                        106         .
                        205        .j  .j. Ineligible: External site
                                       client

-------------------------------------------------------------------------------
Reg1_DateServRecv3                       Registration 1: Date Service 3 Received
-------------------------------------------------------------------------------

               type:  numeric daily date (int)

              range:  [19709,20037]                units:  1
    or equivalently:  [17dec2013,10nov2014]        units:  days
      unique values:  241                      missing .:  106/4168
    unique mv codes:  2                       missing .*:  205/4168

               mean:   19865.2 = 22may2014 (+ 6 hours)
           std. dev:   74.7751

        percentiles:        10%       25%       50%       75%       90%
                          19771     19806     19862     19921     19970
                      17feb2014 24mar2014 19may2014 17jul2014 04sep2014

-------------------------------------------------------------------------------
Reg1_ServDate3_sys                Registration 1: System Date Service 3 Received
-------------------------------------------------------------------------------
```

```
                 type:  numeric (float)

                range:  [1.703e+12,1.731e+12]        units:  100000
        unique values:  3739                     missing .:  1/4168
      unique mv codes:  2                       missing .*:  205/4168

                 mean:   1.7e+12
             std. dev:   6.5e+09

          percentiles:        10%       25%       50%       75%       90%
                          1.7e+12   1.7e+12   1.7e+12   1.7e+12   1.7e+12

--------------------------------------------------------------------------------------------
Reg1_ServCameFor4                                 Registration 1: Service 4 Came For
--------------------------------------------------------------------------------------------

                 type:  numeric (int)
                label:  Service

                range:  [13,1000]                    units:  1
        unique values:  3                        missing .:  0/4168
      unique mv codes:  1                       missing .*:  205/4168

           tabulation:  Freq.   Numeric  Label
                            1        13  13. STI: TREATMENT
                            3        14  14. VMMC: COUNSELING ONLY
                         3959      1000  1000. NO ADDITIONAL SERVICE
                          205        .j  .j. Ineligible: External site
                                         client

--------------------------------------------------------------------------------------------
Reg1_ServRecv4                                    Registration 1: Service 4 Received
--------------------------------------------------------------------------------------------

                 type:  numeric (int)
                label:  Service

                range:  [5,1000]                     units:  1
        unique values:  3                        missing .:  106/4168
      unique mv codes:  2                       missing .*:  205/4168

           tabulation:  Freq.   Numeric  Label
                            1         5  5. FP: SHORT TERM METHODS
                                         PROVIDED
                            3        14  14. VMMC: COUNSELING ONLY
                         3853      1000  1000. NO ADDITIONAL SERVICE
                          106         .
                          205        .j  .j. Ineligible: External site
                                         client

--------------------------------------------------------------------------------------------
Reg1_DateServRecv4                           Registration 1: Date Service 4 Received
--------------------------------------------------------------------------------------------

                 type:  numeric daily date (int)

                range:  [19709,20037]                units:  1
      or equivalently:  [17dec2013,10nov2014]        units:  days
        unique values:  241                      missing .:  106/4168
      unique mv codes:  2                       missing .*:  205/4168

                 mean:   19865.2 = 22may2014 (+ 6 hours)
             std. dev:   74.7751

          percentiles:        10%       25%       50%       75%       90%
                            19771     19806     19862     19921     19970
                        17feb2014 24mar2014 19may2014 17jul2014 04sep2014

--------------------------------------------------------------------------------------------
```

```
Reg1_ServDate4_sys                          Registration 1: System Date Service 4 Received
--------------------------------------------------------------------------------------------

                  type:  numeric (float)

                 range:  [1.703e+12,1.731e+12]        units:  100000
         unique values:  3739                     missing .:  1/4168
       unique mv codes:  2                       missing .*:  205/4168

                  mean:   1.7e+12
              std. dev:   6.5e+09

           percentiles:        10%       25%       50%       75%       90%
                            1.7e+12   1.7e+12   1.7e+12   1.7e+12   1.7e+12

--------------------------------------------------------------------------------------------
Reg1_ServCameFor5                                       Registration 1: Service 5 Came For
--------------------------------------------------------------------------------------------

                  type:  numeric (int)
                 label:  Service

                 range:  [5,1000]                     units:  1
         unique values:  3                        missing .:  1291/4168
       unique mv codes:  2                       missing .*:  205/4168

            tabulation:  Freq.   Numeric  Label
                             1         5  5. FP: SHORT TERM METHODS
                                          PROVIDED
                             1        13  13. STI: TREATMENT
                          2670      1000  1000. NO ADDITIONAL SERVICE
                          1291         .
                           205        .j  .j. Ineligible: External site
                                          client

--------------------------------------------------------------------------------------------
Reg1_ServRecv5                                          Registration 1: Service 5 Received
--------------------------------------------------------------------------------------------

                  type:  numeric (int)
                 label:  Service

                 range:  [5,1000]                     units:  1
         unique values:  3                        missing .:  1370/4168
       unique mv codes:  2                       missing .*:  205/4168

            tabulation:  Freq.   Numeric  Label
                             1         5  5. FP: SHORT TERM METHODS
                                          PROVIDED
                             2        13  13. STI: TREATMENT
                          2590      1000  1000. NO ADDITIONAL SERVICE
                          1370         .
                           205        .j  .j. Ineligible: External site
                                          client

--------------------------------------------------------------------------------------------
Reg1_DateServRecv5                                 Registration 1: Date Service 5 Received
--------------------------------------------------------------------------------------------

                  type:  numeric daily date (int)

                 range:  [19709,20037]                units:  1
       or equivalently:  [17dec2013,10nov2014]        units:  days
         unique values:  214                      missing .:  1369/4168
       unique mv codes:  2                       missing .*:  205/4168

                  mean:   19879.5 = 05jun2014 (+ 11 hours)
              std. dev:   75.3854

           percentiles:        10%       25%       50%       75%       90%
```

```
                       19775     19823     19878     19935     19981
                   21feb2014 10apr2014 04jun2014 31jul2014 15sep2014

-----------------------------------------------------------------------------------------------
Reg1_ServDate5_sys                              Registration 1: System Date Service 5 Received
-----------------------------------------------------------------------------------------------

                  type:  numeric (float)

                 range:  [1.703e+12,1.731e+12]        units:  100000
         unique values:  2571                     missing .:  1291/4168
       unique mv codes:  2                       missing .*:  205/4168

                  mean:   1.7e+12
             std. dev:   6.5e+09

           percentiles:        10%       25%       50%       75%       90%
                           1.7e+12   1.7e+12   1.7e+12   1.7e+12   1.7e+12

-----------------------------------------------------------------------------------------------
Reg2_ID                                                                      Registration 2 ID
-----------------------------------------------------------------------------------------------

                  type:  numeric (float)

                 range:  [597,67184]                  units:  1
         unique values:  16                       missing .:  3947/4168
       unique mv codes:  2                       missing .*:  205/4168

            tabulation:  Freq.  Value
                             1  597
                             1  47106
                             1  55214
                             1  55237
                             1  55274
                             1  55276
                             1  56358
                             1  56622
                             1  56775
                             1  58009
                             1  66336
                             1  66475
                             1  67015
                             1  67150
                             1  67181
                             1  67184
                          3947  .
                           205  .j

-----------------------------------------------------------------------------------------------
Reg2_PersID                                  Registration 2: Respondent or Person Accompanying
-----------------------------------------------------------------------------------------------

                  type:  numeric (float)
                 label:  RegPersID, but 16 nonmissing values are not labeled

                 range:  [1020,85004]                 units:  1
         unique values:  16                       missing .:  3947/4168
       unique mv codes:  2                       missing .*:  205/4168

            tabulation:  Freq.   Numeric  Label
                             1      1020
                             1     53008
                             1     61001
                             1     61002
                             1     61003
                             1     61004
                             1     62001
                             1     62002
                             1     62003
```

```
                      1     64001
                      1     73001
                      1     73002
                      1     85001
                      1     85002
                      1     85003
                      1     85004
                   3947         .
                    205        .j  .j. Ineligible: External site
                                   client

-------------------------------------------------------------------------------------------------
Reg2_Counseling                                         Registration 2: Counseling Received
-------------------------------------------------------------------------------------------------

                  type:  numeric (byte)
                 label:  Counseling

                 range:  [1,3]                        units:  1
         unique values:  3                        missing .:  3949/4168
       unique mv codes:  2                       missing .*:  205/4168

            tabulation:  Freq.   Numeric  Label
                             1         1  1. GROUP
                            12         2  2. ONE-ON-ONE
                             1         3  3. BOTH
                          3949         .
                           205        .j  .j. Ineligible: External site
                                          client

-------------------------------------------------------------------------------------------------
Reg2_Escort                                                 Registration 2: Escort Provided
-------------------------------------------------------------------------------------------------

                  type:  numeric (byte)
                 label:  Reg2_Escort

                 range:  [.,.]                        units:  .
         unique values:  0                        missing .:  0/4168
       unique mv codes:  4                       missing .*:  4168/4168

            tabulation:  Freq.   Numeric  Label
                          2642        .a  .a. Not in integrated arm
                             7        .b  .b. Integrated arm, no referrals
                          1314        .c  .c. Integrated arm, no one
                                          accompanied at registration
                           205        .j  .j. Ineligible: External site
                                          client

-------------------------------------------------------------------------------------------------
Reg2_EscortTime                                              Registration 2: Time of Escort
-------------------------------------------------------------------------------------------------

                  type:  string (str1), but longest is str0

         unique values:  0                        missing "":  4168/4168

            tabulation:  Freq.  Value
                          4168  ""

-------------------------------------------------------------------------------------------------
Reg2_ServCameFor1                                        Registration 2: Service 1 Came For
-------------------------------------------------------------------------------------------------

                  type:  numeric (int)
                 label:  Service

                 range:  [2,1000]                     units:  1
         unique values:  5                        missing .:  3947/4168
       unique mv codes:  2                       missing .*:  205/4168
```

```
          tabulation:  Freq.   Numeric  Label
                           2         2  2. FP: GROUP COUNSELING
                          10         6  6. HIV: HTC - COUPLES
                           1         7  7. HIV: HTC - INDIVIDUAL
                           1         8  8. HIV: ARV PROVISION
                           2      1000  1000. NO ADDITIONAL SERVICE
                        3947         .
                         205        .j  .j. Ineligible: External site
                                        client

-------------------------------------------------------------------------------
Reg2_ServRecv1                                  Registration 2: Service 1 Received
-------------------------------------------------------------------------------

                type:  numeric (byte)
               label:  Service

               range:  [2,8]                        units:  1
       unique values:  5                        missing .:  3949/4168
     unique mv codes:  2                       missing .*:  205/4168

          tabulation:  Freq.   Numeric  Label
                           1         2  2. FP: GROUP COUNSELING
                           1         3  3. FP: INDIVIDUAL COUNSELING
                          10         6  6. HIV: HTC - COUPLES
                           1         7  7. HIV: HTC - INDIVIDUAL
                           1         8  8. HIV: ARV PROVISION
                        3949         .
                         205        .j  .j. Ineligible: External site
                                        client

-------------------------------------------------------------------------------
Reg2_DateServRecv1                         Registration 2: Date Service 1 Received
-------------------------------------------------------------------------------

                type:  numeric daily date (int)

               range:  [19747,19989]                units:  1
   or equivalently:  [24jan2014,23sep2014]        units:  days
       unique values:  12                       missing .:  3949/4168
     unique mv codes:  2                       missing .*:  205/4168

          tabulation:  Freq.  Value
                           1  19747  24jan2014
                           1  19799  17mar2014
                           1  19808  26mar2014
                           1  19813  31mar2014
                           2  19823  10apr2014
                           2  19830  17apr2014
                           1  19837  24apr2014
                           1  19855  12may2014
                           1  19880  06jun2014
                           1  19892  18jun2014
                           1  19936  01aug2014
                           1  19989  23sep2014
                        3949  .              .
                         205  .j            .j

-------------------------------------------------------------------------------
Reg2_ServDate1_sys                  Registration 2: System Date Service 1 Received
-------------------------------------------------------------------------------

                type:  numeric (float)

               range:  [1.706e+12,1.727e+12]        units:  100000
       unique values:  16                       missing .:  3947/4168
     unique mv codes:  2                       missing .*:  205/4168

          tabulation:  Freq.  Value
```

```
                              1  1.706e+12
                              1  1.711e+12
                              1  1.711e+12
                              1  1.712e+12
                              1  1.712e+12
                              1  1.713e+12
                              1  1.713e+12
                              1  1.713e+12
                              1  1.713e+12
                              1  1.715e+12
                              1  1.716e+12
                              1  1.718e+12
                              1  1.719e+12
                              1  1.719e+12
                              1  1.723e+12
                              1  1.727e+12
                           3947  .
                            205  .j

-------------------------------------------------------------------------------------------------
Reg2_ServCameFor2                                         Registration 2: Service 2 Came For
-------------------------------------------------------------------------------------------------

                  type:  numeric (int)
                 label:  Service

                 range:  [1000,1000]                    units:  1
         unique values:  1                          missing .:  3947/4168
       unique mv codes:  2                         missing .*:  205/4168

           tabulation:  Freq.   Numeric  Label
                           16      1000  1000. NO ADDITIONAL SERVICE
                         3947         .
                          205        .j  .j. Ineligible: External site
                                         client

-------------------------------------------------------------------------------------------------
Reg2_ServRecv2                                            Registration 2: Service 2 Received
-------------------------------------------------------------------------------------------------

                  type:  numeric (int)
                 label:  Service

                 range:  [1000,1000]                    units:  1
         unique values:  1                          missing .:  3949/4168
       unique mv codes:  2                         missing .*:  205/4168

           tabulation:  Freq.   Numeric  Label
                           14      1000  1000. NO ADDITIONAL SERVICE
                         3949         .
                          205        .j  .j. Ineligible: External site
                                         client

-------------------------------------------------------------------------------------------------
Reg2_DateServRecv2                                   Registration 2: Date Service 2 Received
-------------------------------------------------------------------------------------------------

                  type:  numeric daily date (int)

                 range:  [19747,19989]                  units:  1
       or equivalently:  [24jan2014,23sep2014]          units:  days
         unique values:  12                         missing .:  3949/4168
       unique mv codes:  2                         missing .*:  205/4168

           tabulation:  Freq.  Value
                            1  19747  24jan2014
                            1  19799  17mar2014
                            1  19808  26mar2014
                            1  19813  31mar2014
                            2  19823  10apr2014
```

```
                          2  19830  17apr2014
                          1  19837  24apr2014
                          1  19855  12may2014
                          1  19880  06jun2014
                          1  19892  18jun2014
                          1  19936  01aug2014
                          1  19989  23sep2014
                       3949  .              .
                        205  .j            .j

-------------------------------------------------------------------------------
Reg2_ServDate2_sys                    Registration 2: System Date Service 2 Received
-------------------------------------------------------------------------------

                  type:  numeric (float)

                 range:  [1.706e+12,1.727e+12]        units:  100000
         unique values:  16                       missing .:  3947/4168
       unique mv codes:  2                       missing .*:  205/4168

            tabulation:  Freq.  Value
                             1  1.706e+12
                             1  1.711e+12
                             1  1.711e+12
                             1  1.712e+12
                             1  1.712e+12
                             1  1.713e+12
                             1  1.713e+12
                             1  1.713e+12
                             1  1.713e+12
                             1  1.715e+12
                             1  1.716e+12
                             1  1.718e+12
                             1  1.719e+12
                             1  1.719e+12
                             1  1.723e+12
                             1  1.727e+12
                          3947  .
                           205  .j

-------------------------------------------------------------------------------
Reg2_ServCameFor3                          Registration 2: Service 3 Came For
-------------------------------------------------------------------------------

                  type:  numeric (int)
                 label:  Service

                 range:  [1000,1000]                 units:  1
         unique values:  1                        missing .:  3947/4168
       unique mv codes:  2                       missing .*:  205/4168

            tabulation:  Freq.   Numeric  Label
                            16      1000  1000. NO ADDITIONAL SERVICE
                          3947         .
                           205        .j  .j. Ineligible: External site
                                          client

-------------------------------------------------------------------------------
Reg2_ServRecv3                             Registration 2: Service 3 Received
-------------------------------------------------------------------------------

                  type:  numeric (int)
                 label:  Service

                 range:  [1000,1000]                 units:  1
         unique values:  1                        missing .:  3949/4168
       unique mv codes:  2                       missing .*:  205/4168

            tabulation:  Freq.   Numeric  Label
                            14      1000  1000. NO ADDITIONAL SERVICE
```

```
                        3949         .
                         205        .j  .j. Ineligible: External site
                                        client

-------------------------------------------------------------------------------------------
Reg2_DateServRecv3                                Registration 2: Date Service 3 Received
-------------------------------------------------------------------------------------------

                  type:  numeric daily date (int)

                 range:  [19747,19989]                units:  1
       or equivalently:  [24jan2014,23sep2014]        units:  days
         unique values:  12                       missing .:  3949/4168
       unique mv codes:  2                       missing .*:  205/4168

            tabulation:  Freq.  Value
                             1  19747  24jan2014
                             1  19799  17mar2014
                             1  19808  26mar2014
                             1  19813  31mar2014
                             2  19823  10apr2014
                             2  19830  17apr2014
                             1  19837  24apr2014
                             1  19855  12may2014
                             1  19880  06jun2014
                             1  19892  18jun2014
                             1  19936  01aug2014
                             1  19989  23sep2014
                          3949  .              .
                           205  .j            .j

-------------------------------------------------------------------------------------------
Reg2_ServDate3_sys                         Registration 2: System Date Service 3 Received
-------------------------------------------------------------------------------------------

                  type:  numeric (float)

                 range:  [1.706e+12,1.727e+12]        units:  100000
         unique values:  16                       missing .:  3947/4168
       unique mv codes:  2                       missing .*:  205/4168

            tabulation:  Freq.  Value
                             1  1.706e+12
                             1  1.711e+12
                             1  1.711e+12
                             1  1.712e+12
                             1  1.712e+12
                             1  1.713e+12
                             1  1.713e+12
                             1  1.713e+12
                             1  1.713e+12
                             1  1.715e+12
                             1  1.716e+12
                             1  1.718e+12
                             1  1.719e+12
                             1  1.719e+12
                             1  1.723e+12
                             1  1.727e+12
                          3947  .
                           205  .j

-------------------------------------------------------------------------------------------
Reg2_ServCameFor4                                    Registration 2: Service 4 Came For
-------------------------------------------------------------------------------------------

                  type:  numeric (int)
                 label:  Service

                 range:  [1000,1000]                  units:  1
         unique values:  1                        missing .:  3947/4168
```

```
      unique mv codes:  2                       missing .*:  205/4168

           tabulation:  Freq.   Numeric  Label
                           16      1000  1000. NO ADDITIONAL SERVICE
                         3947         .
                          205        .j  .j. Ineligible: External site
                                         client

-------------------------------------------------------------------------------
Reg2_ServRecv4                                 Registration 2: Service 4 Received
-------------------------------------------------------------------------------

                 type:  numeric (int)
                label:  Service

                range:  [1000,1000]                  units:  1
        unique values:  1                        missing .:  3949/4168
      unique mv codes:  2                       missing .*:  205/4168

           tabulation:  Freq.   Numeric  Label
                           14      1000  1000. NO ADDITIONAL SERVICE
                         3949         .
                          205        .j  .j. Ineligible: External site
                                         client

-------------------------------------------------------------------------------
Reg2_DateServRecv4                        Registration 2: Date Service 4 Received
-------------------------------------------------------------------------------

                 type:  numeric daily date (int)

                range:  [19747,19989]                units:  1
      or equivalently:  [24jan2014,23sep2014]        units:  days
        unique values:  12                       missing .:  3949/4168
      unique mv codes:  2                       missing .*:  205/4168

           tabulation:  Freq.  Value
                            1  19747  24jan2014
                            1  19799  17mar2014
                            1  19808  26mar2014
                            1  19813  31mar2014
                            2  19823  10apr2014
                            2  19830  17apr2014
                            1  19837  24apr2014
                            1  19855  12may2014
                            1  19880  06jun2014
                            1  19892  18jun2014
                            1  19936  01aug2014
                            1  19989  23sep2014
                         3949  .              .
                          205  .j            .j

-------------------------------------------------------------------------------
Reg2_ServDate4_sys                   Registration 2: System Date Service 4 Received
-------------------------------------------------------------------------------

                 type:  numeric (float)

                range:  [1.706e+12,1.727e+12]        units:  100000
        unique values:  16                       missing .:  3947/4168
      unique mv codes:  2                       missing .*:  205/4168

           tabulation:  Freq.  Value
                            1  1.706e+12
                            1  1.711e+12
                            1  1.711e+12
                            1  1.712e+12
                            1  1.712e+12
                            1  1.713e+12
                            1  1.713e+12
```

```
                         1  1.713e+12
                         1  1.713e+12
                         1  1.715e+12
                         1  1.716e+12
                         1  1.718e+12
                         1  1.719e+12
                         1  1.719e+12
                         1  1.723e+12
                         1  1.727e+12
                      3947  .
                       205  .j

-------------------------------------------------------------------------------------------------
Reg2_ServCameFor5                                        Registration 2: Service 5 Came For
-------------------------------------------------------------------------------------------------

                 type:  numeric (int)
                label:  Service

                range:  [1000,1000]                  units:  1
        unique values:  1                        missing .:  3957/4168
      unique mv codes:  2                       missing .*:  205/4168

           tabulation:  Freq.   Numeric  Label
                            6      1000  1000. NO ADDITIONAL SERVICE
                         3957         .
                          205        .j  .j. Ineligible: External site
                                         client

-------------------------------------------------------------------------------------------------
Reg2_ServRecv5                                           Registration 2: Service 5 Received
-------------------------------------------------------------------------------------------------

                 type:  numeric (int)
                label:  Service

                range:  [1000,1000]                  units:  1
        unique values:  1                        missing .:  3958/4168
      unique mv codes:  2                       missing .*:  205/4168

           tabulation:  Freq.   Numeric  Label
                            5      1000  1000. NO ADDITIONAL SERVICE
                         3958         .
                          205        .j  .j. Ineligible: External site
                                         client

-------------------------------------------------------------------------------------------------
Reg2_DateServRecv5                                  Registration 2: Date Service 5 Received
-------------------------------------------------------------------------------------------------

                 type:  numeric daily date (int)

                range:  [19747,19892]                units:  1
      or equivalently:  [24jan2014,18jun2014]        units:  days
        unique values:  5                        missing .:  3958/4168
      unique mv codes:  2                       missing .*:  205/4168

           tabulation:  Freq.  Value
                            1  19747  24jan2014
                            1  19830  17apr2014
                            1  19855  12may2014
                            1  19880  06jun2014
                            1  19892  18jun2014
                         3958  .              .
                          205  .j            .j

-------------------------------------------------------------------------------------------------
Reg2_ServDate5_sys                           Registration 2: System Date Service 5 Received
-------------------------------------------------------------------------------------------------
```

```
                 type:  numeric (float)

                range:  [1.706e+12,1.719e+12]        units:  100000
        unique values:  6                        missing .:  3957/4168
      unique mv codes:  2                       missing .*:  205/4168

           tabulation:  Freq.  Value
                            1  1.706e+12
                            1  1.713e+12
                            1  1.716e+12
                            1  1.718e+12
                            1  1.719e+12
                            1  1.719e+12
                         3957  .
                          205  .j

-----------------------------------------------------------------------------------------------
Ref1_ID                                                                           Referral 1 ID
-----------------------------------------------------------------------------------------------

                 type:  numeric (long)

                range:  [267,86021]                  units:  1
        unique values:  1958                     missing .:  2005/4168
      unique mv codes:  2                       missing .*:  205/4168

                 mean:   55888.1
             std. dev:   16105.7

          percentiles:        10%       25%       50%       75%       90%
                            44356     47537     56184     67247     71531

Ref1_ID:
  1.  if begins with 99, referral created for analysis

-----------------------------------------------------------------------------------------------
Ref1_Source_an                                                                Referral 1 Source
-----------------------------------------------------------------------------------------------

                 type:  numeric (byte)
                label:  RefSource

                range:  [1,3]                        units:  1
        unique values:  2                        missing .:  2005/4168
      unique mv codes:  2                       missing .*:  205/4168

           tabulation:  Freq.   Numeric  Label
                         1956         1  1. Registration
                            2         3  3. Self
                         2005         .
                          205        .j  .j. Ineligible: External site
                                         client

Ref1_Source_an:
  1.  created for analysis

-----------------------------------------------------------------------------------------------
Ref1_ParentID                                                 Referral 1 Parent ID: RegID or RefID
-----------------------------------------------------------------------------------------------

                 type:  numeric (long)

                range:  [265,86020]                  units:  1
        unique values:  1958                     missing .:  2005/4168
      unique mv codes:  2                       missing .*:  205/4168

                 mean:   55742.8
             std. dev:     16051

          percentiles:        10%       25%       50%       75%       90%
```

```
                        44345     47533     56134     67207     71525

-----------------------------------------------------------------------------------------------
Ref1_Self_an                                                          Referral 1 for Respondent
-----------------------------------------------------------------------------------------------

                  type:  numeric (byte)
                 label:  yesno

                 range:  [0,1]                        units:  1
         unique values:  2                        missing .:  2007/4168
       unique mv codes:  2                       missing .*:  205/4168

            tabulation:  Freq.   Numeric  Label
                           529         0  0. No
                          1427         1  1. Yes
                          2007         .  
                           205        .j  .j. Ineligible: External site
                                          client

Ref1_Self_an:
  1.  created for analysis

-----------------------------------------------------------------------------------------------
Ref1_Partner_an                                              Referral 1 for Respondent's Partner
-----------------------------------------------------------------------------------------------

                  type:  numeric (byte)
                 label:  yesno

                 range:  [0,1]                        units:  1
         unique values:  2                        missing .:  2007/4168
       unique mv codes:  2                       missing .*:  205/4168

            tabulation:  Freq.   Numeric  Label
                          1504         0  0. No
                           452         1  1. Yes
                          2007         .  
                           205        .j  .j. Ineligible: External site
                                          client

Ref1_Partner_an:
  1.  created for analysis

-----------------------------------------------------------------------------------------------
Ref1_Other_an                                                              Referral 1 for Other
-----------------------------------------------------------------------------------------------

                  type:  numeric (byte)
                 label:  yesno

                 range:  [0,1]                        units:  1
         unique values:  2                        missing .:  2007/4168
       unique mv codes:  2                       missing .*:  205/4168

            tabulation:  Freq.   Numeric  Label
                          1879         0  0. No
                            77         1  1. Yes
                          2007         .  
                           205        .j  .j. Ineligible: External site
                                          client

Ref1_Other_an:
  1.  created for analysis

-----------------------------------------------------------------------------------------------
Ref1_PersID                                 Referral 1 Given To: Respondent or Person Accompanying
-----------------------------------------------------------------------------------------------

                  type:  numeric (byte)
```

```
                label:  RefPersID

                range:  [0,0]                        units:  1
        unique values:  1                        missing .:  2005/4168
      unique mv codes:  2                       missing .*:  205/4168

           tabulation:  Freq.   Numeric  Label
                         1958         0  0. Respondent
                         2005         .  
                          205        .j  .j. Ineligible: External site
                                         client

-----------------------------------------------------------------------------------------------
Ref1_Date                                                               Referral 1: Date Given
-----------------------------------------------------------------------------------------------

                 type:  numeric daily date (int)

                range:  [19710,20037]                units:  1
     or equivalently:  [18dec2013,10nov2014]        units:  days
        unique values:  222                      missing .:  2005/4168
      unique mv codes:  2                       missing .*:  205/4168

                 mean:   19861.2 = 18may2014 (+ 4 hours)
             std. dev:    72.675

          percentiles:        10%       25%       50%       75%       90%
                            19771     19803     19857     19914     19964
                        17feb2014 21mar2014 14may2014 10jul2014 29aug2014

-----------------------------------------------------------------------------------------------
Ref1_ReferralFOR                                Referral 1 For: Relationship to RefPersID_Ref1
-----------------------------------------------------------------------------------------------

                 type:  numeric (byte)
                label:  relation

                range:  [0,12]                       units:  1
        unique values:  9                        missing .:  2005/4168
      unique mv codes:  2                       missing .*:  205/4168

           tabulation:  Freq.   Numeric  Label
                         1429         0  0. SELF
                          440         1  1. SPOUSE
                           12         2  2. COHABITATING PARTNER
                            1         3  3. DAUGHTER
                            6         4  4. SON
                            2         7  7. SISTER
                           26         8  8. BROTHER
                            1        11  11. OTHER RELATIVE
                           41        12  12. OTHER NON-RELATIVE
                         2005         .  
                          205        .j  .j. Ineligible: External site
                                         client

-----------------------------------------------------------------------------------------------
Ref1_FromSite                                                         Referral 1 from Site
-----------------------------------------------------------------------------------------------

                 type:  numeric (byte)
                label:  site

                range:  [1,13]                       units:  1
        unique values:  10                       missing .:  2005/4168
      unique mv codes:  2                       missing .*:  205/4168

           tabulation:  Freq.   Numeric  Label
                          261         1  1. SFH HCT - Cairo Road
                          301         2  2. Chawama Clinic - MCH
                           10         3  3. Chawama Clinic - Out Patient
```

```
                                    Ward (VMMC)
                       151         5  5. Kamwala Clinic - TB, STI &
                                      HIV Clinic
                       332         6  6. Kapata Urban Clinic, MCH
                       400         7  7. Kapata Urban Clinic, TB, STI
                                      & HIV
                        59         8  8. Chipata Gen Hosp - OP VMMC
                       426        11  11. SFH New Start
                         1        12  12. SFH - VMMC - CHACHACHA RD
                        17        13  13. SFH VMMC
                      2005         .
                       205        .j  .j. Ineligible: External site
                                      client

-----------------------------------------------------------------------------------------------
Ref1_ToSite                                                                 Referral 1 to Site
-----------------------------------------------------------------------------------------------

                  type:  numeric (byte)
                 label:  site

                 range:  [1,13]                       units:  1
         unique values:  13                       missing .:  2005/4168
       unique mv codes:  2                       missing .*:  205/4168

            tabulation:  Freq.   Numeric  Label
                           101         1  1. SFH HCT - Cairo Road
                           389         2  2. Chawama Clinic - MCH
                            28         3  3. Chawama Clinic - Out Patient
                                          Ward (VMMC)
                             7         4  4. Chawama Clinic - TB, STI &
                                          HIV Clinic
                           116         5  5. Kamwala Clinic - TB, STI &
                                          HIV Clinic
                            17         6  6. Kapata Urban Clinic, MCH
                            68         7  7. Kapata Urban Clinic, TB, STI
                                          & HIV
                           354         8  8. Chipata Gen Hosp - OP VMMC
                           455         9  9. Chipata Gen Hosp - MCH
                           106        10  10. Chipata Gen Hosp - TB, STI &
                                          HIV
                           156        11  11. SFH New Start
                            83        12  12. SFH - VMMC - CHACHACHA RD
                            78        13  13. SFH VMMC
                          2005         .
                           205        .j  .j. Ineligible: External site
                                          client

-----------------------------------------------------------------------------------------------
Ref1_ServiceFor                                                         Referral 1 for Service
-----------------------------------------------------------------------------------------------

                  type:  numeric (int)
                 label:  Service

                 range:  [1,1002]                     units:  1
         unique values:  14                       missing .:  2005/4168
       unique mv codes:  2                       missing .*:  205/4168

            tabulation:  Freq.   Numeric  Label
                           839         1  1. CCS: CERVICAL CANCER
                                          SCREENING
                            11         3  3. FP: INDIVIDUAL COUNSELING
                            25         4  4. FP: LONG TERM METHODS
                                          PROVIDED
                             7         5  5. FP: SHORT TERM METHODS
                                          PROVIDED
                           133         6  6. HIV: HTC - COUPLES
                           154         7  7. HIV: HTC - INDIVIDUAL
                             1         8  8. HIV: ARV PROVISION
```

```
                       172         9  9. HIV: CD4 TESTING
                        11        11  11. HIV: TB TESTING
                        63        12  12. STI: ASSESSMENT
                        10        13  13. STI: TREATMENT
                         6        14  14. VMMC: COUNSELING ONLY
                       524        15  15. VMMC: PROCEDURE
                         2      1002  1002. SELF-REFERRAL
                      2005         .
                       205        .j  .j. Ineligible: External site
                                      client
```

Ref1_ServiceFor:
  1. Those who received referrals for couples HTC were coded in a specific manner based on the services they received prior. - If a respondent had received individual HTC, VMMC counseling or VMMC procedure prior to receiving the referral for couples HTC, s/he received one referral for couples HTC for his/her spouse or partner. - If a respondent did not receive individual HTC, VMMC counseling or VMMC procedure prior to receiving his/her referral for couples HTC, s/he received two referrals for couples HTC: one for self and one for spouse or partner.

```
-----------------------------------------------------------------------------------------------
Ref1_ShowDate                                         Referral 1: Date Return to Act On Referral
-----------------------------------------------------------------------------------------------

                  type:  numeric daily date (int)

                 range:  [19710,20034]                units:  1
       or equivalently:  [18dec2013,07nov2014]        units:  days
         unique values:  126                      missing .:  3710/4168
       unique mv codes:  2                       missing .*:  205/4168

                  mean:   19851.4 = 08may2014 (+ 10 hours)
              std. dev:   70.6133

           percentiles:        10%       25%       50%       75%       90%
                             19764     19800     19829     19904     19949
                         10feb2014 18mar2014 16apr2014 30jun2014 14aug2014

-----------------------------------------------------------------------------------------------
Ref1_ShowHMM                                          Referral 1: Time Return to Act On Referral
-----------------------------------------------------------------------------------------------

                  type:  string (str5)

         unique values:  159                      missing "":  3916/4168

              examples:  ""
                         ""
                         ""
                         ""

-----------------------------------------------------------------------------------------------
Ref1_ServDate                                                 Referral 1: Date Services Received
-----------------------------------------------------------------------------------------------

                  type:  numeric daily date (int)

                 range:  [19710,20025]                units:  1
       or equivalently:  [18dec2013,29oct2014]        units:  days
         unique values:  107                      missing .:  3769/4168
       unique mv codes:  2                       missing .*:  205/4168

                  mean:   19844.1 = 01may2014 (+ 3 hours)
              std. dev:   69.8849

           percentiles:        10%       25%       50%       75%       90%
                             19760     19795     19823     19900     19949
                         06feb2014 13mar2014 10apr2014 26jun2014 14aug2014

-----------------------------------------------------------------------------------------------
```

```
Ref1_ServHMM                                       Referral 1: Time Services Received
-------------------------------------------------------------------------------------

                  type:  string (str5)

         unique values:  142                      missing "":  3994/4168

              examples:  ""
                         ""
                         ""
                         ""

Ref1_ServHMM:
  1.  some missing due to correcting date service received, but not knowing time service
      received

-------------------------------------------------------------------------------------
Ref1_Counseling                                      Referral 1: Counseling Received
-------------------------------------------------------------------------------------

                  type:  numeric (byte)
                 label:  Counseling

                 range:  [1,4]                        units:  1
         unique values:  3                        missing .:  3789/4168
       unique mv codes:  2                       missing .*:  205/4168

            tabulation:  Freq.   Numeric  Label
                            24         1  1. GROUP
                           131         2  2. ONE-ON-ONE
                            19         4  4. NONE
                          3789         .
                           205        .j  .j. Ineligible: External site
                                          client

-------------------------------------------------------------------------------------
Ref1_ReceivedDayReturned                   Referral 1: Services Received Same Day Returned
-------------------------------------------------------------------------------------

                  type:  numeric (byte)
                 label:  yesno

                 range:  [1,1]                        units:  1
         unique values:  1                        missing .:  3789/4168
       unique mv codes:  2                       missing .*:  205/4168

            tabulation:  Freq.   Numeric  Label
                           174         1  1. Yes
                          3789         .
                           205        .j  .j. Ineligible: External site
                                          client

Ref1_ReceivedDayReturned:
  1.  Yes if Date showed up for services = Date services received
      (Ref1_ShowDate=Ref1_ServDate)

-------------------------------------------------------------------------------------
Ref1_Escort                                              Referral 1: Escort Provided
-------------------------------------------------------------------------------------

                  type:  numeric (byte)
                 label:  Ref1_Escort

                 range:  [0,1]                        units:  1
         unique values:  2                        missing .:  0/4168
       unique mv codes:  6                       missing .*:  4052/4168

            tabulation:  Freq.   Numeric  Label
                            16         0  0. No
                           100         1  1. Yes
```

```
                        2642        .a  .a. Not in integrated arm
                         337        .b  .b. No referral
                          11        .c  .c. Received services same day
                                        as registration: escort recorded
                                        in Reg1_Escort
                         839        .d  .d. Have not yet receieved
                                        services from referral
                         205        .j  .j. Ineligible: External site
                                        client
                          18        .m  .m. Missing

Ref1_Escort:
  1.  Those with value of .c received services the same day as registration.  Check
      Reg1_Escort to know whether they received an escort or not (many did).  Their escort
      data is recorded only in Reg1_Escort to enable an accurate count of escorts.  (If
      Reg1_Escort == 1, that is the escort who brought the client to get services from
      registration site to referral 1 site.)

-----------------------------------------------------------------------------------------------
Ref1_EscortTime                                               Referral 1: Time Escort Provided
-----------------------------------------------------------------------------------------------

                  type:  string (str5)

         unique values:  79                      missing "":  4068/4168

              examples:  ""
                         ""
                         ""
                         ""

-----------------------------------------------------------------------------------------------
Ref1_VoucherNumber                                                   Referral 1 Voucher Number
-----------------------------------------------------------------------------------------------

                  type:  string (str10)

         unique values:  1955                    missing "":  2213/4168

              examples:  ""
                         ""
                         "02-02-0079"
                         "07-08-7604"

Ref1_VoucherNumber:
  1.  if begins with 99, referral created for analysis

-----------------------------------------------------------------------------------------------
Ref1_ServCameFor1                                               Referral 1: Service 1 Came For
-----------------------------------------------------------------------------------------------

                  type:  numeric (byte)
                 label:  Service

                 range:  [1,15]                      units:  1
         unique values:  11                      missing .:  3710/4168
       unique mv codes:  2                      missing .*:  205/4168

            tabulation:  Freq.   Numeric  Label
                           153         1  1. CCS: CERVICAL CANCER
                                          SCREENING
                             2         4  4. FP: LONG TERM METHODS
                                          PROVIDED
                             8         6  6. HIV: HTC - COUPLES
                            20         7  7. HIV: HTC - INDIVIDUAL
                             1         8  8. HIV: ARV PROVISION
                            21         9  9. HIV: CD4 TESTING
                             4        11  11. HIV: TB TESTING
                             8        12  12. STI: ASSESSMENT
                             3        13  13. STI: TREATMENT
```

```
                         1        14  14. VMMC: COUNSELING ONLY
                        32        15  15. VMMC: PROCEDURE
                      3710         .
                       205        .j  .j. Ineligible: External site
                                      client

-------------------------------------------------------------------------------
Ref1_ServRecv1                                  Referral 1: Service 1 Received
-------------------------------------------------------------------------------

                  type:  numeric (int)
                 label:  Service

                 range:  [1,1000]                     units:  1
         unique values:  13                       missing .:  3769/4168
       unique mv codes:  2                       missing .*:  205/4168

            tabulation:  Freq.   Numeric  Label
                            99         1  1. CCS: CERVICAL CANCER
                                          SCREENING
                             1         3  3. FP: INDIVIDUAL COUNSELING
                             4         4  4. FP: LONG TERM METHODS
                                          PROVIDED
                             1         5  5. FP: SHORT TERM METHODS
                                          PROVIDED
                             3         6  6. HIV: HTC - COUPLES
                            23         7  7. HIV: HTC - INDIVIDUAL
                            15         9  9. HIV: CD4 TESTING
                             2        11  11. HIV: TB TESTING
                             6        12  12. STI: ASSESSMENT
                             1        13  13. STI: TREATMENT
                             2        14  14. VMMC: COUNSELING ONLY
                            28        15  15. VMMC: PROCEDURE
                             9      1000  1000. NO ADDITIONAL SERVICE
                          3769         .
                           205        .j  .j. Ineligible: External site
                                          client

-------------------------------------------------------------------------------
Ref1_DateServRec1                          Referral 1: Date Service 1 Received
-------------------------------------------------------------------------------

                  type:  numeric daily date (int)

                 range:  [19710,20025]                units:  1
     or equivalently:  [18dec2013,29oct2014]        units:  days
         unique values:  107                      missing .:  3769/4168
       unique mv codes:  2                       missing .*:  205/4168

                  mean:   19844.1 = 01may2014 (+ 3 hours)
              std. dev:   69.8849

           percentiles:        10%       25%       50%       75%       90%
                             19760     19795     19823     19900     19949
                         06feb2014 13mar2014 10apr2014 26jun2014 14aug2014

-------------------------------------------------------------------------------
Ref1_ServDate1_sys                  Referral 1: System Date Service 1 Received
-------------------------------------------------------------------------------

                  type:  numeric (double)

                 range:  [1.704e+12,1.730e+12]        units:  1000
         unique values:  174                      missing .:  3789/4168
       unique mv codes:  2                       missing .*:  205/4168

                  mean:   1.7e+12
              std. dev:   6.0e+09

           percentiles:        10%       25%       50%       75%       90%
```

```
                        1.7e+12   1.7e+12   1.7e+12   1.7e+12   1.7e+12

Ref1_ServDate1_sys:
  1.  some missing due to correcting date service received, but not knowing time service
      received

-----------------------------------------------------------------------------------------------
Ref1_ServCameFor2                                           Referral 1: Service 2 Came For
-----------------------------------------------------------------------------------------------

                  type:  numeric (int)
                 label:  Service

                 range:  [5,1000]                     units:  1
         unique values:  2                        missing .:  3710/4168
       unique mv codes:  2                       missing .*:  205/4168

            tabulation:  Freq.   Numeric  Label
                             1         5  5. FP: SHORT TERM METHODS
                                          PROVIDED
                           252      1000  1000. NO ADDITIONAL SERVICE
                          3710         .
                           205        .j  .j. Ineligible: External site
                                          client

-----------------------------------------------------------------------------------------------
Ref1_ServRecv2                                              Referral 1: Service 2 Received
-----------------------------------------------------------------------------------------------

                  type:  numeric (int)
                 label:  Service

                 range:  [1000,1000]                  units:  1
         unique values:  1                        missing .:  3769/4168
       unique mv codes:  2                       missing .*:  205/4168

            tabulation:  Freq.   Numeric  Label
                           194      1000  1000. NO ADDITIONAL SERVICE
                          3769         .
                           205        .j  .j. Ineligible: External site
                                          client

-----------------------------------------------------------------------------------------------
Ref1_DateServRec2                                      Referral 1: Date Service 2 Received
-----------------------------------------------------------------------------------------------

                  type:  numeric daily date (int)

                 range:  [19710,20025]                units:  1
       or equivalently:  [18dec2013,29oct2014]        units:  days
         unique values:  107                      missing .:  3769/4168
       unique mv codes:  2                       missing .*:  205/4168

                  mean:   19844.1 = 01may2014 (+ 3 hours)
              std. dev:   69.8849

           percentiles:        10%       25%       50%       75%       90%
                             19760     19795     19823     19900     19949
                         06feb2014 13mar2014 10apr2014 26jun2014 14aug2014

-----------------------------------------------------------------------------------------------
Ref1_ServDate2_sys                                Referral 1: System Date Service 2 Received
-----------------------------------------------------------------------------------------------

                  type:  numeric (double)

                 range:  [1.704e+12,1.730e+12]        units:  1000
         unique values:  174                      missing .:  3789/4168
       unique mv codes:  2                       missing .*:  205/4168
```

```
                 mean:   1.7e+12
            std. dev:   6.0e+09

         percentiles:        10%       25%       50%       75%       90%
                          1.7e+12   1.7e+12   1.7e+12   1.7e+12   1.7e+12

Ref1_ServDate2_sys:
  1.  some missing due to correcting date service received, but not knowing time service
      received

-----------------------------------------------------------------------------------------------
Ref1_ServCameFor3                                            Referral 1: Service 3 Came For
-----------------------------------------------------------------------------------------------

                 type:  numeric (int)
                label:  Service

                range:  [13,1000]                    units:  1
        unique values:  2                        missing .:  3710/4168
      unique mv codes:  2                       missing .*:  205/4168

          tabulation:  Freq.   Numeric  Label
                           1        13  13. STI: TREATMENT
                         252      1000  1000. NO ADDITIONAL SERVICE
                        3710         .
                         205        .j  .j. Ineligible: External site
                                        client

-----------------------------------------------------------------------------------------------
Ref1_ServRecv3                                               Referral 1: Service 3 Received
-----------------------------------------------------------------------------------------------

                 type:  numeric (int)
                label:  Service

                range:  [1000,1000]                  units:  1
        unique values:  1                        missing .:  3769/4168
      unique mv codes:  2                       missing .*:  205/4168

          tabulation:  Freq.   Numeric  Label
                         194      1000  1000. NO ADDITIONAL SERVICE
                        3769         .
                         205        .j  .j. Ineligible: External site
                                        client

-----------------------------------------------------------------------------------------------
Ref1_DateServRec3                                       Referral 1: Date Service 3 Received
-----------------------------------------------------------------------------------------------

                 type:  numeric daily date (int)

                range:  [19710,20025]                units:  1
      or equivalently:  [18dec2013,29oct2014]        units:  days
        unique values:  107                      missing .:  3769/4168
      unique mv codes:  2                       missing .*:  205/4168

                 mean:   19844.1 = 01may2014 (+ 3 hours)
            std. dev:   69.8849

         percentiles:        10%       25%       50%       75%       90%
                           19760     19795     19823     19900     19949
                        06feb2014 13mar2014 10apr2014 26jun2014 14aug2014

-----------------------------------------------------------------------------------------------
Ref1_ServDate3_sys                                Referral 1: System Date Service 3 Received
-----------------------------------------------------------------------------------------------

                 type:  numeric (double)

                range:  [1.704e+12,1.730e+12]        units:  1000
```

```
        unique values:  174                      missing .:  3789/4168
      unique mv codes:  2                       missing .*:  205/4168

                 mean:   1.7e+12
             std. dev:   6.0e+09

          percentiles:        10%       25%       50%       75%       90%
                          1.7e+12   1.7e+12   1.7e+12   1.7e+12   1.7e+12

Ref1_ServDate3_sys:
  1.  some missing due to correcting date service received, but not knowing time service
      received

-----------------------------------------------------------------------------------------------
Ref1_ServCameFor4                                          Referral 1: Service 4 Came For
-----------------------------------------------------------------------------------------------

                 type:  numeric (int)
                label:  Service

                range:  [5,1000]                     units:  1
        unique values:  2                        missing .:  3710/4168
      unique mv codes:  2                       missing .*:  205/4168

           tabulation:  Freq.   Numeric  Label
                            1         5  5. FP: SHORT TERM METHODS
                                         PROVIDED
                          252      1000  1000. NO ADDITIONAL SERVICE
                         3710         .
                          205        .j  .j. Ineligible: External site
                                         client

-----------------------------------------------------------------------------------------------
Ref1_ServRecv4                                             Referral 1: Service 4 Received
-----------------------------------------------------------------------------------------------

                 type:  numeric (int)
                label:  Service

                range:  [1000,1000]                  units:  1
        unique values:  1                        missing .:  3769/4168
      unique mv codes:  2                       missing .*:  205/4168

           tabulation:  Freq.   Numeric  Label
                          194      1000  1000. NO ADDITIONAL SERVICE
                         3769         .
                          205        .j  .j. Ineligible: External site
                                         client

-----------------------------------------------------------------------------------------------
Ref1_DateServRec4                                     Referral 1: Date Service 4 Received
-----------------------------------------------------------------------------------------------

                 type:  numeric daily date (int)

                range:  [19710,20025]                units:  1
      or equivalently:  [18dec2013,29oct2014]        units:  days
        unique values:  107                      missing .:  3769/4168
      unique mv codes:  2                       missing .*:  205/4168

                 mean:   19844.1 = 01may2014 (+ 3 hours)
             std. dev:   69.8849

          percentiles:        10%       25%       50%       75%       90%
                            19760     19795     19823     19900     19949
                        06feb2014 13mar2014 10apr2014 26jun2014 14aug2014

-----------------------------------------------------------------------------------------------
Ref1_ServDate4_sys                              Referral 1: System Date Service 4 Received
-----------------------------------------------------------------------------------------------
```

```
                  type:  numeric (double)

                 range:  [1.704e+12,1.730e+12]        units:  1000
         unique values:  174                      missing .:  3789/4168
       unique mv codes:  2                       missing .*:  205/4168

                  mean:   1.7e+12
              std. dev:   6.0e+09

           percentiles:        10%       25%       50%       75%       90%
                            1.7e+12   1.7e+12   1.7e+12   1.7e+12   1.7e+12

Ref1_ServDate4_sys:
  1.  some missing due to correcting date service received, but not knowing time service
      received

-----------------------------------------------------------------------------------------------
Ref1_ServCameFor5                                           Referral 1: Service 5 Came For
-----------------------------------------------------------------------------------------------

                  type:  numeric (int)
                 label:  Service

                 range:  [1000,1000]                  units:  1
         unique values:  1                        missing .:  3736/4168
       unique mv codes:  2                       missing .*:  205/4168

            tabulation:  Freq.   Numeric  Label
                           227      1000  1000. NO ADDITIONAL SERVICE
                          3736         .
                           205        .j  .j. Ineligible: External site
                                          client

-----------------------------------------------------------------------------------------------
Ref1_ServRecv5                                              Referral 1: Service 5 Received
-----------------------------------------------------------------------------------------------

                  type:  numeric (int)
                 label:  Service

                 range:  [5,1000]                     units:  1
         unique values:  2                        missing .:  3793/4168
       unique mv codes:  2                       missing .*:  205/4168

            tabulation:  Freq.   Numeric  Label
                             1         5  5. FP: SHORT TERM METHODS
                                          PROVIDED
                           169      1000  1000. NO ADDITIONAL SERVICE
                          3793         .
                           205        .j  .j. Ineligible: External site
                                          client

-----------------------------------------------------------------------------------------------
Ref1_DateServRec5                                      Referral 1: Date Service 5 Received
-----------------------------------------------------------------------------------------------

                  type:  numeric daily date (int)

                 range:  [19710,20025]                units:  1
       or equivalently:  [18dec2013,29oct2014]        units:  days
         unique values:  99                       missing .:  3792/4168
       unique mv codes:  2                       missing .*:  205/4168

                  mean:   19842.7 = 29apr2014 (+ 18 hours)
              std. dev:   69.2496

           percentiles:        10%       25%       50%       75%       90%
                              19760     19795     19821     19898     19947
                          06feb2014 13mar2014 08apr2014 24jun2014 12aug2014
```

```
-------------------------------------------------------------------------------------------
Ref1_ServDate5_sys                                 Referral 1: System Date Service 5 Received
-------------------------------------------------------------------------------------------

                  type:  numeric (double)

                 range:  [1.704e+12,1.730e+12]        units:  1000
         unique values:  151                       missing .:  3812/4168
       unique mv codes:  2                        missing .*:  205/4168

                  mean:   1.7e+12
              std. dev:   5.9e+09

           percentiles:        10%       25%       50%       75%       90%
                            1.7e+12   1.7e+12   1.7e+12   1.7e+12   1.7e+12

Ref1_ServDate5_sys:
  1.  some missing due to correcting date service received, but not knowing time service
      received

-------------------------------------------------------------------------------------------
Ref2_ID                                                                        Referral 2 ID
-------------------------------------------------------------------------------------------

                  type:  numeric (long)

                 range:  [268,99172]                  units:  1
         unique values:  759                       missing .:  3204/4168
       unique mv codes:  2                        missing .*:  205/4168

                  mean:   54161.1
              std. dev:   15312.8

           percentiles:        10%       25%       50%       75%       90%
                              44316     51113     56077     63125     70218

Ref2_ID:
  1.  if begins with 99, referral created for analysis

-------------------------------------------------------------------------------------------
Ref2_Source_an                                                             Referral 2 Source
-------------------------------------------------------------------------------------------

                  type:  numeric (byte)
                 label:  RefSource

                 range:  [1,2]                        units:  1
         unique values:  2                         missing .:  3204/4168
       unique mv codes:  2                        missing .*:  205/4168

            tabulation:  Freq.   Numeric  Label
                           755         1  1. Registration
                             4         2  2. Other Referral
                          3204         .
                           205        .j  .j. Ineligible: External site
                                          client

Ref2_Source_an:
  1.  created for analysis

-------------------------------------------------------------------------------------------
Ref2_ParentID                                       Referral 2 Parent ID: RegID or RefID
-------------------------------------------------------------------------------------------

                  type:  numeric (long)

                 range:  [265,86020]                  units:  1
         unique values:  759                       missing .:  3204/4168
       unique mv codes:  2                        missing .*:  205/4168
```

```
                 mean:   53653.5
             std. dev:   14838.1

          percentiles:        10%       25%       50%       75%       90%
                            44312     51106     56025     63007     70143

-----------------------------------------------------------------------------------------------
Ref2_Self_an                                                          Referral 2 for Respondent
-----------------------------------------------------------------------------------------------

                  type:  numeric (byte)
                 label:  yesno

                 range:  [0,1]                        units:  1
         unique values:  2                        missing .:  3204/4168
       unique mv codes:  2                       missing .*:  205/4168

            tabulation:  Freq.   Numeric  Label
                           253         0  0. No
                           506         1  1. Yes
                          3204         .
                           205        .j  .j. Ineligible: External site
                                          client

Ref2_Self_an:
  1.  created for analysis

-----------------------------------------------------------------------------------------------
Ref2_Partner_an                                              Referral 2 for Respondent's Partner
-----------------------------------------------------------------------------------------------

                  type:  numeric (byte)
                 label:  yesno

                 range:  [0,1]                        units:  1
         unique values:  2                        missing .:  3204/4168
       unique mv codes:  2                       missing .*:  205/4168

            tabulation:  Freq.   Numeric  Label
                           512         0  0. No
                           247         1  1. Yes
                          3204         .
                           205        .j  .j. Ineligible: External site
                                          client

Ref2_Partner_an:
  1.  created for analysis

-----------------------------------------------------------------------------------------------
Ref2_Other_an                                                              Referral 2 for Other
-----------------------------------------------------------------------------------------------

                  type:  numeric (byte)
                 label:  yesno

                 range:  [0,1]                        units:  1
         unique values:  2                        missing .:  3204/4168
       unique mv codes:  2                       missing .*:  205/4168

            tabulation:  Freq.   Numeric  Label
                           753         0  0. No
                             6         1  1. Yes
                          3204         .
                           205        .j  .j. Ineligible: External site
                                          client

Ref2_Other_an:
  1.  created for analysis
```

```
-------------------------------------------------------------------------------------
Ref2_PersID                       Referral 2 Given To: Respondent or Person Accompanying
-------------------------------------------------------------------------------------

                  type:  numeric (byte)
                 label:  RefPersID

                 range:  [0,0]                        units:  1
         unique values:  1                        missing .:  3204/4168
       unique mv codes:  2                       missing .*:  205/4168

            tabulation:  Freq.   Numeric  Label
                           759         0  0. Respondent
                          3204         .
                           205        .j  .j. Ineligible: External site
                                          client

-------------------------------------------------------------------------------------
Ref2_Date                                                      Referral 2: Date Given
-------------------------------------------------------------------------------------

                  type:  numeric daily date (int)

                 range:  [19711,20034]                units:  1
       or equivalently:  [19dec2013,07nov2014]        units:  days
         unique values:  159                      missing .:  3204/4168
       unique mv codes:  2                       missing .*:  205/4168

                  mean:   19830.6 = 17apr2014 (+ 15 hours)
              std. dev:   55.9934

           percentiles:        10%       25%       50%       75%       90%
                             19764     19792     19821     19872     19904
                         10feb2014 10mar2014 08apr2014 29may2014 30jun2014

-------------------------------------------------------------------------------------
Ref2_ReferralFOR                         Referral 2 For: Relationship to RefPersID_Ref2
-------------------------------------------------------------------------------------

                  type:  numeric (byte)
                 label:  relation

                 range:  [0,12]                       units:  1
         unique values:  8                        missing .:  3204/4168
       unique mv codes:  2                       missing .*:  205/4168

            tabulation:  Freq.   Numeric  Label
                           506         0  0. SELF
                           243         1  1. SPOUSE
                             4         2  2. COHABITATING PARTNER
                             1         4  4. SON
                             1         7  7. SISTER
                             1         8  8. BROTHER
                             1        11  11. OTHER RELATIVE
                             2        12  12. OTHER NON-RELATIVE
                          3204         .
                           205        .j  .j. Ineligible: External site
                                          client

-------------------------------------------------------------------------------------
Ref2_FromSite                                                    Referral 2 from Site
-------------------------------------------------------------------------------------

                  type:  numeric (byte)
                 label:  site

                 range:  [1,13]                       units:  1
         unique values:  9                        missing .:  3204/4168
       unique mv codes:  2                       missing .*:  205/4168
```

```
          tabulation:  Freq.   Numeric  Label
                          43         1  1. SFH HCT - Cairo Road
                         267         2  2. Chawama Clinic - MCH
                           1         3  3. Chawama Clinic - Out Patient
                                        Ward (VMMC)
                          13         5  5. Kamwala Clinic - TB, STI &
                                        HIV Clinic
                         247         6  6. Kapata Urban Clinic, MCH
                          70         7  7. Kapata Urban Clinic, TB, STI
                                        & HIV
                           5         8  8. Chipata Gen Hosp - OP VMMC
                         101        11  11. SFH New Start
                          12        13  13. SFH VMMC
                        3204         .
                         205        .j  .j. Ineligible: External site
                                        client

-------------------------------------------------------------------------------------------------
Ref2_ToSite                                                                  Referral 2 to Site
-------------------------------------------------------------------------------------------------

                type:  numeric (byte)
               label:  site

               range:  [1,13]                      units:  1
       unique values:  13                      missing .:  3204/4168
     unique mv codes:  2                      missing .*:  205/4168

          tabulation:  Freq.   Numeric  Label
                           2         1  1. SFH HCT - Cairo Road
                          22         2  2. Chawama Clinic - MCH
                         242         3  3. Chawama Clinic - Out Patient
                                        Ward (VMMC)
                          21         4  4. Chawama Clinic - TB, STI &
                                        HIV Clinic
                          13         5  5. Kamwala Clinic - TB, STI &
                                        HIV Clinic
                           2         6  6. Kapata Urban Clinic, MCH
                           8         7  7. Kapata Urban Clinic, TB, STI
                                        & HIV
                          52         8  8. Chipata Gen Hosp - OP VMMC
                         186         9  9. Chipata Gen Hosp - MCH
                          97        10  10. Chipata Gen Hosp - TB, STI &
                                        HIV
                          67        11  11. SFH New Start
                          24        12  12. SFH - VMMC - CHACHACHA RD
                          23        13  13. SFH VMMC
                        3204         .
                         205        .j  .j. Ineligible: External site
                                        client

-------------------------------------------------------------------------------------------------
Ref2_ServiceFor                                                           Referral 2 for Service
-------------------------------------------------------------------------------------------------

                type:  numeric (byte)
               label:  Service

               range:  [1,15]                      units:  1
       unique values:  13                      missing .:  3204/4168
     unique mv codes:  2                      missing .*:  205/4168

          tabulation:  Freq.   Numeric  Label
                         203         1  1. CCS: CERVICAL CANCER
                                        SCREENING
                           1         2  2. FP: GROUP COUNSELING
                           1         3  3. FP: INDIVIDUAL COUNSELING
                          11         4  4. FP: LONG TERM METHODS
                                        PROVIDED
                           1         5  5. FP: SHORT TERM METHODS
```

		PROVIDED
166	6	6. HIV: HTC - COUPLES
72	7	7. HIV: HTC - INDIVIDUAL
82	9	9. HIV: CD4 TESTING
1	10	10. HIV: PCYHOSOCIAL SUPPORT (PSS)
6	11	11. HIV: TB TESTING
34	12	12. STI: ASSESSMENT
1	13	13. STI: TREATMENT
180	15	15. VMMC: PROCEDURE
3204	.	
205	.j	.j. Ineligible: External site client

Ref2_ServiceFor:

1. Those who received referrals for couples HTC were coded in a specific manner based on the services they received prior. - If a respondent had received individual HTC, VMMC counseling or VMMC procedure prior to receiving the referral for couples HTC, s/he received one referral for couples HTC for his/her spouse or partner. - If a respondent did not receive individual HTC, VMMC counseling or VMMC procedure prior to receiving his/her referral for couples HTC, s/he received two referrals for couples HTC: one for self and one for spouse or partner.

Ref2_ShowDate — Referral 2: Date Return to Act On Referral

---

type: numeric daily date (int)

range: [19761,19960] units: 1
or equivalently: [07feb2014,25aug2014] units: days
unique values: 21 missing .: 3938/4168
unique mv codes: 2 missing .*: 205/4168

tabulation:

Freq.	Value	
1	19761	07feb2014
2	19771	17feb2014
1	19786	04mar2014
1	19788	06mar2014
1	19792	10mar2014
1	19814	01apr2014
2	19823	10apr2014
1	19835	22apr2014
2	19855	12may2014
1	19856	13may2014
2	19858	15may2014
1	19872	29may2014
1	19887	13jun2014
1	19893	19jun2014
1	19905	01jul2014
1	19907	03jul2014
1	19920	16jul2014
1	19925	21jul2014
1	19947	12aug2014
1	19954	19aug2014
1	19960	25aug2014
3938	.	.
205	.j	.j

Ref2_ShowHMM — Referral 2: Time Return to Act On Referral

---

type: string (str5)

unique values: 24 missing "": 4143/4168

tabulation:

Freq.	Value
4143	""
1	"09-00"

```
                          1  "09-05"
                          1  "09-07"
                          1  "09-15"
                          1  "09-17"
                          1  "09-19"
                          1  "09-25"
                          1  "09-30"
                          1  "09-33"
                          2  "09-40"
                          1  "09-57"
                          1  "10-00"
                          1  "10-10"
                          1  "10-20"
                          1  "10-39"
                          1  "10-43"
                          1  "11-19"
                          1  "11-24"
                          1  "11-41"
                          1  "11-45"
                          1  "12-07"
                          1  "12-31"
                          1  "12-37"
                          1  "13-06"

-------------------------------------------------------------------------------
Ref2_ServDate                               Referral 2: Date Services Received
-------------------------------------------------------------------------------

                  type:  numeric daily date (int)

                 range:  [19761,19954]                units:  1
       or equivalently:  [07feb2014,19aug2014]        units:  days
         unique values:  13                       missing .:  3950/4168
       unique mv codes:  2                       missing .*:  205/4168

            tabulation:  Freq.  Value
                             1  19761  07feb2014
                             1  19771  17feb2014
                             1  19806  24mar2014
                             1  19810  28mar2014
                             1  19814  01apr2014
                             1  19830  17apr2014
                             1  19845  02may2014
                             1  19862  19may2014
                             1  19872  29may2014
                             1  19905  01jul2014
                             1  19925  21jul2014
                             1  19947  12aug2014
                             1  19954  19aug2014
                          3950  .              .
                           205  .j            .j

-------------------------------------------------------------------------------
Ref2_ServHMM                                Referral 2: Time Services Received
-------------------------------------------------------------------------------

                  type:  string (str5)

         unique values:  8                        missing "":  4160/4168

            tabulation:  Freq.  Value
                          4160  ""
                             1  "10-21"
                             1  "10-49"
                             1  "10-50"
                             1  "11-46"
                             1  "12-10"
                             1  "12-38"
                             1  "14-17"
                             1  "15-54"
```

```
Ref2_ServHMM:
  1.  some missing due to correcting date service received, but not knowing time service
      received

-----------------------------------------------------------------------------------------------
Ref2_Counseling                                                 Referral 2: Counseling Received
-----------------------------------------------------------------------------------------------

                  type:  numeric (byte)
                 label:  Counseling

                 range:  [2,4]                        units:  1
         unique values:  2                        missing .:  3952/4168
       unique mv codes:  2                       missing .*:  205/4168

            tabulation:  Freq.   Numeric  Label
                             8         2  2. ONE-ON-ONE
                             3         4  4. NONE
                          3952         .
                           205        .j  .j. Ineligible: External site
                                          client

-----------------------------------------------------------------------------------------------
Ref2_ReceivedDayReturned                        Referral 2: Services Received Same Day Returned
-----------------------------------------------------------------------------------------------

                  type:  numeric (byte)
                 label:  yesno

                 range:  [1,1]                        units:  1
         unique values:  1                        missing .:  3952/4168
       unique mv codes:  2                       missing .*:  205/4168

            tabulation:  Freq.   Numeric  Label
                            11         1  1. Yes
                          3952         .
                           205        .j  .j. Ineligible: External site
                                          client

Ref2_ReceivedDayReturned:
  1.  Yes if Date showed up for services = Date services received
      (Ref1_ShowDate=Ref1_ServDate)

-----------------------------------------------------------------------------------------------
Ref2_Escort                                                         Referral 2: Escort Provided
-----------------------------------------------------------------------------------------------

                  type:  numeric (byte)
                 label:  Ref2_Escort

                 range:  [0,1]                        units:  1
         unique values:  2                        missing .:  0/4168
       unique mv codes:  6                       missing .*:  4159/4168

            tabulation:  Freq.   Numeric  Label
                             1         0  0. No
                             8         1  1. Yes
                          2642        .a  .a. Not in integrated arm
                           959        .b  .b. No referral
                             1        .c  .c. Received services same
                                          day/same site as referral 1:
                                          escort recorded in Ref1_Escort
                           350        .d  .d. Have not yet receieved
                                          services from referral
                           205        .j  .j. Ineligible: External site
                                          client
                             2        .m  .m. Missing

Ref2_Escort:
```

1. Those with value of .c received services the same day/same site as referral 1. Check Ref1_Escort to know whether they received an escort. Their escort data is recorded only in Ref1_Escort to enable an accurate count of escorts. (If Ref1_Escort == 1, that is the escort who brought the client to one site to get services from referral 1 and referral 2.)

```
--------------------------------------------------------------------------------------------
Ref2_EscortTime                                             Referral 2: Time Escort Provided
--------------------------------------------------------------------------------------------

                  type:  string (str5)

         unique values:  8                        missing "":  4160/4168

           tabulation:  Freq.  Value
                         4160  ""
                            1  "09-33"
                            1  "09-43"
                            1  "10-50"
                            1  "11-00"
                            1  "11-47"
                            1  "12-11"
                            1  "12-40"
                            1  "13-08"

--------------------------------------------------------------------------------------------
Ref2_VoucherNumber                                                 Referral 2 Voucher Number
--------------------------------------------------------------------------------------------

                  type:  string (str10)

         unique values:  759                      missing "":  3409/4168

             examples:  ""
                        ""
                        ""
                        ""

Ref2_VoucherNumber:
  1.  if begins with 99, referral created for analysis

--------------------------------------------------------------------------------------------
Ref2_ServCameFor1                                             Referral 2: Service 1 Came For
--------------------------------------------------------------------------------------------

                  type:  numeric (byte)
                 label:  Service

                 range:  [1,15]                       units:  1
         unique values:  5                        missing .:  3938/4168
       unique mv codes:  2                       missing .*:  205/4168

           tabulation:  Freq.   Numeric  Label
                            7         1  1. CCS: CERVICAL CANCER
                                         SCREENING
                            5         6  6. HIV: HTC - COUPLES
                            6         9  9. HIV: CD4 TESTING
                            6        12  12. STI: ASSESSMENT
                            1        15  15. VMMC: PROCEDURE
                         3938         .  
                          205        .j  .j. Ineligible: External site
                                         client

--------------------------------------------------------------------------------------------
Ref2_ServRecv1                                                Referral 2: Service 1 Received
--------------------------------------------------------------------------------------------

                  type:  numeric (byte)
                 label:  Service
```

```
               range:  [1,15]                       units:  1
       unique values:  5                        missing .:  3950/4168
     unique mv codes:  2                       missing .*:  205/4168

          tabulation:  Freq.   Numeric  Label
                           5         1  1. CCS: CERVICAL CANCER
                                        SCREENING
                           2         6  6. HIV: HTC - COUPLES
                           2         9  9. HIV: CD4 TESTING
                           3        12  12. STI: ASSESSMENT
                           1        15  15. VMMC: PROCEDURE
                        3950         .
                         205        .j  .j. Ineligible: External site
                                        client

-------------------------------------------------------------------------------------------------
Ref2_DateServRec1                                           Referral 2: Date Service 1 Received
-------------------------------------------------------------------------------------------------

                type:  numeric daily date (int)

               range:  [19761,19954]                units:  1
     or equivalently:  [07feb2014,19aug2014]        units:  days
       unique values:  13                       missing .:  3950/4168
     unique mv codes:  2                       missing .*:  205/4168

          tabulation:  Freq.  Value
                           1  19761  07feb2014
                           1  19771  17feb2014
                           1  19806  24mar2014
                           1  19810  28mar2014
                           1  19814  01apr2014
                           1  19830  17apr2014
                           1  19845  02may2014
                           1  19862  19may2014
                           1  19872  29may2014
                           1  19905  01jul2014
                           1  19925  21jul2014
                           1  19947  12aug2014
                           1  19954  19aug2014
                        3950  .              .
                         205  .j            .j

-------------------------------------------------------------------------------------------------
Ref2_ServDate1_sys                                   Referral 2: System Date Service 1 Received
-------------------------------------------------------------------------------------------------

                type:  numeric (double)

               range:  [1.707e+12,1.724e+12]        units:  10000
       unique values:  8                        missing .:  3955/4168
     unique mv codes:  2                       missing .*:  205/4168

          tabulation:  Freq.  Value
                           1  1.707e+12
                           1  1.708e+12
                           1  1.712e+12
                           1  1.717e+12
                           1  1.720e+12
                           1  1.722e+12
                           1  1.723e+12
                           1  1.724e+12
                        3955  .
                         205  .j

Ref2_ServDate1_sys:
  1.  some missing due to correcting date service received, but not knowing time service
      received

-------------------------------------------------------------------------------------------------
```

```
Ref2_ServCameFor2                                  Referral 2: Service 2 Came For
-------------------------------------------------------------------------------------------

                  type:  numeric (int)
                 label:  Service

                 range:  [1000,1000]                  units:  1
         unique values:  1                        missing .:  3938/4168
       unique mv codes:  2                       missing .*:  205/4168

            tabulation:  Freq.   Numeric  Label
                            25      1000  1000. NO ADDITIONAL SERVICE
                          3938         .
                           205        .j  .j. Ineligible: External site
                                          client

-------------------------------------------------------------------------------------------
Ref2_ServRecv2                                     Referral 2: Service 2 Received
-------------------------------------------------------------------------------------------

                  type:  numeric (int)
                 label:  Service

                 range:  [1000,1000]                  units:  1
         unique values:  1                        missing .:  3950/4168
       unique mv codes:  2                       missing .*:  205/4168

            tabulation:  Freq.   Numeric  Label
                            13      1000  1000. NO ADDITIONAL SERVICE
                          3950         .
                           205        .j  .j. Ineligible: External site
                                          client

-------------------------------------------------------------------------------------------
Ref2_DateServRec2                             Referral 2: Date Service 2 Received
-------------------------------------------------------------------------------------------

                  type:  numeric daily date (int)

                 range:  [19761,19954]                units:  1
       or equivalently:  [07feb2014,19aug2014]        units:  days
         unique values:  13                       missing .:  3950/4168
       unique mv codes:  2                       missing .*:  205/4168

            tabulation:  Freq.  Value
                             1  19761  07feb2014
                             1  19771  17feb2014
                             1  19806  24mar2014
                             1  19810  28mar2014
                             1  19814  01apr2014
                             1  19830  17apr2014
                             1  19845  02may2014
                             1  19862  19may2014
                             1  19872  29may2014
                             1  19905  01jul2014
                             1  19925  21jul2014
                             1  19947  12aug2014
                             1  19954  19aug2014
                          3950  .              .
                           205  .j            .j

-------------------------------------------------------------------------------------------
Ref2_ServDate2_sys                     Referral 2: System Date Service 2 Received
-------------------------------------------------------------------------------------------

                  type:  numeric (double)

                 range:  [1.707e+12,1.724e+12]        units:  10000
         unique values:  8                        missing .:  3955/4168
       unique mv codes:  2                       missing .*:  205/4168
```

```
           tabulation:  Freq.  Value
                            1  1.707e+12
                            1  1.708e+12
                            1  1.712e+12
                            1  1.717e+12
                            1  1.720e+12
                            1  1.722e+12
                            1  1.723e+12
                            1  1.724e+12
                         3955  .
                          205  .j

Ref2_ServDate2_sys:
  1.  some missing due to correcting date service received, but not knowing time service
      received

-----------------------------------------------------------------------------------------------
Ref2_ServCameFor3                                              Referral 2: Service 3 Came For
-----------------------------------------------------------------------------------------------

                 type:  numeric (int)
                label:  Service

                range:  [1000,1000]                  units:  1
        unique values:  1                        missing .:  3938/4168
      unique mv codes:  2                       missing .*:  205/4168

           tabulation:  Freq.   Numeric  Label
                           25      1000  1000. NO ADDITIONAL SERVICE
                         3938         .
                          205        .j  .j. Ineligible: External site
                                         client

-----------------------------------------------------------------------------------------------
Ref2_ServRecv3                                                 Referral 2: Service 3 Received
-----------------------------------------------------------------------------------------------

                 type:  numeric (int)
                label:  Service

                range:  [1000,1000]                  units:  1
        unique values:  1                        missing .:  3950/4168
      unique mv codes:  2                       missing .*:  205/4168

           tabulation:  Freq.   Numeric  Label
                           13      1000  1000. NO ADDITIONAL SERVICE
                         3950         .
                          205        .j  .j. Ineligible: External site
                                         client

-----------------------------------------------------------------------------------------------
Ref2_DateServRec3                                         Referral 2: Date Service 3 Received
-----------------------------------------------------------------------------------------------

                 type:  numeric daily date (int)

                range:  [19761,19954]                units:  1
      or equivalently:  [07feb2014,19aug2014]        units:  days
        unique values:  13                       missing .:  3950/4168
      unique mv codes:  2                       missing .*:  205/4168

           tabulation:  Freq.  Value
                            1  19761  07feb2014
                            1  19771  17feb2014
                            1  19806  24mar2014
                            1  19810  28mar2014
                            1  19814  01apr2014
                            1  19830  17apr2014
                            1  19845  02may2014
```

```
                          1  19862  19may2014
                          1  19872  29may2014
                          1  19905  01jul2014
                          1  19925  21jul2014
                          1  19947  12aug2014
                          1  19954  19aug2014
                       3950  .              .
                        205  .j            .j

-----------------------------------------------------------------------------------------------
Ref2_ServDate3_sys                                  Referral 2: System Date Service 3 Received
-----------------------------------------------------------------------------------------------

                  type:  numeric (double)

                 range:  [1.707e+12,1.724e+12]        units:  10000
         unique values:  8                        missing .:  3955/4168
       unique mv codes:  2                       missing .*:  205/4168

            tabulation:  Freq.  Value
                             1  1.707e+12
                             1  1.708e+12
                             1  1.712e+12
                             1  1.717e+12
                             1  1.720e+12
                             1  1.722e+12
                             1  1.723e+12
                             1  1.724e+12
                          3955  .
                           205  .j

Ref2_ServDate3_sys:
  1.  some missing due to correcting date service received, but not knowing time service
      received

-----------------------------------------------------------------------------------------------
Ref2_ServCameFor4                                               Referral 2: Service 4 Came For
-----------------------------------------------------------------------------------------------

                  type:  numeric (int)
                 label:  Service

                 range:  [1000,1000]                  units:  1
         unique values:  1                        missing .:  3938/4168
       unique mv codes:  2                       missing .*:  205/4168

            tabulation:  Freq.   Numeric  Label
                            25      1000  1000. NO ADDITIONAL SERVICE
                          3938         .
                           205        .j  .j. Ineligible: External site
                                          client

-----------------------------------------------------------------------------------------------
Ref2_ServRecv4                                                  Referral 2: Service 4 Received
-----------------------------------------------------------------------------------------------

                  type:  numeric (int)
                 label:  Service

                 range:  [1000,1000]                  units:  1
         unique values:  1                        missing .:  3950/4168
       unique mv codes:  2                       missing .*:  205/4168

            tabulation:  Freq.   Numeric  Label
                            13      1000  1000. NO ADDITIONAL SERVICE
                          3950         .
                           205        .j  .j. Ineligible: External site
                                          client

-----------------------------------------------------------------------------------------------
```

```
Ref2_DateServRec4                                    Referral 2: Date Service 4 Received
-----------------------------------------------------------------------------------------------

                  type:  numeric daily date (int)

                 range:  [19761,19954]                units:  1
       or equivalently:  [07feb2014,19aug2014]        units:  days
         unique values:  13                       missing .:  3950/4168
       unique mv codes:  2                       missing .*:  205/4168

            tabulation:  Freq.  Value
                             1  19761  07feb2014
                             1  19771  17feb2014
                             1  19806  24mar2014
                             1  19810  28mar2014
                             1  19814  01apr2014
                             1  19830  17apr2014
                             1  19845  02may2014
                             1  19862  19may2014
                             1  19872  29may2014
                             1  19905  01jul2014
                             1  19925  21jul2014
                             1  19947  12aug2014
                             1  19954  19aug2014
                          3950  .              .
                           205  .j            .j

-----------------------------------------------------------------------------------------------
Ref2_ServDate4_sys                            Referral 2: System Date Service 4 Received
-----------------------------------------------------------------------------------------------

                  type:  numeric (double)

                 range:  [1.707e+12,1.724e+12]        units:  10000
         unique values:  8                        missing .:  3955/4168
       unique mv codes:  2                       missing .*:  205/4168

            tabulation:  Freq.  Value
                             1  1.707e+12
                             1  1.708e+12
                             1  1.712e+12
                             1  1.717e+12
                             1  1.720e+12
                             1  1.722e+12
                             1  1.723e+12
                             1  1.724e+12
                          3955  .
                           205  .j

Ref2_ServDate4_sys:
  1.  some missing due to correcting date service received, but not knowing time service
      received

-----------------------------------------------------------------------------------------------
Ref2_ServCameFor5                                         Referral 2: Service 5 Came For
-----------------------------------------------------------------------------------------------

                  type:  numeric (int)
                 label:  Service

                 range:  [1000,1000]                  units:  1
         unique values:  1                        missing .:  3938/4168
       unique mv codes:  2                       missing .*:  205/4168

            tabulation:  Freq.   Numeric  Label
                            25      1000  1000. NO ADDITIONAL SERVICE
                          3938         .
                           205        .j  .j. Ineligible: External site
                                          client
```

```
--------------------------------------------------------------------------------------------
Ref2_ServRecv5                                            Referral 2: Service 5 Received
--------------------------------------------------------------------------------------------

                  type:  numeric (int)
                 label:  Service

                 range:  [1000,1000]                  units:  1
         unique values:  1                        missing .:  3950/4168
       unique mv codes:  2                       missing .*:  205/4168

            tabulation:  Freq.   Numeric  Label
                            13      1000  1000. NO ADDITIONAL SERVICE
                          3950         .
                           205        .j  .j. Ineligible: External site
                                          client

--------------------------------------------------------------------------------------------
Ref2_DateServRec5                                    Referral 2: Date Service 5 Received
--------------------------------------------------------------------------------------------

                  type:  numeric daily date (int)

                 range:  [19761,19954]                units:  1
       or equivalently:  [07feb2014,19aug2014]        units:  days
         unique values:  13                       missing .:  3950/4168
       unique mv codes:  2                       missing .*:  205/4168

            tabulation:  Freq.  Value
                             1  19761  07feb2014
                             1  19771  17feb2014
                             1  19806  24mar2014
                             1  19810  28mar2014
                             1  19814  01apr2014
                             1  19830  17apr2014
                             1  19845  02may2014
                             1  19862  19may2014
                             1  19872  29may2014
                             1  19905  01jul2014
                             1  19925  21jul2014
                             1  19947  12aug2014
                             1  19954  19aug2014
                          3950  .              .
                           205  .j            .j

--------------------------------------------------------------------------------------------
Ref2_ServDate5_sys                            Referral 2: System Date Service 5 Received
--------------------------------------------------------------------------------------------

                  type:  numeric (double)

                 range:  [1.707e+12,1.724e+12]        units:  10000
         unique values:  8                        missing .:  3955/4168
       unique mv codes:  2                       missing .*:  205/4168

            tabulation:  Freq.  Value
                             1  1.707e+12
                             1  1.708e+12
                             1  1.712e+12
                             1  1.717e+12
                             1  1.720e+12
                             1  1.722e+12
                             1  1.723e+12
                             1  1.724e+12
                          3955  .
                           205  .j

Ref2_ServDate5_sys:
  1.  some missing due to correcting date service received, but not knowing time service
      received
```

```
-------------------------------------------------------------------------------------------------
Ref3_ID                                                                           Referral 3 ID
-------------------------------------------------------------------------------------------------

                  type:  numeric (long)

                 range:  [279,99194]                  units:  1
         unique values:  273                      missing .:  3690/4168
       unique mv codes:  2                       missing .*:  205/4168

                  mean:   66250.5
              std. dev:   24881.5

           percentiles:        10%       25%       50%       75%       90%
                             51063     51384     56563     99018     99129

Ref3_ID:
  1.  if begins with 99, referral created for analysis

-------------------------------------------------------------------------------------------------
Ref3_Source_an                                                                Referral 3 Source
-------------------------------------------------------------------------------------------------

                  type:  numeric (byte)
                 label:  RefSource

                 range:  [1,2]                        units:  1
         unique values:  2                        missing .:  3690/4168
       unique mv codes:  2                       missing .*:  205/4168

            tabulation:  Freq.   Numeric  Label
                           269         1  1. Registration
                             4         2  2. Other Referral
                          3690         .
                           205        .j  .j. Ineligible: External site
                                          client

Ref3_Source_an:
  1.  created for analysis

-------------------------------------------------------------------------------------------------
Ref3_ParentID                                                Referral 3 Parent ID: RegID or RefID
-------------------------------------------------------------------------------------------------

                  type:  numeric (long)

                 range:  [265,86020]                  units:  1
         unique values:  273                      missing .:  3690/4168
       unique mv codes:  2                       missing .*:  205/4168

                  mean:   52884.1
              std. dev:   15853.8

           percentiles:        10%       25%       50%       75%       90%
                             51002     51220     51630     56775     70097

-------------------------------------------------------------------------------------------------
Ref3_Self_an                                                           Referral 3 for Respondent
-------------------------------------------------------------------------------------------------

                  type:  numeric (byte)
                 label:  yesno

                 range:  [0,1]                        units:  1
         unique values:  2                        missing .:  3690/4168
       unique mv codes:  2                       missing .*:  205/4168

            tabulation:  Freq.   Numeric  Label
                           175         0  0. No
```

```
                           98         1  1. Yes
                         3690         .
                          205        .j  .j. Ineligible: External site
                                         client

Ref3_Self_an:
  1.  created for analysis

-----------------------------------------------------------------------------------------------
Ref3_Partner_an                                              Referral 3 for Respondent's Partner
-----------------------------------------------------------------------------------------------

                  type:  numeric (byte)
                 label:  yesno

                 range:  [0,1]                        units:  1
         unique values:  2                        missing .:  3690/4168
       unique mv codes:  2                       missing .*:  205/4168

            tabulation:  Freq.   Numeric  Label
                          114         0  0. No
                          159         1  1. Yes
                         3690         .
                          205        .j  .j. Ineligible: External site
                                         client

Ref3_Partner_an:
  1.  created for analysis

-----------------------------------------------------------------------------------------------
Ref3_Other_an                                                              Referral 3 for Other
-----------------------------------------------------------------------------------------------

                  type:  numeric (byte)
                 label:  yesno

                 range:  [0,1]                        units:  1
         unique values:  2                        missing .:  3690/4168
       unique mv codes:  2                       missing .*:  205/4168

            tabulation:  Freq.   Numeric  Label
                          257         0  0. No
                           16         1  1. Yes
                         3690         .
                          205        .j  .j. Ineligible: External site
                                         client

Ref3_Other_an:
  1.  created for analysis

-----------------------------------------------------------------------------------------------
Ref3_PersID                                Referral 3 Given To: Respondent or Person Accompanying
-----------------------------------------------------------------------------------------------

                  type:  numeric (byte)
                 label:  RefPersID

                 range:  [0,0]                        units:  1
         unique values:  1                        missing .:  3690/4168
       unique mv codes:  2                       missing .*:  205/4168

            tabulation:  Freq.   Numeric  Label
                          273         0  0. Respondent
                         3690         .
                          205        .j  .j. Ineligible: External site
                                         client

-----------------------------------------------------------------------------------------------
Ref3_Date                                                               Referral 3: Date Given
-----------------------------------------------------------------------------------------------
```

```
                 type:  numeric daily date (int)

                range:  [19712,19996]                units:  1
     or equivalently:  [20dec2013,30sep2014]        units:  days
        unique values:  112                      missing .:  3690/4168
      unique mv codes:  2                       missing .*:  205/4168

                 mean:   19820.6 = 07apr2014 (+ 13 hours)
            std. dev:   51.3441

          percentiles:        10%       25%       50%       75%       90%
                            19764     19782     19813     19859     19890
                        10feb2014 28feb2014 31mar2014 16may2014 16jun2014
```

```
--------------------------------------------------------------------------------
Ref3_ReferralFOR                          Referral 3 For: Relationship to RefPersID_Ref3
--------------------------------------------------------------------------------

                 type:  numeric (byte)
                label:  relation

                range:  [0,12]                       units:  1
        unique values:  6                        missing .:  3690/4168
      unique mv codes:  2                       missing .*:  205/4168

           tabulation:  Freq.   Numeric  Label
                           98         0  0. SELF
                          156         1  1. SPOUSE
                            3         2  2. COHABITATING PARTNER
                            1         3  3. DAUGHTER
                            1         4  4. SON
                           14        12  12. OTHER NON-RELATIVE
                         3690         .
                          205        .j  .j. Ineligible: External site
                                         client
```

```
--------------------------------------------------------------------------------
Ref3_FromSite                                                   Referral 3 from Site
--------------------------------------------------------------------------------

                 type:  numeric (byte)
                label:  site

                range:  [1,13]                       units:  1
        unique values:  9                        missing .:  3690/4168
      unique mv codes:  2                       missing .*:  205/4168

           tabulation:  Freq.   Numeric  Label
                            1         1  1. SFH HCT - Cairo Road
                          191         2  2. Chawama Clinic - MCH
                            1         3  3. Chawama Clinic - Out Patient
                                         Ward (VMMC)
                            1         5  5. Kamwala Clinic - TB, STI &
                                         HIV Clinic
                           58         6  6. Kapata Urban Clinic, MCH
                            2         7  7. Kapata Urban Clinic, TB, STI
                                         & HIV
                            3         8  8. Chipata Gen Hosp - OP VMMC
                           14        11  11. SFH New Start
                            2        13  13. SFH VMMC
                         3690         .
                          205        .j  .j. Ineligible: External site
                                         client
```

```
--------------------------------------------------------------------------------
Ref3_ToSite                                                       Referral 3 to Site
--------------------------------------------------------------------------------

                 type:  numeric (byte)
```

```
               label:  site

               range:  [2,13]                       units:  1
       unique values:  11                       missing .:  3690/4168
     unique mv codes:  2                       missing .*:  205/4168

          tabulation:  Freq.   Numeric  Label
                           3         2  2. Chawama Clinic - MCH
                         169         3  3. Chawama Clinic - Out Patient
                                        Ward (VMMC)
                          21         4  4. Chawama Clinic - TB, STI &
                                        HIV Clinic
                           1         6  6. Kapata Urban Clinic, MCH
                           4         7  7. Kapata Urban Clinic, TB, STI
                                        & HIV
                           5         8  8. Chipata Gen Hosp - OP VMMC
                           9         9  9. Chipata Gen Hosp - MCH
                          24        10  10. Chipata Gen Hosp - TB, STI &
                                        HIV
                          31        11  11. SFH New Start
                           1        12  12. SFH - VMMC - CHACHACHA RD
                           5        13  13. SFH VMMC
                        3690         .
                         205        .j  .j. Ineligible: External site
                                        client

-----------------------------------------------------------------------------------------------
Ref3_ServiceFor                                                          Referral 3 for Service
-----------------------------------------------------------------------------------------------

                type:  numeric (byte)
               label:  Service

               range:  [1,15]                       units:  1
       unique values:  9                        missing .:  3690/4168
     unique mv codes:  2                       missing .*:  205/4168

          tabulation:  Freq.   Numeric  Label
                          11         1  1. CCS: CERVICAL CANCER
                                        SCREENING
                           2         4  4. FP: LONG TERM METHODS
                                        PROVIDED
                         134         6  6. HIV: HTC - COUPLES
                          17         7  7. HIV: HTC - INDIVIDUAL
                          25         9  9. HIV: CD4 TESTING
                           1        10  10. HIV: PCYHOSOCIAL SUPPORT
                                        (PSS)
                           1        11  11. HIV: TB TESTING
                           1        12  12. STI: ASSESSMENT
                          81        15  15. VMMC: PROCEDURE
                        3690         .
                         205        .j  .j. Ineligible: External site
                                        client

Ref3_ServiceFor:
  1.  Those who received referrals for couples HTC were coded in a specific manner based on
      the services they received prior.  - If a respondent had received individual HTC, VMMC
      counseling or VMMC procedure prior to receiving the referral for couples HTC, s/he
      received one referral for couples HTC for his/her spouse or partner.  - If a respondent
      did not receive individual HTC, VMMC counseling or VMMC procedure prior to receiving
      his/her referral for couples HTC, s/he received two referrals for couples HTC: one for
      self and one for spouse or partner.

-----------------------------------------------------------------------------------------------
Ref3_ShowDate                                       Referral 3: Date Return to Act On Referral
-----------------------------------------------------------------------------------------------

                type:  numeric daily date (int)

               range:  [19823,19907]                units:  1
```

```
       or equivalently:  [10apr2014,03jul2014]        units:  days
         unique values:  2                        missing .:  3961/4168
       unique mv codes:  2                       missing .*:  205/4168

            tabulation:  Freq.  Value
                             1  19823  10apr2014
                             1  19907  03jul2014
                          3961  .              .
                           205  .j            .j

-------------------------------------------------------------------------------
Ref3_ShowHMM                                Referral 3: Time Return to Act On Referral
-------------------------------------------------------------------------------

                  type:  string (str5)

         unique values:  2                        missing "":  4166/4168

            tabulation:  Freq.  Value
                          4166  ""
                             1  "09-07"
                             1  "12-31"

-------------------------------------------------------------------------------
Ref3_ServDate                                      Referral 3: Date Services Received
-------------------------------------------------------------------------------

                  type:  numeric daily date (int)

                 range:  [19806,19806]                units:  1
       or equivalently:  [24mar2014,24mar2014]        units:  days
         unique values:  1                        missing .:  3962/4168
       unique mv codes:  2                       missing .*:  205/4168

            tabulation:  Freq.  Value
                             1  19806  24mar2014
                          3962  .              .
                           205  .j            .j

-------------------------------------------------------------------------------
Ref3_ServHMM                                       Referral 3: Time Services Received
-------------------------------------------------------------------------------

                  type:  string (str1), but longest is str0

         unique values:  0                        missing "":  4168/4168

            tabulation:  Freq.  Value
                          4168  ""

Ref3_ServHMM:
  1.  some missing due to correcting date service received, but not knowing time service
      received

-------------------------------------------------------------------------------
Ref3_Counseling                                       Referral 3: Counseling Received
-------------------------------------------------------------------------------

                  type:  numeric (byte)
                 label:  Counseling

                 range:  [.,.]                        units:  .
         unique values:  0                        missing .:  3963/4168
       unique mv codes:  2                       missing .*:  205/4168

            tabulation:  Freq.   Numeric  Label
                          3963         .
                           205        .j  .j. Ineligible: External site
                                          client
```

```
-------------------------------------------------------------------------------------------
Ref3_ReceivedDayReturned                      Referral 3: Services Received Same Day Returned
-------------------------------------------------------------------------------------------

                  type:  numeric (byte)
                 label:  yesno

                 range:  [.,.]                         units:  .
         unique values:  0                         missing .:  3963/4168
       unique mv codes:  2                        missing .*:  205/4168

            tabulation:  Freq.   Numeric  Label
                          3963         .
                           205        .j  .j. Ineligible: External site
                                          client

Ref3_ReceivedDayReturned:
  1.  Yes if Date showed up for services = Date services received
      (Ref1_ShowDate=Ref1_ServDate)

-------------------------------------------------------------------------------------------
Ref3_Escort                                                   Referral 3: Escort Provided
-------------------------------------------------------------------------------------------

                  type:  numeric (byte)
                 label:  Ref3_Escort

                 range:  [.,.]                         units:  .
         unique values:  0                         missing .:  0/4168
       unique mv codes:  5                        missing .*:  4168/4168

            tabulation:  Freq.   Numeric  Label
                          2642        .a  .a. Not in integrated arm
                          1187        .b  .b. No referral
                           133        .d  .d. Have not yet receieved
                                          services from referral
                           205        .j  .j. Ineligible: External site
                                          client
                             1        .m  .m. Missing

-------------------------------------------------------------------------------------------
Ref3_EscortTime                                          Referral 3: Time Escort Provided
-------------------------------------------------------------------------------------------

                  type:  string (str1), but longest is str0

         unique values:  0                         missing "":  4168/4168

            tabulation:  Freq.  Value
                          4168  ""

-------------------------------------------------------------------------------------------
Ref3_VoucherNumber                                             Referral 3 Voucher Number
-------------------------------------------------------------------------------------------

                  type:  string (str10)

         unique values:  273                       missing "":  3895/4168

              examples:  ""
                         ""
                         ""
                         ""

Ref3_VoucherNumber:
  1.  if begins with 99, referral created for analysis

-------------------------------------------------------------------------------------------
Ref3_ServCameFor1                                          Referral 3: Service 1 Came For
-------------------------------------------------------------------------------------------
```

```
                 type:  numeric (byte)
                label:  Service

                range:  [6,6]                        units:  1
        unique values:  1                        missing .:  3961/4168
      unique mv codes:  2                       missing .*:  205/4168

           tabulation:  Freq.   Numeric  Label
                            2         6  6. HIV: HTC - COUPLES
                         3961         .
                          205        .j  .j. Ineligible: External site
                                         client
```

```
--------------------------------------------------------------------------------------------
Ref3_ServRecv1                                             Referral 3: Service 1 Received
--------------------------------------------------------------------------------------------

                 type:  numeric (byte)
                label:  Service

                range:  [6,6]                        units:  1
        unique values:  1                        missing .:  3962/4168
      unique mv codes:  2                       missing .*:  205/4168

           tabulation:  Freq.   Numeric  Label
                            1         6  6. HIV: HTC - COUPLES
                         3962         .
                          205        .j  .j. Ineligible: External site
                                         client
```

```
--------------------------------------------------------------------------------------------
Ref3_DateServRec1                                     Referral 3: Date Service 1 Received
--------------------------------------------------------------------------------------------

                 type:  numeric daily date (int)

                range:  [19806,19806]                units:  1
      or equivalently:  [24mar2014,24mar2014]        units:  days
        unique values:  1                        missing .:  3962/4168
      unique mv codes:  2                       missing .*:  205/4168

           tabulation:  Freq.  Value
                            1  19806  24mar2014
                         3962  .              .
                          205  .j            .j
```

```
--------------------------------------------------------------------------------------------
Ref3_ServDate1_sys                             Referral 3: System Date Service 1 Received
--------------------------------------------------------------------------------------------

                 type:  numeric (byte)

                range:  [.,.]                        units:  .
        unique values:  0                        missing .:  3963/4168
      unique mv codes:  2                       missing .*:  205/4168

           tabulation:  Freq.  Value
                         3963  .
                          205  .j

Ref3_ServDate1_sys:
  1.  some missing due to correcting date service received, but not knowing time service
      received
```

```
--------------------------------------------------------------------------------------------
Ref3_ServCameFor2                                          Referral 3: Service 2 Came For
--------------------------------------------------------------------------------------------

                 type:  numeric (int)
```

```
                label:  Service

                range:  [1000,1000]                  units:  1
        unique values:  1                        missing .:  3961/4168
      unique mv codes:  2                       missing .*:  205/4168

           tabulation:  Freq.   Numeric  Label
                            2      1000  1000. NO ADDITIONAL SERVICE
                         3961         .
                          205        .j  .j. Ineligible: External site
                                         client
```

```
-----------------------------------------------------------------------------------------------
Ref3_ServRecv2                                                    Referral 3: Service 2 Received
-----------------------------------------------------------------------------------------------

                 type:  numeric (int)
                label:  Service

                range:  [1000,1000]                  units:  1000
        unique values:  1                        missing .:  3962/4168
      unique mv codes:  2                       missing .*:  205/4168

           tabulation:  Freq.   Numeric  Label
                            1      1000  1000. NO ADDITIONAL SERVICE
                         3962         .
                          205        .j  .j. Ineligible: External site
                                         client
```

```
-----------------------------------------------------------------------------------------------
Ref3_DateServRec2                                            Referral 3: Date Service 2 Received
-----------------------------------------------------------------------------------------------

                 type:  numeric daily date (int)

                range:  [19806,19806]                units:  1
     or equivalently:  [24mar2014,24mar2014]        units:  days
        unique values:  1                        missing .:  3962/4168
      unique mv codes:  2                       missing .*:  205/4168

           tabulation:  Freq.  Value
                            1  19806  24mar2014
                         3962  .              .
                          205  .j            .j
```

```
-----------------------------------------------------------------------------------------------
Ref3_ServDate2_sys                                    Referral 3: System Date Service 2 Received
-----------------------------------------------------------------------------------------------

                 type:  numeric (byte)

                range:  [.,.]                        units:  .
        unique values:  0                        missing .:  3963/4168
      unique mv codes:  2                       missing .*:  205/4168

           tabulation:  Freq.  Value
                         3963  .
                          205  .j

Ref3_ServDate2_sys:
  1.  some missing due to correcting date service received, but not knowing time service
      received
```

```
-----------------------------------------------------------------------------------------------
Ref3_ServCameFor3                                                 Referral 3: Service 3 Came For
-----------------------------------------------------------------------------------------------

                 type:  numeric (int)
                label:  Service
```

```
                range:  [1000,1000]                  units:  1
        unique values:  1                        missing .:  3961/4168
      unique mv codes:  2                       missing .*:  205/4168

           tabulation:  Freq.   Numeric  Label
                            2      1000  1000. NO ADDITIONAL SERVICE
                         3961         .
                          205        .j  .j. Ineligible: External site
                                         client

-----------------------------------------------------------------------------------------------
Ref3_ServRecv3                                               Referral 3: Service 3 Received
-----------------------------------------------------------------------------------------------

                 type:  numeric (int)
                label:  Service

                range:  [1000,1000]                  units:  1000
        unique values:  1                        missing .:  3962/4168
      unique mv codes:  2                       missing .*:  205/4168

           tabulation:  Freq.   Numeric  Label
                            1      1000  1000. NO ADDITIONAL SERVICE
                         3962         .
                          205        .j  .j. Ineligible: External site
                                         client

-----------------------------------------------------------------------------------------------
Ref3_DateServRec3                                       Referral 3: Date Service 3 Received
-----------------------------------------------------------------------------------------------

                 type:  numeric daily date (int)

                range:  [19806,19806]                units:  1
     or equivalently:  [24mar2014,24mar2014]        units:  days
        unique values:  1                        missing .:  3962/4168
      unique mv codes:  2                       missing .*:  205/4168

           tabulation:  Freq.  Value
                            1  19806  24mar2014
                         3962  .              .
                          205  .j            .j

-----------------------------------------------------------------------------------------------
Ref3_ServDate3_sys                               Referral 3: System Date Service 3 Received
-----------------------------------------------------------------------------------------------

                 type:  numeric (byte)

                range:  [.,.]                        units:  .
        unique values:  0                        missing .:  3963/4168
      unique mv codes:  2                       missing .*:  205/4168

           tabulation:  Freq.  Value
                         3963  .
                          205  .j

Ref3_ServDate3_sys:
  1.  some missing due to correcting date service received, but not knowing time service
      received

-----------------------------------------------------------------------------------------------
Ref3_ServCameFor4                                            Referral 3: Service 4 Came For
-----------------------------------------------------------------------------------------------

                 type:  numeric (int)
                label:  Service

                range:  [1000,1000]                  units:  1
        unique values:  1                        missing .:  3961/4168
```

```
      unique mv codes:  2                       missing .*:  205/4168

          tabulation:  Freq.   Numeric  Label
                           2      1000  1000. NO ADDITIONAL SERVICE
                        3961         .
                         205        .j  .j. Ineligible: External site
                                        client

-------------------------------------------------------------------------------------------------
Ref3_ServRecv4                                              Referral 3: Service 4 Received
-------------------------------------------------------------------------------------------------

                type:  numeric (int)
               label:  Service

               range:  [1000,1000]                  units:  1000
       unique values:  1                        missing .:  3962/4168
     unique mv codes:  2                       missing .*:  205/4168

          tabulation:  Freq.   Numeric  Label
                           1      1000  1000. NO ADDITIONAL SERVICE
                        3962         .
                         205        .j  .j. Ineligible: External site
                                        client

-------------------------------------------------------------------------------------------------
Ref3_DateServRec4                                      Referral 3: Date Service 4 Received
-------------------------------------------------------------------------------------------------

                type:  numeric daily date (int)

               range:  [19806,19806]                units:  1
     or equivalently:  [24mar2014,24mar2014]        units:  days
       unique values:  1                        missing .:  3962/4168
     unique mv codes:  2                       missing .*:  205/4168

          tabulation:  Freq.  Value
                           1  19806  24mar2014
                        3962  .              .
                         205  .j            .j

-------------------------------------------------------------------------------------------------
Ref3_ServDate4_sys                              Referral 3: System Date Service 4 Received
-------------------------------------------------------------------------------------------------

                type:  numeric (byte)

               range:  [.,.]                        units:  .
       unique values:  0                        missing .:  3963/4168
     unique mv codes:  2                       missing .*:  205/4168

          tabulation:  Freq.  Value
                        3963  .
                         205  .j

Ref3_ServDate4_sys:
  1.  some missing due to correcting date service received, but not knowing time service
      received

-------------------------------------------------------------------------------------------------
Ref3_ServCameFor5                                           Referral 3: Service 5 Came For
-------------------------------------------------------------------------------------------------

                type:  numeric (int)
               label:  Service

               range:  [1000,1000]                  units:  1
       unique values:  1                        missing .:  3961/4168
     unique mv codes:  2                       missing .*:  205/4168
```

```
           tabulation:  Freq.   Numeric  Label
                            2      1000  1000. NO ADDITIONAL SERVICE
                         3961         .
                          205        .j  .j. Ineligible: External site
                                         client

-------------------------------------------------------------------------------------------
Ref3_ServRecv5                                              Referral 3: Service 5 Received
-------------------------------------------------------------------------------------------

                 type:  numeric (int)
                label:  Service

                range:  [1000,1000]                  units:  1000
        unique values:  1                        missing .:  3962/4168
      unique mv codes:  2                       missing .*:  205/4168

           tabulation:  Freq.   Numeric  Label
                            1      1000  1000. NO ADDITIONAL SERVICE
                         3962         .
                          205        .j  .j. Ineligible: External site
                                         client

-------------------------------------------------------------------------------------------
Ref3_DateServRec5                                      Referral 3: Date Service 5 Received
-------------------------------------------------------------------------------------------

                 type:  numeric daily date (int)

                range:  [19806,19806]                units:  1
      or equivalently:  [24mar2014,24mar2014]        units:  days
        unique values:  1                        missing .:  3962/4168
      unique mv codes:  2                       missing .*:  205/4168

           tabulation:  Freq.  Value
                            1  19806  24mar2014
                         3962  .              .
                          205  .j            .j

-------------------------------------------------------------------------------------------
Ref3_ServDate5_sys                              Referral 3: System Date Service 5 Received
-------------------------------------------------------------------------------------------

                 type:  numeric (byte)

                range:  [.,.]                        units:  .
        unique values:  0                        missing .:  3963/4168
      unique mv codes:  2                       missing .*:  205/4168

           tabulation:  Freq.  Value
                         3963  .
                          205  .j

Ref3_ServDate5_sys:
  1.  some missing due to correcting date service received, but not knowing time service
      received

-------------------------------------------------------------------------------------------
Ref4_ID                                                                      Referral 4 ID
-------------------------------------------------------------------------------------------

                 type:  numeric (long)

                range:  [282,99195]                  units:  1
        unique values:  123                      missing .:  3840/4168
      unique mv codes:  2                       missing .*:  205/4168

                 mean:   94656.2
             std. dev:   14769.5
```

```
          percentiles:        10%       25%       50%       75%       90%
                            99002     99037     99087     99145     99171

Ref4_ID:
  1.  if begins with 99, referral created for analysis

-----------------------------------------------------------------------------------------------
Ref4_Source_an                                                              Referral 4 Source
-----------------------------------------------------------------------------------------------

                  type:  numeric (byte)
                 label:  RefSource

                 range:  [1,2]                        units:  1
         unique values:  2                        missing .:  3840/4168
       unique mv codes:  2                       missing .*:  205/4168

            tabulation:  Freq.   Numeric  Label
                           121         1  1. Registration
                             2         2  2. Other Referral
                          3840         .
                           205        .j  .j. Ineligible: External site
                                          client

Ref4_Source_an:
  1.  created for analysis

-----------------------------------------------------------------------------------------------
Ref4_ParentID                                           Referral 4 Parent ID: RegID or RefID
-----------------------------------------------------------------------------------------------

                  type:  numeric (long)

                 range:  [273,71369]                  units:  1
         unique values:  123                      missing .:  3840/4168
       unique mv codes:  2                       missing .*:  205/4168

                  mean:   54143.4
             std. dev:    12050.4

          percentiles:        10%       25%       50%       75%       90%
                            51077     51281     51602     56545     70055

-----------------------------------------------------------------------------------------------
Ref4_Self_an                                                      Referral 4 for Respondent
-----------------------------------------------------------------------------------------------

                  type:  numeric (byte)
                 label:  yesno

                 range:  [0,1]                        units:  1
         unique values:  2                        missing .:  3840/4168
       unique mv codes:  2                       missing .*:  205/4168

            tabulation:  Freq.   Numeric  Label
                           117         0  0. No
                             6         1  1. Yes
                          3840         .
                           205        .j  .j. Ineligible: External site
                                          client

Ref4_Self_an:
  1.  created for analysis

-----------------------------------------------------------------------------------------------
Ref4_Partner_an                                       Referral 4 for Respondent's Partner
-----------------------------------------------------------------------------------------------

                  type:  numeric (byte)
                 label:  yesno
```

```
            range:  [0,1]                        units:  1
    unique values:  2                        missing .:  3840/4168
  unique mv codes:  2                       missing .*:  205/4168

       tabulation:  Freq.   Numeric  Label
                       18         0  0. No
                      105         1  1. Yes
                     3840         .
                      205        .j  .j. Ineligible: External site
                                     client

Ref4_Partner_an:
  1.  created for analysis

-----------------------------------------------------------------------------------------------
Ref4_Other_an                                                              Referral 4 for Other
-----------------------------------------------------------------------------------------------

             type:  numeric (byte)
            label:  yesno

            range:  [0,1]                        units:  1
    unique values:  2                        missing .:  3840/4168
  unique mv codes:  2                       missing .*:  205/4168

       tabulation:  Freq.   Numeric  Label
                      111         0  0. No
                       12         1  1. Yes
                     3840         .
                      205        .j  .j. Ineligible: External site
                                     client

Ref4_Other_an:
  1.  created for analysis

-----------------------------------------------------------------------------------------------
Ref4_PersID                            Referral 4 Given To: Respondent or Person Accompanying
-----------------------------------------------------------------------------------------------

             type:  numeric (byte)
            label:  RefPersID

            range:  [0,0]                        units:  1
    unique values:  1                        missing .:  3840/4168
  unique mv codes:  2                       missing .*:  205/4168

       tabulation:  Freq.   Numeric  Label
                      123         0  0. Respondent
                     3840         .
                      205        .j  .j. Ineligible: External site
                                     client

-----------------------------------------------------------------------------------------------
Ref4_Date                                                              Referral 4: Date Given
-----------------------------------------------------------------------------------------------

             type:  numeric daily date (int)

            range:  [19715,19928]                units:  1
 or equivalently:  [23dec2013,24jul2014]        units:  days
    unique values:  67                       missing .:  3840/4168
  unique mv codes:  2                       missing .*:  205/4168

             mean:   19817.7 = 04apr2014 (+ 17 hours)
         std. dev:   44.7929

      percentiles:        10%       25%       50%       75%       90%
                        19766     19787     19810     19851     19885
                    12feb2014 05mar2014 28mar2014 08may2014 11jun2014
```

```
-------------------------------------------------------------------------------------------
Ref4_ReferralFOR                               Referral 4 For: Relationship to RefPersID_Ref4
-------------------------------------------------------------------------------------------

                  type:  numeric (byte)
                 label:  relation

                 range:  [0,12]                       units:  1
         unique values:  4                        missing .:  3840/4168
       unique mv codes:  2                       missing .*:  205/4168

            tabulation:  Freq.   Numeric  Label
                             6         0  0. SELF
                           104         1  1. SPOUSE
                             1         2  2. COHABITATING PARTNER
                            12        12  12. OTHER NON-RELATIVE
                          3840         .
                           205        .j  .j. Ineligible: External site
                                          client

-------------------------------------------------------------------------------------------
Ref4_FromSite                                                         Referral 4 from Site
-------------------------------------------------------------------------------------------

                  type:  numeric (byte)
                 label:  site

                 range:  [2,11]                       units:  1
         unique values:  5                        missing .:  3840/4168
       unique mv codes:  2                       missing .*:  205/4168

            tabulation:  Freq.   Numeric  Label
                            87         2  2. Chawama Clinic - MCH
                             1         4  4. Chawama Clinic - TB, STI &
                                          HIV Clinic
                            30         6  6. Kapata Urban Clinic, MCH
                             2         8  8. Chipata Gen Hosp - OP VMMC
                             3        11  11. SFH New Start
                          3840         .
                           205        .j  .j. Ineligible: External site
                                          client

-------------------------------------------------------------------------------------------
Ref4_ToSite                                                             Referral 4 to Site
-------------------------------------------------------------------------------------------

                  type:  numeric (byte)
                 label:  site

                 range:  [3,13]                       units:  1
         unique values:  7                        missing .:  3840/4168
       unique mv codes:  2                       missing .*:  205/4168

            tabulation:  Freq.   Numeric  Label
                            84         3  3. Chawama Clinic - Out Patient
                                          Ward (VMMC)
                             4         4  4. Chawama Clinic - TB, STI &
                                          HIV Clinic
                             8         7  7. Kapata Urban Clinic, TB, STI
                                          & HIV
                             1         9  9. Chipata Gen Hosp - MCH
                             7        10  10. Chipata Gen Hosp - TB, STI &
                                          HIV
                            18        11  11. SFH New Start
                             1        13  13. SFH VMMC
                          3840         .
                           205        .j  .j. Ineligible: External site
                                          client
```

```
-------------------------------------------------------------------------------------------
Ref4_ServiceFor                                                        Referral 4 for Service
-------------------------------------------------------------------------------------------

                  type:  numeric (byte)
                 label:  Service

                 range:  [1,15]                        units:  1
         unique values:  6                         missing .:  3840/4168
       unique mv codes:  2                        missing .*:  205/4168

            tabulation:  Freq.   Numeric  Label
                             1         1  1. CCS: CERVICAL CANCER
                                          SCREENING
                           112         6  6. HIV: HTC - COUPLES
                             2         7  7. HIV: HTC - INDIVIDUAL
                             4         9  9. HIV: CD4 TESTING
                             1        12  12. STI: ASSESSMENT
                             3        15  15. VMMC: PROCEDURE
                          3840         .
                           205        .j  .j. Ineligible: External site
                                          client

Ref4_ServiceFor:
  1.  Those who received referrals for couples HTC were coded in a specific manner based on
      the services they received prior.  - If a respondent had received individual HTC, VMMC
      counseling or VMMC procedure prior to receiving the referral for couples HTC, s/he
      received one referral for couples HTC for his/her spouse or partner.  - If a respondent
      did not receive individual HTC, VMMC counseling or VMMC procedure prior to receiving
      his/her referral for couples HTC, s/he received two referrals for couples HTC: one for
      self and one for spouse or partner.

-------------------------------------------------------------------------------------------
Ref4_ShowDate                                      Referral 4: Date Return to Act On Referral
-------------------------------------------------------------------------------------------

                  type:  numeric daily date (byte)

                 range:  [.,.]                         units:  .
       or equivalently:  [.,.]                         units:  days
         unique values:  0                         missing .:  3963/4168
       unique mv codes:  2                        missing .*:  205/4168

            tabulation:  Freq.  Value
                          3963  .              .
                           205  .j            .j

-------------------------------------------------------------------------------------------
Ref4_ShowHMM                                       Referral 4: Time Return to Act On Referral
-------------------------------------------------------------------------------------------

                  type:  string (str1), but longest is str0

         unique values:  0                         missing "":  4168/4168

            tabulation:  Freq.  Value
                          4168  ""

-------------------------------------------------------------------------------------------
Ref4_ServDate                                              Referral 4: Date Services Received
-------------------------------------------------------------------------------------------

                  type:  numeric daily date (byte)

                 range:  [.,.]                         units:  .
       or equivalently:  [.,.]                         units:  days
         unique values:  0                         missing .:  3963/4168
       unique mv codes:  2                        missing .*:  205/4168

            tabulation:  Freq.  Value
```

```
                      3963  .              .
                       205  .j            .j

-----------------------------------------------------------------------------------------------
Ref4_ServHMM                                                 Referral 4: Time Services Received
-----------------------------------------------------------------------------------------------

                  type:  string (str1), but longest is str0

         unique values:  0                        missing "":  4168/4168

            tabulation:  Freq.  Value
                          4168  ""

Ref4_ServHMM:
  1.  some missing due to correcting date service received, but not knowing time service
      received

-----------------------------------------------------------------------------------------------
Ref4_Counseling                                                 Referral 4: Counseling Received
-----------------------------------------------------------------------------------------------

                  type:  numeric (byte)
                 label:  Counseling

                 range:  [.,.]                        units:  .
         unique values:  0                        missing .:  3963/4168
       unique mv codes:  2                       missing .*:  205/4168

            tabulation:  Freq.   Numeric  Label
                          3963         .
                           205        .j  .j. Ineligible: External site
                                          client

-----------------------------------------------------------------------------------------------
Ref4_ReceivedDayReturned                          Referral 4: Services Received Same Day Returned
-----------------------------------------------------------------------------------------------

                  type:  numeric (byte)
                 label:  yesno

                 range:  [.,.]                        units:  .
         unique values:  0                        missing .:  3963/4168
       unique mv codes:  2                       missing .*:  205/4168

            tabulation:  Freq.   Numeric  Label
                          3963         .
                           205        .j  .j. Ineligible: External site
                                          client

Ref4_ReceivedDayReturned:
  1.  Yes if Date showed up for services = Date services received
      (Ref1_ShowDate=Ref1_ServDate)

-----------------------------------------------------------------------------------------------
Ref4_Escort                                                        Referral 4: Escort Provided
-----------------------------------------------------------------------------------------------

                  type:  numeric (byte)
                 label:  Ref4_Escort

                 range:  [.,.]                        units:  .
         unique values:  0                        missing .:  0/4168
       unique mv codes:  4                       missing .*:  4168/4168

            tabulation:  Freq.   Numeric  Label
                          2642        .a  .a. Not in integrated arm
                          1264        .b  .b. No referral
                            57        .d  .d. Have not yet receieved
                                          services from referral
```

```
                        205        .j  .j. Ineligible: External site
                                       client

-----------------------------------------------------------------------------------------------
Ref4_EscortTime                                                Referral 4: Time Escort Provided
-----------------------------------------------------------------------------------------------

                  type:  string (str1), but longest is str0

         unique values:  0                        missing "":  4168/4168

            tabulation:  Freq.  Value
                          4168  ""

-----------------------------------------------------------------------------------------------
Ref4_VoucherNumber                                                    Referral 4 Voucher Number
-----------------------------------------------------------------------------------------------

                  type:  string (str10)

         unique values:  123                      missing "":  4045/4168

              examples:  ""
                         ""
                         ""
                         ""

Ref4_VoucherNumber:
  1.  if begins with 99, referral created for analysis

-----------------------------------------------------------------------------------------------
Ref4_ServCameFor1                                                Referral 4: Service 1 Came For
-----------------------------------------------------------------------------------------------

                  type:  numeric (byte)
                 label:  Service

                 range:  [.,.]                        units:  .
         unique values:  0                        missing .:  3963/4168
       unique mv codes:  2                       missing .*:  205/4168

            tabulation:  Freq.   Numeric  Label
                          3963         .
                           205        .j  .j. Ineligible: External site
                                          client

-----------------------------------------------------------------------------------------------
Ref4_ServRecv1                                                   Referral 4: Service 1 Received
-----------------------------------------------------------------------------------------------

                  type:  numeric (byte)
                 label:  Service

                 range:  [.,.]                        units:  .
         unique values:  0                        missing .:  3963/4168
       unique mv codes:  2                       missing .*:  205/4168

            tabulation:  Freq.   Numeric  Label
                          3963         .
                           205        .j  .j. Ineligible: External site
                                          client

-----------------------------------------------------------------------------------------------
Ref4_DateServRec1                                           Referral 4: Date Service 1 Received
-----------------------------------------------------------------------------------------------

                  type:  numeric daily date (byte)

                 range:  [.,.]                        units:  .
       or equivalently:  [.,.]                        units:  days
```

```
        unique values:  0                          missing .:  3963/4168
      unique mv codes:  2                         missing .*:  205/4168

          tabulation:  Freq.  Value
                        3963  .               .
                         205  .j             .j
```

```
-------------------------------------------------------------------------------------------------
Ref4_ServDate1_sys                                  Referral 4: System Date Service 1 Received
-------------------------------------------------------------------------------------------------

                type:  numeric (byte)

               range:  [.,.]                        units:  .
        unique values:  0                          missing .:  3963/4168
      unique mv codes:  2                         missing .*:  205/4168

          tabulation:  Freq.  Value
                        3963  .
                         205  .j

Ref4_ServDate1_sys:
  1.  some missing due to correcting date service received, but not knowing time service
      received
```

```
-------------------------------------------------------------------------------------------------
Ref4_ServCameFor2                                              Referral 4: Service 2 Came For
-------------------------------------------------------------------------------------------------

                type:  numeric (byte)
               label:  Service

               range:  [.,.]                        units:  .
        unique values:  0                          missing .:  3963/4168
      unique mv codes:  2                         missing .*:  205/4168

          tabulation:  Freq.   Numeric  Label
                        3963         .
                         205        .j  .j. Ineligible: External site
                                        client
```

```
-------------------------------------------------------------------------------------------------
Ref4_ServRecv2                                                 Referral 4: Service 2 Received
-------------------------------------------------------------------------------------------------

                type:  numeric (byte)
               label:  Service

               range:  [.,.]                        units:  .
        unique values:  0                          missing .:  3963/4168
      unique mv codes:  2                         missing .*:  205/4168

          tabulation:  Freq.   Numeric  Label
                        3963         .
                         205        .j  .j. Ineligible: External site
                                        client
```

```
-------------------------------------------------------------------------------------------------
Ref4_DateServRec2                                         Referral 4: Date Service 2 Received
-------------------------------------------------------------------------------------------------

                type:  numeric daily date (byte)

               range:  [.,.]                        units:  .
      or equivalently:  [.,.]                        units:  days
        unique values:  0                          missing .:  3963/4168
      unique mv codes:  2                         missing .*:  205/4168

          tabulation:  Freq.  Value
                        3963  .               .
```

```
                          205  .j            .j

-----------------------------------------------------------------------------------------------
Ref4_ServDate2_sys                                  Referral 4: System Date Service 2 Received
-----------------------------------------------------------------------------------------------

                  type:  numeric (byte)

                 range:  [.,.]                         units:  .
         unique values:  0                         missing .:  3963/4168
       unique mv codes:  2                        missing .*:  205/4168

            tabulation:  Freq.  Value
                          3963  .
                           205  .j

Ref4_ServDate2_sys:
  1.  some missing due to correcting date service received, but not knowing time service
      received

-----------------------------------------------------------------------------------------------
Ref4_ServCameFor3                                               Referral 4: Service 3 Came For
-----------------------------------------------------------------------------------------------

                  type:  numeric (byte)
                 label:  Service

                 range:  [.,.]                         units:  .
         unique values:  0                         missing .:  3963/4168
       unique mv codes:  2                        missing .*:  205/4168

            tabulation:  Freq.   Numeric  Label
                          3963         .
                           205        .j  .j. Ineligible: External site
                                          client

-----------------------------------------------------------------------------------------------
Ref4_ServRecv3                                                  Referral 4: Service 3 Received
-----------------------------------------------------------------------------------------------

                  type:  numeric (byte)
                 label:  Service

                 range:  [.,.]                         units:  .
         unique values:  0                         missing .:  3963/4168
       unique mv codes:  2                        missing .*:  205/4168

            tabulation:  Freq.   Numeric  Label
                          3963         .
                           205        .j  .j. Ineligible: External site
                                          client

-----------------------------------------------------------------------------------------------
Ref4_DateServRec3                                          Referral 4: Date Service 3 Received
-----------------------------------------------------------------------------------------------

                  type:  numeric daily date (byte)

                 range:  [.,.]                         units:  .
     or equivalently:  [.,.]                         units:  days
         unique values:  0                         missing .:  3963/4168
       unique mv codes:  2                        missing .*:  205/4168

            tabulation:  Freq.  Value
                          3963  .             .
                           205  .j            .j

-----------------------------------------------------------------------------------------------
Ref4_ServDate3_sys                                  Referral 4: System Date Service 3 Received
-----------------------------------------------------------------------------------------------
```

```
                  type:  numeric (byte)

                 range:  [.,.]                        units:  .
         unique values:  0                        missing .:  3963/4168
       unique mv codes:  2                       missing .*:  205/4168

            tabulation:  Freq.  Value
                          3963  .
                           205  .j

Ref4_ServDate3_sys:
  1.  some missing due to correcting date service received, but not knowing time service
      received

-----------------------------------------------------------------------------------------------
Ref4_ServCameFor4                                          Referral 4: Service 4 Came For
-----------------------------------------------------------------------------------------------

                  type:  numeric (byte)
                 label:  Service

                 range:  [.,.]                        units:  .
         unique values:  0                        missing .:  3963/4168
       unique mv codes:  2                       missing .*:  205/4168

            tabulation:  Freq.   Numeric  Label
                          3963         .
                           205        .j  .j. Ineligible: External site
                                          client

-----------------------------------------------------------------------------------------------
Ref4_ServRecv4                                             Referral 4: Service 4 Received
-----------------------------------------------------------------------------------------------

                  type:  numeric (byte)
                 label:  Service

                 range:  [.,.]                        units:  .
         unique values:  0                        missing .:  3963/4168
       unique mv codes:  2                       missing .*:  205/4168

            tabulation:  Freq.   Numeric  Label
                          3963         .
                           205        .j  .j. Ineligible: External site
                                          client

-----------------------------------------------------------------------------------------------
Ref4_DateServRec4                                     Referral 4: Date Service 4 Received
-----------------------------------------------------------------------------------------------

                  type:  numeric daily date (byte)

                 range:  [.,.]                        units:  .
       or equivalently:  [.,.]                        units:  days
         unique values:  0                        missing .:  3963/4168
       unique mv codes:  2                       missing .*:  205/4168

            tabulation:  Freq.  Value
                          3963  .              .
                           205  .j            .j

-----------------------------------------------------------------------------------------------
Ref4_ServDate4_sys                             Referral 4: System Date Service 4 Received
-----------------------------------------------------------------------------------------------

                  type:  numeric (byte)

                 range:  [.,.]                        units:  .
         unique values:  0                        missing .:  3963/4168
```

```
       unique mv codes:  2                       missing .*:  205/4168

            tabulation:  Freq.  Value
                          3963  .
                           205  .j
```

Ref4_ServDate4_sys:
1. some missing due to correcting date service received, but not knowing time service received

```
-----------------------------------------------------------------------------------------------
Ref4_ServCameFor5                                              Referral 4: Service 5 Came For
-----------------------------------------------------------------------------------------------

                  type:  numeric (byte)
                 label:  Service

                 range:  [.,.]                        units:  .
         unique values:  0                        missing .:  3963/4168
       unique mv codes:  2                       missing .*:  205/4168

            tabulation:  Freq.   Numeric  Label
                          3963         .
                           205        .j  .j. Ineligible: External site
                                          client

-----------------------------------------------------------------------------------------------
Ref4_ServRecv5                                                 Referral 4: Service 5 Received
-----------------------------------------------------------------------------------------------

                  type:  numeric (byte)
                 label:  Service

                 range:  [.,.]                        units:  .
         unique values:  0                        missing .:  3963/4168
       unique mv codes:  2                       missing .*:  205/4168

            tabulation:  Freq.   Numeric  Label
                          3963         .
                           205        .j  .j. Ineligible: External site
                                          client

-----------------------------------------------------------------------------------------------
Ref4_DateServRec5                                         Referral 4: Date Service 5 Received
-----------------------------------------------------------------------------------------------

                  type:  numeric daily date (byte)

                 range:  [.,.]                        units:  .
       or equivalently:  [.,.]                        units:  days
         unique values:  0                        missing .:  3963/4168
       unique mv codes:  2                       missing .*:  205/4168

            tabulation:  Freq.  Value
                          3963  .             .
                           205  .j           .j

-----------------------------------------------------------------------------------------------
Ref4_ServDate5_sys                                 Referral 4: System Date Service 5 Received
-----------------------------------------------------------------------------------------------

                  type:  numeric (byte)

                 range:  [.,.]                        units:  .
         unique values:  0                        missing .:  3963/4168
       unique mv codes:  2                       missing .*:  205/4168

            tabulation:  Freq.  Value
                          3963  .
                           205  .j
```

Ref4_ServDate5_sys:
  1. some missing due to correcting date service received, but not knowing time service
      received

```
-----------------------------------------------------------------------------------------------
Ref5_ID                                                                          Referral 5 ID
-----------------------------------------------------------------------------------------------

                  type:  numeric (long)

                 range:  [99014,99066]                units:  1
         unique values:  2                        missing .:  3961/4168
       unique mv codes:  2                       missing .*:  205/4168

            tabulation:  Freq.  Value
                             1  99014
                             1  99066
                          3961  .
                           205  .j
```

Ref5_ID:
  1. if begins with 99, referral created for analysis

```
-----------------------------------------------------------------------------------------------
Ref5_Source_an                                                               Referral 5 Source
-----------------------------------------------------------------------------------------------

                  type:  numeric (byte)
                 label:  RefSource

                 range:  [1,1]                        units:  1
         unique values:  1                        missing .:  3961/4168
       unique mv codes:  2                       missing .*:  205/4168

            tabulation:  Freq.   Numeric  Label
                             2         1  1. Registration
                          3961         .
                           205        .j  .j. Ineligible: External site
                                          client
```

Ref5_Source_an:
  1. created for analysis

```
-----------------------------------------------------------------------------------------------
Ref5_ParentID                                              Referral 5 Parent ID: RegID or RefID
-----------------------------------------------------------------------------------------------

                  type:  numeric (long)

                 range:  [51050,51441]                units:  1
         unique values:  2                        missing .:  3961/4168
       unique mv codes:  2                       missing .*:  205/4168

            tabulation:  Freq.  Value
                             1  51050
                             1  51441
                          3961  .
                           205  .j

-----------------------------------------------------------------------------------------------
Ref5_Self_an                                                          Referral 5 for Respondent
-----------------------------------------------------------------------------------------------

                  type:  numeric (byte)
                 label:  yesno

                 range:  [0,0]                        units:  1
         unique values:  1                        missing .:  3961/4168
       unique mv codes:  2                       missing .*:  205/4168
```

```
           tabulation:  Freq.   Numeric  Label
                            2         0  0. No
                         3961         .
                          205        .j  .j. Ineligible: External site
                                         client
```

Ref5_Self_an:
  1. created for analysis

```
-----------------------------------------------------------------------------------------------
Ref5_Partner_an                                            Referral 5 for Respondent's Partner
-----------------------------------------------------------------------------------------------

                 type:  numeric (byte)
                label:  yesno

                range:  [1,1]                        units:  1
        unique values:  1                        missing .:  3961/4168
      unique mv codes:  2                       missing .*:  205/4168

           tabulation:  Freq.   Numeric  Label
                            2         1  1. Yes
                         3961         .
                          205        .j  .j. Ineligible: External site
                                         client
```

Ref5_Partner_an:
  1. created for analysis

```
-----------------------------------------------------------------------------------------------
Ref5_Other_an                                                              Referral 5 for Other
-----------------------------------------------------------------------------------------------

                 type:  numeric (byte)
                label:  yesno

                range:  [0,0]                        units:  1
        unique values:  1                        missing .:  3961/4168
      unique mv codes:  2                       missing .*:  205/4168

           tabulation:  Freq.   Numeric  Label
                            2         0  0. No
                         3961         .
                          205        .j  .j. Ineligible: External site
                                         client
```

Ref5_Other_an:
  1. created for analysis

```
-----------------------------------------------------------------------------------------------
Ref5_PersID                                Referral 5 Given To: Respondent or Person Accompanying
-----------------------------------------------------------------------------------------------

                 type:  numeric (byte)
                label:  RefPersID

                range:  [0,0]                        units:  1
        unique values:  1                        missing .:  3961/4168
      unique mv codes:  2                       missing .*:  205/4168

           tabulation:  Freq.   Numeric  Label
                            2         0  0. Respondent
                         3961         .
                          205        .j  .j. Ineligible: External site
                                         client

-----------------------------------------------------------------------------------------------
Ref5_Date                                                                Referral 5: Date Given
-----------------------------------------------------------------------------------------------
```

```
                 type:  numeric daily date (int)

                range:  [19761,19808]                units:  1
       or equivalently:  [07feb2014,26mar2014]        units:  days
         unique values:  2                        missing .:  3961/4168
       unique mv codes:  2                       missing .*:  205/4168

            tabulation:  Freq.  Value
                             1  19761  07feb2014
                             1  19808  26mar2014
                          3961  .             .
                           205  .j           .j

-------------------------------------------------------------------------------
Ref5_ReferralFOR                Referral 5 For: Relationship to RefPersID_Ref5
-------------------------------------------------------------------------------

                  type:  numeric (byte)
                 label:  relation

                 range:  [1,1]                        units:  1
         unique values:  1                        missing .:  3961/4168
       unique mv codes:  2                       missing .*:  205/4168

            tabulation:  Freq.   Numeric  Label
                             2         1  1. SPOUSE
                          3961         .
                           205        .j  .j. Ineligible: External site
                                          client

-------------------------------------------------------------------------------
Ref5_FromSite                                             Referral 5 from Site
-------------------------------------------------------------------------------

                  type:  numeric (byte)
                 label:  site

                 range:  [2,2]                        units:  1
         unique values:  1                        missing .:  3961/4168
       unique mv codes:  2                       missing .*:  205/4168

            tabulation:  Freq.   Numeric  Label
                             2         2  2. Chawama Clinic - MCH
                          3961         .
                           205        .j  .j. Ineligible: External site
                                          client

-------------------------------------------------------------------------------
Ref5_ToSite                                                 Referral 5 to Site
-------------------------------------------------------------------------------

                  type:  numeric (byte)
                 label:  site

                 range:  [3,3]                        units:  1
         unique values:  1                        missing .:  3961/4168
       unique mv codes:  2                       missing .*:  205/4168

            tabulation:  Freq.   Numeric  Label
                             2         3  3. Chawama Clinic - Out Patient
                                          Ward (VMMC)
                          3961         .
                           205        .j  .j. Ineligible: External site
                                          client

-------------------------------------------------------------------------------
Ref5_ServiceFor                                         Referral 5 for Service
-------------------------------------------------------------------------------
```

```
                 type:  numeric (byte)
                label:  Service

                range:  [6,6]                        units:  1
        unique values:  1                        missing .:  3961/4168
      unique mv codes:  2                       missing .*:  205/4168

           tabulation:  Freq.   Numeric  Label
                            2         6  6. HIV: HTC - COUPLES
                         3961         .
                          205        .j  .j. Ineligible: External site
                                         client
```

Ref5_ServiceFor:
  1. Those who received referrals for couples HTC were coded in a specific manner based on the services they received prior. - If a respondent had received individual HTC, VMMC counseling or VMMC procedure prior to receiving the referral for couples HTC, s/he received one referral for couples HTC for his/her spouse or partner. - If a respondent did not receive individual HTC, VMMC counseling or VMMC procedure prior to receiving his/her referral for couples HTC, s/he received two referrals for couples HTC: one for self and one for spouse or partner.

```
-----------------------------------------------------------------------------------------------
Ref5_ShowDate                                       Referral 5: Date Return to Act On Referral
-----------------------------------------------------------------------------------------------

                 type:  numeric daily date (byte)

                range:  [.,.]                        units:  .
      or equivalently:  [.,.]                        units:  days
        unique values:  0                        missing .:  3963/4168
      unique mv codes:  2                       missing .*:  205/4168

           tabulation:  Freq.  Value
                         3963  .              .
                          205  .j            .j

-----------------------------------------------------------------------------------------------
Ref5_ShowHMM                                        Referral 5: Time Return to Act On Referral
-----------------------------------------------------------------------------------------------

                 type:  string (str1), but longest is str0

        unique values:  0                        missing "":  4168/4168

           tabulation:  Freq.  Value
                         4168  ""

-----------------------------------------------------------------------------------------------
Ref5_ServDate                                               Referral 5: Date Services Received
-----------------------------------------------------------------------------------------------

                 type:  numeric daily date (byte)

                range:  [.,.]                        units:  .
      or equivalently:  [.,.]                        units:  days
        unique values:  0                        missing .:  3963/4168
      unique mv codes:  2                       missing .*:  205/4168

           tabulation:  Freq.  Value
                         3963  .              .
                          205  .j            .j

-----------------------------------------------------------------------------------------------
Ref5_ServHMM                                                Referral 5: Time Services Received
-----------------------------------------------------------------------------------------------

                 type:  string (str1), but longest is str0

        unique values:  0                        missing "":  4168/4168
```

```
           tabulation:  Freq.  Value
                         4168  ""

Ref5_ServHMM:
  1.  some missing due to correcting date service received, but not knowing time service
      received

-------------------------------------------------------------------------------------------
Ref5_Counseling                                           Referral 5: Counseling Received
-------------------------------------------------------------------------------------------

                 type:  numeric (byte)
                label:  Counseling

                range:  [.,.]                        units:  .
        unique values:  0                        missing .:  3963/4168
      unique mv codes:  2                       missing .*:  205/4168

           tabulation:  Freq.   Numeric  Label
                         3963         .
                          205        .j  .j. Ineligible: External site
                                         client

-------------------------------------------------------------------------------------------
Ref5_ReceivedDayReturned                        Referral 5: Services Received Same Day Returned
-------------------------------------------------------------------------------------------

                 type:  numeric (byte)
                label:  yesno

                range:  [.,.]                        units:  .
        unique values:  0                        missing .:  3963/4168
      unique mv codes:  2                       missing .*:  205/4168

           tabulation:  Freq.   Numeric  Label
                         3963         .
                          205        .j  .j. Ineligible: External site
                                         client

Ref5_ReceivedDayReturned:
  1.  Yes if Date showed up for services = Date services received
      (Ref1_ShowDate=Ref1_ServDate)

-------------------------------------------------------------------------------------------
Ref5_Escort                                                   Referral 5: Escort Provided
-------------------------------------------------------------------------------------------

                 type:  numeric (byte)
                label:  Ref5_Escort

                range:  [.,.]                        units:  .
        unique values:  0                        missing .:  0/4168
      unique mv codes:  4                       missing .*:  4168/4168

           tabulation:  Freq.   Numeric  Label
                         2642        .a  .a. Not in integrated arm
                         1320        .b  .b. No referral
                            1        .d  .d. Have not yet receieved
                                         services from referral
                          205        .j  .j. Ineligible: External site
                                         client

-------------------------------------------------------------------------------------------
Ref5_EscortTime                                          Referral 5: Time Escort Provided
-------------------------------------------------------------------------------------------

                 type:  string (str1), but longest is str0

        unique values:  0                        missing "":  4168/4168
```

```
            tabulation:  Freq.  Value
                          4168  ""

-------------------------------------------------------------------------------------------
Ref5_VoucherNumber                                              Referral 5 Voucher Number
-------------------------------------------------------------------------------------------

                  type:  string (str10)

         unique values:  2                        missing "":  4166/4168

            tabulation:  Freq.  Value
                          4166  ""
                             1  "99-03-0099"
                             1  "99-03-0278"

Ref5_VoucherNumber:
  1.  if begins with 99, referral created for analysis

-------------------------------------------------------------------------------------------
Ref5_ServCameFor1                                          Referral 5: Service 1 Came For
-------------------------------------------------------------------------------------------

                  type:  numeric (byte)
                 label:  Service

                 range:  [.,.]                        units:  .
         unique values:  0                        missing .:  3963/4168
       unique mv codes:  2                       missing .*:  205/4168

            tabulation:  Freq.   Numeric  Label
                          3963         .
                           205        .j  .j. Ineligible: External site
                                          client

-------------------------------------------------------------------------------------------
Ref5_ServRecv1                                             Referral 5: Service 1 Received
-------------------------------------------------------------------------------------------

                  type:  numeric (byte)
                 label:  Service

                 range:  [.,.]                        units:  .
         unique values:  0                        missing .:  3963/4168
       unique mv codes:  2                       missing .*:  205/4168

            tabulation:  Freq.   Numeric  Label
                          3963         .
                           205        .j  .j. Ineligible: External site
                                          client

-------------------------------------------------------------------------------------------
Ref5_DateServRec1                                     Referral 5: Date Service 1 Received
-------------------------------------------------------------------------------------------

                  type:  numeric daily date (byte)

                 range:  [.,.]                        units:  .
       or equivalently:  [.,.]                        units:  days
         unique values:  0                        missing .:  3963/4168
       unique mv codes:  2                       missing .*:  205/4168

            tabulation:  Freq.  Value
                          3963  .             .
                           205  .j            .j

-------------------------------------------------------------------------------------------
Ref5_ServDate1_sys                              Referral 5: System Date Service 1 Received
-------------------------------------------------------------------------------------------
```

```
                  type:  numeric (byte)

                 range:  [.,.]                        units:  .
         unique values:  0                        missing .:  3963/4168
       unique mv codes:  2                       missing .*:  205/4168

            tabulation:  Freq.  Value
                          3963  .
                           205  .j

Ref5_ServDate1_sys:
  1.  some missing due to correcting date service received, but not knowing time service
      received

-----------------------------------------------------------------------------------------------
Ref5_ServCameFor2                                              Referral 5: Service 2 Came For
-----------------------------------------------------------------------------------------------

                  type:  numeric (byte)
                 label:  Service

                 range:  [.,.]                        units:  .
         unique values:  0                        missing .:  3963/4168
       unique mv codes:  2                       missing .*:  205/4168

            tabulation:  Freq.   Numeric  Label
                          3963         .
                           205        .j  .j. Ineligible: External site
                                          client

-----------------------------------------------------------------------------------------------
Ref5_ServRecv2                                                 Referral 5: Service 2 Received
-----------------------------------------------------------------------------------------------

                  type:  numeric (byte)
                 label:  Service

                 range:  [.,.]                        units:  .
         unique values:  0                        missing .:  3963/4168
       unique mv codes:  2                       missing .*:  205/4168

            tabulation:  Freq.   Numeric  Label
                          3963         .
                           205        .j  .j. Ineligible: External site
                                          client

-----------------------------------------------------------------------------------------------
Ref5_DateServRec2                                         Referral 5: Date Service 2 Received
-----------------------------------------------------------------------------------------------

                  type:  numeric daily date (byte)

                 range:  [.,.]                        units:  .
       or equivalently:  [.,.]                        units:  days
         unique values:  0                        missing .:  3963/4168
       unique mv codes:  2                       missing .*:  205/4168

            tabulation:  Freq.  Value
                          3963  .             .
                           205  .j           .j

-----------------------------------------------------------------------------------------------
Ref5_ServDate2_sys                                 Referral 5: System Date Service 2 Received
-----------------------------------------------------------------------------------------------

                  type:  numeric (byte)

                 range:  [.,.]                        units:  .
         unique values:  0                        missing .:  3963/4168
```

```
       unique mv codes:  2                        missing .*:  205/4168

            tabulation:  Freq.  Value
                          3963  .
                           205  .j

Ref5_ServDate2_sys:
  1.  some missing due to correcting date service received, but not knowing time service
      received

-------------------------------------------------------------------------------------------
Ref5_ServCameFor3                                          Referral 5: Service 3 Came For
-------------------------------------------------------------------------------------------

                  type:  numeric (byte)
                 label:  Service

                 range:  [.,.]                        units:  .
         unique values:  0                        missing .:  3963/4168
       unique mv codes:  2                        missing .*:  205/4168

            tabulation:  Freq.   Numeric  Label
                          3963         .
                           205        .j  .j. Ineligible: External site
                                          client

-------------------------------------------------------------------------------------------
Ref5_ServRecv3                                             Referral 5: Service 3 Received
-------------------------------------------------------------------------------------------

                  type:  numeric (byte)
                 label:  Service

                 range:  [.,.]                        units:  .
         unique values:  0                        missing .:  3963/4168
       unique mv codes:  2                        missing .*:  205/4168

            tabulation:  Freq.   Numeric  Label
                          3963         .
                           205        .j  .j. Ineligible: External site
                                          client

-------------------------------------------------------------------------------------------
Ref5_DateServRec3                                     Referral 5: Date Service 3 Received
-------------------------------------------------------------------------------------------

                  type:  numeric daily date (byte)

                 range:  [.,.]                        units:  .
       or equivalently:  [.,.]                        units:  days
         unique values:  0                        missing .:  3963/4168
       unique mv codes:  2                        missing .*:  205/4168

            tabulation:  Freq.  Value
                          3963  .              .
                           205  .j            .j

-------------------------------------------------------------------------------------------
Ref5_ServDate3_sys                             Referral 5: System Date Service 3 Received
-------------------------------------------------------------------------------------------

                  type:  numeric (byte)

                 range:  [.,.]                        units:  .
         unique values:  0                        missing .:  3963/4168
       unique mv codes:  2                        missing .*:  205/4168

            tabulation:  Freq.  Value
                          3963  .
                           205  .j
```

```
Ref5_ServDate3_sys:
  1.  some missing due to correcting date service received, but not knowing time service
      received

-----------------------------------------------------------------------------------------------
Ref5_ServCameFor4                                          Referral 5: Service 4 Came For
-----------------------------------------------------------------------------------------------

                  type:  numeric (byte)
                 label:  Service

                 range:  [.,.]                        units:  .
         unique values:  0                        missing .:  3963/4168
       unique mv codes:  2                       missing .*:  205/4168

            tabulation:  Freq.   Numeric  Label
                          3963         .
                           205        .j  .j. Ineligible: External site
                                          client

-----------------------------------------------------------------------------------------------
Ref5_ServRecv4                                             Referral 5: Service 4 Received
-----------------------------------------------------------------------------------------------

                  type:  numeric (byte)
                 label:  Service

                 range:  [.,.]                        units:  .
         unique values:  0                        missing .:  3963/4168
       unique mv codes:  2                       missing .*:  205/4168

            tabulation:  Freq.   Numeric  Label
                          3963         .
                           205        .j  .j. Ineligible: External site
                                          client

-----------------------------------------------------------------------------------------------
Ref5_DateServRec4                                     Referral 5: Date Service 4 Received
-----------------------------------------------------------------------------------------------

                  type:  numeric daily date (byte)

                 range:  [.,.]                        units:  .
       or equivalently:  [.,.]                        units:  days
         unique values:  0                        missing .:  3963/4168
       unique mv codes:  2                       missing .*:  205/4168

            tabulation:  Freq.  Value
                          3963  .             .
                           205  .j           .j

-----------------------------------------------------------------------------------------------
Ref5_ServDate4_sys                             Referral 5: System Date Service 4 Received
-----------------------------------------------------------------------------------------------

                  type:  numeric (byte)

                 range:  [.,.]                        units:  .
         unique values:  0                        missing .:  3963/4168
       unique mv codes:  2                       missing .*:  205/4168

            tabulation:  Freq.  Value
                          3963  .
                           205  .j

Ref5_ServDate4_sys:
  1.  some missing due to correcting date service received, but not knowing time service
      received
```

```
--------------------------------------------------------------------------------------------
Ref5_ServCameFor5                                           Referral 5: Service 5 Came For
--------------------------------------------------------------------------------------------

                  type:  numeric (byte)
                 label:  Service

                 range:  [.,.]                        units:  .
         unique values:  0                        missing .:  3963/4168
       unique mv codes:  2                       missing .*:  205/4168

            tabulation:  Freq.   Numeric  Label
                          3963         .
                           205        .j  .j. Ineligible: External site
                                          client

--------------------------------------------------------------------------------------------
Ref5_ServRecv5                                              Referral 5: Service 5 Received
--------------------------------------------------------------------------------------------

                  type:  numeric (byte)
                 label:  Service

                 range:  [.,.]                        units:  .
         unique values:  0                        missing .:  3963/4168
       unique mv codes:  2                       missing .*:  205/4168

            tabulation:  Freq.   Numeric  Label
                          3963         .
                           205        .j  .j. Ineligible: External site
                                          client

--------------------------------------------------------------------------------------------
Ref5_DateServRec5                                      Referral 5: Date Service 5 Received
--------------------------------------------------------------------------------------------

                  type:  numeric daily date (byte)

                 range:  [.,.]                        units:  .
       or equivalently:  [.,.]                        units:  days
         unique values:  0                        missing .:  3963/4168
       unique mv codes:  2                       missing .*:  205/4168

            tabulation:  Freq.  Value
                          3963  .             .
                           205  .j           .j

--------------------------------------------------------------------------------------------
Ref5_ServDate5_sys                              Referral 5: System Date Service 5 Received
--------------------------------------------------------------------------------------------

                  type:  numeric (byte)

                 range:  [.,.]                        units:  .
         unique values:  0                        missing .:  3963/4168
       unique mv codes:  2                       missing .*:  205/4168

            tabulation:  Freq.  Value
                          3963  .
                           205  .j

Ref5_ServDate5_sys:
  1.  some missing due to correcting date service received, but not knowing time service
      received

--------------------------------------------------------------------------------------------
soc_MedRecord                                                  Medical record availability
--------------------------------------------------------------------------------------------

                  type:  numeric (byte)
```

```
                label:  MedRecord

                range:  [0,1]                        units:  1
        unique values:  2                        missing .:  362/4168
      unique mv codes:  3                       missing .*:  2849/4168

           tabulation:  Freq.   Numeric  Label
                          107         0  0. Medical Record Not
                                         Located/Not Available
                          850         1  1. Medical Record Found
                          362         .
                          205        .j  .j. Ineligible: External site
                                         client
                         2644        .l  .l. Ineligible: Experimental Arm

-----------------------------------------------------------------------------------------------
soc_fRefTotal                                         Number of referrals in facility records
-----------------------------------------------------------------------------------------------

                 type:  numeric (byte)

                range:  [0,1]                        units:  1
        unique values:  2                        missing .:  362/4168
      unique mv codes:  3                       missing .*:  2849/4168

           tabulation:  Freq.  Value
                          862  0
                           95  1
                          362  .
                          205  .j
                         2644  .l

-----------------------------------------------------------------------------------------------
soc_fRefForm                                                                           FRefForm
-----------------------------------------------------------------------------------------------

                 type:  numeric (byte)
                label:  fRefForm, but 2 nonmissing values are not labeled

                range:  [0,6]                        units:  1
        unique values:  4                        missing .:  1091/4168
      unique mv codes:  3                       missing .*:  2849/4168

           tabulation:  Freq.   Numeric  Label
                           47         0
                           78         1  1. Intake form
                          102         3  3. Register
                            1         6
                         1091         .
                          205        .j  .j. Ineligible: External site
                                         client
                         2644        .l  .l. Ineligible: Experimental Arm

-----------------------------------------------------------------------------------------------
soc_fRef1_ServiceFor                         Service referred for (report by facility records)
-----------------------------------------------------------------------------------------------

                 type:  numeric (byte)
                label:  fRef_ServiceFor

                range:  [4,15]                       units:  1
        unique values:  4                        missing .:  1272/4168
      unique mv codes:  3                       missing .*:  2849/4168

           tabulation:  Freq.   Numeric  Label
                            5         4  4. FP
                           35         8  8. ART Screening
                            1        12  12. STI screening
                            6        15  15. VMMC
                         1272         .
```

```
                     205        .j  .j. Ineligible: External site
                                        client
                    2644        .l  .l. Ineligible: Experimental Arm

-------------------------------------------------------------------------------------------
soc_fRef1_ToSite                         Service site referred to (report by facility records)
-------------------------------------------------------------------------------------------

                 type:  numeric (byte)
                label:  fRef_ToSite

                range:  [7,14]                       units:  1
        unique values:  3                        missing .:  1272/4168
      unique mv codes:  3                       missing .*:  2849/4168

           tabulation:  Freq.   Numeric  Label
                           14         7  7. Kapata Clinic TB, STI, HIV
                            1         8  8. Chipata Gen Hosp VMMC
                           32        14  14. Other/Not specific
                         1272         .
                          205        .j  .j. Ineligible: External site
                                         client
                         2644        .l  .l. Ineligible: Experimental Arm

-------------------------------------------------------------------------------------------
soc_fRef1_Serv                                     Service uptake (report by facility records)
-------------------------------------------------------------------------------------------

                 type:  numeric (byte)
                label:  fRef_Serv

                range:  [0,0]                        units:  1
        unique values:  1                        missing .:  1272/4168
      unique mv codes:  3                       missing .*:  2849/4168

           tabulation:  Freq.   Numeric  Label
                           47         0  0. No information on whether
                                         accessed service
                         1272         .
                          205        .j  .j. Ineligible: External site
                                         client
                         2644        .l  .l. Ineligible: Experimental Arm

-------------------------------------------------------------------------------------------
soc_fRef2_ServiceFor                          Service referred for (report by facility records)
-------------------------------------------------------------------------------------------

                 type:  numeric (byte)
                label:  fRef_ServiceFor

                range:  [4,12]                       units:  1
        unique values:  5                        missing .:  1287/4168
      unique mv codes:  3                       missing .*:  2849/4168

           tabulation:  Freq.   Numeric  Label
                            3         4  4. FP
                           24         8  8. ART Screening
                            1         9  9. CD4 Screening
                            1        10  10. Psychosocial counseling
                            3        12  12. STI screening
                         1287         .
                          205        .j  .j. Ineligible: External site
                                         client
                         2644        .l  .l. Ineligible: Experimental Arm

-------------------------------------------------------------------------------------------
soc_fRef2_ToSite                         Service site referred to (report by facility records)
-------------------------------------------------------------------------------------------

                 type:  numeric (byte)
```

```
              label:  fRef_ToSite

              range:  [7,14]                       units:  1
      unique values:  3                        missing .:  1287/4168
    unique mv codes:  3                       missing .*:  2849/4168

         tabulation:  Freq.   Numeric  Label
                         17         7  7. Kapata Clinic TB, STI, HIV
                          4         8  8. Chipata Gen Hosp VMMC
                         11        14  14. Other/Not specific
                       1287         .  
                        205        .j  .j. Ineligible: External site
                                       client
                       2644        .l  .l. Ineligible: Experimental Arm

-----------------------------------------------------------------------------------------------
soc_fRef2_Serv                                  Service uptake (report by facility records)
-----------------------------------------------------------------------------------------------

               type:  numeric (byte)
              label:  fRef_Serv

              range:  [0,0]                        units:  1
      unique values:  1                        missing .:  1287/4168
    unique mv codes:  3                       missing .*:  2849/4168

         tabulation:  Freq.   Numeric  Label
                         32         0  0. No information on whether
                                       accessed service
                       1287         .  
                        205        .j  .j. Ineligible: External site
                                       client
                       2644        .l  .l. Ineligible: Experimental Arm

-----------------------------------------------------------------------------------------------
soc_fRef3_ServiceFor                      Service referred for (report by facility records)
-----------------------------------------------------------------------------------------------

               type:  numeric (byte)
              label:  fRef_ServiceFor

              range:  [4,15]                       units:  1
      unique values:  3                        missing .:  1303/4168
    unique mv codes:  3                       missing .*:  2849/4168

         tabulation:  Freq.   Numeric  Label
                          1         4  4. FP
                         14         8  8. ART Screening
                          1        15  15. VMMC
                       1303         .  
                        205        .j  .j. Ineligible: External site
                                       client
                       2644        .l  .l. Ineligible: Experimental Arm

-----------------------------------------------------------------------------------------------
soc_fRef3_ToSite                      Service site referred to (report by facility records)
-----------------------------------------------------------------------------------------------

               type:  numeric (byte)
              label:  fRef_ToSite

              range:  [7,14]                       units:  1
      unique values:  2                        missing .:  1303/4168
    unique mv codes:  3                       missing .*:  2849/4168

         tabulation:  Freq.   Numeric  Label
                          8         7  7. Kapata Clinic TB, STI, HIV
                          8        14  14. Other/Not specific
                       1303         .  
                        205        .j  .j. Ineligible: External site
```

```
                                         client
                          2644        .l  .l. Ineligible: Experimental Arm

-------------------------------------------------------------------------------------------
soc_fRef3_Serv                                 Service uptake (report by facility records)
-------------------------------------------------------------------------------------------

                  type:  numeric (byte)
                 label:  fRef_Serv

                 range:  [0,0]                        units:  1
         unique values:  1                        missing .:  1303/4168
       unique mv codes:  3                       missing .*:  2849/4168

            tabulation:  Freq.   Numeric  Label
                            16         0  0. No information on whether
                                          accessed service
                          1303         .
                           205        .j  .j. Ineligible: External site
                                          client
                          2644        .l  .l. Ineligible: Experimental Arm

-------------------------------------------------------------------------------------------
soc_ClientContact                                                   Client contact attempt
-------------------------------------------------------------------------------------------

                  type:  numeric (byte)
                 label:  ClientContact

                 range:  [0,3]                        units:  1
         unique values:  4                        missing .:  205/4168
       unique mv codes:  3                       missing .*:  2849/4168

            tabulation:  Freq.   Numeric  Label
                             6         0  0. No contact needed/attempted
                           516         1  1. Client contacted by phone
                           492         2  2. Client contacted in person
                           100         3  3. Client could not be
                                          located/contacted
                           205         .
                           205        .j  .j. Ineligible: External site
                                          client
                          2644        .l  .l. Ineligible: Experimental Arm

-------------------------------------------------------------------------------------------
soc_cRefTotal                                       Number of referrals reported by client
-------------------------------------------------------------------------------------------

                  type:  numeric (byte)

                 range:  [0,3]                        units:  1
         unique values:  4                        missing .:  205/4168
       unique mv codes:  3                       missing .*:  2849/4168

            tabulation:  Freq.  Value
                           370  0
                           229  1
                           280  2
                           235  3
                           205  .
                           205  .j
                          2644  .l

-------------------------------------------------------------------------------------------
soc_cRef1_ServiceFor                               Service referred for (report by client)
-------------------------------------------------------------------------------------------

                  type:  numeric (byte)
                 label:  cRef_ServiceFor
```

```
           range:  [0,15]                     units:  1
   unique values:  9                      missing .:  576/4168
 unique mv codes:  3                     missing .*:  2849/4168

      tabulation:  Freq.   Numeric  Label
                       1         0  0. Other
                     219         1  1. Cervical Cancer Screening
                      45         4  4. FP
                      69         6  6. Couples HTC
                      90         7  7. Individual HTC
                       4         8  8. ART Screening
                      53         9  9. CD4 Screening
                     165        12  12. STI screening
                      97        15  15. VMMC
                     576         .
                     205        .j  .j. Ineligible: External site
                                    client
                    2644        .l  .l. Ineligible: Experimental Arm

-----------------------------------------------------------------------------------------------
soc_cRef1_ReferralFOR                                 For whom is referral (report by client)
-----------------------------------------------------------------------------------------------

            type:  numeric (byte)
           label:  cRef_ReferralFOR

           range:  [0,3]                      units:  1
   unique values:  4                      missing .:  576/4168
 unique mv codes:  3                     missing .*:  2849/4168

      tabulation:  Freq.   Numeric  Label
                     628         0  0. Self
                     111         1  1. Spouse
                       2         2  2. Child
                       2         3  3. Other
                     576         .
                     205        .j  .j. Ineligible: External site
                                    client
                    2644        .l  .l. Ineligible: Experimental Arm

-----------------------------------------------------------------------------------------------
soc_cRef1_ToSite                                   Service site referred to (report by client)
-----------------------------------------------------------------------------------------------

            type:  numeric (byte)
           label:  cRef_ToSite

           range:  [1,14]                     units:  1
   unique values:  14                     missing .:  576/4168
 unique mv codes:  3                     missing .*:  2849/4168

      tabulation:  Freq.   Numeric  Label
                       9         1  1. SFH Cairo Road
                      75         2  2. Chawama Clinic Maternity Ward
                       7         3  3. Chawama Clinic VMMC
                      76         4  4. Chawama Clinic TB, STI, HIV
                      28         5  5. Kamwala Clinic TB, STI, HIV
                      24         6  6. Kapata Clinic MCH
                     141         7  7. Kapata Clinic TB, STI, HIV
                      34         8  8. Chipata Gen Hosp VMMC
                     111         9  9. Chipata Gen Hosp MCH
                      41        10  10. Chipata Gen Hosp TB, STI,
                                    HIV
                      19        11  11. SFH Chipata New Start
                       9        12  12. SFH Chachacha Rd VMMC
                      39        13  13. SFH Chipata VMMC
                     130        14  14. Other/Not specific
                     576         .
                     205        .j  .j. Ineligible: External site
                                    client
```

```
                        2644        .l  .l. Ineligible: Experimental Arm

-------------------------------------------------------------------------------
soc_cRef1_Serv7d                          Service uptake within 7 days (report by client)
-------------------------------------------------------------------------------

                  type:  numeric (byte)
                 label:  yesno

                 range:  [0,1]                        units:  1
         unique values:  2                        missing .:  576/4168
       unique mv codes:  3                       missing .*:  2849/4168

            tabulation:  Freq.   Numeric  Label
                           692         0  0. No
                            51         1  1. Yes
                           576         .
                           205        .j  .j. Ineligible: External site
                                          client
                          2644        .l  .l. Ineligible: Experimental Arm

-------------------------------------------------------------------------------
soc_cRef1_Serv6w                         Service uptake within 6 weeks (report by client)
-------------------------------------------------------------------------------

                  type:  numeric (byte)
                 label:  yesno

                 range:  [0,1]                        units:  1
         unique values:  2                        missing .:  576/4168
       unique mv codes:  3                       missing .*:  2849/4168

            tabulation:  Freq.   Numeric  Label
                           616         0  0. No
                           127         1  1. Yes
                           576         .
                           205        .j  .j. Ineligible: External site
                                          client
                          2644        .l  .l. Ineligible: Experimental Arm

-------------------------------------------------------------------------------
soc_cRef1_Serv6m                        Service uptake within 6 months (report by client)
-------------------------------------------------------------------------------

                  type:  numeric (byte)
                 label:  yesno

                 range:  [0,1]                        units:  1
         unique values:  2                        missing .:  577/4168
       unique mv codes:  3                       missing .*:  2849/4168

            tabulation:  Freq.   Numeric  Label
                           518         0  0. No
                           224         1  1. Yes
                           577         .
                           205        .j  .j. Ineligible: External site
                                          client
                          2644        .l  .l. Ineligible: Experimental Arm

-------------------------------------------------------------------------------
soc_cRef1_UptakeVerified                              Service uptake verified in chart
-------------------------------------------------------------------------------

                  type:  numeric (byte)
                 label:  yesno

                 range:  [0,1]                        units:  1
         unique values:  2                        missing .:  1095/4168
       unique mv codes:  3                       missing .*:  2849/4168
```

```
           tabulation:  Freq.   Numeric  Label
                          160         0  0. No
                           64         1  1. Yes
                         1095         .  
                          205        .j  .j. Ineligible: External site
                                         client
                         2644        .l  .l. Ineligible: Experimental Arm

-----------------------------------------------------------------------------------------------
soc_cRef2_ServiceFor                                   Service referred for (report by client)
-----------------------------------------------------------------------------------------------

                 type:  numeric (byte)
                label:  cRef_ServiceFor

                range:  [1,15]                       units:  1
        unique values:  8                        missing .:  810/4168
      unique mv codes:  3                       missing .*:  2849/4168

           tabulation:  Freq.   Numeric  Label
                          134         1  1. Cervical Cancer Screening
                           36         4  4. FP
                           93         6  6. Couples HTC
                           44         7  7. Individual HTC
                           19         9  9. CD4 Screening
                            1        11  11. TB screening
                          128        12  12. STI screening
                           54        15  15. VMMC
                          810         .  
                          205        .j  .j. Ineligible: External site
                                         client
                         2644        .l  .l. Ineligible: Experimental Arm

-----------------------------------------------------------------------------------------------
soc_cRef2_ReferralFOR                                  For whom is referral (report by client)
-----------------------------------------------------------------------------------------------

                 type:  numeric (byte)
                label:  cRef_ReferralFOR

                range:  [0,3]                        units:  1
        unique values:  4                        missing .:  811/4168
      unique mv codes:  3                       missing .*:  2849/4168

           tabulation:  Freq.   Numeric  Label
                          352         0  0. Self
                          143         1  1. Spouse
                            8         2  2. Child
                            5         3  3. Other
                          811         .  
                          205        .j  .j. Ineligible: External site
                                         client
                         2644        .l  .l. Ineligible: Experimental Arm

-----------------------------------------------------------------------------------------------
soc_cRef2_ToSite                                   Service site referred to (report by client)
-----------------------------------------------------------------------------------------------

                 type:  numeric (byte)
                label:  cRef_ToSite

                range:  [1,14]                       units:  1
        unique values:  14                       missing .:  810/4168
      unique mv codes:  3                       missing .*:  2849/4168

           tabulation:  Freq.   Numeric  Label
                           20         1  1. SFH Cairo Road
                           48         2  2. Chawama Clinic Maternity Ward
                            6         3  3. Chawama Clinic VMMC
                           39         4  4. Chawama Clinic TB, STI, HIV
```

```
                          19         5  5. Kamwala Clinic TB, STI, HIV
                          16         6  6. Kapata Clinic MCH
                         115         7  7. Kapata Clinic TB, STI, HIV
                          17         8  8. Chipata Gen Hosp VMMC
                          76         9  9. Chipata Gen Hosp MCH
                          37        10  10. Chipata Gen Hosp TB, STI,
                                        HIV
                          15        11  11. SFH Chipata New Start
                           5        12  12. SFH Chachacha Rd VMMC
                          15        13  13. SFH Chipata VMMC
                          81        14  14. Other/Not specific
                         810         .
                         205        .j  .j. Ineligible: External site
                                        client
                        2644        .l  .l. Ineligible: Experimental Arm

-----------------------------------------------------------------------------------------------
soc_cRef2_Serv7d                               Service uptake within 7 days (report by client)
-----------------------------------------------------------------------------------------------

                  type:  numeric (byte)
                 label:  yesno

                 range:  [0,1]                        units:  1
         unique values:  2                        missing .:  810/4168
       unique mv codes:  3                       missing .*:  2849/4168

            tabulation:  Freq.   Numeric  Label
                         502         0  0. No
                           7         1  1. Yes
                         810         .
                         205        .j  .j. Ineligible: External site
                                        client
                        2644        .l  .l. Ineligible: Experimental Arm

-----------------------------------------------------------------------------------------------
soc_cRef2_Serv6w                              Service uptake within 6 weeks (report by client)
-----------------------------------------------------------------------------------------------

                  type:  numeric (byte)
                 label:  yesno

                 range:  [0,1]                        units:  1
         unique values:  2                        missing .:  810/4168
       unique mv codes:  3                       missing .*:  2849/4168

            tabulation:  Freq.   Numeric  Label
                         484         0  0. No
                          25         1  1. Yes
                         810         .
                         205        .j  .j. Ineligible: External site
                                        client
                        2644        .l  .l. Ineligible: Experimental Arm

-----------------------------------------------------------------------------------------------
soc_cRef2_Serv6m                             Service uptake within 6 months (report by client)
-----------------------------------------------------------------------------------------------

                  type:  numeric (byte)
                 label:  yesno

                 range:  [0,1]                        units:  1
         unique values:  2                        missing .:  810/4168
       unique mv codes:  3                       missing .*:  2849/4168

            tabulation:  Freq.   Numeric  Label
                         451         0  0. No
                          58         1  1. Yes
                         810         .
                         205        .j  .j. Ineligible: External site
```

```
                                    client
                         2644        .l  .l. Ineligible: Experimental Arm

-------------------------------------------------------------------------------------------------
soc_cRef2_UptakeVerified                                     Service uptake verified in chart
-------------------------------------------------------------------------------------------------

                  type:  numeric (byte)
                 label:  yesno

                 range:  [0,1]                        units:  1
         unique values:  2                        missing .:  1261/4168
       unique mv codes:  3                       missing .*:  2849/4168

            tabulation:  Freq.   Numeric  Label
                            43         0  0. No
                            15         1  1. Yes
                          1261         .
                           205        .j  .j. Ineligible: External site
                                          client
                          2644        .l  .l. Ineligible: Experimental Arm

-------------------------------------------------------------------------------------------------
soc_cRef3_ServiceFor                                  Service referred for (report by client)
-------------------------------------------------------------------------------------------------

                  type:  numeric (byte)
                 label:  cRef_ServiceFor

                 range:  [1,15]                       units:  1
         unique values:  7                        missing .:  1079/4168
       unique mv codes:  3                       missing .*:  2849/4168

            tabulation:  Freq.   Numeric  Label
                            49         1  1. Cervical Cancer Screening
                            26         4  4. FP
                            56         6  6. Couples HTC
                            16         7  7. Individual HTC
                             3         9  9. CD4 Screening
                            22        12  12. STI screening
                            68        15  15. VMMC
                          1079         .
                           205        .j  .j. Ineligible: External site
                                          client
                          2644        .l  .l. Ineligible: Experimental Arm

-------------------------------------------------------------------------------------------------
soc_cRef3_ReferralFOR                                 For whom is referral (report by client)
-------------------------------------------------------------------------------------------------

                  type:  numeric (byte)
                 label:  cRef_ReferralFOR

                 range:  [0,3]                        units:  1
         unique values:  4                        missing .:  1079/4168
       unique mv codes:  3                       missing .*:  2849/4168

            tabulation:  Freq.   Numeric  Label
                           103         0  0. Self
                           125         1  1. Spouse
                             2         2  2. Child
                            10         3  3. Other
                          1079         .
                           205        .j  .j. Ineligible: External site
                                          client
                          2644        .l  .l. Ineligible: Experimental Arm

-------------------------------------------------------------------------------------------------
soc_cRef3_ToSite                                   Service site referred to (report by client)
-------------------------------------------------------------------------------------------------
```

```
                  type:  numeric (byte)
                 label:  cRef_ToSite

                 range:  [1,14]                       units:  1
         unique values:  14                       missing .:  1079/4168
       unique mv codes:  3                       missing .*:  2849/4168

            tabulation:  Freq.   Numeric  Label
                             6         1  1. SFH Cairo Road
                            13         2  2. Chawama Clinic Maternity Ward
                             9         3  3. Chawama Clinic VMMC
                            11         4  4. Chawama Clinic TB, STI, HIV
                             8         5  5. Kamwala Clinic TB, STI, HIV
                            12         6  6. Kapata Clinic MCH
                            47         7  7. Kapata Clinic TB, STI, HIV
                            25         8  8. Chipata Gen Hosp VMMC
                            29         9  9. Chipata Gen Hosp MCH
                             6        10  10. Chipata Gen Hosp TB, STI,
                                          HIV
                            15        11  11. SFH Chipata New Start
                             3        12  12. SFH Chachacha Rd VMMC
                            15        13  13. SFH Chipata VMMC
                            41        14  14. Other/Not specific
                          1079         .
                           205        .j  .j. Ineligible: External site
                                          client
                          2644        .l  .l. Ineligible: Experimental Arm

-----------------------------------------------------------------------------------------------
soc_cRef3_Serv7d                               Service uptake within 7 days (report by client)
-----------------------------------------------------------------------------------------------

                  type:  numeric (byte)
                 label:  yesno

                 range:  [0,1]                        units:  1
         unique values:  2                        missing .:  1078/4168
       unique mv codes:  3                       missing .*:  2849/4168

            tabulation:  Freq.   Numeric  Label
                           240         0  0. No
                             1         1  1. Yes
                          1078         .
                           205        .j  .j. Ineligible: External site
                                          client
                          2644        .l  .l. Ineligible: Experimental Arm

-----------------------------------------------------------------------------------------------
soc_cRef3_Serv6w                              Service uptake within 6 weeks (report by client)
-----------------------------------------------------------------------------------------------

                  type:  numeric (byte)
                 label:  yesno

                 range:  [0,1]                        units:  1
         unique values:  2                        missing .:  1078/4168
       unique mv codes:  3                       missing .*:  2849/4168

            tabulation:  Freq.   Numeric  Label
                           238         0  0. No
                             3         1  1. Yes
                          1078         .
                           205        .j  .j. Ineligible: External site
                                          client
                          2644        .l  .l. Ineligible: Experimental Arm

-----------------------------------------------------------------------------------------------
soc_cRef3_Serv6m                             Service uptake within 6 months (report by client)
-----------------------------------------------------------------------------------------------
```

```
                type:  numeric (byte)
               label:  yesno

               range:  [0,1]                        units:  1
       unique values:  2                        missing .:  1078/4168
     unique mv codes:  3                       missing .*:  2849/4168

          tabulation:  Freq.   Numeric  Label
                         234         0  0. No
                           7         1  1. Yes
                        1078         .
                         205        .j  .j. Ineligible: External site
                                        client
                        2644        .l  .l. Ineligible: Experimental Arm

-----------------------------------------------------------------------------------------------
soc_cRef3_UptakeVerified                                      Service uptake verified in chart
-----------------------------------------------------------------------------------------------

                type:  numeric (byte)
               label:  yesno

               range:  [0,1]                        units:  1
       unique values:  2                        missing .:  1312/4168
     unique mv codes:  3                       missing .*:  2849/4168

          tabulation:  Freq.   Numeric  Label
                           6         0  0. No
                           1         1  1. Yes
                        1312         .
                         205        .j  .j. Ineligible: External site
                                        client
                        2644        .l  .l. Ineligible: Experimental Arm

-----------------------------------------------------------------------------------------------
soc_ServiceForfRef4                                                          4 ServiceForfRef
-----------------------------------------------------------------------------------------------

                type:  numeric (byte)
               label:  fRef_ServiceFor

               range:  [.,.]                        units:  .
       unique values:  0                        missing .:  1319/4168
     unique mv codes:  3                       missing .*:  2849/4168

          tabulation:  Freq.   Numeric  Label
                        1319         .
                         205        .j  .j. Ineligible: External site
                                        client
                        2644        .l  .l. Ineligible: Experimental Arm

-----------------------------------------------------------------------------------------------
soc_ToSitefRef4                                                                  4 ToSitefRef
-----------------------------------------------------------------------------------------------

                type:  numeric (byte)
               label:  fRef_ToSite

               range:  [.,.]                        units:  .
       unique values:  0                        missing .:  1319/4168
     unique mv codes:  3                       missing .*:  2849/4168

          tabulation:  Freq.   Numeric  Label
                        1319         .
                         205        .j  .j. Ineligible: External site
                                        client
                        2644        .l  .l. Ineligible: Experimental Arm

-----------------------------------------------------------------------------------------------
```

```
soc_ServfRef4                                                          4 ServfRef
-------------------------------------------------------------------------------

                  type:  numeric (byte)
                 label:  fRef_Serv

                 range:  [.,.]                        units:  .
         unique values:  0                        missing .:  1319/4168
       unique mv codes:  3                       missing .*:  2849/4168

            tabulation:  Freq.   Numeric  Label
                          1319         .
                           205        .j  .j. Ineligible: External site
                                          client
                          2644        .l  .l. Ineligible: Experimental Arm

-------------------------------------------------------------------------------
soc_ServiceForcRef4                                              4 ServiceForcRef
-------------------------------------------------------------------------------

                  type:  numeric (byte)
                 label:  cRef_ServiceFor

                 range:  [6,15]                       units:  1
         unique values:  3                        missing .:  1310/4168
       unique mv codes:  3                       missing .*:  2849/4168

            tabulation:  Freq.   Numeric  Label
                             5         6  6. Couples HTC
                             1        12  12. STI screening
                             3        15  15. VMMC
                          1310         .
                           205        .j  .j. Ineligible: External site
                                          client
                          2644        .l  .l. Ineligible: Experimental Arm

-------------------------------------------------------------------------------
soc_ReferralFORcRef4                                            4 ReferralFORcRef
-------------------------------------------------------------------------------

                  type:  numeric (byte)
                 label:  cRef_ReferralFOR

                 range:  [0,3]                        units:  1
         unique values:  4                        missing .:  1310/4168
       unique mv codes:  3                       missing .*:  2849/4168

            tabulation:  Freq.   Numeric  Label
                             1         0  0. Self
                             6         1  1. Spouse
                             1         2  2. Child
                             1         3  3. Other
                          1310         .
                           205        .j  .j. Ineligible: External site
                                          client
                          2644        .l  .l. Ineligible: Experimental Arm

-------------------------------------------------------------------------------
soc_ToSitecRef4                                                      4 ToSitecRef
-------------------------------------------------------------------------------

                  type:  numeric (byte)
                 label:  cRef_ToSite

                 range:  [7,14]                       units:  1
         unique values:  4                        missing .:  1310/4168
       unique mv codes:  3                       missing .*:  2849/4168

            tabulation:  Freq.   Numeric  Label
                             4         7  7. Kapata Clinic TB, STI, HIV
```

```
                         2         8  8. Chipata Gen Hosp VMMC
                         2        10  10. Chipata Gen Hosp TB, STI,
                                      HIV
                         1        14  14. Other/Not specific
                      1310         .
                       205        .j  .j. Ineligible: External site
                                      client
                      2644        .l  .l. Ineligible: Experimental Arm

-----------------------------------------------------------------------------------------------
soc_Serv7dcRef4                                                                    4 Serv7dcRef
-----------------------------------------------------------------------------------------------

                  type:  numeric (byte)
                 label:  yesno

                 range:  [0,0]                        units:  1
         unique values:  1                        missing .:  1310/4168
       unique mv codes:  3                       missing .*:  2849/4168

            tabulation:  Freq.   Numeric  Label
                             9         0  0. No
                          1310         .
                           205        .j  .j. Ineligible: External site
                                          client
                          2644        .l  .l. Ineligible: Experimental Arm

-----------------------------------------------------------------------------------------------
soc_Serv6wcRef4                                                                    4 Serv6wcRef
-----------------------------------------------------------------------------------------------

                  type:  numeric (byte)
                 label:  yesno

                 range:  [0,1]                        units:  1
         unique values:  2                        missing .:  1310/4168
       unique mv codes:  3                       missing .*:  2849/4168

            tabulation:  Freq.   Numeric  Label
                             8         0  0. No
                             1         1  1. Yes
                          1310         .
                           205        .j  .j. Ineligible: External site
                                          client
                          2644        .l  .l. Ineligible: Experimental Arm

-----------------------------------------------------------------------------------------------
soc_Serv6mcRef4                                                                    4 Serv6mcRef
-----------------------------------------------------------------------------------------------

                  type:  numeric (byte)
                 label:  yesno

                 range:  [0,1]                        units:  1
         unique values:  2                        missing .:  1310/4168
       unique mv codes:  3                       missing .*:  2849/4168

            tabulation:  Freq.   Numeric  Label
                             8         0  0. No
                             1         1  1. Yes
                          1310         .
                           205        .j  .j. Ineligible: External site
                                          client
                          2644        .l  .l. Ineligible: Experimental Arm

-----------------------------------------------------------------------------------------------
soc_UptakeVerifiedcRef4                                                    4 UptakeVerifiedcRef
-----------------------------------------------------------------------------------------------

                  type:  numeric (byte)
```

```
                label:  yesno

                range:  [.,.]                        units:  .
        unique values:  1                        missing .:  1318/4168
      unique mv codes:  3                       missing .*:  2849/4168

           tabulation:  Freq.   Numeric  Label
                            1         0  0. No
                         1318         .
                          205        .j  .j. Ineligible: External site
                                         client
                         2644        .l  .l. Ineligible: Experimental Arm

-----------------------------------------------------------------------------------------------
soc_Comments                                                                            Comments
-----------------------------------------------------------------------------------------------

                 type:  string (str128)

        unique values:  260                      missing "":  3837/4168

             examples:  ""
                        ""
                        ""
                        ""

              warning:  variable has leading, embedded, and trailing blanks

-----------------------------------------------------------------------------------------------
RefSelfTotal_CTD_an                      Number Referrals for Client: Client Tracking Database
-----------------------------------------------------------------------------------------------

                 type:  numeric (byte)

                range:  [0,3]                        units:  1
        unique values:  4                        missing .:  0/4168
      unique mv codes:  1                       missing .*:  205/4168

           tabulation:  Freq.  Value
                         2327  0
                         1246  1
                          379  2
                           11  3
                          205  .j

RefSelfTotal_CTD_an:
  1.  created for analysis. Excludes Ref*_Source_an = 3 (cases where client showed up for
      services without referral.)

-----------------------------------------------------------------------------------------------
RefPartnerTotal_CTD_an         Number Referrals for Client's Partner: Client Tracking Database
-----------------------------------------------------------------------------------------------

                 type:  numeric (byte)

                range:  [0,3]                        units:  1
        unique values:  4                        missing .:  0/4168
      unique mv codes:  1                       missing .*:  205/4168

           tabulation:  Freq.  Value
                         3149  0
                          667  1
                          143  2
                            4  3
                          205  .j

RefPartnerTotal_CTD_an:
  1.  created for analysis. Excludes Ref*_Source_an = 3 (cases where client showed up for
      services without referral.)
```

```
-------------------------------------------------------------------------------------------
RefOtherTotal_CTD_an                  Number Referrals for Others: Client Tracking Database
-------------------------------------------------------------------------------------------

                  type:  numeric (byte)

                 range:  [0,3]                        units:  1
         unique values:  4                        missing .:  0/4168
       unique mv codes:  1                       missing .*:  205/4168

            tabulation:  Freq.  Value
                          3856  0
                           104  1
                             2  2
                             1  3
                           205  .j

RefOtherTotal_CTD_an:
  1.  created for analysis. Excludes Ref*_Source_an = 3 (cases where client showed up for
      services without referral.)

-------------------------------------------------------------------------------------------
RefTotal_CTD_an                            Total Number Referrals: Client Tracking Database
-------------------------------------------------------------------------------------------

                  type:  numeric (byte)

                 range:  [0,5]                        units:  1
         unique values:  6                        missing .:  0/4168
       unique mv codes:  1                       missing .*:  205/4168

            tabulation:  Freq.  Value
                          2006  0
                          1199  1
                           485  2
                           150  3
                           121  4
                             2  5
                           205  .j

RefTotal_CTD_an:
  1.  created for analysis. Excludes Ref*_Source_an = 3 (cases where client showed up for
      services without referral.)

-------------------------------------------------------------------------------------------
RefSelfTotal_SOC_an                          Number Referrals for Client: SOC Referral Data
-------------------------------------------------------------------------------------------

                  type:  numeric (byte)

                 range:  [0,3]                        units:  1
         unique values:  4                        missing .:  2644/4168
       unique mv codes:  2                       missing .*:  205/4168

            tabulation:  Freq.  Value
                           672  0
                           294  1
                           270  2
                            83  3
                          2644  .
                           205  .j

RefSelfTotal_SOC_an:
  1.  created for analysis.

-------------------------------------------------------------------------------------------
RefPartnerTotal_SOC_an               Number Referrals for Client's Partner: SOC Referral Data
-------------------------------------------------------------------------------------------

                  type:  numeric (byte)
```

```
                 range:  [0,3]                        units:  1
         unique values:  4                        missing .:  2644/4168
       unique mv codes:  2                       missing .*:  205/4168

            tabulation:  Freq.  Value
                           999  0
                           267  1
                            47  2
                             6  3
                          2644  .
                           205  .j

RefPartnerTotal_SOC_an:
  1.  created for analysis.

-----------------------------------------------------------------------------------------------
RefOtherTotal_SOC_an                            Number Referrals for Others: SOC Referral Data
-----------------------------------------------------------------------------------------------

                  type:  numeric (byte)

                 range:  [0,2]                        units:  1
         unique values:  3                        missing .:  2644/4168
       unique mv codes:  2                       missing .*:  205/4168

            tabulation:  Freq.  Value
                          1291  0
                            27  1
                             1  2
                          2644  .
                           205  .j

RefOtherTotal_SOC_an:
  1.  created for analysis.

-----------------------------------------------------------------------------------------------
RefTotal_SOC_an                                      Total Number Referrals: SOC Referral Data
-----------------------------------------------------------------------------------------------

                  type:  numeric (byte)

                 range:  [0,3]                        units:  1
         unique values:  4                        missing .:  2644/4168
       unique mv codes:  2                       missing .*:  205/4168

            tabulation:  Freq.  Value
                           576  0
                           235  1
                           268  2
                           240  3
                          2644  .
                           205  .j

RefTotal_SOC_an:
  1.  created for analysis.

-----------------------------------------------------------------------------------------------
RefTotal_an                                  Total Number Referrals: CTD and SOC Referral Data
-----------------------------------------------------------------------------------------------

                  type:  numeric (byte)

                 range:  [0,5]                        units:  1
         unique values:  6                        missing .:  0/4168
       unique mv codes:  1                       missing .*:  205/4168

            tabulation:  Freq.  Value
                          1277  0
                          1422  1
```

```
                           749  2
                           390  3
                           123  4
                             2  5
                           205  .j

RefTotal_an:
  1.  created for analysis.

-----------------------------------------------------------------------------------------------
RefSelfTotal_an                         Number Referrals for Client: CTD and SOC Referral Data
-----------------------------------------------------------------------------------------------

                  type:  numeric (byte)

                 range:  [0,3]                        units:  1
         unique values:  4                        missing .:  0/4168
       unique mv codes:  1                       missing .*:  205/4168

            tabulation:  Freq.  Value
                          1690  0
                          1532  1
                           647  2
                            94  3
                           205  .j

RefSelfTotal_an:
  1.  created for analysis.

-----------------------------------------------------------------------------------------------
RefPartnerTotal_an            Number Referrals for Client's Partner: CTD and SOC Referral Data
-----------------------------------------------------------------------------------------------

                  type:  numeric (byte)

                 range:  [0,3]                        units:  1
         unique values:  4                        missing .:  0/4168
       unique mv codes:  1                       missing .*:  205/4168

            tabulation:  Freq.  Value
                          2829  0
                           934  1
                           190  2
                            10  3
                           205  .j

RefPartnerTotal_an:
  1.  created for analysis.

-----------------------------------------------------------------------------------------------
RefOtherTotal_an                        Number Referrals for Others: CTD and SOC Referral Data
-----------------------------------------------------------------------------------------------

                  type:  numeric (byte)

                 range:  [0,3]                        units:  1
         unique values:  4                        missing .:  0/4168
       unique mv codes:  1                       missing .*:  205/4168

            tabulation:  Freq.  Value
                          3828  0
                           131  1
                             3  2
                             1  3
                           205  .j

RefOtherTotal_an:
  1.  created for analysis.

-----------------------------------------------------------------------------------------------
```

```
EscortTotal_an                                 Total number of escorts client received
----------------------------------------------------------------------------------------

                  type:  numeric (byte)
                 label:  EscortTotal_an, but 4 nonmissing values are not labeled

                 range:  [0,3]                        units:  1
         unique values:  4                        missing .:  0/4168
       unique mv codes:  3                       missing .*:  3184/4168

            tabulation:  Freq.   Numeric  Label
                           280         0
                           612         1
                            86         2
                             6         3
                          2642        .a  .a. Not in integrated arm
                           337        .b  .b. No referrals
                           205        .j  .j. Ineligible: External site
                                          client

----------------------------------------------------------------------------------------
bl_time                                                                       Time Point
----------------------------------------------------------------------------------------

                  type:  numeric (byte)
                 label:  time

                 range:  [0,0]                        units:  1
         unique values:  1                        missing .:  0/4168

            tabulation:  Freq.   Numeric  Label
                          4168         0  0. Baseline

----------------------------------------------------------------------------------------
bl_ID                                                         Baseline Survey: Survey ID
----------------------------------------------------------------------------------------

                  type:  numeric (int)

                 range:  [98,14920]                   units:  1
         unique values:  3832                     missing .:  0/4168

                  mean:   8571.86
              std. dev:   3246.98

           percentiles:        10%       25%       50%       75%       90%
                              3936    5987.5    8929.5   11261.5     12513

----------------------------------------------------------------------------------------
bl_Date                            Baseline Survey: Interviewer entered date, System timestamp
----------------------------------------------------------------------------------------

                  type:  numeric (double)

                 range:  [1.703e+12,1.742e+12]        units:  1000
         unique values:  4163                     missing .:  0/4168

                  mean:   1.7e+12
              std. dev:   6.5e+09

           percentiles:        10%       25%       50%       75%       90%
                           1.7e+12   1.7e+12   1.7e+12   1.7e+12   1.7e+12

----------------------------------------------------------------------------------------
bl_SurveyStatus                                           Baseline Survey: Survey Status
----------------------------------------------------------------------------------------

                  type:  numeric (byte)
                 label:  SurveyStatus
```

```
              range:  [0,2]                        units:  1
      unique values:  3                        missing .:  0/4168

         tabulation:  Freq.   Numeric  Label
                          7         0  0. Interrupted
                       4097         1  1. Completed
                         64         2  2. Completed on resumption

-----------------------------------------------------------------------------------------------
bl_AdminAge                                        Baseline Survey: Age entered by interviewer
-----------------------------------------------------------------------------------------------

               type:  numeric (byte)

              range:  [18,48]                      units:  1
      unique values:  31                       missing .:  0/4168

               mean:   26.5837
           std. dev:   6.59386

        percentiles:        10%       25%       50%       75%       90%
                             19        21        25        31        36

bl_AdminAge:
  1.  Do NOT use this variable for analysis. Entered for administrative purposes. Should use
      bl_102a.

-----------------------------------------------------------------------------------------------
bl_SurveyGender   Baseline Survey: Gender of survey version completed (NOT PARTICIPANT GENDER)
-----------------------------------------------------------------------------------------------

               type:  string (str6)

      unique values:  2                        missing "":  0/4168

         tabulation:  Freq.  Value
                       2133  "Female"
                       2035  "Male"

bl_SurveyGender:
  1.  Survey gender does NOT always match true participant gender.

-----------------------------------------------------------------------------------------------
bl_InterviewerID                                               Baseline Survey: Interviewer ID
-----------------------------------------------------------------------------------------------

               type:  numeric (int)

              range:  [100,223]                    units:  1
      unique values:  25                       missing .:  0/4168

         tabulation:  Freq.  Value
                        303  100
                        249  101
                        117  102
                        268  103
                        234  104
                        118  105
                        173  106
                        145  107
                         60  108
                        297  109
                         11  110
                        133  111
                        100  112
                         88  113
                        258  114
                        320  115
                        183  116
                        362  117
```

```
                         167  118
                         194  119
                         159  120
                          21  210
                           4  211
                         146  222
                          58  223

-------------------------------------------------------------------------------
bl_GUID_sys                                 Baseline Survey: Unique survey identifier
-------------------------------------------------------------------------------

                  type:  string (str36)

         unique values:  4168                     missing "":  0/4168

              examples:  "35a164be-6876-4ae2-886e-9819a3bdab21"
                         "684dc1cf-696d-450a-8430-cca9fb9a084c"
                         "9ae128d9-f9b3-4184-8a4f-4ec1883ea7da"
                         "ce5ea272-0bb5-4ac8-a7ca-1e09828e57fd"

-------------------------------------------------------------------------------
bl_SiteID                                            Baseline Survey: Survey Site ID
-------------------------------------------------------------------------------

                  type:  numeric (byte)
                 label:  bl_SiteID

                 range:  [1,95]                       units:  1
         unique values:  12                       missing .:  0/4168

            tabulation:  Freq.   Numeric  Label
                           557         1  1. SFH HCT - Cairo Road
                           490         2  2. Chawama Clinic - Maternity
                                          Ward
                           244         3  3. Chawama Clinic - Out Patient
                                          Ward (VMMC)
                           362         5  5. Kamwala Clinic - TB, STI &
                                          HIV Clinic
                           545         6  6. Kapata Urban Clinic, MCH
                           775         7  7. Kapata Urban Clinic, TB, STI
                                          & HIV
                           189         8  8. Chipata Gen Hosp - OP VMMC
                           713        11  11. SFH New Start
                            88        13  13. SFH VMMC
                            68        93  93. YWCA FP Site
                            68        94  94. YWCA HTC Site
                            69        95  95. YWCA VMMC

bl_SiteID:
  1.  93, 94, & 95 are external evaluation sites

-------------------------------------------------------------------------------
bl_SiteName                                      Baseline Survey: Survey Site String
-------------------------------------------------------------------------------

                  type:  string (str40)

         unique values:  14                       missing "":  0/4168

            tabulation:  Freq.  Value
                           490  "Chawama Clinic - Maternity Ward"
                           244  "Chawama Clinic - Out Patient Ward
                                (VMMC)"
                           189  "Chipata Gen Hosp - OP VMMC"
                           214  "Kamwala Clinic - TB, STI & HIV Clinic"
                           148  "Kamwala Clinic - TB, STI and HIV
                                Clinic"
                           545  "Kapata Urban Clinic, MCH"
                           413  "Kapata Urban Clinic, TB, STI & HIV"
```

```
                           362  "Kapata Urban Clinic, TB, STI and HIV"
                           557  "SFH HCT - Cairo Road"
                           713  "SFH New Start"
                            88  "SFH VMMC"
                            68  "YWCA FP Site"
                            68  "YWCA HTC Site"
                            69  "YWCA VMMC"

               warning:  variable has embedded blanks

bl_SiteName:
  1.  YWCA sites are external evaluation sites

-----------------------------------------------------------------------------------------------
bl_SurveyType                                                      Baseline Survey: Survey Type
-----------------------------------------------------------------------------------------------

                  type:  numeric (byte)
                 label:  SurveyType

                 range:  [1,2]                        units:  1
         unique values:  2                        missing .:  0/4168

            tabulation:  Freq.   Numeric  Label
                          2133         1  1. Baseline Female
                          2035         2  2. Baseline Male

-----------------------------------------------------------------------------------------------
bl_SurveyTypeName                                           Baseline Survey: Survey Type String
-----------------------------------------------------------------------------------------------

                  type:  string (str27)

         unique values:  2                        missing "":  0/4168

            tabulation:  Freq.  Value
                          2133  "Behavioral Baseline -Female"
                          2035  "Behavioral Baseline -Male"

               warning:  variable has embedded blanks

-----------------------------------------------------------------------------------------------
bl_SurveyDuration_sys                                             Minutes for survey completion
-----------------------------------------------------------------------------------------------

                  type:  numeric (int)

                 range:  [-704,1578]                  units:  1
         unique values:  90                       missing .:  0/4168

                  mean:   38.2354
              std. dev:   126.728

           percentiles:        10%       25%       50%       75%       90%
                                20        24        29        35        42

bl_SurveyDuration_sys:
  1.  Large values may be due to completing survey on resumption (see SurveyStatus).

-----------------------------------------------------------------------------------------------
bl_Weekday_sys                                                     Baseline Survey: Day of week
-----------------------------------------------------------------------------------------------

                  type:  string (str3)

         unique values:  7                        missing "":  0/4168

            tabulation:  Freq.  Value
                           820  "Fri"
                           881  "Mon"
```

```
                           45  "Sat"
                           19  "Sun"
                          887  "Thu"
                          780  "Tue"
                          736  "Wed"

bl_Weekday_sys:
  1.  Generated automatically based on bl_Date

-----------------------------------------------------------------------------------------------
bl_Day_sys                                                        Baseline Survey: Day of month
-----------------------------------------------------------------------------------------------

                  type:  numeric (byte)

                 range:  [1,31]                       units:  1
         unique values:  31                       missing .:  0/4168

                  mean:   15.4592
              std. dev:   8.67083

           percentiles:        10%       25%       50%       75%       90%
                                 4         8        15        23        28

bl_Day_sys:
  1.  Generated automatically based on bl_Date

-----------------------------------------------------------------------------------------------
bl_Month_sys                                                      Baseline Survey: Day of month
-----------------------------------------------------------------------------------------------

                  type:  numeric (byte)

                 range:  [1,12]                       units:  1
         unique values:  12                       missing .:  0/4168

            tabulation:  Freq.  Value
                           165  1
                           394  2
                           612  3
                           534  4
                           590  5
                           599  6
                           385  7
                           340  8
                           283  9
                           155  10
                            62  11
                            49  12

bl_Month_sys:
  1.  Generated automatically based on bl_Date

-----------------------------------------------------------------------------------------------
bl_DBVersion                                                       Baseline Survey: App Version
-----------------------------------------------------------------------------------------------

                  type:  numeric (double)

                 range:  [1.4,2.6]                    units:  .1
         unique values:  10                       missing .:  0/4168

            tabulation:  Freq.  Value
                           262  1.4
                             1  1.5
                           214  1.6
                            45  1.8
                           111  1.9
                            50  2
                          1175  2.1
```

```
                           927  2.2
                           998  2.5
                           385  2.6

--------------------------------------------------------------------------------------------
bl_AppVersion                                                                     AppVersion
--------------------------------------------------------------------------------------------

                  type:  numeric (double)

                 range:  [1.4,2.6]                    units:  .1
         unique values:  9                        missing .:  0/4168

            tabulation:  Freq.  Value
                           263  1.4
                           214  1.6
                            45  1.8
                           111  1.9
                            50  2
                          1175  2.1
                           927  2.2
                           998  2.5
                           385  2.6

--------------------------------------------------------------------------------------------
bl_100a                                                           100: Hour interview began
--------------------------------------------------------------------------------------------

                  type:  numeric (byte)

                 range:  [6,17]                       units:  1
         unique values:  12                       missing .:  0/4168
       unique mv codes:  1                       missing .*:  1/4168

            tabulation:  Freq.  Value
                            97  6
                             3  7
                            56  8
                           567  9
                           971  10
                           939  11
                           692  12
                           291  13
                           256  14
                           251  15
                            43  16
                             1  17
                             1  .m

--------------------------------------------------------------------------------------------
bl_100b                                                         100: Minute interview began
--------------------------------------------------------------------------------------------

                  type:  numeric (byte)

                 range:  [0,59]                       units:  1
         unique values:  60                       missing .:  0/4168
       unique mv codes:  1                       missing .*:  1/4168

                  mean:   28.8275
             std. dev:    17.2749

           percentiles:        10%       25%       50%       75%       90%
                                 5        14        30        44        53

--------------------------------------------------------------------------------------------
bl_101a                                                                 101: Month of birth
--------------------------------------------------------------------------------------------

                  type:  numeric (byte)
```

```
                label:  num_bl, but 12 nonmissing values are not labeled

                range:  [1,88]                       units:  1
        unique values:  13                       missing .:  0/4168

          tabulation:  Freq.   Numeric  Label
                          380         1
                          332         2
                          329         3
                          326         4
                          297         5
                          412         6
                          282         7
                          354         8
                          297         9
                          389        10
                          272        11
                          382        12
                          116        88  88. Don't Know

--------------------------------------------------------------------------------------------
bl_101b                                                                  101: Year of birth
--------------------------------------------------------------------------------------------

                 type:  numeric (int)
                label:  numyr, but 33 nonmissing values are not labeled

                range:  [1964,8888]                  units:  1
        unique values:  34                       missing .:  0/4168

             examples:  1981
                        1987
                        1990
                        1993

--------------------------------------------------------------------------------------------
bl_102a                                      102: Age at last birthday reported by respondent
--------------------------------------------------------------------------------------------

                 type:  numeric (byte)

                range:  [18,49]                      units:  1
        unique values:  32                       missing .:  0/4168

                 mean:    26.517
             std. dev:   6.63663

          percentiles:        10%       25%       50%       75%       90%
                               19        21        25        31        36

--------------------------------------------------------------------------------------------
bl_102b                                                  102: How was age determined in 102a?
--------------------------------------------------------------------------------------------

                 type:  numeric (byte)
                label:  bl_102b

                range:  [1,3]                        units:  1
        unique values:  3                        missing .:  0/4168

           tabulation:  Freq.   Numeric  Label
                         4095         1  1. Age or year known by resp
                           57         2  2. Est. using period/eras
                           16         3  3. Est. using visual
                                         approximation

--------------------------------------------------------------------------------------------
bl_103                                                        103: Living in village currently
--------------------------------------------------------------------------------------------
```

```
                  type:  numeric (byte)
                 label:  yesno_bl

                 range:  [0,1]                        units:  1
         unique values:  2                        missing .:  0/4168

           tabulation:  Freq.   Numeric  Label
                         3928         0  0. No
                          240         1  1. Yes

-------------------------------------------------------------------------------------------
bl_104                                                                    104: Village Name
-------------------------------------------------------------------------------------------

                  type:  string (str41)

         unique values:  155                      missing "":  0/4168

              examples:  "."
                         "."
                         "."
                         "."

               warning:  variable has embedded blanks

-------------------------------------------------------------------------------------------
bl_105                                                                       105: Town name
-------------------------------------------------------------------------------------------

                  type:  string (str31)

         unique values:  802                      missing "":  0/4168

              examples:  "Chipata"
                         "Kamwala South"
                         "Lusaka"
                         "Muthlansembe"

               warning:  variable has embedded blanks

-------------------------------------------------------------------------------------------
bl_106                                                              106: Ever attended school
-------------------------------------------------------------------------------------------

                  type:  numeric (byte)
                 label:  yesno_bl

                 range:  [0,1]                        units:  1
         unique values:  2                        missing .:  0/4168

           tabulation:  Freq.   Numeric  Label
                          230         0  0. No
                         3938         1  1. Yes

-------------------------------------------------------------------------------------------
bl_107                                                        107: Currently attending school
-------------------------------------------------------------------------------------------

                  type:  numeric (byte)
                 label:  bl_107

                 range:  [0,1]                        units:  1
         unique values:  2                        missing .:  0/4168
       unique mv codes:  1                       missing .*:  230/4168

           tabulation:  Freq.   Numeric  Label
                         3042         0  0. No
                          896         1  1. Yes
                          230        .a  .a. Never attended school
```

```
-------------------------------------------------------------------------------------------
bl_108                                             108: Highest level of school attended
-------------------------------------------------------------------------------------------

                  type:  numeric (byte)
                 label:  bl_108

                 range:  [1,4]                        units:  1
         unique values:  4                        missing .:  0/4168
       unique mv codes:  1                       missing .*:  230/4168

            tabulation:  Freq.   Numeric  Label
                           962         1  1. Primary
                          2357         2  2. Secondary
                           308         3  3. Trade School/Private College
                           311         4  4. University/College
                           230        .a  .a. Never attended school

-------------------------------------------------------------------------------------------
bl_109                                           109: Highest grade completed in primary
-------------------------------------------------------------------------------------------

                  type:  numeric (byte)
                 label:  bl_109, but 7 nonmissing values are not labeled

                 range:  [0,7]                        units:  1
         unique values:  8                        missing .:  0/4168
       unique mv codes:  4                       missing .*:  3206/4168

            tabulation:  Freq.   Numeric  Label
                             2         0  0. Did not complete 1st grade
                            15         1
                            41         2
                            70         3
                           108         4
                           137         5
                           182         6
                           407         7
                           230        .a  .a. Never attended school
                          2357        .c  .c. Secondary
                           308        .d  .d. Trade school/private college
                           311        .f  .f. University

-------------------------------------------------------------------------------------------
bl_110                              110: Years completed in trade school/private college
-------------------------------------------------------------------------------------------

                  type:  numeric (byte)
                 label:  bl_110, but 5 nonmissing values are not labeled

                 range:  [0,6]                        units:  1
         unique values:  6                        missing .:  0/4168
       unique mv codes:  4                       missing .*:  3860/4168

            tabulation:  Freq.   Numeric  Label
                            18         0  0. Did not complete 1st year
                            98         1
                           125         2
                            61         3
                             5         4
                             1         6
                           230        .a  .a. Never attended school
                           962        .b  .b. Primary
                          2357        .c  .c. Secondary
                           311        .f  .f. University

-------------------------------------------------------------------------------------------
bl_111                                         111: Highest grade completed in secondary
-------------------------------------------------------------------------------------------
```

```
                  type:  numeric (byte)
                 label:  bl_111, but 5 nonmissing values are not labeled

                 range:  [0,12]                       units:  1
         unique values:  6                        missing .:  0/4168
       unique mv codes:  4                       missing .*:  1504/4168

            tabulation:  Freq.   Numeric  Label
                             9         0  0. Did not complete 8th grade
                           263         8
                           614         9
                           213        10
                           295        11
                          1270        12
                           230        .a  .a. Never attended school
                           962        .b  .b. Primary
                           311        .f  .f. University
                             1        .s  .s. Skipped by participant

-----------------------------------------------------------------------------------------------
bl_112                                              112: Years completed in university/college
-----------------------------------------------------------------------------------------------

                  type:  numeric (byte)
                 label:  bl_112, but 8 nonmissing values are not labeled

                 range:  [0,8]                        units:  1
         unique values:  9                        missing .:  0/4168
       unique mv codes:  4                       missing .*:  3856/4168

            tabulation:  Freq.   Numeric  Label
                             9         0  0. Did not complete 1st year
                            67         1
                           102         2
                            74         3
                            44         4
                            11         5
                             3         6
                             1         7
                             1         8
                           230        .a  .a. Never attended school
                           962        .b  .b. Primary
                          2357        .c  .c. Secondary
                           307        .d  .d. Trade school/private college

-----------------------------------------------------------------------------------------------
bl_113a1                                                113: Community primary school attended
-----------------------------------------------------------------------------------------------

                  type:  numeric (byte)
                 label:  bl_113a

                 range:  [0,1]                        units:  1
         unique values:  2                        missing .:  0/4168
       unique mv codes:  1                       missing .*:  230/4168

            tabulation:  Freq.   Numeric  Label
                          3615         0  0. No
                           323         1  1. Yes
                           230        .a  .a. Never attended school

-----------------------------------------------------------------------------------------------
bl_113a2                                               113: Government primary school attended
-----------------------------------------------------------------------------------------------

                  type:  numeric (byte)
                 label:  bl_113a

                 range:  [0,1]                        units:  1
         unique values:  2                        missing .:  0/4168
```

```
      unique mv codes:  1                      missing .*:  230/4168

           tabulation:  Freq.   Numeric  Label
                          462         0  0. No
                         3476         1  1. Yes
                          230        .a  .a. Never attended school

-------------------------------------------------------------------------------
bl_113a3                                  113: Mission primary school attended
-------------------------------------------------------------------------------

                 type:  numeric (byte)
                label:  bl_113a

                range:  [0,1]                       units:  1
        unique values:  2                       missing .:  0/4168
      unique mv codes:  1                      missing .*:  230/4168

           tabulation:  Freq.   Numeric  Label
                         3776         0  0. No
                          162         1  1. Yes
                          230        .a  .a. Never attended school

-------------------------------------------------------------------------------
bl_113a4                                  113: Private primary school attended
-------------------------------------------------------------------------------

                 type:  numeric (byte)
                label:  bl_113a

                range:  [0,1]                       units:  1
        unique values:  2                       missing .:  0/4168
      unique mv codes:  1                      missing .*:  230/4168

           tabulation:  Freq.   Numeric  Label
                         3532         0  0. No
                          406         1  1. Yes
                          230        .a  .a. Never attended school

-------------------------------------------------------------------------------
bl_113b1                             113: Government secondary school attended
-------------------------------------------------------------------------------

                 type:  numeric (byte)
                label:  bl_113b

                range:  [0,1]                       units:  1
        unique values:  2                       missing .:  0/4168
      unique mv codes:  3                      missing .*:  1811/4168

           tabulation:  Freq.   Numeric  Label
                          231         0  0. No
                         2126         1  1. Yes
                          230        .a  .a. Never attended school
                          962        .b  .b. Primary
                          619        .e  .e. Skip error: Trade
                                         school/university did not answer

-------------------------------------------------------------------------------
bl_113b2                                113: Mission secondary school attended
-------------------------------------------------------------------------------

                 type:  numeric (byte)
                label:  bl_113b

                range:  [0,1]                       units:  1
        unique values:  2                       missing .:  0/4168
      unique mv codes:  3                      missing .*:  1811/4168

           tabulation:  Freq.   Numeric  Label
```

```
                     2217         0  0. No
                      140         1  1. Yes
                      230        .a  .a. Never attended school
                      962        .b  .b. Primary
                      619        .e  .e. Skip error: Trade
                                     school/university did not answer

-------------------------------------------------------------------------------------------
bl_113b3                                             113: Private secondary school attended
-------------------------------------------------------------------------------------------

                  type:  numeric (byte)
                 label:  bl_113b

                 range:  [0,1]                        units:  1
         unique values:  2                        missing .:  0/4168
       unique mv codes:  3                       missing .*:  1811/4168

            tabulation:  Freq.   Numeric  Label
                         2160         0  0. No
                          197         1  1. Yes
                          230        .a  .a. Never attended school
                          962        .b  .b. Primary
                          619        .e  .e. Skip error: Trade
                                         school/university did not answer

-------------------------------------------------------------------------------------------
bl_114                                                                        114: Religion
-------------------------------------------------------------------------------------------

                  type:  numeric (byte)
                 label:  bl_114

                 range:  [1,5]                        units:  1
         unique values:  5                        missing .:  0/4168

            tabulation:  Freq.   Numeric  Label
                          789         1  1. Catholic
                         3182         2  2. Other Christian
                           47         3  3. Muslim
                           32         4  4. No religion
                          118         5  5. Other

-------------------------------------------------------------------------------------------
bl_115a                                                       115: Ethnic group/Tribe: Lozi
-------------------------------------------------------------------------------------------

                  type:  numeric (byte)
                 label:  yesno_bl

                 range:  [0,1]                        units:  1
         unique values:  2                        missing .:  0/4168
       unique mv codes:  1                       missing .*:  1/4168

            tabulation:  Freq.   Numeric  Label
                         4007         0  0. No
                          160         1  1. Yes
                            1        .s  .s. Skipped by participant

-------------------------------------------------------------------------------------------
bl_115b                                                      115: Ethnic group/Tribe: Ngoni
-------------------------------------------------------------------------------------------

                  type:  numeric (byte)
                 label:  yesno_bl

                 range:  [0,1]                        units:  1
         unique values:  2                        missing .:  0/4168
       unique mv codes:  1                       missing .*:  1/4168
```

```
          tabulation:  Freq.   Numeric  Label
                        2845         0  0. No
                        1322         1  1. Yes
                           1        .s  .s. Skipped by participant
```

```
-----------------------------------------------------------------------------------------------
bl_115c                                                       115: Ethnic group/Tribe: Tonga
-----------------------------------------------------------------------------------------------

                  type:  numeric (byte)
                 label:  yesno_bl

                 range:  [0,1]                        units:  1
         unique values:  2                        missing .:  0/4168
       unique mv codes:  1                       missing .*:  1/4168

            tabulation:  Freq.   Numeric  Label
                          3885         0  0. No
                           282         1  1. Yes
                             1        .s  .s. Skipped by participant
```

```
-----------------------------------------------------------------------------------------------
bl_115d                                                       115: Ethnic group/Tribe: Lunda
-----------------------------------------------------------------------------------------------

                  type:  numeric (byte)
                 label:  yesno_bl

                 range:  [0,1]                        units:  1
         unique values:  2                        missing .:  0/4168
       unique mv codes:  1                       missing .*:  1/4168

            tabulation:  Freq.   Numeric  Label
                          4127         0  0. No
                            40         1  1. Yes
                             1        .s  .s. Skipped by participant
```

```
-----------------------------------------------------------------------------------------------
bl_115e                                                       115: Ethnic group/Tribe: Bemba
-----------------------------------------------------------------------------------------------

                  type:  numeric (byte)
                 label:  yesno_bl

                 range:  [0,1]                        units:  1
         unique values:  2                        missing .:  0/4168
       unique mv codes:  1                       missing .*:  1/4168

            tabulation:  Freq.   Numeric  Label
                          3510         0  0. No
                           657         1  1. Yes
                             1        .s  .s. Skipped by participant
```

```
-----------------------------------------------------------------------------------------------
bl_115f                                                      115: Ethnic group/Tribe: Kaonde
-----------------------------------------------------------------------------------------------

                  type:  numeric (byte)
                 label:  yesno_bl

                 range:  [0,1]                        units:  1
         unique values:  2                        missing .:  0/4168
       unique mv codes:  1                       missing .*:  1/4168

            tabulation:  Freq.   Numeric  Label
                          4098         0  0. No
                            69         1  1. Yes
                             1        .s  .s. Skipped by participant

-----------------------------------------------------------------------------------------------
```

```
bl_115g                                              115: Ethnic group/Tribe: Luvale
-------------------------------------------------------------------------------------------

                  type:  numeric (byte)
                 label:  yesno_bl

                 range:  [0,1]                        units:  1
         unique values:  2                        missing .:  0/4168
       unique mv codes:  1                       missing .*:  1/4168

            tabulation:  Freq.   Numeric  Label
                          4115         0  0. No
                            52         1  1. Yes
                             1        .s  .s. Skipped by participant

-------------------------------------------------------------------------------------------
bl_115h                                115: Ethnic group/Tribe: Non-Zambian (Foreign Born)
-------------------------------------------------------------------------------------------

                  type:  numeric (byte)
                 label:  yesno_bl

                 range:  [0,1]                        units:  1
         unique values:  2                        missing .:  0/4168
       unique mv codes:  1                       missing .*:  1/4168

            tabulation:  Freq.   Numeric  Label
                          4110         0  0. No
                            57         1  1. Yes
                             1        .s  .s. Skipped by participant

-------------------------------------------------------------------------------------------
bl_115i                                               115: Ethnic group/Tribe: Other
-------------------------------------------------------------------------------------------

                  type:  numeric (byte)
                 label:  yesno_bl

                 range:  [0,1]                        units:  1
         unique values:  2                        missing .:  0/4168
       unique mv codes:  1                       missing .*:  1/4168

            tabulation:  Freq.   Numeric  Label
                          2480         0  0. No
                          1687         1  1. Yes
                             1        .s  .s. Skipped by participant

-------------------------------------------------------------------------------------------
bl_122                                         122: Source of drinking water for household
-------------------------------------------------------------------------------------------

                  type:  numeric (byte)
                 label:  bl_122

                 range:  [11,62]                      units:  1
         unique values:  12                       missing .:  0/4168
       unique mv codes:  1                       missing .*:  1/4168

            tabulation:  Freq.   Numeric  Label
                           719        11  11. Piped into dwelling
                          1424        12  12. Piped to yard/plot
                          1227        13  13. Public tap/standpipe
                           119        21  21. Open well/spring in
                                          yard/plot
                            91        22  22. Open public well/spring
                           106        31  31. Protected well/spring in
                                          yard/plot
                            51        32  32. Protected public well/spring
                           145        41  41. Borehole in yard/plot
                           254        42  42. Public borehole
```

```
                          3        51  51. River/stream
                         10        61  61. Bottled water
                         18        62  62. Other
                          1        .s  .s. Skipped by participant

-------------------------------------------------------------------------------
bl_123                                    123: Travel time to drinking water (minutes)
-------------------------------------------------------------------------------

                  type:  numeric (int)
                 label:  bl_123, but 24 nonmissing values are not labeled

                 range:  [0,888]                      units:  1
         unique values:  26                       missing .:  0/4168
       unique mv codes:  2                       missing .*:  2409/4168

            tabulation:  Freq.   Numeric  Label
                           121         0  0. On premises
                           139         1
                           232         2
                           234         3
                            84         4
                           486         5
                            17         6
                            18         7
                            10         8
                             1         9
                           226        10
                             4        12
                            46        15
                            45        20
                             1        21
                            52        30
                             1        35
                             3        40
                             1        45
                             9        60
                             1        64
                             1        65
                             1        90
                             4       120
                             1       180
                            21       888  888. Don't Know
                          2407        .a  .a. Skipped due to answer in
                                          bl_122
                             2        .s  .s. Skipped by participant

-------------------------------------------------------------------------------
bl_124                                           124: Toilet facility for household
-------------------------------------------------------------------------------

                  type:  numeric (byte)
                 label:  bl_124

                 range:  [1,8]                        units:  1
         unique values:  8                        missing .:  0/4168
       unique mv codes:  1                       missing .*:  1/4168

            tabulation:  Freq.   Numeric  Label
                          1161         1  1. Flush toilet
                           120         2  2. VIP latrine
                           478         3  3. Covered pit latrine no slab
                          1631         4  4. Covered pit latrine w/ slab
                           286         5  5. Uncovered pit latrine no slab
                           483         6  6. Uncovered pit latrine w/ slab
                             2         7  7. Bush/throw away
                             6         8  8. Other
                             1        .s  .s. Skipped by participant

-------------------------------------------------------------------------------
```

```
bl_125                         125: Number of household using toilet (excluding R's household)
-----------------------------------------------------------------------------------------------

                  type:  numeric (byte)
                 label:  num_bl, but 17 nonmissing values are not labeled

                 range:  [0,88]                       units:  1
         unique values:  18                       missing .:  0/4168
       unique mv codes:  2                       missing .*:  3/4168

           tabulation:  Freq.   Numeric  Label
                         2189         0
                          455         1
                          507         2
                          419         3
                          232         4
                          125         5
                           72         6
                           48         7
                           32         8
                           15         9
                           19        10
                            4        11
                            3        12
                            3        13
                            1        14
                            7        15
                           17        16
                           17        88  88. Don't Know
                            2        .m  .m. Missing
                            1        .s  .s. Skipped by participant

-----------------------------------------------------------------------------------------------
bl_126a                                                  126: Household inventory: Electricity
-----------------------------------------------------------------------------------------------

                  type:  numeric (byte)
                 label:  yesno_bl

                 range:  [0,1]                        units:  1
         unique values:  2                        missing .:  0/4168
       unique mv codes:  1                       missing .*:  1/4168

           tabulation:  Freq.   Numeric  Label
                         1415         0  0. No
                         2752         1  1. Yes
                            1        .s  .s. Skipped by participant

-----------------------------------------------------------------------------------------------
bl_126b                                                        126: Household inventory: Radio
-----------------------------------------------------------------------------------------------

                  type:  numeric (byte)
                 label:  yesno_bl

                 range:  [0,1]                        units:  1
         unique values:  2                        missing .:  0/4168
       unique mv codes:  1                       missing .*:  1/4168

           tabulation:  Freq.   Numeric  Label
                         1183         0  0. No
                         2984         1  1. Yes
                            1        .s  .s. Skipped by participant

-----------------------------------------------------------------------------------------------
bl_126c                                              126: Household inventory: Cassette Player
-----------------------------------------------------------------------------------------------

                  type:  numeric (byte)
                 label:  yesno_bl
```

```
            range:  [0,1]                        units:  1
    unique values:  2                        missing .:  0/4168
  unique mv codes:  1                       missing .*:  1/4168

       tabulation:  Freq.   Numeric  Label
                     2603         0  0. No
                     1564         1  1. Yes
                        1        .s  .s. Skipped by participant

-----------------------------------------------------------------------------------------------
bl_126d                                                 126: Household inventory: Television
-----------------------------------------------------------------------------------------------

             type:  numeric (byte)
            label:  yesno_bl

            range:  [0,1]                        units:  1
    unique values:  2                        missing .:  0/4168
  unique mv codes:  1                       missing .*:  1/4168

       tabulation:  Freq.   Numeric  Label
                     1217         0  0. No
                     2950         1  1. Yes
                        1        .s  .s. Skipped by participant

-----------------------------------------------------------------------------------------------
bl_126e                                               126: Household inventory: Mobile Phone
-----------------------------------------------------------------------------------------------

             type:  numeric (byte)
            label:  yesno_bl

            range:  [0,1]                        units:  1
    unique values:  2                        missing .:  0/4168
  unique mv codes:  1                       missing .*:  1/4168

       tabulation:  Freq.   Numeric  Label
                      775         0  0. No
                     3392         1  1. Yes
                        1        .s  .s. Skipped by participant

-----------------------------------------------------------------------------------------------
bl_126f                                                126: Household inventory: Fixed Phone
-----------------------------------------------------------------------------------------------

             type:  numeric (byte)
            label:  yesno_bl

            range:  [0,1]                        units:  1
    unique values:  2                        missing .:  0/4168
  unique mv codes:  1                       missing .*:  1/4168

       tabulation:  Freq.   Numeric  Label
                     4013         0  0. No
                      154         1  1. Yes
                        1        .s  .s. Skipped by participant

-----------------------------------------------------------------------------------------------
bl_126g                                               126: Household inventory: Refrigerator
-----------------------------------------------------------------------------------------------

             type:  numeric (byte)
            label:  yesno_bl

            range:  [0,1]                        units:  1
    unique values:  2                        missing .:  0/4168
  unique mv codes:  1                       missing .*:  1/4168

       tabulation:  Freq.   Numeric  Label
```

```
                     2205         0  0. No
                     1962         1  1. Yes
                        1        .s  .s. Skipped by participant

-------------------------------------------------------------------------------
bl_126h                                    126: Household inventory: Table
-------------------------------------------------------------------------------

                  type:  numeric (byte)
                 label:  yesno_bl

                 range:  [0,1]                        units:  1
         unique values:  2                        missing .:  0/4168
       unique mv codes:  1                       missing .*:  1/4168

            tabulation:  Freq.   Numeric  Label
                           780         0  0. No
                          3387         1  1. Yes
                             1        .s  .s. Skipped by participant

-------------------------------------------------------------------------------
bl_126i                                     126: Household inventory: Sofa
-------------------------------------------------------------------------------

                  type:  numeric (byte)
                 label:  yesno_bl

                 range:  [0,1]                        units:  1
         unique values:  2                        missing .:  0/4168
       unique mv codes:  1                       missing .*:  1/4168

            tabulation:  Freq.   Numeric  Label
                          1114         0  0. No
                          3053         1  1. Yes
                             1        .s  .s. Skipped by participant

-------------------------------------------------------------------------------
bl_126j                                      126: Household inventory: Bed
-------------------------------------------------------------------------------

                  type:  numeric (byte)
                 label:  yesno_bl

                 range:  [0,1]                        units:  1
         unique values:  2                        missing .:  0/4168
       unique mv codes:  1                       missing .*:  1/4168

            tabulation:  Freq.   Numeric  Label
                           657         0  0. No
                          3510         1  1. Yes
                             1        .s  .s. Skipped by participant

-------------------------------------------------------------------------------
bl_126k                     126: Household inventory: CD/Digital music player
-------------------------------------------------------------------------------

                  type:  numeric (byte)
                 label:  yesno_bl

                 range:  [0,1]                        units:  1
         unique values:  2                        missing .:  0/4168
       unique mv codes:  1                       missing .*:  1/4168

            tabulation:  Freq.   Numeric  Label
                          2026         0  0. No
                          2141         1  1. Yes
                             1        .s  .s. Skipped by participant

-------------------------------------------------------------------------------
bl_126l                               126: Household inventory: VCR/DVD player
```

```
--------------------------------------------------------------------------------------------

                  type:  numeric (byte)
                 label:  yesno_bl

                 range:  [0,1]                        units:  1
         unique values:  2                        missing .:  0/4168
       unique mv codes:  1                       missing .*:  1/4168

            tabulation:  Freq.   Numeric  Label
                          1673         0  0. No
                          2494         1  1. Yes
                             1        .s  .s. Skipped by participant

--------------------------------------------------------------------------------------------
bl_126m                                                       126: Household inventory: Car
--------------------------------------------------------------------------------------------

                  type:  numeric (byte)
                 label:  yesno_bl

                 range:  [0,1]                        units:  1
         unique values:  2                        missing .:  0/4168
       unique mv codes:  1                       missing .*:  1/4168

            tabulation:  Freq.   Numeric  Label
                          3486         0  0. No
                           681         1  1. Yes
                             1        .s  .s. Skipped by participant

--------------------------------------------------------------------------------------------
bl_126n                                                126: Household inventory: Motorcycle
--------------------------------------------------------------------------------------------

                  type:  numeric (byte)
                 label:  yesno_bl

                 range:  [0,1]                        units:  1
         unique values:  2                        missing .:  0/4168
       unique mv codes:  1                       missing .*:  1/4168

            tabulation:  Freq.   Numeric  Label
                          3983         0  0. No
                           184         1  1. Yes
                             1        .s  .s. Skipped by participant

--------------------------------------------------------------------------------------------
bl_126o                                                   126: Household inventory: Bicycle
--------------------------------------------------------------------------------------------

                  type:  numeric (byte)
                 label:  yesno_bl

                 range:  [0,1]                        units:  1
         unique values:  2                        missing .:  0/4168
       unique mv codes:  1                       missing .*:  1/4168

            tabulation:  Freq.   Numeric  Label
                          2849         0  0. No
                          1318         1  1. Yes
                             1        .s  .s. Skipped by participant

--------------------------------------------------------------------------------------------
bl_127                                                         127: Household owns house
--------------------------------------------------------------------------------------------

                  type:  numeric (byte)
                 label:  yesno_bl

                 range:  [0,1]                        units:  1
```

```
        unique values:  2                        missing .:  0/4168
      unique mv codes:  1                       missing .*:  1/4168

          tabulation:  Freq.   Numeric  Label
                        2465         0  0. No
                        1702         1  1. Yes
                           1        .s  .s. Skipped by participant

-------------------------------------------------------------------------------------------
bl_128                                                   128: Own personal cell/mobile phone
-------------------------------------------------------------------------------------------

                 type:  numeric (byte)
                label:  yesno_bl

                range:  [0,1]                        units:  1
        unique values:  2                        missing .:  0/4168

          tabulation:  Freq.   Numeric  Label
                         684         0  0. No
                        3484         1  1. Yes

-------------------------------------------------------------------------------------------
bl_129                                                       129: Kind of work to earn money
-------------------------------------------------------------------------------------------

                 type:  string (str65)

        unique values:  763                      missing "":  0/4168

             examples:  "0 Does not work"
                        "0 Does not work"
                        "Business"
                        "Mechanic"

              warning:  variable has embedded blanks

-------------------------------------------------------------------------------------------
bl_130a                                                        130: Total amount money earned
-------------------------------------------------------------------------------------------

                 type:  numeric (byte)
                label:  bl_130a

                range:  [0,31]                       units:  1
        unique values:  31                       missing .:  0/4168
      unique mv codes:  2                       missing .*:  2281/4168

             examples:  13    13. 551-600 KR
                        26    26. 3001-3500 KR
                        .a    .a. Does not work
                        .a    .a. Does not work

bl_130a:
  1.  Due to programming error, both 51-100 KR and 701-750 KR were coded as 16

-------------------------------------------------------------------------------------------
bl_130b                                                                  130: Unit of measure
-------------------------------------------------------------------------------------------

                 type:  numeric (byte)
                label:  bl_130b

                range:  [1,5]                        units:  1
        unique values:  5                        missing .:  0/4168
      unique mv codes:  3                       missing .*:  2325/4168

          tabulation:  Freq.   Numeric  Label
                           6         1  1. Hourly
                         481         2  2. Daily
```

```
                    1285         3  3. Monthly
                      35         4  4. Yearly
                      36         5  5. Paid in-kind
                    2209        .a  .a. Does not work
                      49        .b  .b. Earns nothing
                      67        .s  .s. Skipped by participant

-------------------------------------------------------------------------------
bl_200                                  200: Ever married or lived with someone
-------------------------------------------------------------------------------

                  type:  numeric (byte)
                 label:  bl_200

                 range:  [0,3]                        units:  1
         unique values:  4                        missing .:  0/4168
       unique mv codes:  1                       missing .*:  1/4168

            tabulation:  Freq.   Numeric  Label
                          1705         0  0. No
                          2178         1  1. Yes, married
                           256         2  2. Yes, lived with partner
                            28         3  3. Both
                             1        .s  .s. Skipped by participant

-------------------------------------------------------------------------------
bl_201                                  201: Currently have primary sex partner
-------------------------------------------------------------------------------

                  type:  numeric (byte)
                 label:  bl_201

                 range:  [0,1]                        units:  1
         unique values:  2                        missing .:  0/4168
       unique mv codes:  2                       missing .*:  2464/4168

            tabulation:  Freq.   Numeric  Label
                           514         0  0. No
                          1190         1  1. Yes
                          2462        .a  .a. Ever married/lived with
                                            partner
                             2        .s  .s. Skipped by participant

-------------------------------------------------------------------------------
bl_202                                  202: Age when sexual relationship began
-------------------------------------------------------------------------------

                  type:  numeric (byte)
                 label:  bl_202, but 30 nonmissing values are not labeled

                 range:  [6,88]                       units:  1
         unique values:  31                       missing .:  0/4168
       unique mv codes:  3                       missing .*:  2983/4168

              examples:  22
                         .a    .a. Ever married/lived with partner
                         .a    .a. Ever married/lived with partner
                         .a    .a. Ever married/lived with partner

-------------------------------------------------------------------------------
bl_203                                          203: Age of primary sex partner
-------------------------------------------------------------------------------

                  type:  numeric (byte)
                 label:  bl_203, but 33 nonmissing values are not labeled

                 range:  [14,88]                      units:  1
         unique values:  34                       missing .:  0/4168
       unique mv codes:  4                       missing .*:  2986/4168
```

```
           examples:  24
                      .a    .a. Ever married/lived with partner
                      .a    .a. Ever married/lived with partner
                      .a    .a. Ever married/lived with partner

-----------------------------------------------------------------------------------------------
bl_204                                       204: Age started living with first spouse/partner
-----------------------------------------------------------------------------------------------

               type:  numeric (byte)
              label:  bl_204, but 33 nonmissing values are not labeled

              range:  [6,88]                       units:  1
      unique values:  34                       missing .:  0/4168
    unique mv codes:  3                       missing .*:  1707/4168

           examples:  19
                      23
                      .a    .a. Never married/lived with partner
                      .a    .a. Never married/lived with partner

-----------------------------------------------------------------------------------------------
bl_205                                               205: Currently married or living together
-----------------------------------------------------------------------------------------------

               type:  numeric (byte)
              label:  bl_205

              range:  [0,2]                        units:  1
      unique values:  3                        missing .:  0/4168
    unique mv codes:  2                       missing .*:  1706/4168

         tabulation:  Freq.   Numeric  Label
                        464         0  0. No
                       1922         1  1. Yes, married
                         76         2  2. Yes, living with partner
                       1705        .a  .a. Never married/lived with
                                       partner
                          1        .m  .m. Missing

-----------------------------------------------------------------------------------------------
bl_206                                       206: Current partner same person as first partner
-----------------------------------------------------------------------------------------------

               type:  numeric (byte)
              label:  bl_206

              range:  [0,1]                        units:  1
      unique values:  2                        missing .:  0/4168
    unique mv codes:  3                       missing .*:  2170/4168

         tabulation:  Freq.   Numeric  Label
                        385         0  0. No
                       1613         1  1. Yes
                       1705        .a  .a. Never married/lived with
                                       partner
                        464        .b  .b. Not currently married/living
                                       with partner
                          1        .m  .m. Missing

-----------------------------------------------------------------------------------------------
bl_207                                            207: Age started living with current partner
-----------------------------------------------------------------------------------------------

               type:  numeric (byte)
              label:  bl_207, but 38 nonmissing values are not labeled

              range:  [7,88]                       units:  1
      unique values:  39                       missing .:  0/4168
    unique mv codes:  5                       missing .*:  3785/4168
```

```
           examples:  .a    .a. Never married/lived with partner
                      .a    .a. Never married/lived with partner
                      .b    .b. Not currently married/living with partner
                      .c    .c. Current partner is first partner: see 204

-----------------------------------------------------------------------------------------------
bl_208                                            208: Age of current partner at last birthday
-----------------------------------------------------------------------------------------------

                  type:  numeric (byte)
                 label:  bl_208, but 50 nonmissing values are not labeled

                 range:  [14,88]                      units:  1
         unique values:  51                       missing .:  0/4168
       unique mv codes:  4                       missing .*:  2174/4168

              examples:  29
                         43
                         .a    .a. Never married/lived with partner
                         .a    .a. Never married/lived with partner

-----------------------------------------------------------------------------------------------
bl_209                                                     209: Current partner staying with R
-----------------------------------------------------------------------------------------------

                  type:  numeric (byte)
                 label:  bl_209

                 range:  [1,2]                        units:  1
         unique values:  2                        missing .:  0/4168
       unique mv codes:  4                       missing .*:  2172/4168

            tabulation:  Freq.   Numeric  Label
                          1864         1  1. Staying with respondent
                           132         2  2. Staying elsewhere
                          1705        .a  .a. Never married/lived with
                                          partner
                           464        .b  .b. Not currently married/living
                                          with partner
                             1        .m  .m. Missing
                             2        .s  .s. Skipped by participant

-----------------------------------------------------------------------------------------------
bl_210                                                            210: Current marital status
-----------------------------------------------------------------------------------------------

                  type:  numeric (byte)
                 label:  bl_210

                 range:  [1,3]                        units:  1
         unique values:  3                        missing .:  0/4168
       unique mv codes:  4                       missing .*:  3749/4168

            tabulation:  Freq.   Numeric  Label
                            36         1  1. Widowed
                           127         2  2. Divorced
                           256         3  3. Separated
                          1705        .a  .a. Never married/lived with
                                          partner
                          1998        .b  .b. Currently married/living
                                          with partner
                             1        .m  .m. Missing
                            45        .s  .s. Skipped by participant

-----------------------------------------------------------------------------------------------
bl_211                                                211: Currently have primary sex partner
-----------------------------------------------------------------------------------------------

                  type:  numeric (byte)
```

```
                label:  bl_211

                range:  [0,1]                        units:  1
        unique values:  2                        missing .:  0/4168
      unique mv codes:  4                       missing .*:  3706/4168

          tabulation:  Freq.   Numeric  Label
                         191         0  0. No
                         271         1  1. Yes
                        1705        .a  .a. Never married/lived with
                                        partner
                        1998        .b  .b. Currently married/living
                                        with partner
                           1        .m  .m. Missing
                           2        .s  .s. Skipped by participant

--------------------------------------------------------------------------------
bl_212_m                                212: Number wives/partners currently live with
--------------------------------------------------------------------------------

                 type:  numeric (byte)
                label:  bl_212, but 10 nonmissing values are not labeled

                range:  [1,16]                       units:  1
        unique values:  10                       missing .:  0/4168
      unique mv codes:  3                       missing .*:  3535/4168

          tabulation:  Freq.   Numeric  Label
                         559         1
                          48         2
                          10         3
                           7         4
                           1         5
                           2         6
                           2         7
                           1         8
                           2         9
                           1        16
                        1197        .a  .a. Never married/lived with
                                        partner
                         203        .b  .b. Not currently married/living
                                        with partner
                        2135        .g  .g. Gender skip pattern

--------------------------------------------------------------------------------
bl_213                                          213: Total spouses or live-in partners
--------------------------------------------------------------------------------

                 type:  numeric (byte)
                label:  bl_213, but 17 nonmissing values are not labeled

                range:  [1,20]                       units:  1
        unique values:  17                       missing .:  0/4168
      unique mv codes:  3                       missing .*:  1715/4168

          tabulation:  Freq.   Numeric  Label
                        1538         1
                         523         2
                         164         3
                          88         4
                          57         5
                          22         6
                          15         7
                          14         8
                           5         9
                          14        10
                           2        11
                           1        12
                           1        13
                           3        15
```

```
                       2        16
                       2        19
                       2        20
                    1705        .a  .a. Never married/lived with
                                    partner
                       1        .m  .m. Missing
                       9        .s  .s. Skipped by participant

-------------------------------------------------------------------------------------------
bl_300                                                       300: Number biological children
-------------------------------------------------------------------------------------------

                  type:  numeric (byte)
                 label:  bl_300, but 12 nonmissing values are not labeled

                 range:  [0,14]                       units:  1
         unique values:  13                       missing .:  0/4168
       unique mv codes:  1                       missing .*:  1/4168

            tabulation:  Freq.   Numeric  Label
                          1440         0  0. No biological children
                           869         1
                           691         2
                           451         3
                           302         4
                           197         5
                           114         6
                            51         7
                            35         8
                             9         9
                             6        10
                             1        11
                             1        14
                             1        .s  .s. Skipped by participant

-------------------------------------------------------------------------------------------
bl_301a                                                               301: Number boys born
-------------------------------------------------------------------------------------------

                  type:  numeric (byte)
                 label:  bl_301a, but 9 nonmissing values are not labeled

                 range:  [0,8]                        units:  1
         unique values:  9                        missing .:  0/4168
       unique mv codes:  2                       missing .*:  1441/4168

            tabulation:  Freq.   Numeric  Label
                           670         0
                          1082         1
                           601         2
                           217         3
                           106         4
                            34         5
                            13         6
                             2         7
                             2         8
                          1440        .a  .a. No biological children
                             1        .s  .s. Skipped by participant

-------------------------------------------------------------------------------------------
bl_301b                                                              301: Number girls born
-------------------------------------------------------------------------------------------

                  type:  numeric (byte)
                 label:  bl_301b, but 9 nonmissing values are not labeled

                 range:  [0,8]                        units:  1
         unique values:  9                        missing .:  0/4168
       unique mv codes:  2                       missing .*:  1441/4168
```

```
           tabulation:  Freq.   Numeric  Label
                          730         0
                         1032         1
                          557         2
                          246         3
                          114         4
                           34         5
                            8         6
                            5         7
                            1         8
                         1440        .a  .a. No biological children
                            1        .s  .s. Skipped by participant

-----------------------------------------------------------------------------------------------
bl_302                                                      302: Number children who have died
-----------------------------------------------------------------------------------------------

                 type:  numeric (byte)
                label:  bl_302, but 6 nonmissing values are not labeled

                range:  [0,6]                        units:  1
        unique values:  7                        missing .:  0/4168
      unique mv codes:  3                       missing .*:  1442/4168

           tabulation:  Freq.   Numeric  Label
                         2211         0  0. No children have died
                          361         1
                          102         2
                           33         3
                           12         4
                            5         5
                            2         6
                         1440        .a  .a. No biological children
                            1        .m  .m. Missing
                            1        .s  .s. Skipped by participant

-----------------------------------------------------------------------------------------------
bl_303_m                                                 303: Number women fathered children with
-----------------------------------------------------------------------------------------------

                 type:  numeric (byte)
                label:  bl_303_m, but 7 nonmissing values are not labeled

                range:  [1,7]                        units:  1
        unique values:  7                        missing .:  0/4168
      unique mv codes:  4                       missing .*:  3298/4168

           tabulation:  Freq.   Numeric  Label
                          678         1
                          161         2
                           24         3
                            3         4
                            1         5
                            1         6
                            2         7
                         1161        .a  .a. No biological children
                         2135        .g  .g. Gender skip pattern
                            1        .m  .m. Missing
                            1        .s  .s. Skipped by participant

-----------------------------------------------------------------------------------------------
bl_304                                304: Number children who live with you but aren't biological
-----------------------------------------------------------------------------------------------

                 type:  numeric (byte)
                label:  num_bl, but 11 nonmissing values are not labeled

                range:  [0,10]                       units:  1
        unique values:  11                       missing .:  0/4168
      unique mv codes:  2                       missing .*:  30/4168
```

tabulation: Freq. Numeric Label

Freq.	Numeric	Label
2337	0	
521	1	
479	2	
300	3	
213	4	
123	5	
74	6	
40	7	
25	8	
12	9	
14	10	
1	.m	.m. Missing
29	.s	.s. Skipped by participant

---

bl_305a — 305: Number boys non-biological children

---

type: numeric (byte)
label: bl_305a, but 10 nonmissing values are not labeled

range: [0,9] units: 1
unique values: 10 missing .: 0/4168
unique mv codes: 2 missing .*: 2367/4168

tabulation:

Freq.	Numeric	Label
457	0	
659	1	
403	2	
173	3	
66	4	
23	5	
15	6	
3	7	
1	8	
1	9	
2337	.a	.a. No non-biological children
30	.s	.s. Skipped by participant

---

bl_305b — 305: Number girls non-biological children

---

type: numeric (byte)
label: bl_305b, but 9 nonmissing values are not labeled

range: [0,8] units: 1
unique values: 9 missing .: 0/4168
unique mv codes: 2 missing .*: 2367/4168

tabulation:

Freq.	Numeric	Label
456	0	
662	1	
392	2	
175	3	
71	4	
28	5	
13	6	
1	7	
3	8	
2337	.a	.a. No non-biological children
30	.s	.s. Skipped by participant

---

bl_306 — 306: Currently pregnant

---

type: numeric (byte)

```
              label:  bl_306

              range:  [0,88]                       units:  1
      unique values:  3                        missing .:  0/4168
    unique mv codes:  4                       missing .*:  1406/4168

        tabulation:  Freq.   Numeric  Label
                      2605         0  0. No
                       127         1  1. Yes
                        30        88  88. Don't Know
                      1196        .a  .a. Males: Never married/lived
                                      with partner
                       204        .b  .b. Males: Not currently
                                      married/living with partner
                         2        .c  .c. Males: Reported no
                                      wife/partner on this question
                                      (bl_209 == 2 for both cases)
                         4        .s  .s. Skipped by participant

---------------------------------------------------------------------------------------------
bl_307a                       307: Pregnant: After child you are expecting, want more children
---------------------------------------------------------------------------------------------

               type:  numeric (byte)
              label:  bl_307a

              range:  [0,88]                       units:  1
      unique values:  4                        missing .:  0/4168
    unique mv codes:  6                       missing .*:  4043/4168

        tabulation:  Freq.   Numeric  Label
                        44         0  0. Want no more/none
                        74         1  1. Yes, have another child
                         1         2  2. Can't have children
                         6        88  88. Undecided/Don't know
                      1183        .a  .a. Males: Never married/lived
                                      with partner
                       204        .b  .b. Males: Not currently
                                      married/living with partner
                         2        .c  .c. Males: Reported no
                                      wife/partner on 306 (bl_209 == 2
                                      for both cases)
                      2635        .d  .d. Females/Males: Reported not
                                      pregnant or don't know in 306
                        13        .e  .e. Skip error: Programming
                                      error
                         6        .s  .s. Skipped by participant

---------------------------------------------------------------------------------------------
bl_307b                              307: Not pregnant/No partner pregnant: Want more children
---------------------------------------------------------------------------------------------

               type:  numeric (byte)
              label:  bl_307b

              range:  [0,88]                       units:  1
      unique values:  4                        missing .:  0/4168
    unique mv codes:  4                       missing .*:  271/4168

        tabulation:  Freq.   Numeric  Label
                       903         0  0. Want no more/none
                      2589         1  1. Yes, have another child
                        55         2  2. Can't have children
                       350        88  88. Undecided/Don't know
                       127        .d  .d. Females/Males: Reported
                                      pregnant in 306
                        13        .e  .e. Skip error: Programming
                                      error
                         1        .m  .m. Missing
                       130        .s  .s. Skipped by participant
```

```
--------------------------------------------------------------------------------------------
bl_308                                 308: How long want to wait before birth of another child
--------------------------------------------------------------------------------------------

                  type:  numeric (byte)
                 label:  bl_308

                 range:  [1,88]                         units:  1
         unique values:  50                         missing .:  0/4168
       unique mv codes:  2                         missing .*:  1493/4168

              examples:  28    28. 3 Years
                         32    32. 5 Years
                         87    87. Other
                         .a    .a. Want no more children, can't have children, or don't know i
> f want more children

--------------------------------------------------------------------------------------------
bl_309a                                                   309: Heard of: Female sterilization
--------------------------------------------------------------------------------------------

                  type:  numeric (byte)
                 label:  bl_309a

                 range:  [0,1]                          units:  1
         unique values:  2                          missing .:  0/4168
       unique mv codes:  1                         missing .*:  509/4168

            tabulation:  Freq.   Numeric  Label
                          1746         0  0. No
                          1913         1  1. Yes
                           509        .e  .e. Skip error: Males who
                                          answered 0,2,88 on 307 skipped
                                          to 400

--------------------------------------------------------------------------------------------
bl_309b                                                     309: Heard of: Male sterilization
--------------------------------------------------------------------------------------------

                  type:  numeric (byte)
                 label:  bl_309b

                 range:  [0,1]                          units:  1
         unique values:  2                          missing .:  0/4168
       unique mv codes:  1                         missing .*:  509/4168

            tabulation:  Freq.   Numeric  Label
                          2671         0  0. No
                           988         1  1. Yes
                           509        .e  .e. Skip error: Males who
                                          answered 0,2,88 on 307 skipped
                                          to 400

--------------------------------------------------------------------------------------------
bl_309c                                                                   309: Heard of: Pill
--------------------------------------------------------------------------------------------

                  type:  numeric (byte)
                 label:  bl_309c

                 range:  [0,1]                          units:  1
         unique values:  2                          missing .:  0/4168
       unique mv codes:  1                         missing .*:  509/4168

            tabulation:  Freq.   Numeric  Label
                           332         0  0. No
                          3327         1  1. Yes
                           509        .e  .e. Skip error: Males who
                                          answered 0,2,88 on 307 skipped
```

```
                                  to 400

-------------------------------------------------------------------------------
bl_309d                                                     309: Heard of: IUD
-------------------------------------------------------------------------------

                  type:  numeric (byte)
                 label:  bl_309d

                 range:  [0,1]                        units:  1
         unique values:  2                        missing .:  0/4168
       unique mv codes:  1                       missing .*:  509/4168

            tabulation:  Freq.   Numeric  Label
                          1676         0  0. No
                          1983         1  1. Yes
                           509        .e  .e. Skip error: Males who
                                          answered 0,2,88 on 307 skipped
                                          to 400

-------------------------------------------------------------------------------
bl_309e                                             309: Heard of: Injectables
-------------------------------------------------------------------------------

                  type:  numeric (byte)
                 label:  bl_309e

                 range:  [0,1]                        units:  1
         unique values:  2                        missing .:  0/4168
       unique mv codes:  2                       missing .*:  510/4168

            tabulation:  Freq.   Numeric  Label
                           388         0  0. No
                          3270         1  1. Yes
                           509        .e  .e. Skip error: Males who
                                          answered 0,2,88 on 307 skipped
                                          to 400
                             1        .m  .m. Missing

-------------------------------------------------------------------------------
bl_309f                                                309: Heard of: Implants
-------------------------------------------------------------------------------

                  type:  numeric (byte)
                 label:  bl_309f

                 range:  [0,1]                        units:  1
         unique values:  2                        missing .:  0/4168
       unique mv codes:  1                       missing .*:  509/4168

            tabulation:  Freq.   Numeric  Label
                          1001         0  0. No
                          2658         1  1. Yes
                           509        .e  .e. Skip error: Males who
                                          answered 0,2,88 on 307 skipped
                                          to 400

-------------------------------------------------------------------------------
bl_309g                                            309: Heard of: Male condom
-------------------------------------------------------------------------------

                  type:  numeric (byte)
                 label:  bl_309g

                 range:  [0,1]                        units:  1
         unique values:  2                        missing .:  0/4168
       unique mv codes:  1                       missing .*:  509/4168

            tabulation:  Freq.   Numeric  Label
                            82         0  0. No
```

```
                   3577         1  1. Yes
                    509        .e  .e. Skip error: Males who
                                   answered 0,2,88 on 307 skipped
                                   to 400

-----------------------------------------------------------------------------------------------
bl_309h                                                            309: Heard of: Female condom
-----------------------------------------------------------------------------------------------

                  type:  numeric (byte)
                 label:  bl_309h

                 range:  [0,1]                        units:  1
         unique values:  2                        missing .:  0/4168
       unique mv codes:  2                       missing .*:  510/4168

            tabulation:  Freq.   Numeric  Label
                            569         0  0. No
                           3089         1  1. Yes
                            509        .e  .e. Skip error: Males who
                                           answered 0,2,88 on 307 skipped
                                           to 400
                              1        .s  .s. Skipped by participant

-----------------------------------------------------------------------------------------------
bl_309k                                            309: Heard of: Lactational amenorrhea method
-----------------------------------------------------------------------------------------------

                  type:  numeric (byte)
                 label:  bl_309k

                 range:  [0,1]                        units:  1
         unique values:  2                        missing .:  0/4168
       unique mv codes:  2                       missing .*:  510/4168

            tabulation:  Freq.   Numeric  Label
                           2524         0  0. No
                           1134         1  1. Yes
                            509        .e  .e. Skip error: Males who
                                           answered 0,2,88 on 307 skipped
                                           to 400
                              1        .s  .s. Skipped by participant

-----------------------------------------------------------------------------------------------
bl_309l                                                            309: Heard of: Rhythm method
-----------------------------------------------------------------------------------------------

                  type:  numeric (byte)
                 label:  bl_309l

                 range:  [0,1]                        units:  1
         unique values:  2                        missing .:  0/4168
       unique mv codes:  1                       missing .*:  509/4168

            tabulation:  Freq.   Numeric  Label
                           1470         0  0. No
                           2189         1  1. Yes
                            509        .e  .e. Skip error: Males who
                                           answered 0,2,88 on 307 skipped
                                           to 400

-----------------------------------------------------------------------------------------------
bl_309m                                                               309: Heard of: Withdrawal
-----------------------------------------------------------------------------------------------

                  type:  numeric (byte)
                 label:  bl_309m

                 range:  [0,1]                        units:  1
         unique values:  2                        missing .:  0/4168
```

```
      unique mv codes:  1                       missing .*:  509/4168

           tabulation:  Freq.   Numeric  Label
                          733         0  0. No
                         2926         1  1. Yes
                          509        .e  .e. Skip error: Males who
                                         answered 0,2,88 on 307 skipped
                                         to 400

-----------------------------------------------------------------------------------------------
bl_309n                                                           309: Heard of: Other method
-----------------------------------------------------------------------------------------------

                 type:  numeric (byte)
                label:  bl_309n

                range:  [0,1]                        units:  1
        unique values:  2                        missing .:  0/4168
      unique mv codes:  1                       missing .*:  509/4168

           tabulation:  Freq.   Numeric  Label
                         3151         0  0. No
                          508         1  1. Yes
                          509        .e  .e. Skip error: Males who
                                         answered 0,2,88 on 307 skipped
                                         to 400

-----------------------------------------------------------------------------------------------
bl_310a_f                                        310: Ever had operation: Female sterilization
-----------------------------------------------------------------------------------------------

                 type:  numeric (byte)
                label:  bl_310a_f

                range:  [0,1]                        units:  1
        unique values:  2                        missing .:  0/4168
      unique mv codes:  4                       missing .*:  3092/4168

           tabulation:  Freq.   Numeric  Label
                         1039         0  0. No
                           37         1  1. Yes
                         1037        .a  .a. Never heard of method
                           21        .b  .b. Currently pregnant
                            1        .e  .e. Skip error: Males who
                                         answered 0,2,88 on 307 skipped
                                         to 400
                         2033        .g  .g. Gender skip pattern

-----------------------------------------------------------------------------------------------
bl_310b_m                                          310: Ever had operation: Male sterilization
-----------------------------------------------------------------------------------------------

                 type:  numeric (byte)
                label:  bl_310b_m

                range:  [0,1]                        units:  1
        unique values:  2                        missing .:  0/4168
      unique mv codes:  4                       missing .*:  3669/4168

           tabulation:  Freq.   Numeric  Label
                          492         0  0. No
                            7         1  1. Yes
                         1024        .a  .a. Never heard of method
                          508        .e  .e. Skip error: Males who
                                         answered 0,2,88 on 307 skipped
                                         to 400
                         2135        .g  .g. Gender skip pattern
                            2        .m  .m. Missing

-----------------------------------------------------------------------------------------------
```

```
bl_310c_f                                                    310: Ever used: Pill
-------------------------------------------------------------------------------------------

                  type:  numeric (byte)
                 label:  bl_310c_f

                 range:  [0,1]                        units:  1
         unique values:  2                        missing .:  0/4168
       unique mv codes:  4                       missing .*:  2160/4168

            tabulation:  Freq.   Numeric  Label
                           869         0  0. No
                          1139         1  1. Yes
                           125        .a  .a. Never heard of method
                             1        .e  .e. Skip error: Males who
                                          answered 0,2,88 on 307 skipped
                                          to 400
                          2033        .g  .g. Gender skip pattern
                             1        .m  .m. Missing

-------------------------------------------------------------------------------------------
bl_310d_f                                                     310: Ever used: IUD
-------------------------------------------------------------------------------------------

                  type:  numeric (byte)
                 label:  bl_310d_f

                 range:  [0,1]                        units:  1
         unique values:  2                        missing .:  0/4168
       unique mv codes:  3                       missing .*:  2722/4168

            tabulation:  Freq.   Numeric  Label
                          1332         0  0. No
                           114         1  1. Yes
                           688        .a  .a. Never heard of method
                             1        .e  .e. Skip error: Males who
                                          answered 0,2,88 on 307 skipped
                                          to 400
                          2033        .g  .g. Gender skip pattern

-------------------------------------------------------------------------------------------
bl_310e_f                                             310: Ever used: Injectables
-------------------------------------------------------------------------------------------

                  type:  numeric (byte)
                 label:  bl_310e_f

                 range:  [0,1]                        units:  1
         unique values:  2                        missing .:  0/4168
       unique mv codes:  4                       missing .*:  2200/4168

            tabulation:  Freq.   Numeric  Label
                           990         0  0. No
                           978         1  1. Yes
                           165        .a  .a. Never heard of method
                             1        .e  .e. Skip error: Males who
                                          answered 0,2,88 on 307 skipped
                                          to 400
                          2033        .g  .g. Gender skip pattern
                             1        .m  .m. Missing

-------------------------------------------------------------------------------------------
bl_310f_f                                                310: Ever used: Implants
-------------------------------------------------------------------------------------------

                  type:  numeric (byte)
                 label:  bl_310f_f

                 range:  [0,1]                        units:  1
         unique values:  2                        missing .:  0/4168
```

```
      unique mv codes:  3                      missing .*:  2410/4168

           tabulation:  Freq.   Numeric  Label
                         1469         0  0. No
                          289         1  1. Yes
                          376        .a  .a. Never heard of method
                            1        .e  .e. Skip error: Males who
                                         answered 0,2,88 on 307 skipped
                                         to 400
                         2033        .g  .g. Gender skip pattern

-----------------------------------------------------------------------------------------------
bl_310g                                                           310: Ever used: Male condom
-----------------------------------------------------------------------------------------------

                 type:  numeric (byte)
                label:  bl_310g

                range:  [0,1]                        units:  1
        unique values:  2                        missing .:  0/4168
      unique mv codes:  2                       missing .*:  591/4168

           tabulation:  Freq.   Numeric  Label
                          595         0  0. No
                         2982         1  1. Yes
                           82        .a  .a. Never heard of method
                          509        .e  .e. Skip error: Males who
                                         answered 0,2,88 on 307 skipped
                                         to 400

-----------------------------------------------------------------------------------------------
bl_310h_f                                                       310: Ever used: Female condom
-----------------------------------------------------------------------------------------------

                 type:  numeric (byte)
                label:  bl_310h_f

                range:  [0,1]                        units:  1
        unique values:  2                        missing .:  0/4168
      unique mv codes:  5                       missing .*:  2429/4168

           tabulation:  Freq.   Numeric  Label
                         1533         0  0. No
                          206         1  1. Yes
                          393        .a  .a. Never heard of method
                            1        .e  .e. Skip error: Males who
                                         answered 0,2,88 on 307 skipped
                                         to 400
                         2033        .g  .g. Gender skip pattern
                            1        .m  .m. Missing
                            1        .s  .s. Skipped by participant

-----------------------------------------------------------------------------------------------
bl_310k_f                                    310: Ever used: Lactational amenorrhea method
-----------------------------------------------------------------------------------------------

                 type:  numeric (byte)
                label:  bl_310k_f

                range:  [0,1]                        units:  1
        unique values:  2                        missing .:  0/4168
      unique mv codes:  4                       missing .*:  3375/4168

           tabulation:  Freq.   Numeric  Label
                          511         0  0. No
                          282         1  1. Yes
                         1340        .a  .a. Never heard of method
                            1        .e  .e. Skip error: Males who
                                         answered 0,2,88 on 307 skipped
                                         to 400
```

```
                    2033        .g  .g. Gender skip pattern
                       1        .s  .s. Skipped by participant

--------------------------------------------------------------------------------
bl_310l                                           310: Ever used: Rhythm method
--------------------------------------------------------------------------------

                  type:  numeric (byte)
                 label:  bl_310l

                 range:  [0,1]                        units:  1
         unique values:  2                        missing .:  0/4168
       unique mv codes:  4                       missing .*:  1946/4168

            tabulation:  Freq.   Numeric  Label
                          1165         0  0. No
                          1057         1  1. Yes
                          1422        .a  .a. Never heard of method
                           509        .e  .e. Skip error: Males who
                                          answered 0,2,88 on 307 skipped
                                          to 400
                             7        .m  .m. Missing
                             8        .s  .s. Skipped by participant

--------------------------------------------------------------------------------
bl_310m                                              310: Ever used: Withdrawal
--------------------------------------------------------------------------------

                  type:  numeric (byte)
                 label:  bl_310m

                 range:  [0,1]                        units:  1
         unique values:  2                        missing .:  0/4168
       unique mv codes:  3                       missing .*:  1243/4168

            tabulation:  Freq.   Numeric  Label
                          1089         0  0. No
                          1836         1  1. Yes
                           733        .a  .a. Never heard of method
                           509        .e  .e. Skip error: Males who
                                          answered 0,2,88 on 307 skipped
                                          to 400
                             1        .s  .s. Skipped by participant

--------------------------------------------------------------------------------
bl_310n                                            310: Ever used: Other method
--------------------------------------------------------------------------------

                  type:  numeric (byte)
                 label:  bl_310n

                 range:  [0,1]                        units:  1
         unique values:  2                        missing .:  0/4168
       unique mv codes:  3                       missing .*:  3661/4168

            tabulation:  Freq.   Numeric  Label
                           323         0  0. No
                           184         1  1. Yes
                          3151        .a  .a. Never heard of method
                           509        .e  .e. Skip error: Males who
                                          answered 0,2,88 on 307 skipped
                                          to 400
                             1        .s  .s. Skipped by participant

--------------------------------------------------------------------------------
bl_311                                 311: Currently doing something to avoid pregnancy
--------------------------------------------------------------------------------

                  type:  numeric (byte)
                 label:  bl_311
```

```
             range:  [0,1]                        units:  1
     unique values:  2                        missing .:  0/4168
   unique mv codes:  6                       missing .*:  800/4168

        tabulation:  Freq.   Numeric  Label
                      1475         0  0. No
                      1893         1  1. Yes
                        55        .a  .a. Can't have children
                       127        .b  .b. Currently pregnant
                        85        .c  .c. Wants children now
                       458        .e  .e. Skip error: Males who
                                      answered 0,2,88 on 307 skipped
                                      to 400
                        30        .m  .m. Missing
                        45        .s  .s. Skipped by participant

bl_311:
  1.  29 cases made NO who originally reported YES to bl_311, but said NO to all methods in
      bl_313.

-----------------------------------------------------------------------------------------------
bl_312                                               312: Know place to get family planning
-----------------------------------------------------------------------------------------------

              type:  numeric (byte)
             label:  bl_312

             range:  [0,1]                        units:  1
     unique values:  2                        missing .:  0/4168
   unique mv codes:  3                       missing .*:  2431/4168

        tabulation:  Freq.   Numeric  Label
                       382         0  0. No
                      1355         1  1. Yes
                      1893        .a  .a. Currently using method to
                                      delay pregnancy
                       509        .e  .e. Skip error: Males who
                                      answered 0,2,88 on 307 skipped
                                      to 400
                        29        .m  .m. Missing

-----------------------------------------------------------------------------------------------
bl_313a                                          313: Currently using: Female sterilization
-----------------------------------------------------------------------------------------------

              type:  numeric (byte)
             label:  bl_313a

             range:  [0,1]                        units:  1
     unique values:  2                        missing .:  0/4168
   unique mv codes:  7                       missing .*:  2277/4168

        tabulation:  Freq.   Numeric  Label
                      1884         0  0. No
                         7         1  1. Yes
                        55        .a  .a. Can't have children
                       127        .b  .b. Currently pregnant
                        85        .c  .c. Wants children now
                      1475        .d  .d. Not currently using family
                                      planning
                       458        .e  .e. Skip error: Males who
                                      answered 0,2,88 on 307 skipped
                                      to 400
                        32        .m  .m. Missing
                        45        .s  .s. Skipped by participant

-----------------------------------------------------------------------------------------------
bl_313b                                            313: Currently using: Male sterilization
-----------------------------------------------------------------------------------------------
```

```
               type:  numeric (byte)
              label:  bl_313b

              range:  [0,1]                        units:  1
      unique values:  2                        missing .:  0/4168
    unique mv codes:  7                       missing .*:  2277/4168

         tabulation:  Freq.   Numeric  Label
                       1887         0  0. No
                          4         1  1. Yes
                         55        .a  .a. Can't have children
                        127        .b  .b. Currently pregnant
                         85        .c  .c. Wants children now
                       1475        .d  .d. Not currently using family
                                       planning
                        458        .e  .e. Skip error: Males who
                                       answered 0,2,88 on 307 skipped
                                       to 400
                         32        .m  .m. Missing
                         45        .s  .s. Skipped by participant

-----------------------------------------------------------------------------------------------
bl_313c                                                          313: Currently using: Pill
-----------------------------------------------------------------------------------------------

               type:  numeric (byte)
              label:  bl_313c

              range:  [0,1]                        units:  1
      unique values:  2                        missing .:  0/4168
    unique mv codes:  7                       missing .*:  2277/4168

         tabulation:  Freq.   Numeric  Label
                       1574         0  0. No
                        317         1  1. Yes
                         55        .a  .a. Can't have children
                        127        .b  .b. Currently pregnant
                         85        .c  .c. Wants children now
                       1475        .d  .d. Not currently using family
                                       planning
                        458        .e  .e. Skip error: Males who
                                       answered 0,2,88 on 307 skipped
                                       to 400
                         32        .m  .m. Missing
                         45        .s  .s. Skipped by participant

-----------------------------------------------------------------------------------------------
bl_313d                                                           313: Currently using: IUD
-----------------------------------------------------------------------------------------------

               type:  numeric (byte)
              label:  bl_313d

              range:  [0,1]                        units:  1
      unique values:  2                        missing .:  0/4168
    unique mv codes:  7                       missing .*:  2277/4168

         tabulation:  Freq.   Numeric  Label
                       1849         0  0. No
                         42         1  1. Yes
                         55        .a  .a. Can't have children
                        127        .b  .b. Currently pregnant
                         85        .c  .c. Wants children now
                       1475        .d  .d. Not currently using family
                                       planning
                        458        .e  .e. Skip error: Males who
                                       answered 0,2,88 on 307 skipped
                                       to 400
                         32        .m  .m. Missing
```

```
                        45        .s  .s. Skipped by participant

-------------------------------------------------------------------------------------------------
bl_313e                                                    313: Currently using: Injectables
-------------------------------------------------------------------------------------------------

                  type:  numeric (byte)
                 label:  bl_313e

                 range:  [0,1]                        units:  1
         unique values:  2                        missing .:  0/4168
       unique mv codes:  7                       missing .*:  2277/4168

            tabulation:  Freq.   Numeric  Label
                          1551         0  0. No
                           340         1  1. Yes
                            55        .a  .a. Can't have children
                           127        .b  .b. Currently pregnant
                            85        .c  .c. Wants children now
                          1475        .d  .d. Not currently using family
                                              planning
                           458        .e  .e. Skip error: Males who
                                              answered 0,2,88 on 307 skipped
                                              to 400
                            32        .m  .m. Missing
                            45        .s  .s. Skipped by participant

-------------------------------------------------------------------------------------------------
bl_313f                                                       313: Currently using: Implants
-------------------------------------------------------------------------------------------------

                  type:  numeric (byte)
                 label:  bl_313f

                 range:  [0,1]                        units:  1
         unique values:  2                        missing .:  0/4168
       unique mv codes:  7                       missing .*:  2277/4168

            tabulation:  Freq.   Numeric  Label
                          1726         0  0. No
                           165         1  1. Yes
                            55        .a  .a. Can't have children
                           127        .b  .b. Currently pregnant
                            85        .c  .c. Wants children now
                          1475        .d  .d. Not currently using family
                                              planning
                           458        .e  .e. Skip error: Males who
                                              answered 0,2,88 on 307 skipped
                                              to 400
                            32        .m  .m. Missing
                            45        .s  .s. Skipped by participant

-------------------------------------------------------------------------------------------------
bl_313g                                                    313: Currently using: Male condom
-------------------------------------------------------------------------------------------------

                  type:  numeric (byte)
                 label:  bl_313g

                 range:  [0,1]                        units:  1
         unique values:  2                        missing .:  0/4168
       unique mv codes:  7                       missing .*:  2277/4168

            tabulation:  Freq.   Numeric  Label
                           891         0  0. No
                          1000         1  1. Yes
                            55        .a  .a. Can't have children
                           127        .b  .b. Currently pregnant
                            85        .c  .c. Wants children now
                          1475        .d  .d. Not currently using family
```

```
                                      planning
                        458        .e  .e. Skip error: Males who
                                      answered 0,2,88 on 307 skipped
                                      to 400
                         32        .m  .m. Missing
                         45        .s  .s. Skipped by participant

-------------------------------------------------------------------------------
bl_313h                                   313: Currently using: Female condom
-------------------------------------------------------------------------------

                  type:  numeric (byte)
                 label:  bl_313h

                 range:  [0,1]                        units:  1
         unique values:  2                        missing .:  0/4168
       unique mv codes:  7                       missing .*:  2277/4168

            tabulation:  Freq.   Numeric  Label
                          1842         0  0. No
                            49         1  1. Yes
                            55        .a  .a. Can't have children
                           127        .b  .b. Currently pregnant
                            85        .c  .c. Wants children now
                          1475        .d  .d. Not currently using family
                                          planning
                           458        .e  .e. Skip error: Males who
                                          answered 0,2,88 on 307 skipped
                                          to 400
                            32        .m  .m. Missing
                            45        .s  .s. Skipped by participant

-------------------------------------------------------------------------------
bl_313i                                       313: Currently using: Diaphragm
-------------------------------------------------------------------------------

                  type:  numeric (byte)
                 label:  bl_313i

                 range:  [0,1]                        units:  1
         unique values:  2                        missing .:  0/4168
       unique mv codes:  7                       missing .*:  2277/4168

            tabulation:  Freq.   Numeric  Label
                          1890         0  0. No
                             1         1  1. Yes
                            55        .a  .a. Can't have children
                           127        .b  .b. Currently pregnant
                            85        .c  .c. Wants children now
                          1475        .d  .d. Not currently using family
                                          planning
                           458        .e  .e. Skip error: Males who
                                          answered 0,2,88 on 307 skipped
                                          to 400
                            32        .m  .m. Missing
                            45        .s  .s. Skipped by participant

-------------------------------------------------------------------------------
bl_313j                                      313: Currently using: Foam/Jelly
-------------------------------------------------------------------------------

                  type:  numeric (byte)
                 label:  bl_313j

                 range:  [0,1]                        units:  1
         unique values:  2                        missing .:  0/4168
       unique mv codes:  7                       missing .*:  2277/4168

            tabulation:  Freq.   Numeric  Label
                          1890         0  0. No
```

```
                      1         1  1. Yes
                     55        .a  .a. Can't have children
                    127        .b  .b. Currently pregnant
                     85        .c  .c. Wants children now
                   1475        .d  .d. Not currently using family
                                       planning
                    458        .e  .e. Skip error: Males who
                                       answered 0,2,88 on 307 skipped
                                       to 400
                     32        .m  .m. Missing
                     45        .s  .s. Skipped by participant
```

```
--------------------------------------------------------------------------------
bl_313k                                313: Ever used: Lactational amenorrhea method
--------------------------------------------------------------------------------

                  type:  numeric (byte)
                 label:  bl_313k

                 range:  [0,1]                        units:  1
         unique values:  2                        missing .:  0/4168
       unique mv codes:  7                       missing .*:  2277/4168

            tabulation:  Freq.   Numeric  Label
                          1879         0  0. No
                            12         1  1. Yes
                            55        .a  .a. Can't have children
                           127        .b  .b. Currently pregnant
                            85        .c  .c. Wants children now
                          1475        .d  .d. Not currently using family
                                              planning
                           458        .e  .e. Skip error: Males who
                                              answered 0,2,88 on 307 skipped
                                              to 400
                            32        .m  .m. Missing
                            45        .s  .s. Skipped by participant
```

```
--------------------------------------------------------------------------------
bl_313l                                           313: Ever used: Rhythm method
--------------------------------------------------------------------------------

                  type:  numeric (byte)
                 label:  bl_313l

                 range:  [0,1]                        units:  1
         unique values:  2                        missing .:  0/4168
       unique mv codes:  7                       missing .*:  2277/4168

            tabulation:  Freq.   Numeric  Label
                          1767         0  0. No
                           124         1  1. Yes
                            55        .a  .a. Can't have children
                           127        .b  .b. Currently pregnant
                            85        .c  .c. Wants children now
                          1475        .d  .d. Not currently using family
                                              planning
                           458        .e  .e. Skip error: Males who
                                              answered 0,2,88 on 307 skipped
                                              to 400
                            32        .m  .m. Missing
                            45        .s  .s. Skipped by participant
```

```
--------------------------------------------------------------------------------
bl_313m                                              313: Ever used: Withdrawal
--------------------------------------------------------------------------------

                  type:  numeric (byte)
                 label:  bl_313m

                 range:  [0,1]                        units:  1
```

```
        unique values:  2                        missing .:  0/4168
      unique mv codes:  7                       missing .*:  2277/4168

           tabulation:  Freq.   Numeric  Label
                         1666         0  0. No
                          225         1  1. Yes
                           55        .a  .a. Can't have children
                          127        .b  .b. Currently pregnant
                           85        .c  .c. Wants children now
                         1475        .d  .d. Not currently using family
                                            planning
                          458        .e  .e. Skip error: Males who
                                            answered 0,2,88 on 307 skipped
                                            to 400
                           32        .m  .m. Missing
                           45        .s  .s. Skipped by participant

-----------------------------------------------------------------------------------------------
bl_313n                                                       313: Ever used: Other method
-----------------------------------------------------------------------------------------------

                 type:  numeric (byte)
                label:  bl_313n

                range:  [0,1]                        units:  1
        unique values:  2                        missing .:  0/4168
      unique mv codes:  7                       missing .*:  2277/4168

           tabulation:  Freq.   Numeric  Label
                         1860         0  0. No
                           31         1  1. Yes
                           55        .a  .a. Can't have children
                          127        .b  .b. Currently pregnant
                           85        .c  .c. Wants children now
                         1475        .d  .d. Not currently using family
                                            planning
                          458        .e  .e. Skip error: Males who
                                            answered 0,2,88 on 307 skipped
                                            to 400
                           32        .m  .m. Missing
                           45        .s  .s. Skipped by participant

-----------------------------------------------------------------------------------------------
bl_314_f                                        314: Past month, how many days forgot pill
-----------------------------------------------------------------------------------------------

                 type:  numeric (byte)
                label:  bl_314, but 11 nonmissing values are not labeled

                range:  [0,88]                       units:  1
        unique values:  12                       missing .:  0/4168
      unique mv codes:  9                       missing .*:  3949/4168

           tabulation:  Freq.   Numeric  Label
                          141         0
                           17         1
                           21         2
                           11         3
                            6         4
                            3         5
                            3         6
                            6         7
                            1        14
                            1        20
                            2        30
                            7        88  88. Don't Know
                           35        .a  .a. Can't have children
                           45        .b  .b. Currently pregnant
                           54        .c  .c. Wants children now
                          971        .d  .d. Not currently using family
```

```
                                       planning
                    1        .e  .e. Skip pattern error
                  808        .f  .f. Not using pill: Using other
                                     method of family planning
                 2033        .g  .g. Gender skip pattern
                    1        .m  .m. Missing
                    1        .s  .s. Skipped by participant

-----------------------------------------------------------------------------------------------
bl_315                                    315: Past month, how many times sex without condom
-----------------------------------------------------------------------------------------------

                  type:  numeric (byte)
                 label:  bl_315, but 8 nonmissing values are not labeled

                 range:  [0,88]                       units:  1
         unique values:  9                        missing .:  0/4168
       unique mv codes:  7                       missing .*:  4124/4168

            tabulation:  Freq.   Numeric  Label
                            13         0
                            10         1
                             7         2
                             5         3
                             1         4
                             1         6
                             2         7
                             1        14
                             4        88  88. Don't Know
                            55        .a  .a. Can't have children
                           127        .b  .b. Currently pregnant
                            85        .c  .c. Wants children now
                          1475        .d  .d. Not currently using family
                                          planning
                           458        .e  .e. Skip pattern error
                          1849        .f  .f. Not using condom: Using
                                          other method of family planning
                            75        .m  .m. Missing

-----------------------------------------------------------------------------------------------
bl_400                                                    400: Perceived risk of getting HIV
-----------------------------------------------------------------------------------------------

                  type:  numeric (byte)
                 label:  bl_400

                 range:  [0,88]                       units:  1
         unique values:  6                        missing .:  0/4168
       unique mv codes:  1                       missing .*:  7/4168

            tabulation:  Freq.   Numeric  Label
                           880         0  0. No risk
                           942         1  1. Low
                           895         2  2. Moderate
                           970         3  3. High
                            82         4  4. HIV+
                           392        88  88. Don't know
                             7        .s  .s. Skipped by participant

-----------------------------------------------------------------------------------------------
bl_401                                                              401: Ever tested for HIV
-----------------------------------------------------------------------------------------------

                  type:  numeric (byte)
                 label:  yesno_bl

                 range:  [0,1]                        units:  1
         unique values:  2                        missing .:  0/4168

            tabulation:  Freq.   Numeric  Label
```

```
                        632         0  0. No
                       3536         1  1. Yes

-------------------------------------------------------------------------------
bl_402                                       402: Received results for last HIV test
-------------------------------------------------------------------------------

                  type:  numeric (byte)
                 label:  bl_402

                 range:  [0,1]                        units:  1
         unique values:  2                        missing .:  0/4168
       unique mv codes:  2                       missing .*:  633/4168

            tabulation:  Freq.   Numeric  Label
                            65         0  0. No
                          3470         1  1. Yes
                           632        .a  .a. Never been tested for HIV
                             1        .s  .s. Skipped by participant

-------------------------------------------------------------------------------
bl_403                                               403: Last time tested for HIV
-------------------------------------------------------------------------------

                  type:  numeric (byte)
                 label:  bl_403

                 range:  [1,88]                       units:  1
         unique values:  5                        missing .:  0/4168
       unique mv codes:  2                       missing .*:  637/4168

            tabulation:  Freq.   Numeric  Label
                          1623         1  1. <6 months ago
                           821         2  2. 6-11 months ago
                           477         3  3. 12-23 months ago
                           507         4  4. 2+ years ago
                           103        88  88. Don't remember
                           632        .a  .a. Never been tested for HIV
                             5        .s  .s. Skipped by participant

-------------------------------------------------------------------------------
bl_404                          404: Ever tested with spouse/current partner for HIV
-------------------------------------------------------------------------------

                  type:  numeric (byte)
                 label:  bl_404

                 range:  [0,1]                        units:  1
         unique values:  2                        missing .:  0/4168
       unique mv codes:  3                       missing .*:  1179/4168

            tabulation:  Freq.   Numeric  Label
                          1452         0  0. No
                          1537         1  1. Yes
                           468        .a  .a. Never been tested for HIV
                           705        .b  .b. No primary sex partner
                             6        .s  .s. Skipped by participant

-------------------------------------------------------------------------------
bl_405                      405: Last time tested with spouse/current partner for HIV
-------------------------------------------------------------------------------

                  type:  numeric (byte)
                 label:  bl_405

                 range:  [1,88]                       units:  1
         unique values:  5                        missing .:  0/4168
       unique mv codes:  4                       missing .*:  2633/4168

            tabulation:  Freq.   Numeric  Label
```

```
                        594         1  1. <6 months ago
                        420         2  2. 6-11 months ago
                        225         3  3. 12-23 months ago
                        233         4  4. 2+ years ago
                         63        88  88. Don't remember
                        468        .a  .a. Never been tested for HIV
                        705        .b  .b. No primary sex partner
                       1452        .c  .c. Never been tested with
                                       partner
                          8        .s  .s. Skipped by participant

-------------------------------------------------------------------------------
bl_406                                 406: Both received results for last HIV test
-------------------------------------------------------------------------------

                  type:  numeric (byte)
                 label:  bl_406

                 range:  [0,1]                        units:  1
         unique values:  2                        missing .:  0/4168
       unique mv codes:  4                       missing .*:  2632/4168

            tabulation:  Freq.   Numeric  Label
                         40         0  0. No
                       1496         1  1. Yes
                        468        .a  .a. Never been tested for HIV
                        705        .b  .b. No primary sex partner
                       1452        .c  .c. Never been tested with
                                       partner
                          7        .s  .s. Skipped by participant

-------------------------------------------------------------------------------
bl_407                          407: Self/Partner ever screened for cervical cancer
-------------------------------------------------------------------------------

                  type:  numeric (byte)
                 label:  bl_407

                 range:  [0,88]                       units:  1
         unique values:  3                        missing .:  0/4168
       unique mv codes:  3                       missing .*:  2508/4168

            tabulation:  Freq.   Numeric  Label
                       1264         0  0. No
                        335         1  1. Yes
                         61        88  88. Don't Know
                        479        .b  .b. Males: No primary sex
                                       partner
                       2024        .e  .e. Skip error: Programming
                                       error (No on 401 or 404: skip to
                                       500)
                          5        .s  .s. Skipped by participant

-------------------------------------------------------------------------------
bl_408                                  408: Last time screened for cervical cancer
-------------------------------------------------------------------------------

                  type:  numeric (byte)
                 label:  bl_408

                 range:  [1,88]                       units:  1
         unique values:  3                        missing .:  0/4168
       unique mv codes:  4                       missing .*:  3785/4168

            tabulation:  Freq.   Numeric  Label
                        257         1  1. <2 years ago
                         67         2  2. 2+ years ago
                         59        88  88. Don't know
                       1173        .a  .a. Never been screened for
                                       cervical cancer
```

```
                     479        .b  .b. Males: No primary sex
                                    partner
                    2115        .e  .e. Skip error: Programming
                                    error (No on 401 or 404: skip to
                                    500)
                      18        .s  .s. Skipped by participant

-------------------------------------------------------------------------------------------
bl_500                                                             500: Ever heard of AIDS
-------------------------------------------------------------------------------------------

                  type:  numeric (byte)
                 label:  yesno_bl

                 range:  [0,1]                        units:  1
         unique values:  2                        missing .:  0/4168

           tabulation:  Freq.   Numeric  Label
                          140         0  0. No
                         4028         1  1. Yes

-------------------------------------------------------------------------------------------
bl_501                        501: Can reduce risk of HIV by having one uninfected sex partner
-------------------------------------------------------------------------------------------

                  type:  numeric (byte)
                 label:  bl_501

                 range:  [0,88]                       units:  1
         unique values:  3                        missing .:  0/4168
       unique mv codes:  2                       missing .*:  141/4168

           tabulation:  Freq.   Numeric  Label
                          442         0  0. No
                         3512         1  1. Yes
                           73        88  88. Don't Know
                          140        .a  .a. Never heard of AIDS
                            1        .s  .s. Skipped by participant

-------------------------------------------------------------------------------------------
bl_502                                                 502: Can get HIV from mosquito bites
-------------------------------------------------------------------------------------------

                  type:  numeric (byte)
                 label:  bl_502

                 range:  [0,88]                       units:  1
         unique values:  3                        missing .:  0/4168
       unique mv codes:  2                       missing .*:  141/4168

           tabulation:  Freq.   Numeric  Label
                         3033         0  0. No
                          764         1  1. Yes
                          230        88  88. Don't Know
                          140        .a  .a. Never heard of AIDS
                            1        .s  .s. Skipped by participant

-------------------------------------------------------------------------------------------
bl_503                                       503: Can reduce risk by using condom every time
-------------------------------------------------------------------------------------------

                  type:  numeric (byte)
                 label:  bl_503

                 range:  [0,88]                       units:  1
         unique values:  3                        missing .:  0/4168
       unique mv codes:  2                       missing .*:  141/4168

           tabulation:  Freq.   Numeric  Label
                          687         0  0. No
```

```
                     3278         1  1. Yes
                       62        88  88. Don't Know
                      140        .a  .a. Never heard of AIDS
                        1        .s  .s. Skipped by participant

-------------------------------------------------------------------------------------------
bl_504                              504: Can get HIV by sharing food with someone with AIDS
-------------------------------------------------------------------------------------------

                  type:  numeric (byte)
                 label:  bl_504

                 range:  [0,88]                       units:  1
         unique values:  3                        missing .:  0/4168
       unique mv codes:  1                       missing .*:  140/4168

            tabulation:  Freq.   Numeric  Label
                          3781         0  0. No
                           188         1  1. Yes
                            59        88  88. Don't Know
                           140        .a  .a. Never heard of AIDS

-------------------------------------------------------------------------------------------
bl_505                                        505: Can reduce risk of HIV by not having sex
-------------------------------------------------------------------------------------------

                  type:  numeric (byte)
                 label:  bl_505

                 range:  [0,88]                       units:  1
         unique values:  3                        missing .:  0/4168
       unique mv codes:  1                       missing .*:  140/4168

            tabulation:  Freq.   Numeric  Label
                           890         0  0. No
                          3103         1  1. Yes
                            35        88  88. Don't Know
                           140        .a  .a. Never heard of AIDS

-------------------------------------------------------------------------------------------
bl_506                           506: Can get HIV by witchcraft or other supernatural means
-------------------------------------------------------------------------------------------

                  type:  numeric (byte)
                 label:  bl_506

                 range:  [0,88]                       units:  1
         unique values:  3                        missing .:  0/4168
       unique mv codes:  2                       missing .*:  142/4168

            tabulation:  Freq.   Numeric  Label
                          3621         0  0. No
                           300         1  1. Yes
                           105        88  88. Don't Know
                           140        .a  .a. Never heard of AIDS
                             2        .s  .s. Skipped by participant

-------------------------------------------------------------------------------------------
bl_507                                 507: Possible for healthy-looking person to have HIV
-------------------------------------------------------------------------------------------

                  type:  numeric (byte)
                 label:  bl_507

                 range:  [0,88]                       units:  1
         unique values:  3                        missing .:  0/4168
       unique mv codes:  2                       missing .*:  141/4168

            tabulation:  Freq.   Numeric  Label
                           468         0  0. No
```

```
                      3528         1  1. Yes
                        31        88  88. Don't Know
                       140        .a  .a. Never heard of AIDS
                         1        .s  .s. Skipped by participant

-------------------------------------------------------------------------------------------
bl_508a          508: Pregnancy: Can virus that causes AIDS be transmitted from mother to baby
-------------------------------------------------------------------------------------------

                  type:  numeric (byte)
                 label:  bl_508a

                 range:  [0,88]                      units:  1
         unique values:  3                       missing .:  0/4168
       unique mv codes:  2                      missing .*:  142/4168

            tabulation:  Freq.   Numeric  Label
                          1405         0  0. No
                          2368         1  1. Yes
                           253        88  88. Don't Know
                           140        .a  .a. Never heard of AIDS
                             2        .s  .s. Skipped by participant

-------------------------------------------------------------------------------------------
bl_508b           508: Delivery: Can virus that causes AIDS be transmitted from mother to baby
-------------------------------------------------------------------------------------------

                  type:  numeric (byte)
                 label:  bl_508b

                 range:  [0,88]                      units:  1
         unique values:  3                       missing .:  0/4168
       unique mv codes:  2                      missing .*:  142/4168

            tabulation:  Freq.   Numeric  Label
                           325         0  0. No
                          3530         1  1. Yes
                           171        88  88. Don't Know
                           140        .a  .a. Never heard of AIDS
                             2        .s  .s. Skipped by participant

-------------------------------------------------------------------------------------------
bl_508c       508: Breastfeeding: Can virus that causes AIDS be transmitted from mother to bab
-------------------------------------------------------------------------------------------

                  type:  numeric (byte)
                 label:  bl_508c

                 range:  [0,88]                      units:  1
         unique values:  3                       missing .:  0/4168
       unique mv codes:  2                      missing .*:  142/4168

            tabulation:  Freq.   Numeric  Label
                           255         0  0. No
                          3596         1  1. Yes
                           175        88  88. Don't Know
                           140        .a  .a. Never heard of AIDS
                             2        .s  .s. Skipped by participant

-------------------------------------------------------------------------------------------
bl_600                                                  600: Male circumcised: Self or Partner
-------------------------------------------------------------------------------------------

                  type:  numeric (byte)
                 label:  bl_600

                 range:  [0,88]                      units:  1
         unique values:  3                       missing .:  0/4168
       unique mv codes:  3                      missing .*:  231/4168
```

```
          tabulation:  Freq.   Numeric  Label
                        2648         0  0. No
                        1260         1  1. Yes
                          29        88  88. Don't Know
                         226        .b  .b. Females: No primary sex
                                        partner
                           2        .m  .m. Missing
                           3        .s  .s. Skipped by participant

-----------------------------------------------------------------------------
bl_601                                           601: Male circumcised: When
-----------------------------------------------------------------------------

                type:  numeric (byte)
               label:  bl_601

               range:  [1,88]                       units:  1
       unique values:  3                        missing .:  0/4168
     unique mv codes:  4                       missing .*:  2913/4168

          tabulation:  Freq.   Numeric  Label
                         357         1  1. <2 yrs ago
                         657         2  2.  >2 yrs ago
                         241        88  88. Don't Know
                        2677        .a  .a. Uncircumcised
                         226        .b  .b. Females: No primary sex
                                        partner
                           1        .m  .m. Missing
                           9        .s  .s. Skipped by participant

-----------------------------------------------------------------------------
bl_602                                    602: Male circumcised: At what age
-----------------------------------------------------------------------------

                type:  numeric (byte)
               label:  bl_602, but 50 nonmissing values are not labeled

               range:  [1,88]                       units:  1
       unique values:  51                       missing .:  0/4168
     unique mv codes:  4                       missing .*:  2988/4168

            examples:  26
                       .a    .a. Uncircumcised
                       .a    .a. Uncircumcised
                       .a    .a. Uncircumcised

-----------------------------------------------------------------------------
bl_603                        603: Male circumcised: Who performed circumcision
-----------------------------------------------------------------------------

                type:  numeric (byte)
               label:  bl_603

               range:  [1,88]                       units:  1
       unique values:  3                        missing .:  0/4168
     unique mv codes:  4                       missing .*:  2914/4168

          tabulation:  Freq.   Numeric  Label
                         967         1  1. Health professional
                         145         2  2. Traditional circumciser
                         142        88  88. Don't Know
                        2677        .a  .a. Uncircumcised
                         226        .b  .b. Females: No primary sex
                                        partner
                           2        .m  .m. Missing
                           9        .s  .s. Skipped by participant

-----------------------------------------------------------------------------
bl_604                                           604: MC's effect on HIV risk
-----------------------------------------------------------------------------
```

```
                  type:  numeric (byte)
                 label:  bl_604

                 range:  [1,88]                       units:  1
         unique values:  4                        missing .:  0/4168
       unique mv codes:  2                       missing .*:  3/4168

            tabulation:  Freq.   Numeric  Label
                            74         1  1. Increases risk
                          3673         2  2. Reduces risk
                           199         3  3. Has no effect
                           219        88  88. Don't Know
                             2        .m  .m. Missing
                             1        .s  .s. Skipped by participant

-----------------------------------------------------------------------------------------------
bl_605                                                              605: MC's effect on STD risk
-----------------------------------------------------------------------------------------------

                  type:  numeric (byte)
                 label:  bl_605

                 range:  [1,88]                       units:  1
         unique values:  4                        missing .:  0/4168
       unique mv codes:  2                       missing .*:  3/4168

            tabulation:  Freq.   Numeric  Label
                            94         1  1. Increases risk
                          3693         2  2. Reduces risk
                           146         3  3. Has no effect
                           232        88  88. Don't Know
                             1        .m  .m. Missing
                             2        .s  .s. Skipped by participant

-----------------------------------------------------------------------------------------------
bl_606                                                   606: Know anyone who has had medical MC
-----------------------------------------------------------------------------------------------

                  type:  numeric (byte)
                 label:  yesno_bl

                 range:  [0,1]                        units:  1
         unique values:  2                        missing .:  0/4168
       unique mv codes:  2                       missing .*:  4/4168

            tabulation:  Freq.   Numeric  Label
                          1159         0  0. No
                          3005         1  1. Yes
                             1        .m  .m. Missing
                             3        .s  .s. Skipped by participant

-----------------------------------------------------------------------------------------------
bl_607a                                                         607: Male circumcised: Father
-----------------------------------------------------------------------------------------------

                  type:  numeric (byte)
                 label:  bl_607a

                 range:  [0,1]                        units:  1
         unique values:  2                        missing .:  0/4168
       unique mv codes:  3                       missing .*:  1170/4168

            tabulation:  Freq.   Numeric  Label
                          2946         0  0. No
                            52         1  1. Yes
                          1159        .a  .a. Don't know anyone who has
                                          undergone medical MC
                             1        .m  .m. Missing
                            10        .s  .s. Skipped by participant
```

```
--------------------------------------------------------------------------------------------
bl_607b                                                        607: Male circumcised: Son
--------------------------------------------------------------------------------------------

                  type:  numeric (byte)
                 label:  bl_607b

                 range:  [0,1]                        units:  1
         unique values:  2                        missing .:  0/4168
       unique mv codes:  3                       missing .*:  1170/4168

            tabulation:  Freq.   Numeric  Label
                          2722         0  0. No
                           276         1  1. Yes
                          1159        .a  .a. Don't know anyone who has
                                          undergone medical MC
                             1        .m  .m. Missing
                            10        .s  .s. Skipped by participant

--------------------------------------------------------------------------------------------
bl_607c                                                    607: Male circumcised: Brother
--------------------------------------------------------------------------------------------

                  type:  numeric (byte)
                 label:  bl_607c

                 range:  [0,1]                        units:  1
         unique values:  2                        missing .:  0/4168
       unique mv codes:  3                       missing .*:  1170/4168

            tabulation:  Freq.   Numeric  Label
                          2167         0  0. No
                           831         1  1. Yes
                          1159        .a  .a. Don't know anyone who has
                                          undergone medical MC
                             1        .m  .m. Missing
                            10        .s  .s. Skipped by participant

--------------------------------------------------------------------------------------------
bl_607d                                        607: Male circumcised: Other male relative
--------------------------------------------------------------------------------------------

                  type:  numeric (byte)
                 label:  bl_607d

                 range:  [0,1]                        units:  1
         unique values:  2                        missing .:  0/4168
       unique mv codes:  3                       missing .*:  1170/4168

            tabulation:  Freq.   Numeric  Label
                          1914         0  0. No
                          1084         1  1. Yes
                          1159        .a  .a. Don't know anyone who has
                                          undergone medical MC
                             1        .m  .m. Missing
                            10        .s  .s. Skipped by participant

--------------------------------------------------------------------------------------------
bl_607e                                                607: Male circumcised: Male friend
--------------------------------------------------------------------------------------------

                  type:  numeric (byte)
                 label:  bl_607e

                 range:  [0,1]                        units:  1
         unique values:  2                        missing .:  0/4168
       unique mv codes:  3                       missing .*:  1170/4168

            tabulation:  Freq.   Numeric  Label
```

```
                        1119         0  0. No
                        1879         1  1. Yes
                        1159        .a  .a. Don't know anyone who has
                                        undergone medical MC
                           1        .m  .m. Missing
                          10        .s  .s. Skipped by participant

-------------------------------------------------------------------------------
bl_607f                                 607: Male circumcised: Other male acquaintance
-------------------------------------------------------------------------------

                  type:  numeric (byte)
                 label:  bl_607f

                 range:  [0,1]                        units:  1
         unique values:  2                        missing .:  0/4168
       unique mv codes:  3                       missing .*:  1170/4168

            tabulation:  Freq.   Numeric  Label
                        2098         0  0. No
                         900         1  1. Yes
                        1159        .a  .a. Don't know anyone who has
                                        undergone medical MC
                           1        .m  .m. Missing
                          10        .s  .s. Skipped by participant

-------------------------------------------------------------------------------
bl_608                              608: Considered male circumcision: Self or Partner
-------------------------------------------------------------------------------

                  type:  numeric (byte)
                 label:  bl_608

                 range:  [0,1]                        units:  1
         unique values:  2                        missing .:  0/4168
       unique mv codes:  4                       missing .*:  1503/4168

            tabulation:  Freq.   Numeric  Label
                         773         0  0. No
                        1892         1  1. Yes
                        1260        .a  .a. Self/partner circumcised
                         226        .b  .b. Females: No primary sex
                                        partner
                           2        .m  .m. Missing
                          15        .s  .s. Skipped by participant

-------------------------------------------------------------------------------
bl_609                          609: Main reason have not considered male circumcision
-------------------------------------------------------------------------------

                  type:  numeric (byte)
                 label:  bl_609

                 range:  [1,21]                       units:  1
         unique values:  16                       missing .:  0/4168
       unique mv codes:  6                       missing .*:  3859/4168

            tabulation:  Freq.   Numeric  Label
                           8         1  1. Not at risk: Married
                           5         2  2. Not at risk: One partner
                           2         3  3. Not at risk: Uses condoms
                           8         4  4. Not at risk: Too old
                          32         5  5. Not at risk: Other reason
                           2         6  6. Opposed: Spouse/partner
                           3         7  7. Opposed: Parent/guardian
                           3         8  8. Opposed: Other person
                          95         9  9. Fear: Pain, surgery
                           5        10  10. Fear: Not safe
                          20        11  11. Fear: Complications
                           4        13  13. Time: Off non-work
```

```
                                             activities
                             9        14  14. Time: Off work
                             9        16  16. Time: Other
                             6        20  20. MC not in culture/tradition
                            98        21  21. Other reason
                          1260        .a  .a. Self/partner circumcised
                           226        .b  .b. Females: No primary sex
                                          partner
                          1892        .c  .c. Have considered MC
                           451        .e  .e. Skip error: Females with
                                          uncircumcised partner skipped
                            15        .m  .m. Missing
                            15        .s  .s. Skipped by participant

bl_609:
  1.  Coding error in female survey led to few valid answers.

-----------------------------------------------------------------------------------------------
bl_610                                                              610: Know place offering MC
-----------------------------------------------------------------------------------------------

                  type:  numeric (byte)
                 label:  yesno_bl

                 range:  [0,1]                        units:  1
         unique values:  2                        missing .:  0/4168
       unique mv codes:  2                       missing .*:  10/4168

            tabulation:  Freq.   Numeric  Label
                          1064         0  0. No
                          3094         1  1. Yes
                             2        .m  .m. Missing
                             8        .s  .s. Skipped by participant

-----------------------------------------------------------------------------------------------
bl_611                                    611: Cost of transport to nearest place offering MC
-----------------------------------------------------------------------------------------------

                  type:  numeric (byte)
                 label:  bl_611

                 range:  [0,88]                       units:  1
         unique values:  20                       missing .:  0/4168
       unique mv codes:  3                       missing .*:  1098/4168

            tabulation:  Freq.   Numeric  Label
                           455         0  0. Would not cost anything
                            18         1  1. <1 KR
                            31         2  2. 1 KR
                           186         3  3. 2 KR
                           223         4  4. 3 KR
                           129         5  5. 4 KR
                           358         6  6. 5 KR
                           600         7  7. 6-10 KR
                           250         8  8. 11-15 KR
                           367         9  9. 16-20 KR
                            71        10  10. 21-25 KR
                           108        11  11. 26-30 KR
                            14        12  12. 31-35 KR
                            13        13  13. 36-40 KR
                             9        14  14. 41-45 KR
                            41        15  15. 46-50 KR
                            18        16  16. 51-100 KR
                             6        17  17. 101-200 KR
                             1        19  19. 301-400 KR
                           172        88  88. Don't Know
                          1064        .a  .a. Don't know place offering MC
                             2        .m  .m. Missing
                            32        .s  .s. Skipped by participant
```

```
-------------------------------------------------------------------------------------------
bl_612a                                               612: Minutes to nearest place offering MC
-------------------------------------------------------------------------------------------

                  type:  numeric (byte)
                 label:  bl_612a, but 44 nonmissing values are not labeled

                 range:  [0,88]                       units:  1
         unique values:  45                       missing .:  0/4168
       unique mv codes:  3                       missing .*:  1097/4168

              examples:  10
                         25
                         40
                         .a    .a. Don't know place offering MC

-------------------------------------------------------------------------------------------
bl_612b                                                 612: Hours to nearest place offering MC
-------------------------------------------------------------------------------------------

                  type:  numeric (byte)
                 label:  bl_612b, but 7 nonmissing values are not labeled

                 range:  [0,88]                       units:  1
         unique values:  8                        missing .:  0/4168
       unique mv codes:  3                       missing .*:  1097/4168

            tabulation:  Freq.   Numeric  Label
                          2353         0
                           382         1
                            77         2
                            13         3
                             1         4
                             1         5
                             2         6
                           242        88  88. Don't Know
                          1064        .a  .a. Don't know place offering MC
                             2        .m  .m. Missing
                            31        .s  .s. Skipped by participant

-------------------------------------------------------------------------------------------
bl_700                                           700: Travel time to health facility (minutes)
-------------------------------------------------------------------------------------------

                  type:  numeric (int)
                 label:  nummin, but 81 nonmissing values are not labeled

                 range:  [0,888]                      units:  1
         unique values:  82                       missing .:  0/4168
       unique mv codes:  2                       missing .*:  27/4168

              examples:  15
                         30
                         45
                         60

-------------------------------------------------------------------------------------------
bl_701                                             701: Transportation mode to health facility
-------------------------------------------------------------------------------------------

                  type:  numeric (byte)
                 label:  bl_701

                 range:  [1,7]                        units:  1
         unique values:  7                        missing .:  0/4168
       unique mv codes:  2                       missing .*:  6/4168

            tabulation:  Freq.   Numeric  Label
                          1259         1  1. Bus
                           176         2  2. Taxi
```

109	3	3. Someone drove me in private car
96	4	4. Own car, self-drive
2345	5	5. Walked
163	6	6. Bicycle
14	7	7. Other
2	.m	.m. Missing
4	.s	.s. Skipped by participant

---

bl_702 — 702: Cost of transport to health facility

---

type: numeric (byte)
label: bl_702

range: [0,16] units: 1
unique values: 17 missing .: 0/4168
unique mv codes: 3 missing .*: 2619/4168

tabulation:

Freq.	Numeric	Label
128	0	0. Nothing
9	1	1. <1 KR
47	2	2. 1 KR
284	3	3. 2 KR
253	4	4. 3 KR
190	5	5. 4 KR
298	6	6. 5 KR
185	7	7. 6-10 KR
61	8	8. 11-15 KR
46	9	9. 16-20 KR
17	10	10. 21-25 KR
13	11	11. 26-30 KR
2	12	12. 31-35 KR
2	13	13. 36-40 KR
1	14	14. 41-45 KR
9	15	15. 46-50 KR
4	16	16. 51-100 KR
2604	.a	.a. Own car, walked, or bicycle
2	.m	.m. Missing
13	.s	.s. Skipped by participant

---

bl_703 — 703: Distance travelled to health facility (kilometers)

---

type: numeric (int)
label: bl_703, but 40 nonmissing values are not labeled

range: [0,888] units: 1
unique values: 42 missing .: 0/4168
unique mv codes: 2 missing .*: 38/4168

examples: 1
4
60
888 888. Don't Know

---

bl_704 — 704: Number people who accompanied respondent to health facility today

---

type: numeric (byte)
label: bl_704

range: [0,3] units: 1
unique values: 4 missing .: 0/4168
unique mv codes: 2 missing .*: 3/4168

tabulation: Freq. Numeric Label

```
                   2690         0  0. Came alone
                   1149         1  1. One other person
                    195         2  2. Two other people
                    131         3  3. More than 2 other people
                      2        .m  .m. Missing
                      1        .s  .s. Skipped by participant

-------------------------------------------------------------------------------
bl_705                            705: Relationship of 1st oldest person who came with
-------------------------------------------------------------------------------

                  type:  numeric (byte)
                 label:  bl_705

                 range:  [1,8]                        units:  1
         unique values:  8                        missing .:  0/4168
       unique mv codes:  3                       missing .*:  2693/4168

            tabulation:  Freq.   Numeric  Label
                          140         1  1. Spouse
                           71         2  2. Son/Daughter
                           11         3  3. Parent
                           22         4  4. Uncle/Aunt
                            2         5  5. Grandparent
                            1         6  6. Grandchild
                          401         7  7. Other relative
                          827         8  8. Other non-relative
                         2690        .a  .a. Came alone
                            2        .m  .m. Missing
                            1        .s  .s. Skipped by participant

-------------------------------------------------------------------------------
bl_706                   706: Cost of transport for 1st person, in addition to what R paid
-------------------------------------------------------------------------------

                  type:  numeric (byte)
                 label:  bl_706

                 range:  [0,88]                       units:  1
         unique values:  12                       missing .:  0/4168
       unique mv codes:  4                       missing .*:  3872/4168

            tabulation:  Freq.   Numeric  Label
                          221         0  0. Nothing
                           10         3  3. 2 KR
                           16         4  4. 3 KR
                           10         5  5. 4 KR
                           12         6  6. 5 KR
                            6         7  7. 6-10 KR
                            9         8  8. 11-15 KR
                            3         9  9. 16-20 KR
                            3        10  10. 21-25 KR
                            1        11  11. 26-30 KR
                            1        15  15. 46-50 KR
                            4        88  88. Don't Know
                         2690        .a  .a. Came alone
                         1149        .e  .e. Skip error: Those with 1
                                         other skipped out
                            2        .m  .m. Missing
                           31        .s  .s. Skipped by participant

-------------------------------------------------------------------------------
bl_707                            707: Relationship of 2nd oldest person who came with
-------------------------------------------------------------------------------

                  type:  numeric (byte)
                 label:  bl_707

                 range:  [1,8]                        units:  1
         unique values:  5                        missing .:  0/4168
```

```
      unique mv codes:  5                      missing .*:  3973/4168

           tabulation:  Freq.   Numeric  Label
                            2         1  1. Spouse
                           15         2  2. Son/Daughter
                            2         4  4. Uncle/Aunt
                           57         7  7. Other relative
                          119         8  8. Other non-relative
                         2690        .a  .a. Came alone
                         1149        .b  .b. Came with one person
                          131        .e  .e. Skip error: Those with >2
                                         others skipped out
                            2        .m  .m. Missing
                            1        .s  .s. Skipped by participant

-----------------------------------------------------------------------------------------------
bl_708                       708: Cost of transport for 2nd person, in addition to what R paid
-----------------------------------------------------------------------------------------------

                 type:  numeric (byte)
                label:  bl_708

                range:  [0,88]                         units:  1
        unique values:  9                          missing .:  0/4168
      unique mv codes:  5                         missing .*:  3992/4168

           tabulation:  Freq.   Numeric  Label
                          134         0  0. Nothing
                            6         3  3. 2 KR
                           10         4  4. 3 KR
                            5         5  5. 4 KR
                            8         6  6. 5 KR
                            4         7  7. 6-10 KR
                            4         8  8. 11-15 KR
                            1         9  9. 16-20 KR
                            4        88  88. Don't Know
                         2690        .a  .a. Came alone
                         1149        .b  .b. Came with one person
                          131        .e  .e. Skip error: Those with >2
                                         others skipped out
                            2        .m  .m. Missing
                           20        .s  .s. Skipped by participant

-----------------------------------------------------------------------------------------------
bl_709          709: Source of money used to pay for transport and services at health facility
-----------------------------------------------------------------------------------------------

                 type:  numeric (byte)
                label:  bl_709

                range:  [1,6]                          units:  1
        unique values:  6                          missing .:  0/4168
      unique mv codes:  4                         missing .*:  2746/4168

           tabulation:  Freq.   Numeric  Label
                          155         1  1. Cutting down on other
                                         expenses
                          664         2  2. Savings
                           68         3  3. Borrowing (to pay back)
                           14         4  4. Selling assets
                          451         5  5. Donation from family/friend
                           70         6  6. Other
                         2604        .a  .a. Own car, walked, or bicycle
                          128        .b  .b. Paid nothing for transport
                            2        .m  .m. Missing
                           12        .s  .s. Skipped by participant

-----------------------------------------------------------------------------------------------
bl_710                           710: What respondent would be doing if not at health facility
-----------------------------------------------------------------------------------------------
```

```
                 type:  numeric (byte)
                label:  bl_710

                range:  [1,6]                        units:  1
        unique values:  6                        missing .:  0/4168
      unique mv codes:  2                       missing .*:  11/4168

           tabulation:  Freq.   Numeric  Label
                          621         1  1. Paid employment
                          719         2  2. Own business
                         1043         3  3. Unpaid work at home/Housework
                          351         4  4. In school
                         1139         5  5. Resting/No specific activity
                          284         6  6. Other
                            2        .m  .m. Missing
                            9        .s  .s. Skipped by participant

-----------------------------------------------------------------------------------------------
bl_800                      800: Self-rated health in past YEAR: 1 (very bad) - 10 (very good)
-----------------------------------------------------------------------------------------------

                 type:  numeric (byte)
                label:  num_bl, but 10 nonmissing values are not labeled

                range:  [1,10]                       units:  1
        unique values:  10                       missing .:  0/4168
      unique mv codes:  2                       missing .*:  3/4168

           tabulation:  Freq.   Numeric  Label
                           48         1
                           23         2
                           26         3
                          361         4
                          183         5
                          159         6
                         1042         7
                          490         8
                          507         9
                         1326        10
                            2        .m  .m. Missing
                            1        .s  .s. Skipped by participant

-----------------------------------------------------------------------------------------------
bl_801                     801: Self-rated health in past MONTH: 1 (very bad) - 10 (very good)
-----------------------------------------------------------------------------------------------

                 type:  numeric (byte)
                label:  num_bl, but 10 nonmissing values are not labeled

                range:  [1,10]                       units:  1
        unique values:  10                       missing .:  0/4168
      unique mv codes:  2                       missing .*:  3/4168

           tabulation:  Freq.   Numeric  Label
                           31         1
                           29         2
                           33         3
                          342         4
                          169         5
                          172         6
                          856         7
                          457         8
                          561         9
                         1515        10
                            2        .m  .m. Missing
                            1        .s  .s. Skipped by participant

-----------------------------------------------------------------------------------------------
bl_802a                                                 802: Health problems past month: Fever
```

```
-------------------------------------------------------------------------------------------

                  type:  numeric (byte)
                 label:  yesno_bl

                 range:  [0,1]                        units:  1
         unique values:  2                        missing .:  0/4168
       unique mv codes:  1                       missing .*:  2/4168

            tabulation:  Freq.   Numeric  Label
                          3085         0  0. No
                          1081         1  1. Yes
                             2        .m  .m. Missing

-------------------------------------------------------------------------------------------
bl_802b                                          802: Health problems past month: Night sweat
-------------------------------------------------------------------------------------------

                  type:  numeric (byte)
                 label:  yesno_bl

                 range:  [0,1]                        units:  1
         unique values:  2                        missing .:  0/4168
       unique mv codes:  1                       missing .*:  2/4168

            tabulation:  Freq.   Numeric  Label
                          3364         0  0. No
                           802         1  1. Yes
                             2        .m  .m. Missing

-------------------------------------------------------------------------------------------
bl_802c                                    802: Health problems past month: Rapid weight loss
-------------------------------------------------------------------------------------------

                  type:  numeric (byte)
                 label:  yesno_bl

                 range:  [0,1]                        units:  1
         unique values:  2                        missing .:  0/4168
       unique mv codes:  1                       missing .*:  2/4168

            tabulation:  Freq.   Numeric  Label
                          3657         0  0. No
                           509         1  1. Yes
                             2        .m  .m. Missing

-------------------------------------------------------------------------------------------
bl_802d                                   802: Health problems past month: Recurring diarrhea
-------------------------------------------------------------------------------------------

                  type:  numeric (byte)
                 label:  yesno_bl

                 range:  [0,1]                        units:  1
         unique values:  2                        missing .:  0/4168
       unique mv codes:  1                       missing .*:  2/4168

            tabulation:  Freq.   Numeric  Label
                          3483         0  0. No
                           683         1  1. Yes
                             2        .m  .m. Missing

-------------------------------------------------------------------------------------------
bl_802e            802: Health problems past month: Recurring coughing or shortness of breath
-------------------------------------------------------------------------------------------

                  type:  numeric (byte)
                 label:  yesno_bl

                 range:  [0,1]                        units:  1
```

```
        unique values:  2                        missing .:  0/4168
      unique mv codes:  1                       missing .*:  2/4168

          tabulation:  Freq.   Numeric  Label
                        3433         0  0. No
                         733         1  1. Yes
                           2        .m  .m. Missing

-----------------------------------------------------------------------------------------------
bl_802f                                    802: Health problems past month: Recurring vomiting
-----------------------------------------------------------------------------------------------

                 type:  numeric (byte)
                label:  yesno_bl

                range:  [0,1]                        units:  1
        unique values:  2                        missing .:  0/4168
      unique mv codes:  1                       missing .*:  2/4168

          tabulation:  Freq.   Numeric  Label
                        3689         0  0. No
                         477         1  1. Yes
                           2        .m  .m. Missing

-----------------------------------------------------------------------------------------------
bl_802g                                     802: Health problems past month: Recurring fatigue
-----------------------------------------------------------------------------------------------

                 type:  numeric (byte)
                label:  yesno_bl

                range:  [0,1]                        units:  1
        unique values:  2                        missing .:  0/4168
      unique mv codes:  1                       missing .*:  2/4168

          tabulation:  Freq.   Numeric  Label
                        3503         0  0. No
                         663         1  1. Yes
                           2        .m  .m. Missing

-----------------------------------------------------------------------------------------------
bl_803                                                 803: Covered by health insurance/scheme
-----------------------------------------------------------------------------------------------

                 type:  numeric (byte)
                label:  yesno_bl

                range:  [0,88]                       units:  1
        unique values:  3                        missing .:  0/4168
      unique mv codes:  1                       missing .*:  2/4168

          tabulation:  Freq.   Numeric  Label
                        3921         0  0. No
                         228         1  1. Yes
                          17        88  88. Don't Know
                           2        .m  .m. Missing

-----------------------------------------------------------------------------------------------
bl_804a       804: Health insurance: Mutual health organization/community-based health insuran
-----------------------------------------------------------------------------------------------

                 type:  numeric (byte)
                label:  bl_804a

                range:  [0,1]                        units:  1
        unique values:  2                        missing .:  0/4168
      unique mv codes:  3                       missing .*:  3943/4168

          tabulation:  Freq.   Numeric  Label
                         214         0  0. No
```

```
                      11         1  1. Yes
                    3938        .a  .a. No/Don't know health
                                    insurance
                       2        .m  .m. Missing
                       3        .s  .s. Skipped by participant

-----------------------------------------------------------------------------------------------
bl_804b                             804: Health insurance: Health insurance through employer
-----------------------------------------------------------------------------------------------

                  type:  numeric (byte)
                 label:  bl_804b

                 range:  [0,1]                        units:  1
         unique values:  2                        missing .:  0/4168
       unique mv codes:  3                       missing .*:  3943/4168

            tabulation:  Freq.   Numeric  Label
                           107         0  0. No
                           118         1  1. Yes
                          3938        .a  .a. No/Don't know health
                                          insurance
                             2        .m  .m. Missing
                             3        .s  .s. Skipped by participant

-----------------------------------------------------------------------------------------------
bl_804c                                                  804: Health insurance: Social security
-----------------------------------------------------------------------------------------------

                  type:  numeric (byte)
                 label:  bl_804c

                 range:  [0,1]                        units:  1
         unique values:  2                        missing .:  0/4168
       unique mv codes:  3                       missing .*:  3943/4168

            tabulation:  Freq.   Numeric  Label
                           217         0  0. No
                             8         1  1. Yes
                          3938        .a  .a. No/Don't know health
                                          insurance
                             2        .m  .m. Missing
                             3        .s  .s. Skipped by participant

-----------------------------------------------------------------------------------------------
bl_804d          804: Health insurance: Other privately purchased commercial health insurance
-----------------------------------------------------------------------------------------------

                  type:  numeric (byte)
                 label:  bl_804d

                 range:  [0,1]                        units:  1
         unique values:  2                        missing .:  0/4168
       unique mv codes:  3                       missing .*:  3943/4168

            tabulation:  Freq.   Numeric  Label
                           197         0  0. No
                            28         1  1. Yes
                          3938        .a  .a. No/Don't know health
                                          insurance
                             2        .m  .m. Missing
                             3        .s  .s. Skipped by participant

-----------------------------------------------------------------------------------------------
bl_804e                                   804: Health insurance: Low cost pre-payment scheme
-----------------------------------------------------------------------------------------------

                  type:  numeric (byte)
                 label:  bl_804e
```

```
                 range:  [0,1]                        units:  1
         unique values:  2                        missing .:  0/4168
       unique mv codes:  3                       missing .*:  3943/4168

            tabulation:  Freq.   Numeric  Label
                           182         0  0. No
                            43         1  1. Yes
                          3938        .a  .a. No/Don't know health
                                          insurance
                             2        .m  .m. Missing
                             3        .s  .s. Skipped by participant

-----------------------------------------------------------------------------------------------
bl_804f                                    804: Health insurance: High cost pre-payment scheme
-----------------------------------------------------------------------------------------------

                  type:  numeric (byte)
                 label:  bl_804f

                 range:  [0,1]                        units:  1
         unique values:  2                        missing .:  0/4168
       unique mv codes:  3                       missing .*:  3943/4168

            tabulation:  Freq.   Numeric  Label
                           206         0  0. No
                            19         1  1. Yes
                          3938        .a  .a. No/Don't know health
                                          insurance
                             2        .m  .m. Missing
                             3        .s  .s. Skipped by participant

-----------------------------------------------------------------------------------------------
bl_804g                                                 804: Health insurance: Other (specify)
-----------------------------------------------------------------------------------------------

                  type:  string (str41)

         unique values:  7                        missing "":  0/4168

            tabulation:  Freq.  Value
                          4162  "."
                             1  "African Life insurance and zisc
                                insurance"
                             1  "African life health insurance"
                             1  "Airtel life insurance"
                             1  "Chesher Homes"
                             1  "Partners health scheme"
                             1  "We would just buy books"

               warning:  variable has embedded blanks

-----------------------------------------------------------------------------------------------
bl_900                                                   900: Age at first sexual intercourse
-----------------------------------------------------------------------------------------------

                  type:  numeric (byte)
                 label:  bl_900, but 35 nonmissing values are not labeled

                 range:  [0,88]                       units:  1
         unique values:  37                       missing .:  0/4168
       unique mv codes:  2                       missing .*:  37/4168

              examples:  16
                         18
                         19
                         23

-----------------------------------------------------------------------------------------------
bl_901                        901: Relationship to FIRST person with whom had sexual intercourse
-----------------------------------------------------------------------------------------------
```

```
              type:  numeric (byte)
             label:  bl_901

             range:  [1,6]                        units:  1
     unique values:  6                        missing .:  0/4168
   unique mv codes:  3                       missing .*:  196/4168

        tabulation:  Freq.   Numeric  Label
                      1130         1  1. Husband/wife
                       709         2  2. Live-in partner
                      1790         3  3. Girlfriend/boyfriend not
                                      living with you
                        86         4  4. Person you paid or who paid
                                      you for sex
                       197         5  5. Casual acquaintance
                        60         6  6. Other
                       158        .a  .a. Never had sex
                         6        .m  .m. Missing
                        32        .s  .s. Skipped by participant

--------------------------------------------------------------------------------------------
bl_902                                                   902: Last time had sexual intercourse
--------------------------------------------------------------------------------------------

              type:  numeric (byte)
             label:  bl_902

             range:  [1,4]                        units:  1
     unique values:  4                        missing .:  0/4168
   unique mv codes:  3                       missing .*:  181/4168

        tabulation:  Freq.   Numeric  Label
                      1301         1  1. Today/Days ago
                      1147         2  2. More than a week ago
                      1127         3  3. More than a month ago
                       412         4  4. More than a year ago
                       158        .a  .a. Never had sex
                         6        .m  .m. Missing
                        17        .s  .s. Skipped by participant

--------------------------------------------------------------------------------------------
bl_903                                                         903: Times had sex in last week
--------------------------------------------------------------------------------------------

              type:  numeric (byte)
             label:  bl_903, but 19 nonmissing values are not labeled

             range:  [1,88]                       units:  1
     unique values:  20                       missing .:  0/4168
   unique mv codes:  4                       missing .*:  2883/4168

        tabulation:  Freq.   Numeric  Label
                       331         1
                       319         2
                       222         3
                        72         4
                        29         5
                        20         6
                        36         7
                        13         8
                         7         9
                         2        10
                        10        11
                        10        12
                         1        13
                         2        14
                         1        15
                         1        16
                         1        17
```

```
                          1        18
                          1        19
                        206        88  88. Don't Know
                        158        .a  .a. Never had sex
                       2686        .b  .b. Sex >1 week ago
                          6        .m  .m. Missing
                         33        .s  .s. Skipped by participant

-----------------------------------------------------------------------------------------------
bl_904                                        904: Times used condom when had sex in last week
-----------------------------------------------------------------------------------------------

                  type:  numeric (byte)
                 label:  bl_904, but 16 nonmissing values are not labeled

                 range:  [1,88]                       units:  1
         unique values:  17                       missing .:  0/4168
       unique mv codes:  4                       missing .*:  2979/4168

            tabulation:  Freq.   Numeric  Label
                           382         1
                           242         2
                           101         3
                            29         4
                            10         5
                            11         6
                            16         7
                             7         8
                             7         9
                             1        10
                             2        11
                             6        12
                             2        13
                             1        14
                             1        15
                             1        16
                           370        88  88. Don't Know
                           158        .a  .a. Never had sex
                          2686        .b  .b. Sex >1 week ago
                             6        .m  .m. Missing
                           129        .s  .s. Skipped by participant

bl_904:
  1.  There was no "0" option on this question.

-----------------------------------------------------------------------------------------------
bl_905                          905: Relationship to LAST person with whom had sexual intercourse
-----------------------------------------------------------------------------------------------

                  type:  numeric (byte)
                 label:  bl_905

                 range:  [1,6]                        units:  1
         unique values:  6                        missing .:  0/4168
       unique mv codes:  4                       missing .*:  600/4168

            tabulation:  Freq.   Numeric  Label
                          1542         1  1. Husband/wife
                           513         2  2. Live-in partner
                          1280         3  3. Girlfriend/boyfriend not
                                          living with you
                            89         4  4. Person you paid or who paid
                                          you for sex
                           101         5  5. Casual acquaintance
                            43         6  6. Other
                           158        .a  .a. Never had sex
                           412        .b  .b. Sex >1 year ago
                             6        .m  .m. Missing
                            24        .s  .s. Skipped by participant
```

```
-------------------------------------------------------------------------------
bl_906                                      906: Condom used last time had sex
-------------------------------------------------------------------------------

                  type:  numeric (byte)
                 label:  bl_906

                 range:  [0,88]                       units:  1
         unique values:  3                        missing .:  0/4168
       unique mv codes:  4                       missing .*:  579/4168

            tabulation:  Freq.   Numeric  Label
                          2012         0  0. No
                          1458         1  1. Yes
                           119        88  88. Don't Know
                           158        .a  .a. Never had sex
                           412        .b  .b. Sex >1 year ago
                             6        .m  .m. Missing
                             3        .s  .s. Skipped by participant

-------------------------------------------------------------------------------
bl_907                   907: Use condom every time had sex with LAST person in last 12 months
-------------------------------------------------------------------------------

                  type:  numeric (byte)
                 label:  bl_907

                 range:  [0,88]                       units:  1
         unique values:  3                        missing .:  0/4168
       unique mv codes:  4                       missing .*:  580/4168

            tabulation:  Freq.   Numeric  Label
                          2225         0  0. No
                          1141         1  1. Yes
                           222        88  88. Don't Know
                           158        .a  .a. Never had sex
                           412        .b  .b. Sex >1 year ago
                             6        .m  .m. Missing
                             4        .s  .s. Skipped by participant

-------------------------------------------------------------------------------
bl_908              908: Having sex with other people during time having sex with LAST person
-------------------------------------------------------------------------------

                  type:  numeric (byte)
                 label:  bl_908

                 range:  [0,88]                       units:  1
         unique values:  3                        missing .:  0/4168
       unique mv codes:  4                       missing .*:  581/4168

            tabulation:  Freq.   Numeric  Label
                          2514         0  0. No
                           896         1  1. Yes
                           177        88  88. Don't Know
                           158        .a  .a. Never had sex
                           412        .b  .b. Sex >1 year ago
                             6        .m  .m. Missing
                             5        .s  .s. Skipped by participant

-------------------------------------------------------------------------------
bl_909                         909: Total number of people had sex with in last 12 months
-------------------------------------------------------------------------------

                  type:  numeric (byte)
                 label:  bl_909, but 31 nonmissing values are not labeled

                 range:  [1,88]                       units:  1
         unique values:  32                       missing .:  0/4168
       unique mv codes:  4                       missing .*:  630/4168
```

```
             examples:  1
                        2
                        3
                        88    88. Don't Know

--------------------------------------------------------------------------------------------
bl_910                                   910: Total number of people had sex with in lifetime
--------------------------------------------------------------------------------------------

                 type:  numeric (byte)
                label:  bl_910, but 34 nonmissing values are not labeled

                range:  [1,88]                       units:  1
        unique values:  35                       missing .:  0/4168
      unique mv codes:  3                       missing .*:  231/4168

             examples:  2
                        3
                        6
                        88    88. Don't Know

--------------------------------------------------------------------------------------------
bl_1000       1000: Told by medical professional had sexually transmitted infection in last 12
--------------------------------------------------------------------------------------------

                 type:  numeric (byte)
                label:  yesno_bl

                range:  [0,1]                        units:  1
        unique values:  2                        missing .:  0/4168
      unique mv codes:  2                       missing .*:  11/4168

           tabulation:  Freq.   Numeric  Label
                         3508         0  0. No
                          649         1  1. Yes
                            7        .m  .m. Missing
                            4        .s  .s. Skipped by participant

--------------------------------------------------------------------------------------------
bl_1001                 1001: White or foul smelling dischaged from genitals in last 12 months
--------------------------------------------------------------------------------------------

                 type:  numeric (byte)
                label:  yesno_bl

                range:  [0,1]                        units:  1
        unique values:  2                        missing .:  0/4168
      unique mv codes:  2                       missing .*:  9/4168

           tabulation:  Freq.   Numeric  Label
                         3286         0  0. No
                          873         1  1. Yes
                            7        .m  .m. Missing
                            2        .s  .s. Skipped by participant

--------------------------------------------------------------------------------------------
bl_1002                                1002: Burning or pain during urination in last 12 months
--------------------------------------------------------------------------------------------

                 type:  numeric (byte)
                label:  yesno_bl

                range:  [0,1]                        units:  1
        unique values:  2                        missing .:  0/4168
      unique mv codes:  2                       missing .*:  9/4168

           tabulation:  Freq.   Numeric  Label
                         3207         0  0. No
                          952         1  1. Yes
```

```
                           7        .m  .m. Missing
                           2        .s  .s. Skipped by participant

-------------------------------------------------------------------------------------------------
bl_1003                                    1003: Sores or ulcers on genitals in last 12 months
-------------------------------------------------------------------------------------------------

                  type:  numeric (byte)
                 label:  yesno_bl

                 range:  [0,1]                        units:  1
         unique values:  2                        missing .:  0/4168
       unique mv codes:  2                       missing .*:  10/4168

           tabulation:  Freq.   Numeric  Label
                         3445         0  0. No
                          713         1  1. Yes
                            7        .m  .m. Missing
                            3        .s  .s. Skipped by participant

-------------------------------------------------------------------------------------------------
bl_1004                                        1004: Itching in genital area in last 12 months
-------------------------------------------------------------------------------------------------

                  type:  numeric (byte)
                 label:  yesno_bl

                 range:  [0,1]                        units:  1
         unique values:  2                        missing .:  0/4168
       unique mv codes:  2                       missing .*:  9/4168

           tabulation:  Freq.   Numeric  Label
                         3163         0  0. No
                          996         1  1. Yes
                            7        .m  .m. Missing
                            2        .s  .s. Skipped by participant

-------------------------------------------------------------------------------------------------
bl_1005                           1005: Swelling or tenderness in genital area in last 12 months
-------------------------------------------------------------------------------------------------

                  type:  numeric (byte)
                 label:  yesno_bl

                 range:  [0,1]                        units:  1
         unique values:  2                        missing .:  0/4168
       unique mv codes:  2                       missing .*:  11/4168

           tabulation:  Freq.   Numeric  Label
                         3674         0  0. No
                          483         1  1. Yes
                            7        .m  .m. Missing
                            4        .s  .s. Skipped by participant

-------------------------------------------------------------------------------------------------
bl_currpart_an                              Analysis: Currently has spouse/primary sex partner
-------------------------------------------------------------------------------------------------

                  type:  numeric (byte)
                 label:  yesno_bl

                 range:  [0,1]                        units:  1
         unique values:  2                        missing .:  0/4168
       unique mv codes:  1                       missing .*:  4/4168

           tabulation:  Freq.   Numeric  Label
                          705         0  0. No
                         3459         1  1. Yes
                            4        .s  .s. Skipped by participant
```

```
bl_currpart_an:
  1.  Created for analysis: from 201, 205, 211; gen bl_currpart_an = bl_201/ replace
      bl_currpart_an = 1 if bl_205== 1|bl_205== 2/ replace bl_currpart_an = 0 if bl_211 == 0/
      replace bl_currpart_an = 1 if bl_211 == 1 replace bl_currpart_an = .s if bl_211 == .s &
      bl_205 == 0 replace bl_currpart_an = 1 if bl_212_m >0 & bl_212_m < . & bl_currpart_an >.

----------------------------------------------------------------------------------------------
w6_time                                                                             Time Point
----------------------------------------------------------------------------------------------

                  type:  numeric (byte)
                 label:  time

                 range:  [1,2]                        units:  1
         unique values:  2                        missing .:  51/4168
       unique mv codes:  2                       missing .*:  699/4168

            tabulation:  Freq.   Numeric  Label
                          3413         1  1. 6 Weeks
                             5         2  2. 6 Months
                            51         .
                           699        .x

----------------------------------------------------------------------------------------------
w6_ID                                                                6 Week Survey: Survey ID
----------------------------------------------------------------------------------------------

                  type:  numeric (int)
                 label:  admin_w6, but 3169 nonmissing values are not labeled

                 range:  [272,14919]                  units:  1
         unique values:  3169                     missing .:  51/4168
       unique mv codes:  2                       missing .*:  699/4168

              examples:  6414
                         9336
                         11437
                         13900

----------------------------------------------------------------------------------------------
w6_Date                              6 Week Survey: Interviewer entered date, System timestamp
----------------------------------------------------------------------------------------------

                  type:  numeric (double)
                 label:  admin_w6, but 3418 nonmissing values are not labeled

                 range:  [1.705e+12,1.744e+12]        units:  1000
         unique values:  3418                     missing .:  51/4168
       unique mv codes:  2                       missing .*:  699/4168

              examples:  1.716e+12
                         1.721e+12
                         1.725e+12
                         1.734e+12

----------------------------------------------------------------------------------------------
w6_SurveyStatus                                                  6 Week Survey: Survey Status
----------------------------------------------------------------------------------------------

                  type:  numeric (byte)
                 label:  SurveyStatus_w6

                 range:  [0,5]                        units:  1
         unique values:  6                        missing .:  0/4168
       unique mv codes:  1                       missing .*:  726/4168

            tabulation:  Freq.   Numeric  Label
                            10         0  0. Interrupted
                          3390         1  1. Completed
                            13         2  2. Completed on resumption
```

```
                          5         3  3. Completed 6 mon survey at 6
                                       wk visit
                         20         4  4. Withdrew from study
                          4         5  5. Deceased
                        726        .x  .x. No 6 week survey

-------------------------------------------------------------------------------------------------
w6_AdminAge                                        6 Week Survey: Age entered by interviewer
-------------------------------------------------------------------------------------------------

                  type:  numeric (byte)
                 label:  admin_w6, but 31 nonmissing values are not labeled

                 range:  [18,48]                      units:  1
         unique values:  31                       missing .:  51/4168
       unique mv codes:  2                       missing .*:  699/4168

              examples:  21
                         25
                         30
                         43

w6_AdminAge:
  1.  Do not use this variable for analysis. Entered for administrative purposes. Should use
      bl_102a.

-------------------------------------------------------------------------------------------------
w6_SurveyGender                                       6 Week Survey: Administrative variable
-------------------------------------------------------------------------------------------------

                  type:  string (str6)

         unique values:  2                        missing "":  750/4168

            tabulation:  Freq.  Value
                           750  ""
                          1785  "Female"
                          1633  "Male"

w6_SurveyGender:
  1.  Survey gender does NOT always match true participant gender.

-------------------------------------------------------------------------------------------------
w6_InterviewerID                                               6 Week Survey: Interviewer ID
-------------------------------------------------------------------------------------------------

                  type:  numeric (int)
                 label:  admin_w6, but 28 nonmissing values are not labeled

                 range:  [100,323]                    units:  1
         unique values:  28                       missing .:  51/4168
       unique mv codes:  2                       missing .*:  699/4168

            tabulation:  Freq.   Numeric  Label
                           119       100
                           176       101
                           116       102
                           212       103
                           113       104
                           106       105
                           132       106
                            26       107
                            70       108
                           182       109
                            72       111
                            90       112
                            72       113
                           176       114
                           251       115
                           179       116
```

```
                        259       117
                        200       118
                        173       119
                        149       120
                          1       147
                          1       156
                          1       158
                        154       210
                         59       211
                        246       222
                         82       223
                          1       323
                         51         .
                        699        .x  .x. No 6 week survey

-------------------------------------------------------------------------------
w6_GUID_sys                                 6 Week Survey: Unique survey identifier
-------------------------------------------------------------------------------

                  type:  string (str36)

         unique values:  3418                     missing "":  750/4168

              examples:  "05ee75bd-0deb-4717-83fc-dfab11bb9766"
                         "447ce40c-5fa0-45a8-8f0f-fbdf0717b553"
                         "80cb364e-e947-47c2-b7c9-41ef85fff4b4"
                         "c28c59b6-3d80-4e90-a400-247248be0e05"

-------------------------------------------------------------------------------
w6_SiteID                                            6 Week Survey: Survey Site ID
-------------------------------------------------------------------------------

                  type:  numeric (byte)
                 label:  w6_SiteID

                 range:  [1,95]                       units:  1
         unique values:  12                       missing .:  51/4168
       unique mv codes:  2                       missing .*:  699/4168

            tabulation:  Freq.   Numeric  Label
                          423         1  1. SFH HCT - Cairo Road
                          378         2  2. Chawama Clinic - MCH
                          198         3  3. Chawama Clinic - Out Patient
                                         Ward (VMMC)
                          264         5  5. Kamwala Clinic - TB, STI &
                                         HIV Clinic
                          497         6  6. Kapata Urban Clinic, MCH
                          659         7  7. Kapata Urban Clinic, TB, STI
                                         & HIV
                          167         8  8. Chipata Gen Hosp - OP VMMC
                          614        11  11. SFH New Start
                          109        13  13. SFH VMMC
                            7        93  93. YWCA FP Site
                           46        94  94. YWCA HTC Site
                           56        95  95. YWCA VMMC
                           51         .
                          699        .x  .x. No 6 week survey

w6_SiteID:
  1.  93, 94, & 95 are external evaluation sites

-------------------------------------------------------------------------------
w6_SiteName                                     6 Week Survey: Survey Site String
-------------------------------------------------------------------------------

                  type:  string (str40)

         unique values:  14                       missing "":  750/4168

            tabulation:  Freq.  Value
```

```
                          750  ""
                          378  "Chawama Clinic - Maternity Ward"
                          198  "Chawama Clinic - Out Patient Ward
                               (VMMC)"
                          167  "Chipata Gen Hosp - OP VMMC"
                          186  "Kamwala Clinic - TB, STI & HIV Clinic"
                           78  "Kamwala Clinic - TB, STI and HIV
                               Clinic"
                          497  "Kapata Urban Clinic, MCH"
                          415  "Kapata Urban Clinic, TB, STI & HIV"
                          244  "Kapata Urban Clinic, TB, STI and HIV"
                          423  "SFH HCT - Cairo Road"
                          614  "SFH New Start"
                          109  "SFH VMMC"
                            7  "YWCA FP Site"
                           46  "YWCA HTC Site"
                           56  "YWCA VMMC"

             warning:  variable has embedded blanks

w6_SiteName:
  1.  YWCA sites are external evaluation sites

-----------------------------------------------------------------------------------------------
w6_SurveyType                                                        6 Week Survey: Survey Type
-----------------------------------------------------------------------------------------------

                type:  numeric (byte)
               label:  admin_w6, but 2 nonmissing values are not labeled

               range:  [3,4]                        units:  1
       unique values:  2                        missing .:  51/4168
     unique mv codes:  2                       missing .*:  699/4168

          tabulation:  Freq.   Numeric  Label
                        3413         3
                           5         4
                          51         .
                         699        .x  .x. No 6 week survey

-----------------------------------------------------------------------------------------------
w6_SurveyTypeName                                             6 Week Survey: Survey Type String
-----------------------------------------------------------------------------------------------

                type:  string (str21)

       unique values:  2                        missing "":  750/4168

          tabulation:  Freq.  Value
                         750  ""
                           5  "Reach 6 MONTHS - Both"
                        3413  "Reach 6 WEEKS - Both"

             warning:  variable has embedded blanks

-----------------------------------------------------------------------------------------------
w6_SurveyDuration_sys                                             Minutes for survey completion
-----------------------------------------------------------------------------------------------

                type:  numeric (int)
               label:  admin_w6, but 62 nonmissing values are not labeled

               range:  [-708,1459]                  units:  1
       unique values:  62                       missing .:  51/4168
     unique mv codes:  2                       missing .*:  699/4168

            examples:  7
                       10
                       14
                       26
```

```
w6_SurveyDuration_sys:
  1.  Large values may be due to completing survey on resumption (see SurveyStatus).

-----------------------------------------------------------------------------------------------
w6_Weekday_sys                                                     6 Week Survey: Day of week
-----------------------------------------------------------------------------------------------

                  type:  string (str3)

         unique values:  7                        missing "":  750/4168

           tabulation:  Freq.  Value
                          750  ""
                          746  "Fri"
                          635  "Mon"
                          131  "Sat"
                           38  "Sun"
                          737  "Thu"
                          571  "Tue"
                          560  "Wed"

w6_Weekday_sys:
  1.  Generated automatically based on w6_Date

-----------------------------------------------------------------------------------------------
w6_Day_sys                                                        6 Week Survey: Day of month
-----------------------------------------------------------------------------------------------

                  type:  numeric (byte)
                 label:  admin_w6, but 31 nonmissing values are not labeled

                 range:  [1,31]                       units:  1
         unique values:  31                       missing .:  51/4168
       unique mv codes:  2                       missing .*:  699/4168

              examples:  8
                         16
                         24
                         31

w6_Day_sys:
  1.  Generated automatically based on w6_Date

-----------------------------------------------------------------------------------------------
w6_Month_sys                                                      6 Week Survey: Day of month
-----------------------------------------------------------------------------------------------

                  type:  numeric (byte)
                 label:  admin_w6, but 12 nonmissing values are not labeled

                 range:  [1,12]                       units:  1
         unique values:  12                       missing .:  51/4168
       unique mv codes:  2                       missing .*:  699/4168

           tabulation:  Freq.   Numeric  Label
                           12         1
                           64         2
                          198         3
                          337         4
                          525         5
                          445         6
                          548         7
                          389         8
                          320         9
                          260        10
                          196        11
                          124        12
                           51         .
                          699        .x  .x. No 6 week survey
```

```
w6_Month_sys:
  1.  Generated automatically based on w6_Date

-----------------------------------------------------------------------------------------------
w6_DBVersion                                                                          DBVersion
-----------------------------------------------------------------------------------------------

                  type:  numeric (double)
                 label:  admin_w6, but 8 nonmissing values are not labeled

                 range:  [1.5,2.6]                    units:  .1
         unique values:  8                        missing .:  51/4168
       unique mv codes:  2                       missing .*:  699/4168

            tabulation:  Freq.   Numeric  Label
                             1       1.5
                             1       1.8
                            62       1.9
                            22         2
                           582       2.1
                           727       2.2
                          1240       2.5
                           783       2.6
                            51         .
                           699        .x  .x. No 6 week survey

-----------------------------------------------------------------------------------------------
w6_AppVersion                                                                        AppVersion
-----------------------------------------------------------------------------------------------

                  type:  numeric (double)
                 label:  admin_w6, but 8 nonmissing values are not labeled

                 range:  [1.4,2.6]                    units:  .1
         unique values:  8                        missing .:  51/4168
       unique mv codes:  2                       missing .*:  699/4168

            tabulation:  Freq.   Numeric  Label
                             1       1.4
                             1       1.8
                            62       1.9
                            22         2
                           582       2.1
                           727       2.2
                          1240       2.5
                           783       2.6
                            51         .
                           699        .x  .x. No 6 week survey

-----------------------------------------------------------------------------------------------
w6_100                                                                              100: Gender
-----------------------------------------------------------------------------------------------

                  type:  numeric (byte)
                 label:  w6_100

                 range:  [0,1]                        units:  1
         unique values:  2                        missing .:  51/4168
       unique mv codes:  2                       missing .*:  699/4168

            tabulation:  Freq.   Numeric  Label
                          1633         0  0. Male
                          1785         1  1. Female
                            51         .
                           699        .x  .x. No 6 week survey

-----------------------------------------------------------------------------------------------
w6_101                                                       101: Married or living with someone
-----------------------------------------------------------------------------------------------
```

```
                type:  numeric (byte)
               label:  w6_101

               range:  [0,2]                        units:  1
       unique values:  3                        missing .:  51/4168
     unique mv codes:  4                       missing .*:  703/4168

          tabulation:  Freq.   Numeric  Label
                        1697         0  0. No
                        1656         1  1. Yes, married
                          61         2  2. Yes, living with partner
                          51         .
                           2        .m  .m. Missing
                           2        .s  .s. Skipped by participant
                         699        .x  .x. No 6 week survey

-----------------------------------------------------------------------------------------------
w6_102                                                                  102: Primary sex partner
-----------------------------------------------------------------------------------------------

                type:  numeric (byte)
               label:  w6_102

               range:  [0,1]                        units:  1
       unique values:  2                        missing .:  51/4168
     unique mv codes:  5                       missing .*:  2420/4168

          tabulation:  Freq.   Numeric  Label
                         563         0  0. No
                        1134         1  1. Yes
                          51         .
                        1717        .a  .a. Married/Living with someone
                           3        .m  .m. Missing
                           1        .s  .s. Skipped by participant
                         699        .x  .x. No 6 week survey

-----------------------------------------------------------------------------------------------
w6_103                                                                103: Children 15 and older
-----------------------------------------------------------------------------------------------

                type:  numeric (byte)
               label:  w6_yesno

               range:  [0,1]                        units:  1
       unique values:  2                        missing .:  51/4168
     unique mv codes:  4                       missing .*:  705/4168

          tabulation:  Freq.   Numeric  Label
                        2983         0  0. No
                         429         1  1. Yes
                          51         .
                           5        .m  .m. Missing
                           1        .s  .s. Skipped by participant
                         699        .x  .x. No 6 week survey

-----------------------------------------------------------------------------------------------
w6_104                 104: Respondent understands to only include services from last 6 weeks
-----------------------------------------------------------------------------------------------

                type:  numeric (byte)
               label:  w6_yesno

               range:  [1,1]                        units:  1
       unique values:  1                        missing .:  51/4168
     unique mv codes:  4                       missing .*:  707/4168

          tabulation:  Freq.   Numeric  Label
                        3410         1  1. Yes
                          51         .
```

```
                           3        .m  .m. Missing
                           5        .w  .w. Answered 6 mon survey at 6
                                        wk visit
                         699        .x  .x. No 6 week survey
```

```
w6_105a                                105: Respondent: HIV testing/counseling in last 6 weeks
```

```
                  type:  numeric (byte)
                 label:  w6_yesno

                 range:  [0,1]                        units:  1
         unique values:  2                        missing .:  51/4168
       unique mv codes:  4                       missing .*:  709/4168

            tabulation:  Freq.   Numeric  Label
                          2847         0  0. No
                           561         1  1. Yes
                            51         .
                             5        .m  .m. Missing
                             5        .w  .w. Answered 6 mon survey at 6
                                          wk visit
                           699        .x  .x. No 6 week survey
```

```
w6_105b                                105: Respondent: HIV care and treatment in last 6 weeks
```

```
                  type:  numeric (byte)
                 label:  w6_yesno

                 range:  [0,1]                        units:  1
         unique values:  2                        missing .:  51/4168
       unique mv codes:  5                       missing .*:  717/4168

            tabulation:  Freq.   Numeric  Label
                          3083         0  0. No
                           317         1  1. Yes
                            51         .
                             5        .m  .m. Missing
                             8        .s  .s. Skipped by participant
                             5        .w  .w. Answered 6 mon survey at 6
                                          wk visit
                           699        .x  .x. No 6 week survey
```

```
w6_105c                                105: Respondent: STD care and treatment in last 6 weeks
```

```
                  type:  numeric (byte)
                 label:  w6_yesno

                 range:  [0,1]                        units:  1
         unique values:  2                        missing .:  51/4168
       unique mv codes:  5                       missing .*:  713/4168

            tabulation:  Freq.   Numeric  Label
                          3263         0  0. No
                           141         1  1. Yes
                            51         .
                             5        .m  .m. Missing
                             4        .s  .s. Skipped by participant
                             5        .w  .w. Answered 6 mon survey at 6
                                          wk visit
                           699        .x  .x. No 6 week survey
```

```
w6_105d_f                                     105: Respondent: Family planning in last 6 weeks
```

```
                  type:  numeric (byte)
                 label:  w6_yesno

                 range:  [0,1]                        units:  1
         unique values:  2                        missing .:  51/4168
       unique mv codes:  6                       missing .*:  2337/4168

            tabulation:  Freq.   Numeric  Label
                          1565         0  0. No
                           215         1  1. Yes
                            51         .
                          1630        .g  .g. Gender skip pattern
                             2        .m  .m. Missing
                             1        .s  .s. Skipped by participant
                             5        .w  .w. Answered 6 mon survey at 6
                                          wk visit
                           699        .x  .x. No 6 week survey

-----------------------------------------------------------------------------------------------
w6_105e_f                                          105: Respondent: Cervical cancer screening
-----------------------------------------------------------------------------------------------

                  type:  numeric (byte)
                 label:  w6_yesno

                 range:  [0,1]                        units:  1
         unique values:  2                        missing .:  51/4168
       unique mv codes:  5                       missing .*:  2336/4168

            tabulation:  Freq.   Numeric  Label
                          1494         0  0. No
                           287         1  1. Yes
                            51         .
                          1630        .g  .g. Gender skip pattern
                             2        .m  .m. Missing
                             5        .w  .w. Answered 6 mon survey at 6
                                          wk visit
                           699        .x  .x. No 6 week survey

-----------------------------------------------------------------------------------------------
w6_105f_m                                    105: Respondent: Male circumcision in last 6 weeks
-----------------------------------------------------------------------------------------------

                  type:  numeric (byte)
                 label:  w6_yesno

                 range:  [0,1]                        units:  1
         unique values:  2                        missing .:  51/4168
       unique mv codes:  6                       missing .*:  2495/4168

            tabulation:  Freq.   Numeric  Label
                          1325         0  0. No
                           297         1  1. Yes
                            51         .
                          1783        .g  .g. Gender skip pattern
                             6        .m  .m. Missing
                             2        .s  .s. Skipped by participant
                             5        .w  .w. Answered 6 mon survey at 6
                                          wk visit
                           699        .x  .x. No 6 week survey

-----------------------------------------------------------------------------------------------
w6_106a                                    106: Partner: HIV testing/counseling in last 6 weeks
-----------------------------------------------------------------------------------------------

                  type:  numeric (byte)
                 label:  w6_106

                 range:  [0,88]                       units:  1
```

```
        unique values:  3                        missing .:  51/4168
      unique mv codes:  6                       missing .*:  1275/4168

          tabulation:  Freq.   Numeric  Label
                        2066         0  0. No
                         602         1  1. Yes
                         174        88  88. Don't Know
                          51         .  
                         562        .a  .a. Married/Living with someone
                           7        .m  .m. Missing
                           2        .s  .s. Skipped by participant
                           5        .w  .w. Answered 6 mon survey at 6
                                        wk visit
                         699        .x  .x. No 6 week survey

-------------------------------------------------------------------------------------------
w6_106b                                 106: Partner: HIV care and treatment in last 6 weeks
-------------------------------------------------------------------------------------------

                 type:  numeric (byte)
                label:  w6_106

                range:  [0,88]                       units:  1
        unique values:  3                        missing .:  51/4168
      unique mv codes:  6                       missing .*:  1275/4168

          tabulation:  Freq.   Numeric  Label
                        2469         0  0. No
                         216         1  1. Yes
                         157        88  88. Don't Know
                          51         .  
                         562        .a  .a. Married/Living with someone
                           7        .m  .m. Missing
                           2        .s  .s. Skipped by participant
                           5        .w  .w. Answered 6 mon survey at 6
                                        wk visit
                         699        .x  .x. No 6 week survey

-------------------------------------------------------------------------------------------
w6_106c                                 106: Partner: STD care and treatment in last 6 weeks
-------------------------------------------------------------------------------------------

                 type:  numeric (byte)
                label:  w6_106

                range:  [0,88]                       units:  1
        unique values:  3                        missing .:  51/4168
      unique mv codes:  6                       missing .*:  1275/4168

          tabulation:  Freq.   Numeric  Label
                        2564         0  0. No
                         131         1  1. Yes
                         147        88  88. Don't Know
                          51         .  
                         562        .a  .a. Married/Living with someone
                           7        .m  .m. Missing
                           2        .s  .s. Skipped by participant
                           5        .w  .w. Answered 6 mon survey at 6
                                        wk visit
                         699        .x  .x. No 6 week survey

-------------------------------------------------------------------------------------------
w6_106d_m                                      106: Partner: Family planning in last 6 weeks
-------------------------------------------------------------------------------------------

                 type:  numeric (byte)
                label:  w6_106

                range:  [0,88]                       units:  1
        unique values:  3                        missing .:  51/4168
```

```
     unique mv codes:  6                      missing .*:  2884/4168

          tabulation:  Freq.   Numeric  Label
                         943         0  0. No
                         199         1  1. Yes
                          91        88  88. Don't Know
                          51         .
                         390        .a  .a. Married/Living with someone
                        1783        .g  .g. Gender skip pattern
                           7        .m  .m. Missing
                           5        .w  .w. Answered 6 mon survey at 6
                                        wk visit
                         699        .x  .x. No 6 week survey

-------------------------------------------------------------------------------
w6_106e_m                                  106: Partner: Cervical cancer screening
-------------------------------------------------------------------------------

                type:  numeric (byte)
               label:  w6_106

               range:  [0,88]                      units:  1
       unique values:  3                       missing .:  51/4168
     unique mv codes:  6                      missing .*:  2884/4168

          tabulation:  Freq.   Numeric  Label
                        1030         0  0. No
                          96         1  1. Yes
                         107        88  88. Don't Know
                          51         .
                         390        .a  .a. Married/Living with someone
                        1783        .g  .g. Gender skip pattern
                           7        .m  .m. Missing
                           5        .w  .w. Answered 6 mon survey at 6
                                        wk visit
                         699        .x  .x. No 6 week survey

-------------------------------------------------------------------------------
w6_106f_f                          106: Partner: Male circumcision in last 6 weeks
-------------------------------------------------------------------------------

                type:  numeric (byte)
               label:  w6_106

               range:  [0,88]                      units:  1
       unique values:  3                       missing .:  51/4168
     unique mv codes:  7                      missing .*:  2509/4168

          tabulation:  Freq.   Numeric  Label
                        1508         0  0. No
                          71         1  1. Yes
                          29        88  88. Don't Know
                          51         .
                         172        .a  .a. Married/Living with someone
                        1630        .g  .g. Gender skip pattern
                           2        .m  .m. Missing
                           1        .s  .s. Skipped by participant
                           5        .w  .w. Answered 6 mon survey at 6
                                        wk visit
                         699        .x  .x. No 6 week survey

-------------------------------------------------------------------------------
w6_107a                         107: Children: HIV testing/counseling in last 6 weeks
-------------------------------------------------------------------------------

                type:  numeric (byte)
               label:  w6_107

               range:  [0,88]                      units:  1
       unique values:  3                       missing .:  51/4168
```

```
      unique mv codes:  6                      missing .*:  3688/4168

          tabulation:  Freq.   Numeric  Label
                         345         0  0. No
                          56         1  1. Yes
                          28        88  88. Don't Know
                          51         .
                        2979        .a  .a. No children aged 15 or older
                           3        .m  .m. Missing
                           2        .s  .s. Skipped by participant
                           5        .w  .w. Answered 6 mon survey at 6
                                        wk visit
                         699        .x  .x. No 6 week survey

-------------------------------------------------------------------------------
w6_107b                          107: Children: HIV care and treatment in last 6 weeks
-------------------------------------------------------------------------------

                type:  numeric (byte)
               label:  w6_107

               range:  [0,88]                     units:  1
       unique values:  3                      missing .:  51/4168
      unique mv codes:  6                     missing .*:  3688/4168

          tabulation:  Freq.   Numeric  Label
                         368         0  0. No
                          34         1  1. Yes
                          27        88  88. Don't Know
                          51         .
                        2979        .a  .a. No children aged 15 or older
                           3        .m  .m. Missing
                           2        .s  .s. Skipped by participant
                           5        .w  .w. Answered 6 mon survey at 6
                                        wk visit
                         699        .x  .x. No 6 week survey

-------------------------------------------------------------------------------
w6_107c                          107: Children: STD care and treatment in last 6 weeks
-------------------------------------------------------------------------------

                type:  numeric (byte)
               label:  w6_107

               range:  [0,88]                     units:  1
       unique values:  3                      missing .:  51/4168
      unique mv codes:  6                     missing .*:  3688/4168

          tabulation:  Freq.   Numeric  Label
                         386         0  0. No
                          14         1  1. Yes
                          29        88  88. Don't Know
                          51         .
                        2979        .a  .a. No children aged 15 or older
                           3        .m  .m. Missing
                           2        .s  .s. Skipped by participant
                           5        .w  .w. Answered 6 mon survey at 6
                                        wk visit
                         699        .x  .x. No 6 week survey

-------------------------------------------------------------------------------
w6_107d                                107: Children: Family planning in last 6 weeks
-------------------------------------------------------------------------------

                type:  numeric (byte)
               label:  w6_107

               range:  [0,88]                     units:  1
       unique values:  3                      missing .:  51/4168
      unique mv codes:  6                     missing .*:  3688/4168
```

```
          tabulation:  Freq.   Numeric  Label
                         377         0  0. No
                          21         1  1. Yes
                          31        88  88. Don't Know
                          51         .
                        2979        .a  .a. No children aged 15 or older
                           3        .m  .m. Missing
                           2        .s  .s. Skipped by participant
                           5        .w  .w. Answered 6 mon survey at 6
                                        wk visit
                         699        .x  .x. No 6 week survey

-----------------------------------------------------------------------------------------------
w6_107e                                                107: Children: Cervical cancer screening
-----------------------------------------------------------------------------------------------

                type:  numeric (byte)
               label:  w6_107

               range:  [0,88]                       units:  1
       unique values:  3                        missing .:  51/4168
     unique mv codes:  6                       missing .*:  3688/4168

          tabulation:  Freq.   Numeric  Label
                         387         0  0. No
                          17         1  1. Yes
                          25        88  88. Don't Know
                          51         .
                        2979        .a  .a. No children aged 15 or older
                           3        .m  .m. Missing
                           2        .s  .s. Skipped by participant
                           5        .w  .w. Answered 6 mon survey at 6
                                        wk visit
                         699        .x  .x. No 6 week survey

-----------------------------------------------------------------------------------------------
w6_107f                                        107: Children: Male circumcision in last 6 weeks
-----------------------------------------------------------------------------------------------

                type:  numeric (byte)
               label:  w6_107

               range:  [0,88]                       units:  1
       unique values:  3                        missing .:  51/4168
     unique mv codes:  6                       missing .*:  3688/4168

          tabulation:  Freq.   Numeric  Label
                         397         0  0. No
                          17         1  1. Yes
                          15        88  88. Don't Know
                          51         .
                        2979        .a  .a. No children aged 15 or older
                           3        .m  .m. Missing
                           2        .s  .s. Skipped by participant
                           5        .w  .w. Answered 6 mon survey at 6
                                        wk visit
                         699        .x  .x. No 6 week survey

-----------------------------------------------------------------------------------------------
w6_200             200: HTC: Respondent understands to only include services from last 6 weeks
-----------------------------------------------------------------------------------------------

                type:  numeric (byte)
               label:  yesnoserv

               range:  [1,1]                        units:  1
       unique values:  1                        missing .:  51/4168
     unique mv codes:  6                       missing .*:  3558/4168
```

```
           tabulation:  Freq.   Numeric  Label
                          559         1  1. Yes
                           51         .  
                         2847        .a  .a. Did not receive service
                            5        .m  .m. Missing
                            2        .s  .s. Skipped by participant
                            5        .w  .w. Answered 6 mon survey at 6
                                         wk visit
                          699        .x  .x. No 6 week survey

-------------------------------------------------------------------------------------------------
w6_201                                        201: HTC: numservber times to health facility
-------------------------------------------------------------------------------------------------

                 type:  numeric (byte)
                label:  numserv, but 6 nonmissing values are not labeled

                range:  [1,6]                        units:  1
        unique values:  6                        missing .:  51/4168
      unique mv codes:  6                       missing .*:  3567/4168

           tabulation:  Freq.   Numeric  Label
                          378         1  
                           90         2  
                           40         3  
                           18         4  
                            7         5  
                           17         6  
                           51         .  
                         2847        .a  .a. Did not receive service
                            5        .m  .m. Missing
                           11        .s  .s. Skipped by participant
                            5        .w  .w. Answered 6 mon survey at 6
                                         wk visit
                          699        .x  .x. No 6 week survey

-------------------------------------------------------------------------------------------------
w6_202                                                       202: HTC: Health facility name
-------------------------------------------------------------------------------------------------

                 type:  string (str55)

        unique values:  217                      missing "":  755/4168

             examples:  "."
                        "."
                        "."
                        "."

              warning:  variable has embedded blanks

-------------------------------------------------------------------------------------------------
w6_203                                                       203: HTC: Alone or accompanied
-------------------------------------------------------------------------------------------------

                 type:  numeric (byte)
                label:  hfpeople

                range:  [1,3]                        units:  1
        unique values:  3                        missing .:  51/4168
      unique mv codes:  6                       missing .*:  3563/4168

           tabulation:  Freq.   Numeric  Label
                          360         1  1. Alone
                          147         2  2. Spouse/Partner
                           47         3  3. Someone else
                           51         .  
                         2847        .a  .a. Did not receive service
                            5        .m  .m. Missing
                            7        .s  .s. Skipped by participant
```

```
                          5        .w  .w. Answered 6 mon survey at 6
                                       wk visit
                        699        .x  .x. No 6 week survey

-------------------------------------------------------------------------------------------------
w6_204                                                          204: HTC: Satisfied with services
-------------------------------------------------------------------------------------------------

                  type:  numeric (byte)
                 label:  hfsat

                 range:  [1,3]                        units:  1
         unique values:  3                        missing .:  51/4168
       unique mv codes:  6                       missing .*:  3563/4168

            tabulation:  Freq.   Numeric  Label
                            24         1  1. Not satisfied
                             9         2  2. Neither
                           521         3  3. Satisfied
                            51         .
                          2847        .a  .a. Did not receive service
                             5        .m  .m. Missing
                             7        .s  .s. Skipped by participant
                             5        .w  .w. Answered 6 mon survey at 6
                                          wk visit
                           699        .x  .x. No 6 week survey

-------------------------------------------------------------------------------------------------
w6_205                                          205: HTC: Waiting time at health facility (min)
-------------------------------------------------------------------------------------------------

                  type:  numeric (int)
                 label:  numserv, but 53 nonmissing values are not labeled

                 range:  [1,2400]                     units:  1
         unique values:  53                       missing .:  51/4168
       unique mv codes:  6                       missing .*:  3567/4168

              examples:  .a    .a. Did not receive service
                         .a    .a. Did not receive service
                         .a    .a. Did not receive service
                         .a    .a. Did not receive service

-------------------------------------------------------------------------------------------------
w6_206                                           206: HTC: Travel time to health facility (min)
-------------------------------------------------------------------------------------------------

                  type:  numeric (int)
                 label:  hfttime, but 46 nonmissing values are not labeled

                 range:  [1,240]                      units:  1
         unique values:  46                       missing .:  51/4168
       unique mv codes:  6                       missing .*:  3567/4168

              examples:  .a    .a. Did not receive service
                         .a    .a. Did not receive service
                         .a    .a. Did not receive service
                         .a    .a. Did not receive service

-------------------------------------------------------------------------------------------------
w6_207                                                  207: HTC: Travel mode to health faciity
-------------------------------------------------------------------------------------------------

                  type:  numeric (byte)
                 label:  hftmode

                 range:  [1,7]                        units:  1
         unique values:  7                        missing .:  51/4168
       unique mv codes:  6                       missing .*:  3568/4168
```

tabulation:

Freq.	Numeric	Label
167	1	1. Bus
57	2	2. Taxi
13	3	3. Someone drove in private car
9	4	4. Drove self
269	5	5. Walked
32	6	6. Bicycle
2	7	7. Other
51	.	
2847	.a	.a. Did not receive service
5	.m	.m. Missing
12	.s	.s. Skipped by participant
5	.w	.w. Answered 6 mon survey at 6 wk visit
699	.x	.x. No 6 week survey

---

w6_208 — 208: HTC: Transport cost

---

type: numeric (byte)
label: hftcost, but 23 nonmissing values are not labeled

range: [0,100] — units: 1
unique values: 24 — missing .: 51/4168
unique mv codes: 7 — missing .*: 3877/4168

tabulation:

Freq.	Numeric	Label
21	0	0. Nothing
1	1	
24	2	
32	3	
24	4	
46	5	
3	6	
3	7	
2	8	
27	10	
2	12	
1	14	
14	15	
1	18	
18	20	
3	25	
1	28	
9	30	
1	35	
1	40	
1	45	
3	50	
1	60	
1	100	
51	.	
2847	.a	.a. Did not receive service
310	.b	.b. Own car, walked, biked
5	.m	.m. Missing
11	.s	.s. Skipped by participant
5	.w	.w. Answered 6 mon survey at 6 wk visit
699	.x	.x. No 6 week survey

---

w6_209 — 209: HTC: Distance traveled (km)

---

type: numeric (int)
label: hftkm, but 24 nonmissing values are not labeled

range: [0,888] — units: 1
unique values: 26 — missing .: 51/4168

```
      unique mv codes:  6                      missing .*:  3612/4168

          tabulation:  Freq.   Numeric  Label
                          31         0  0. <1
                          63         1
                          50         2
                          34         3
                          23         4
                          20         5
                          18         6
                           4         7
                           4         8
                           1         9
                           6        10
                           1        11
                           3        12
                           1        13
                           1        14
                           5        15
                           5        20
                           1        21
                           1        25
                           1        27
                           2        30
                           1        40
                           1        45
                           1        60
                           1        99
                         226       888  888. Don't Know
                          51         .
                        2847        .a  .a. Did not receive service
                           5        .m  .m. Missing
                          56        .s  .s. Skipped by participant
                           5        .w  .w. Answered 6 mon survey at 6
                                        wk visit
                         699        .x  .x. No 6 week survey

-------------------------------------------------------------------------------
w6_210                                      210: HTC: Source of transport money
-------------------------------------------------------------------------------

                type:  numeric (byte)
               label:  hftsource

               range:  [1,6]                        units:  1
       unique values:  6                        missing .:  51/4168
     unique mv codes:  8                       missing .*:  3898/4168

          tabulation:  Freq.   Numeric  Label
                          19         1  1. Cutting down on other
                                        expenses
                         107         2  2. Savings
                          11         3  3. Borrowing
                           3         4  4. Selling assets
                          56         5  5. Donation
                          23         6  6. Other
                          51         .
                        2847        .a  .a. Did not receive service
                          21        .b  .b. Did not pay for transport
                         310        .c  .c. Own car, walked, biked
                           5        .m  .m. Missing
                          11        .s  .s. Skipped by participant
                           5        .w  .w. Answered 6 mon survey at 6
                                        wk visit
                         699        .x  .x. No 6 week survey

-------------------------------------------------------------------------------
w6_211                     211: HTC: What respondent would be doing if not at health facility
-------------------------------------------------------------------------------
```

```
                  type:  numeric (byte)
                 label:  hfnot

                 range:  [1,6]                        units:  1
         unique values:  6                        missing .:  51/4168
       unique mv codes:  6                       missing .*:  3568/4168

            tabulation:  Freq.   Numeric  Label
                           113         1  1. Paid employment
                           118         2  2. Own business
                           119         3  3. Unpaid work/Housework
                            53         4  4. In school
                           108         5  5. Resting/No specific activity
                            38         6  6. Other
                            51         .
                          2847        .a  .a. Did not receive service
                             5        .m  .m. Missing
                            12        .s  .s. Skipped by participant
                             5        .w  .w. Answered 6 mon survey at 6
                                          wk visit
                           699        .x  .x. No 6 week survey
```

```
w6_300        300: HIV Care/Trt: Respondent understands to only include services from last 6 w
```

```
                  type:  numeric (byte)
                 label:  yesnoserv

                 range:  [1,1]                        units:  1
         unique values:  1                        missing .:  51/4168
       unique mv codes:  6                       missing .*:  3796/4168

            tabulation:  Freq.   Numeric  Label
                           321         1  1. Yes
                            51         .
                          3083        .a  .a. Did not receive service
                             7        .m  .m. Missing
                             2        .s  .s. Skipped by participant
                             5        .w  .w. Answered 6 mon survey at 6
                                          wk visit
                           699        .x  .x. No 6 week survey
```

```
w6_301                                 301: HIV Care/Trt: numservber times to health facility
```

```
                  type:  numeric (byte)
                 label:  numserv, but 6 nonmissing values are not labeled

                 range:  [1,6]                        units:  1
         unique values:  6                        missing .:  51/4168
       unique mv codes:  6                       missing .*:  3826/4168

            tabulation:  Freq.   Numeric  Label
                           159         1
                            70         2
                            29         3
                            16         4
                             7         5
                            10         6
                            51         .
                          3083        .a  .a. Did not receive service
                             7        .m  .m. Missing
                            32        .s  .s. Skipped by participant
                             5        .w  .w. Answered 6 mon survey at 6
                                          wk visit
                           699        .x  .x. No 6 week survey
```

```
w6_302                                        302: HIV Care/Trt: Health facility name
-------------------------------------------------------------------------------------

                  type:  string (str43)

         unique values:  101                      missing "":  801/4168

              examples:  "."
                         "."
                         "."
                         "."

               warning:  variable has embedded blanks

-------------------------------------------------------------------------------------
w6_303                                        303: HIV Care/Trt: Alone or accompanied
-------------------------------------------------------------------------------------

                  type:  numeric (byte)
                 label:  hfpeople

                 range:  [1,3]                         units:  1
         unique values:  3                         missing .:  51/4168
       unique mv codes:  6                        missing .*:  3832/4168

            tabulation:  Freq.   Numeric  Label
                           205         1  1. Alone
                            57         2  2. Spouse/Partner
                            23         3  3. Someone else
                            51         .
                          3083        .a  .a. Did not receive service
                             5        .m  .m. Missing
                            40        .s  .s. Skipped by participant
                             5        .w  .w. Answered 6 mon survey at 6
                                          wk visit
                           699        .x  .x. No 6 week survey

-------------------------------------------------------------------------------------
w6_304                                     304: HIV Care/Trt: Satisfied with services
-------------------------------------------------------------------------------------

                  type:  numeric (byte)
                 label:  hfsat

                 range:  [1,3]                         units:  1
         unique values:  3                         missing .:  51/4168
       unique mv codes:  6                        missing .*:  3828/4168

            tabulation:  Freq.   Numeric  Label
                            20         1  1. Not satisfied
                             5         2  2. Neither
                           264         3  3. Satisfied
                            51         .
                          3083        .a  .a. Did not receive service
                             5        .m  .m. Missing
                            36        .s  .s. Skipped by participant
                             5        .w  .w. Answered 6 mon survey at 6
                                          wk visit
                           699        .x  .x. No 6 week survey

-------------------------------------------------------------------------------------
w6_305                          305: HIV Care/Trt:  Waiting time at health facility (min)
-------------------------------------------------------------------------------------

                  type:  numeric (int)
                 label:  numserv, but 51 nonmissing values are not labeled

                 range:  [1,855]                       units:  1
         unique values:  51                        missing .:  51/4168
       unique mv codes:  6                        missing .*:  3832/4168
```

```
           examples:  .a    .a. Did not receive service
                      .a    .a. Did not receive service
                      .a    .a. Did not receive service
                      .a    .a. Did not receive service

-------------------------------------------------------------------------------------------
w6_306                                                  306: HIV Care/Trt: TB testing
-------------------------------------------------------------------------------------------

               type:  numeric (byte)
              label:  yesnoserv

              range:  [0,1]                        units:  1
      unique values:  2                        missing .:  51/4168
    unique mv codes:  6                       missing .*:  3801/4168

         tabulation:  Freq.   Numeric  Label
                        255         0  0. No
                         61         1  1. Yes
                         51         .
                       3083        .a  .a. Did not receive service
                          5        .m  .m. Missing
                          9        .s  .s. Skipped by participant
                          5        .w  .w. Answered 6 mon survey at 6
                                       wk visit
                        699        .x  .x. No 6 week survey

-------------------------------------------------------------------------------------------
w6_307                                                 307: HIV Care/Trt: CD4 testing
-------------------------------------------------------------------------------------------

               type:  numeric (byte)
              label:  yesnoserv

              range:  [0,88]                       units:  1
      unique values:  3                        missing .:  51/4168
    unique mv codes:  6                       missing .*:  3800/4168

         tabulation:  Freq.   Numeric  Label
                        170         0  0. No
                        146         1  1. Yes
                          1        88  88. Don't Know
                         51         .
                       3083        .a  .a. Did not receive service
                          5        .m  .m. Missing
                          8        .s  .s. Skipped by participant
                          5        .w  .w. Answered 6 mon survey at 6
                                       wk visit
                        699        .x  .x. No 6 week survey

-------------------------------------------------------------------------------------------
w6_308                                           308: HIV Care/Trt: Eligible for ARV
-------------------------------------------------------------------------------------------

               type:  numeric (byte)
              label:  w6_308

              range:  [0,88]                       units:  1
      unique values:  3                        missing .:  51/4168
    unique mv codes:  7                       missing .*:  3969/4168

         tabulation:  Freq.   Numeric  Label
                         55         0  0. No
                         91         1  1. Yes
                          2        88  88. Don't Know
                         51         .
                       3083        .a  .a. Did not receive service
                        170        .b  .b. Have not received CD4
                                       testing
```

```
                          5        .m  .m. Missing
                          7        .s  .s. Skipped by participant
                          5        .w  .w. Answered 6 mon survey at 6
                                       wk visit
                        699        .x  .x. No 6 week survey

-------------------------------------------------------------------------------
w6_309                                       309: HIV Care/Trt: Initiated ARV
-------------------------------------------------------------------------------

                  type:  numeric (byte)
                 label:  w6_309

                 range:  [0,1]                        units:  1
         unique values:  2                        missing .:  51/4168
       unique mv codes:  8                       missing .*:  4024/4168

            tabulation:  Freq.   Numeric  Label
                            10         0  0. No
                            83         1  1. Yes
                            51         .  
                          3083        .a  .a. Did not receive service
                           170        .b  .b. Have not received CD4
                                          testing
                            55        .c  .c. Not eligible for ARV
                             5        .m  .m. Missing
                             7        .s  .s. Skipped by participant
                             5        .w  .w. Answered 6 mon survey at 6
                                          wk visit
                           699        .x  .x. No 6 week survey

-------------------------------------------------------------------------------
w6_310                     310: HIV Care/Trt: numservber missed follow-ups since started ARV
-------------------------------------------------------------------------------

                  type:  numeric (byte)
                 label:  w6_310, but 2 nonmissing values are not labeled

                 range:  [0,2]                        units:  1
         unique values:  3                        missing .:  51/4168
       unique mv codes:  9                       missing .*:  4034/4168

            tabulation:  Freq.   Numeric  Label
                            81         0  0. Never
                             1         1  
                             1         2  
                            51         .  
                          3083        .a  .a. Did not receive service
                           170        .b  .b. Have not received CD4
                                          testing
                            55        .c  .c. Not eligible for ARV
                            10        .d  .d. Did not initiate ARV
                             5        .m  .m. Missing
                             7        .s  .s. Skipped by participant
                             5        .w  .w. Answered 6 mon survey at 6
                                          wk visit
                           699        .x  .x. No 6 week survey

-------------------------------------------------------------------------------
w6_311                         311: HIV Care/Trt: numservber missed doses ARV
-------------------------------------------------------------------------------

                  type:  numeric (byte)
                 label:  w6_311, but 4 nonmissing values are not labeled

                 range:  [0,5]                        units:  1
         unique values:  5                        missing .:  51/4168
       unique mv codes:  9                       missing .*:  4034/4168

            tabulation:  Freq.   Numeric  Label
```

```
                    68         0  0. Never
                     8         1
                     5         2
                     1         4
                     1         5
                    51         .
                  3083        .a  .a. Did not receive service
                   170        .b  .b. Have not received CD4
                                      testing
                    55        .c  .c. Not eligible for ARV
                    10        .d  .d. Did not initiate ARV
                     5        .m  .m. Missing
                     7        .s  .s. Skipped by participant
                     5        .w  .w. Answered 6 mon survey at 6
                                      wk visit
                   699        .x  .x. No 6 week survey

-----------------------------------------------------------------------------------------------
w6_312                         312: HIV Care/Trt: Participated in psycho-social support groups
-----------------------------------------------------------------------------------------------

                  type:  numeric (byte)
                 label:  yesnoserv

                 range:  [0,1]                        units:  1
         unique values:  2                        missing .:  51/4168
       unique mv codes:  6                       missing .*:  3801/4168

            tabulation:  Freq.   Numeric  Label
                           239         0  0. No
                            77         1  1. Yes
                            51         .
                          3083        .a  .a. Did not receive service
                             5        .m  .m. Missing
                             9        .s  .s. Skipped by participant
                             5        .w  .w. Answered 6 mon survey at 6
                                              wk visit
                           699        .x  .x. No 6 week survey

-----------------------------------------------------------------------------------------------
w6_313                                 313: HIV Care/Trt: Travel time to health facility (min)
-----------------------------------------------------------------------------------------------

                  type:  numeric (int)
                 label:  hfttime, but 33 nonmissing values are not labeled

                 range:  [2,888]                      units:  1
         unique values:  34                       missing .:  51/4168
       unique mv codes:  6                       missing .*:  3830/4168

              examples:  .a    .a. Did not receive service
                         .a    .a. Did not receive service
                         .a    .a. Did not receive service
                         .a    .a. Did not receive service

-----------------------------------------------------------------------------------------------
w6_314                                        314: HIV Care/Trt: Travel mode to health faciity
-----------------------------------------------------------------------------------------------

                  type:  numeric (byte)
                 label:  hftmode

                 range:  [1,7]                        units:  1
         unique values:  7                        missing .:  51/4168
       unique mv codes:  6                       missing .*:  3830/4168

            tabulation:  Freq.   Numeric  Label
                            88         1  1. Bus
                            26         2  2. Taxi
                             3         3  3. Someone drove in private car
```

```
                          4         4  4. Drove self
                        151         5  5. Walked
                         14         6  6. Bicycle
                          1         7  7. Other
                         51         .
                       3083        .a  .a. Did not receive service
                          5        .m  .m. Missing
                         38        .s  .s. Skipped by participant
                          5        .w  .w. Answered 6 mon survey at 6
                                       wk visit
                        699        .x  .x. No 6 week survey

-------------------------------------------------------------------------------
w6_315                                      315: HIV Care/Trt: Transport cost
-------------------------------------------------------------------------------

                  type:  numeric (byte)
                 label:  hftcost, but 20 nonmissing values are not labeled

                 range:  [0,93]                       units:  1
         unique values:  21                       missing .:  51/4168
       unique mv codes:  7                       missing .*:  3993/4168

            tabulation:  Freq.   Numeric  Label
                            11         0  0. Nothing
                             1         1
                             7         2
                            21         3
                            16         4
                            21         5
                             2         6
                             4         7
                             2         8
                             2         9
                            10        10
                             2        12
                             3        15
                             1        18
                            11        20
                             2        25
                             4        30
                             1        35
                             1        40
                             1        50
                             1        93
                            51         .
                          3083        .a  .a. Did not receive service
                           169        .b  .b. Own car, walked, biked
                             5        .m  .m. Missing
                            32        .s  .s. Skipped by participant
                             5        .w  .w. Answered 6 mon survey at 6
                                          wk visit
                           699        .x  .x. No 6 week survey

-------------------------------------------------------------------------------
w6_316                               316: HIV Care/Trt: Distance traveled (km)
-------------------------------------------------------------------------------

                  type:  numeric (int)
                 label:  hftkm, but 16 nonmissing values are not labeled

                 range:  [0,888]                      units:  1
         unique values:  18                       missing .:  51/4168
       unique mv codes:  6                       missing .*:  3836/4168

            tabulation:  Freq.   Numeric  Label
                            22         0  0. <1
                            19         1
                            22         2
                            20         3
```

```
                      10         4
                      12         5
                       7         6
                       4         7
                       3         8
                       4        10
                       1        12
                       1        13
                       1        14
                       3        15
                       2        20
                       2        45
                       1        60
                     147       888  888. Don't Know
                      51         .
                    3083        .a  .a. Did not receive service
                       5        .m  .m. Missing
                      44        .s  .s. Skipped by participant
                       5        .w  .w. Answered 6 mon survey at 6
                                    wk visit
                     699        .x  .x. No 6 week survey
```

----
w6_317   317: HIV Care/Trt: Source of transport money

```
                  type:  numeric (byte)
                 label:  hftsource

                 range:  [1,6]                        units:  1
         unique values:  6                        missing .:  51/4168
       unique mv codes:  8                       missing .*:  4003/4168

            tabulation:  Freq.   Numeric  Label
                            13         1  1. Cutting down on other
                                          expenses
                            54         2  2. Savings
                             4         3  3. Borrowing
                             2         4  4. Selling assets
                            35         5  5. Donation
                             6         6  6. Other
                            51         .
                          3083        .a  .a. Did not receive service
                            11        .b  .b. Did not pay for transport
                           169        .c  .c. Own car, walked, biked
                             5        .m  .m. Missing
                            31        .s  .s. Skipped by participant
                             5        .w  .w. Answered 6 mon survey at 6
                                          wk visit
                           699        .x  .x. No 6 week survey
```

----
w6_318   318: HIV Care/Trt: What respondent would be doing if not at health facility
----

```
                  type:  numeric (byte)
                 label:  hfnot

                 range:  [1,6]                        units:  1
         unique values:  6                        missing .:  51/4168
       unique mv codes:  6                       missing .*:  3832/4168

            tabulation:  Freq.   Numeric  Label
                            46         1  1. Paid employment
                            69         2  2. Own business
                            78         3  3. Unpaid work/Housework
                            13         4  4. In school
                            63         5  5. Resting/No specific activity
                            16         6  6. Other
                            51         .
```

```
                     3083        .a  .a. Did not receive service
                        5        .m  .m. Missing
                       40        .s  .s. Skipped by participant
                        5        .w  .w. Answered 6 mon survey at 6
                                     wk visit
                      699        .x  .x. No 6 week survey

-----------------------------------------------------------------------------------------------
w6_400        400: STI Care/Trt: Respondent understands to only include services from last 6 w
-----------------------------------------------------------------------------------------------

                  type:  numeric (byte)
                 label:  yesnoserv

                 range:  [1,1]                        units:  1
         unique values:  1                        missing .:  51/4168
       unique mv codes:  6                       missing .*:  3980/4168

            tabulation:  Freq.   Numeric  Label
                           137         1  1. Yes
                            51         .
                          3263        .a  .a. Did not receive service
                             5        .m  .m. Missing
                             8        .s  .s. Skipped by participant
                             5        .w  .w. Answered 6 mon survey at 6
                                          wk visit
                           699        .x  .x. No 6 week survey

-----------------------------------------------------------------------------------------------
w6_401                               401: STI Care/Trt: numservber times to health facility
-----------------------------------------------------------------------------------------------

                  type:  numeric (byte)
                 label:  numserv, but 6 nonmissing values are not labeled

                 range:  [1,6]                        units:  1
         unique values:  6                        missing .:  51/4168
       unique mv codes:  6                       missing .*:  3992/4168

            tabulation:  Freq.   Numeric  Label
                            75         1
                            26         2
                            15         3
                             3         4
                             4         5
                             2         6
                            51         .
                          3263        .a  .a. Did not receive service
                             5        .m  .m. Missing
                            20        .s  .s. Skipped by participant
                             5        .w  .w. Answered 6 mon survey at 6
                                          wk visit
                           699        .x  .x. No 6 week survey

-----------------------------------------------------------------------------------------------
w6_402                                           402: STI Care/Trt: Health facility name
-----------------------------------------------------------------------------------------------

                  type:  string (str43)

         unique values:  65                       missing "":  755/4168

              examples:  "."
                         "."
                         "."
                         "."

               warning:  variable has embedded blanks

-----------------------------------------------------------------------------------------------
```

```
w6_403                                          403: STI Care/Trt: Alone or accompanied
-----------------------------------------------------------------------------------------------

                  type:  numeric (byte)
                 label:  hfpeople

                 range:  [1,3]                        units:  1
         unique values:  3                        missing .:  51/4168
       unique mv codes:  6                       missing .*:  3985/4168

            tabulation:  Freq.   Numeric  Label
                            86         1  1. Alone
                            31         2  2. Spouse/Partner
                            15         3  3. Someone else
                            51         .
                          3263        .a  .a. Did not receive service
                             5        .m  .m. Missing
                            13        .s  .s. Skipped by participant
                             5        .w  .w. Answered 6 mon survey at 6
                                          wk visit
                           699        .x  .x. No 6 week survey

-----------------------------------------------------------------------------------------------
w6_404                                       404: STI Care/Trt: Satisfied with services
-----------------------------------------------------------------------------------------------

                  type:  numeric (byte)
                 label:  hfsat

                 range:  [1,3]                        units:  1
         unique values:  3                        missing .:  51/4168
       unique mv codes:  6                       missing .*:  3986/4168

            tabulation:  Freq.   Numeric  Label
                            15         1  1. Not satisfied
                             6         2  2. Neither
                           110         3  3. Satisfied
                            51         .
                          3263        .a  .a. Did not receive service
                             5        .m  .m. Missing
                            14        .s  .s. Skipped by participant
                             5        .w  .w. Answered 6 mon survey at 6
                                          wk visit
                           699        .x  .x. No 6 week survey

-----------------------------------------------------------------------------------------------
w6_405                          405: STI Care/Trt:  Waiting time at health facility (min)
-----------------------------------------------------------------------------------------------

                  type:  numeric (int)
                 label:  numserv, but 28 nonmissing values are not labeled

                 range:  [2,1800]                     units:  1
         unique values:  28                       missing .:  51/4168
       unique mv codes:  6                       missing .*:  3991/4168

            tabulation:  Freq.   Numeric  Label
                             2         2
                             1         3
                             5         5
                             2         6
                             1         8
                             4        10
                             6        15
                             6        20
                            30        30
                             1        35
                             4        40
                             1        41
                             4        45
```

```
                              2        50
                             14        60
                              1        63
                              1        70
                             12        90
                             10       120
                              1       121
                              1       150
                              1       160
                              7       180
                              5       240
                              1       250
                              1       300
                              1       480
                              1      1800
                             51         .
                           3263        .a  .a. Did not receive service
                              5        .m  .m. Missing
                             19        .s  .s. Skipped by participant
                              5        .w  .w. Answered 6 mon survey at 6
                                           wk visit
                            699        .x  .x. No 6 week survey

-----------------------------------------------------------------------------------------------
w6_406                                              406: STI Care/Trt: Need medicine for STD
-----------------------------------------------------------------------------------------------

                  type:  numeric (byte)
                 label:  yesnoserv

                 range:  [0,1]                        units:  1
         unique values:  2                        missing .:  51/4168
       unique mv codes:  6                       missing .*:  3989/4168

            tabulation:  Freq.   Numeric  Label
                            67         0  0. No
                            61         1  1. Yes
                            51         .
                          3263        .a  .a. Did not receive service
                             5        .m  .m. Missing
                            17        .s  .s. Skipped by participant
                             5        .w  .w. Answered 6 mon survey at 6
                                          wk visit
                           699        .x  .x. No 6 week survey

-----------------------------------------------------------------------------------------------
w6_407                                            407: STI Care/Trt: Place obtained medicine
-----------------------------------------------------------------------------------------------

                  type:  numeric (byte)
                 label:  w6_407

                 range:  [1,3]                        units:  1
         unique values:  3                        missing .:  51/4168
       unique mv codes:  7                       missing .*:  4054/4168

            tabulation:  Freq.   Numeric  Label
                            46         1  1. At health facility
                            12         2  2. Somewhere else
                             5         3  3. Did not get medicine
                            51         .
                          3263        .a  .a. Did not receive service
                            67        .b  .b. Did not need medicine
                             5        .m  .m. Missing
                            15        .s  .s. Skipped by participant
                             5        .w  .w. Answered 6 mon survey at 6
                                          wk visit
                           699        .x  .x. No 6 week survey

-----------------------------------------------------------------------------------------------
```

```
w6_408                                   408: STI Care/Trt: Cost of medicine
------------------------------------------------------------------------------------------

                  type:  numeric (byte)
                 label:  w6_408, but 12 nonmissing values are not labeled

                 range:  [0,100]                      units:  1
         unique values:  13                       missing .:  51/4168
       unique mv codes:  8                       missing .*:  4060/4168

           tabulation:  Freq.   Numeric  Label
                           34         0  0. Nothing
                            1         4
                            3        10
                            1        11
                            1        14
                            1        15
                            6        20
                            2        30
                            1        37
                            2        50
                            1        55
                            1        56
                            3       100
                           51         .
                         3263        .a  .a. Did not receive service
                           67        .b  .b. Did not need medicine
                            5        .c  .c. Did not get medicine
                            5        .m  .m. Missing
                           16        .s  .s. Skipped by participant
                            5        .w  .w. Answered 6 mon survey at 6
                                         wk visit
                          699        .x  .x. No 6 week survey

------------------------------------------------------------------------------------------
w6_409                    409: STI Care/Trt: Travel time to health facility (min)
------------------------------------------------------------------------------------------

                  type:  numeric (int)
                 label:  hfttime, but 23 nonmissing values are not labeled

                 range:  [1,888]                      units:  1
         unique values:  24                       missing .:  51/4168
       unique mv codes:  6                       missing .*:  3993/4168

           tabulation:  Freq.   Numeric  Label
                            1         1
                            1         3
                            5         5
                            2         6
                            1         8
                           10        10
                            1        12
                            4        15
                            7        20
                            2        25
                           33        30
                            2        35
                            8        40
                           10        45
                            1        46
                            2        50
                           20        60
                            1        70
                            1        75
                            5        90
                            1       120
                            1       140
                            1       218
                            4       888  888. Don't Know
```

```
                            51          .
                          3263         .a  .a. Did not receive service
                             5         .m  .m. Missing
                            21         .s  .s. Skipped by participant
                             5         .w  .w. Answered 6 mon survey at 6
                                           wk visit
                           699         .x  .x. No 6 week survey

-------------------------------------------------------------------------------
w6_410                               410: STI Care/Trt: Travel mode to health faciity
-------------------------------------------------------------------------------

                  type:  numeric (byte)
                 label:  hftmode

                 range:  [1,6]                        units:  1
         unique values:  6                        missing .:  51/4168
       unique mv codes:  6                       missing .*:  3991/4168

            tabulation:  Freq.   Numeric  Label
                            29         1  1. Bus
                            18         2  2. Taxi
                             1         3  3. Someone drove in private car
                             4         4  4. Drove self
                            62         5  5. Walked
                            12         6  6. Bicycle
                            51         .
                          3263        .a  .a. Did not receive service
                             5        .m  .m. Missing
                            19        .s  .s. Skipped by participant
                             5        .w  .w. Answered 6 mon survey at 6
                                          wk visit
                           699        .x  .x. No 6 week survey

-------------------------------------------------------------------------------
w6_411                                          411: STI Care/Trt: Transport cost
-------------------------------------------------------------------------------

                  type:  numeric (byte)
                 label:  hftcost, but 11 nonmissing values are not labeled

                 range:  [0,60]                       units:  1
         unique values:  12                       missing .:  51/4168
       unique mv codes:  7                       missing .*:  4068/4168

            tabulation:  Freq.   Numeric  Label
                             3         0  0. Nothing
                             3         2
                            10         3
                             5         4
                             8         5
                             6        10
                             2        15
                             2        20
                             2        25
                             5        30
                             2        40
                             1        60
                            51         .
                          3263        .a  .a. Did not receive service
                            78        .b  .b. Own car, walked, biked
                             5        .m  .m. Missing
                            18        .s  .s. Skipped by participant
                             5        .w  .w. Answered 6 mon survey at 6
                                          wk visit
                           699        .x  .x. No 6 week survey

-------------------------------------------------------------------------------
w6_412                                  412: STI Care/Trt: Distance traveled (km)
-------------------------------------------------------------------------------
```

```
                  type:  numeric (byte)
                 label:  hftkm, but 14 nonmissing values are not labeled

                 range:  [0,88]                       units:  1
         unique values:  15                       missing .:  51/4168
       unique mv codes:  6                       missing .*:  3993/4168

            tabulation:  Freq.   Numeric  Label
                            12         0  0. <1
                             9         1
                            16         2
                             6         3
                             6         4
                             2         5
                             4         6
                             3         7
                             1         8
                             2        10
                             1        13
                             1        20
                             1        30
                             1        60
                            59        88
                            51         .
                          3263        .a  .a. Did not receive service
                             5        .m  .m. Missing
                            21        .s  .s. Skipped by participant
                             5        .w  .w. Answered 6 mon survey at 6
                                          wk visit
                           699        .x  .x. No 6 week survey

-------------------------------------------------------------------------------
w6_413                                   413: STI Care/Trt: Source of transport money
-------------------------------------------------------------------------------

                  type:  numeric (byte)
                 label:  hftsource

                 range:  [1,5]                        units:  1
         unique values:  5                        missing .:  51/4168
       unique mv codes:  8                       missing .*:  4071/4168

            tabulation:  Freq.   Numeric  Label
                             3         1  1. Cutting down on other
                                          expenses
                            23         2  2. Savings
                             3         3  3. Borrowing
                             1         4  4. Selling assets
                            16         5  5. Donation
                            51         .
                          3263        .a  .a. Did not receive service
                             3        .b  .b. Did not pay for transport
                            78        .c  .c. Own car, walked, biked
                             5        .m  .m. Missing
                            18        .s  .s. Skipped by participant
                             5        .w  .w. Answered 6 mon survey at 6
                                          wk visit
                           699        .x  .x. No 6 week survey

-------------------------------------------------------------------------------
w6_414            414: STI Care/Trt: What respondent would be doing if not at health facility
-------------------------------------------------------------------------------

                  type:  numeric (byte)
                 label:  hfnot

                 range:  [1,6]                        units:  1
         unique values:  6                        missing .:  51/4168
       unique mv codes:  6                       missing .*:  3990/4168
```

```
          tabulation:  Freq.   Numeric  Label
                          27         1  1. Paid employment
                          25         2  2. Own business
                          27         3  3. Unpaid work/Housework
                          11         4  4. In school
                          28         5  5. Resting/No specific activity
                           9         6  6. Other
                          51         .
                        3263        .a  .a. Did not receive service
                           5        .m  .m. Missing
                          18        .s  .s. Skipped by participant
                           5        .w  .w. Answered 6 mon survey at 6
                                        wk visit
                         699        .x  .x. No 6 week survey

-----------------------------------------------------------------------------------------------
w6_500_f           500: FP: Respondent understands to only include services from last 6 weeks
-----------------------------------------------------------------------------------------------

                type:  numeric (byte)
               label:  yesnoserv

               range:  [1,1]                        units:  1
       unique values:  1                        missing .:  51/4168
     unique mv codes:  7                       missing .*:  3903/4168

          tabulation:  Freq.   Numeric  Label
                         214         1  1. Yes
                          51         .
                        1565        .a  .a. Did not receive service
                        1630        .g  .g. Gender skip pattern
                           2        .m  .m. Missing
                           2        .s  .s. Skipped by participant
                           5        .w  .w. Answered 6 mon survey at 6
                                        wk visit
                         699        .x  .x. No 6 week survey

-----------------------------------------------------------------------------------------------
w6_501_f                                      501: FP: numservber times to health facility
-----------------------------------------------------------------------------------------------

                type:  numeric (byte)
               label:  numserv, but 6 nonmissing values are not labeled

               range:  [1,6]                        units:  1
       unique values:  6                        missing .:  51/4168
     unique mv codes:  7                       missing .*:  3918/4168

          tabulation:  Freq.   Numeric  Label
                         173         1
                          15         2
                           8         3
                           1         4
                           1         5
                           1         6
                          51         .
                        1565        .a  .a. Did not receive service
                        1630        .g  .g. Gender skip pattern
                           2        .m  .m. Missing
                          17        .s  .s. Skipped by participant
                           5        .w  .w. Answered 6 mon survey at 6
                                        wk visit
                         699        .x  .x. No 6 week survey

-----------------------------------------------------------------------------------------------
w6_502_f                                                     502: FP: Health facility name
-----------------------------------------------------------------------------------------------

                type:  string (str56)
```

```
        unique values:  75                       missing "":  755/4168

             examples:  "."
                        "."
                        "."
                        "."

              warning:  variable has embedded blanks

-------------------------------------------------------------------------------------------
w6_503_f                                                  503: FP: Alone or accompanied
-------------------------------------------------------------------------------------------

                 type:  numeric (byte)
                label:  hfpeople

                range:  [1,3]                        units:  1
        unique values:  3                        missing .:  51/4168
      unique mv codes:  7                       missing .*:  3916/4168

           tabulation:  Freq.   Numeric  Label
                          168         1  1. Alone
                           21         2  2. Spouse/Partner
                           12         3  3. Someone else
                           51         .
                         1565        .a  .a. Did not receive service
                         1630        .g  .g. Gender skip pattern
                            2        .m  .m. Missing
                           15        .s  .s. Skipped by participant
                            5        .w  .w. Answered 6 mon survey at 6
                                         wk visit
                          699        .x  .x. No 6 week survey

-------------------------------------------------------------------------------------------
w6_504_f                                               504: FP: Satisfied with services
-------------------------------------------------------------------------------------------

                 type:  numeric (byte)
                label:  hfsat

                range:  [1,3]                        units:  1
        unique values:  3                        missing .:  51/4168
      unique mv codes:  7                       missing .*:  3915/4168

           tabulation:  Freq.   Numeric  Label
                           10         1  1. Not satisfied
                            4         2  2. Neither
                          188         3  3. Satisfied
                           51         .
                         1565        .a  .a. Did not receive service
                         1630        .g  .g. Gender skip pattern
                            2        .m  .m. Missing
                           14        .s  .s. Skipped by participant
                            5        .w  .w. Answered 6 mon survey at 6
                                         wk visit
                          699        .x  .x. No 6 week survey

-------------------------------------------------------------------------------------------
w6_505_f                                 505: FP:  Waiting time at health facility (min)
-------------------------------------------------------------------------------------------

                 type:  numeric (int)
                label:  numserv, but 35 nonmissing values are not labeled

                range:  [1,420]                      units:  1
        unique values:  35                       missing .:  51/4168
      unique mv codes:  7                       missing .*:  3920/4168

             examples:  .a    .a. Did not receive service
```

```
                    .a    .a. Did not receive service
                    .g    .g. Gender skip pattern
                    .g    .g. Gender skip pattern

-------------------------------------------------------------------------------
w6_506_f                                            506: FP: Currently pregnant
-------------------------------------------------------------------------------

                  type:  numeric (byte)
                 label:  yesnoserv

                 range:  [0,88]                       units:  1
         unique values:  3                        missing .:  51/4168
       unique mv codes:  7                       missing .*:  3904/4168

            tabulation:  Freq.   Numeric  Label
                           209         0  0. No
                             2         1  1. Yes
                             2        88  88. Don't Know
                            51         .  
                          1565        .a  .a. Did not receive service
                          1630        .g  .g. Gender skip pattern
                             2        .m  .m. Missing
                             3        .s  .s. Skipped by participant
                             5        .w  .w. Answered 6 mon survey at 6
                                          wk visit
                           699        .x  .x. No 6 week survey

-------------------------------------------------------------------------------
w6_507_f                                            507: FP: Currently using FP
-------------------------------------------------------------------------------

                  type:  numeric (byte)
                 label:  w6_507

                 range:  [0,1]                        units:  1
         unique values:  2                        missing .:  51/4168
       unique mv codes:  8                       missing .*:  3905/4168

            tabulation:  Freq.   Numeric  Label
                            19         0  0. No
                           193         1  1. Yes
                            51         .  
                          1565        .a  .a. Did not receive service
                             2        .b  .b. Currently pregnant
                          1630        .g  .g. Gender skip pattern
                             2        .m  .m. Missing
                             2        .s  .s. Skipped by participant
                             5        .w  .w. Answered 6 mon survey at 6
                                          wk visit
                           699        .x  .x. No 6 week survey

-------------------------------------------------------------------------------
w6_508a_f                                 508: FP: Method: Female sterilization
-------------------------------------------------------------------------------

                  type:  numeric (byte)
                 label:  w6_508a

                 range:  [0,0]                        units:  1
         unique values:  1                        missing .:  51/4168
       unique mv codes:  9                       missing .*:  3924/4168

            tabulation:  Freq.   Numeric  Label
                           193         0  0. No
                            51         .  
                          1565        .a  .a. Did not receive service
                             2        .b  .b. Currently pregnant
                            19        .c  .c. Not using any method
                          1630        .g  .g. Gender skip pattern
```

```
                            2        .m  .m. Missing
                            2        .s  .s. Skipped by participant
                            5        .w  .w. Answered 6 mon survey at 6
                                         wk visit
                          699        .x  .x. No 6 week survey

--------------------------------------------------------------------------------------------
w6_508b_f                                             508: FP: Method: Male sterilization
--------------------------------------------------------------------------------------------

                  type:  numeric (byte)
                 label:  w6_508b

                 range:  [0,1]                        units:  1
         unique values:  2                        missing .:  51/4168
       unique mv codes:  9                       missing .*:  3924/4168

            tabulation:  Freq.   Numeric  Label
                          192         0  0. No
                            1         1  1. Yes
                           51         .
                         1565        .a  .a. Did not receive service
                            2        .b  .b. Currently pregnant
                           19        .c  .c. Not using any method
                         1630        .g  .g. Gender skip pattern
                            2        .m  .m. Missing
                            2        .s  .s. Skipped by participant
                            5        .w  .w. Answered 6 mon survey at 6
                                         wk visit
                          699        .x  .x. No 6 week survey

--------------------------------------------------------------------------------------------
w6_508c_f                                                          508: FP: Method: Pill
--------------------------------------------------------------------------------------------

                  type:  numeric (byte)
                 label:  w6_508c

                 range:  [0,1]                        units:  1
         unique values:  2                        missing .:  51/4168
       unique mv codes:  9                       missing .*:  3924/4168

            tabulation:  Freq.   Numeric  Label
                          162         0  0. No
                           31         1  1. Yes
                           51         .
                         1565        .a  .a. Did not receive service
                            2        .b  .b. Currently pregnant
                           19        .c  .c. Not using any method
                         1630        .g  .g. Gender skip pattern
                            2        .m  .m. Missing
                            2        .s  .s. Skipped by participant
                            5        .w  .w. Answered 6 mon survey at 6
                                         wk visit
                          699        .x  .x. No 6 week survey

--------------------------------------------------------------------------------------------
w6_508d_f                                                           508: FP: Method: IUD
--------------------------------------------------------------------------------------------

                  type:  numeric (byte)
                 label:  w6_508d

                 range:  [0,1]                        units:  1
         unique values:  2                        missing .:  51/4168
       unique mv codes:  9                       missing .*:  3924/4168

            tabulation:  Freq.   Numeric  Label
                          173         0  0. No
                           20         1  1. Yes
```

```
                       51           .
                     1565          .a  .a. Did not receive service
                        2          .b  .b. Currently pregnant
                       19          .c  .c. Not using any method
                     1630          .g  .g. Gender skip pattern
                        2          .m  .m. Missing
                        2          .s  .s. Skipped by participant
                        5          .w  .w. Answered 6 mon survey at 6
                                       wk visit
                      699          .x  .x. No 6 week survey

-------------------------------------------------------------------------------------------
w6_508e_f                                                  508: FP: Method: Injectables
-------------------------------------------------------------------------------------------

                  type:  numeric (byte)
                 label:  w6_508e

                 range:  [0,1]                        units:  1
         unique values:  2                        missing .:  51/4168
       unique mv codes:  9                       missing .*:  3924/4168

            tabulation:  Freq.   Numeric  Label
                           124         0  0. No
                            69         1  1. Yes
                            51         .
                          1565        .a  .a. Did not receive service
                             2        .b  .b. Currently pregnant
                            19        .c  .c. Not using any method
                          1630        .g  .g. Gender skip pattern
                             2        .m  .m. Missing
                             2        .s  .s. Skipped by participant
                             5        .w  .w. Answered 6 mon survey at 6
                                          wk visit
                           699        .x  .x. No 6 week survey

-------------------------------------------------------------------------------------------
w6_508f_f                                                     508: FP: Method: Implants
-------------------------------------------------------------------------------------------

                  type:  numeric (byte)
                 label:  w6_508f

                 range:  [0,1]                        units:  1
         unique values:  2                        missing .:  51/4168
       unique mv codes:  9                       missing .*:  3924/4168

            tabulation:  Freq.   Numeric  Label
                           131         0  0. No
                            62         1  1. Yes
                            51         .
                          1565        .a  .a. Did not receive service
                             2        .b  .b. Currently pregnant
                            19        .c  .c. Not using any method
                          1630        .g  .g. Gender skip pattern
                             2        .m  .m. Missing
                             2        .s  .s. Skipped by participant
                             5        .w  .w. Answered 6 mon survey at 6
                                          wk visit
                           699        .x  .x. No 6 week survey

-------------------------------------------------------------------------------------------
w6_508g_f                                                  508: FP: Method: Male condom
-------------------------------------------------------------------------------------------

                  type:  numeric (byte)
                 label:  w6_508g

                 range:  [0,1]                        units:  1
         unique values:  2                        missing .:  51/4168
```

```
      unique mv codes:  9                      missing .*:  3924/4168

           tabulation:  Freq.   Numeric  Label
                          167         0  0. No
                           26         1  1. Yes
                           51         .  
                         1565        .a  .a. Did not receive service
                            2        .b  .b. Currently pregnant
                           19        .c  .c. Not using any method
                         1630        .g  .g. Gender skip pattern
                            2        .m  .m. Missing
                            2        .s  .s. Skipped by participant
                            5        .w  .w. Answered 6 mon survey at 6
                                         wk visit
                          699        .x  .x. No 6 week survey

-----------------------------------------------------------------------------------------------
w6_508h_f                                                    508: FP: Method: Female condom
-----------------------------------------------------------------------------------------------

                 type:  numeric (byte)
                label:  w6_508h

                range:  [0,1]                        units:  1
        unique values:  2                        missing .:  51/4168
      unique mv codes:  9                       missing .*:  3924/4168

           tabulation:  Freq.   Numeric  Label
                          192         0  0. No
                            1         1  1. Yes
                           51         .  
                         1565        .a  .a. Did not receive service
                            2        .b  .b. Currently pregnant
                           19        .c  .c. Not using any method
                         1630        .g  .g. Gender skip pattern
                            2        .m  .m. Missing
                            2        .s  .s. Skipped by participant
                            5        .w  .w. Answered 6 mon survey at 6
                                         wk visit
                          699        .x  .x. No 6 week survey

-----------------------------------------------------------------------------------------------
w6_508i_f                                                        508: FP: Method: Diaphragm
-----------------------------------------------------------------------------------------------

                 type:  numeric (byte)
                label:  w6_508i

                range:  [0,1]                        units:  1
        unique values:  2                        missing .:  51/4168
      unique mv codes:  9                       missing .*:  3924/4168

           tabulation:  Freq.   Numeric  Label
                          190         0  0. No
                            3         1  1. Yes
                           51         .  
                         1565        .a  .a. Did not receive service
                            2        .b  .b. Currently pregnant
                           19        .c  .c. Not using any method
                         1630        .g  .g. Gender skip pattern
                            2        .m  .m. Missing
                            2        .s  .s. Skipped by participant
                            5        .w  .w. Answered 6 mon survey at 6
                                         wk visit
                          699        .x  .x. No 6 week survey

-----------------------------------------------------------------------------------------------
w6_508j_f                                                       508: FP: Method: Foam/Jelly
-----------------------------------------------------------------------------------------------
```

```
                  type:  numeric (byte)
                 label:  w6_508j

                 range:  [0,0]                        units:  1
         unique values:  1                        missing .:  51/4168
       unique mv codes:  9                       missing .*:  3924/4168

            tabulation:  Freq.   Numeric  Label
                           193         0  0. No
                            51         .
                          1565        .a  .a. Did not receive service
                             2        .b  .b. Currently pregnant
                            19        .c  .c. Not using any method
                          1630        .g  .g. Gender skip pattern
                             2        .m  .m. Missing
                             2        .s  .s. Skipped by participant
                             5        .w  .w. Answered 6 mon survey at 6
                                          wk visit
                           699        .x  .x. No 6 week survey

-----------------------------------------------------------------------------------------------
w6_508k_f                                    508: FP: Method: Lactational amenorrhea method
-----------------------------------------------------------------------------------------------

                  type:  numeric (byte)
                 label:  w6_508k

                 range:  [0,0]                        units:  1
         unique values:  1                        missing .:  51/4168
       unique mv codes:  9                       missing .*:  3924/4168

            tabulation:  Freq.   Numeric  Label
                           193         0  0. No
                            51         .
                          1565        .a  .a. Did not receive service
                             2        .b  .b. Currently pregnant
                            19        .c  .c. Not using any method
                          1630        .g  .g. Gender skip pattern
                             2        .m  .m. Missing
                             2        .s  .s. Skipped by participant
                             5        .w  .w. Answered 6 mon survey at 6
                                          wk visit
                           699        .x  .x. No 6 week survey

-----------------------------------------------------------------------------------------------
w6_508l_f                                                    508: FP: Method: Rhythm method
-----------------------------------------------------------------------------------------------

                  type:  numeric (byte)
                 label:  w6_508l

                 range:  [0,0]                        units:  1
         unique values:  1                        missing .:  51/4168
       unique mv codes:  9                       missing .*:  3924/4168

            tabulation:  Freq.   Numeric  Label
                           193         0  0. No
                            51         .
                          1565        .a  .a. Did not receive service
                             2        .b  .b. Currently pregnant
                            19        .c  .c. Not using any method
                          1630        .g  .g. Gender skip pattern
                             2        .m  .m. Missing
                             2        .s  .s. Skipped by participant
                             5        .w  .w. Answered 6 mon survey at 6
                                          wk visit
                           699        .x  .x. No 6 week survey

-----------------------------------------------------------------------------------------------
w6_508m_f                                                       508: FP: Method: Withdrawal
```

```
--------------------------------------------------------------------------------------------

                type:  numeric (byte)
               label:  w6_508m

               range:  [0,1]                        units:  1
       unique values:  2                        missing .:  51/4168
     unique mv codes:  9                       missing .*:  3924/4168

          tabulation:  Freq.   Numeric  Label
                         192         0  0. No
                           1         1  1. Yes
                          51         .
                        1565        .a  .a. Did not receive service
                           2        .b  .b. Currently pregnant
                          19        .c  .c. Not using any method
                        1630        .g  .g. Gender skip pattern
                           2        .m  .m. Missing
                           2        .s  .s. Skipped by participant
                           5        .w  .w. Answered 6 mon survey at 6
                                        wk visit
                         699        .x  .x. No 6 week survey

--------------------------------------------------------------------------------------------
w6_508n_f                                                      508: FP: Method: Other method
--------------------------------------------------------------------------------------------

                type:  numeric (byte)
               label:  w6_508n

               range:  [0,1]                        units:  1
       unique values:  2                        missing .:  51/4168
     unique mv codes:  9                       missing .*:  3924/4168

          tabulation:  Freq.   Numeric  Label
                         192         0  0. No
                           1         1  1. Yes
                          51         .
                        1565        .a  .a. Did not receive service
                           2        .b  .b. Currently pregnant
                          19        .c  .c. Not using any method
                        1630        .g  .g. Gender skip pattern
                           2        .m  .m. Missing
                           2        .s  .s. Skipped by participant
                           5        .w  .w. Answered 6 mon survey at 6
                                        wk visit
                         699        .x  .x. No 6 week survey

--------------------------------------------------------------------------------------------
w6_509_f                                    509: FP: numservber days forgot pill in past month
--------------------------------------------------------------------------------------------

                type:  numeric (byte)
               label:  w6_509, but 5 nonmissing values are not labeled

               range:  [0,5]                        units:  1
       unique values:  6                        missing .:  51/4168
     unique mv codes:  10                      missing .*:  4086/4168

          tabulation:  Freq.   Numeric  Label
                          20         0  0. Never
                           4         1
                           2         2
                           2         3
                           2         4
                           1         5
                          51         .
                        1565        .a  .a. Did not receive service
                           2        .b  .b. Currently pregnant
                          19        .c  .c. Not using any method
```

```
                      162        .d  .d. Not taking pill
                     1630        .g  .g. Gender skip pattern
                        2        .m  .m. Missing
                        2        .s  .s. Skipped by participant
                        5        .w  .w. Answered 6 mon survey at 6
                                         wk visit
                      699        .x  .x. No 6 week survey

----------------------------------------------------------------------------------------------
w6_510_f                                     510: FP: numservber times had sex without condom
----------------------------------------------------------------------------------------------

                  type:  numeric (byte)
                 label:  w6_510

                 range:  [.,.]                        units:  .
         unique values:  0                        missing .:  51/4168
       unique mv codes:  11                      missing .*:  4117/4168

            tabulation:  Freq.   Numeric  Label
                            51         .
                          1565        .a  .a. Did not receive service
                             2        .b  .b. Currently pregnant
                            19        .c  .c. Not using any method
                           166        .d  .d. Not currently using condoms
                            27        .e  .e. Programming skip error: Had
                                              to say yes to male and female
                                              condom to answer this question
                          1630        .g  .g. Gender skip pattern
                             2        .m  .m. Missing
                             2        .s  .s. Skipped by participant
                             5        .w  .w. Answered 6 mon survey at 6
                                              wk visit
                           699        .x  .x. No 6 week survey

----------------------------------------------------------------------------------------------
w6_511_f                                        511: FP: Travel time to health facility (min)
----------------------------------------------------------------------------------------------

                  type:  numeric (int)
                 label:  hfttime, but 20 nonmissing values are not labeled

                 range:  [1,888]                      units:  1
         unique values:  21                       missing .:  51/4168
       unique mv codes:  7                       missing .*:  3920/4168

            tabulation:  Freq.   Numeric  Label
                             1         1
                             1         3
                             9         5
                            19        10
                             1        11
                             1        12
                            12        15
                             1        16
                            25        20
                             2        25
                             1        28
                            44        30
                            12        40
                            13        45
                             3        50
                            30        60
                             4        90
                             6       120
                             2       130
                             2       180
                             8       888  888. Don't Know
                            51         .
                          1565        .a  .a. Did not receive service
```

```
                     1630        .g  .g. Gender skip pattern
                        2        .m  .m. Missing
                       19        .s  .s. Skipped by participant
                        5        .w  .w. Answered 6 mon survey at 6
                                     wk visit
                      699        .x  .x. No 6 week survey

--------------------------------------------------------------------------------------------
w6_512_f                                              512: FP: Travel mode to health faciity
--------------------------------------------------------------------------------------------

                  type:  numeric (byte)
                 label:  hftmode

                 range:  [1,6]                        units:  1
         unique values:  6                        missing .:  51/4168
       unique mv codes:  7                       missing .*:  3920/4168

            tabulation:  Freq.   Numeric  Label
                            83         1  1. Bus
                            12         2  2. Taxi
                             5         3  3. Someone drove in private car
                             1         4  4. Drove self
                            89         5  5. Walked
                             7         6  6. Bicycle
                            51         .
                          1565        .a  .a. Did not receive service
                          1630        .g  .g. Gender skip pattern
                             2        .m  .m. Missing
                            19        .s  .s. Skipped by participant
                             5        .w  .w. Answered 6 mon survey at 6
                                          wk visit
                           699        .x  .x. No 6 week survey

--------------------------------------------------------------------------------------------
w6_513_f                                                          513: FP: Transport cost
--------------------------------------------------------------------------------------------

                  type:  numeric (byte)
                 label:  hftcost, but 14 nonmissing values are not labeled

                 range:  [0,40]                       units:  1
         unique values:  15                       missing .:  51/4168
       unique mv codes:  8                       missing .*:  4018/4168

            tabulation:  Freq.   Numeric  Label
                             6         0  0. Nothing
                             2         1
                            20         2
                            20         3
                            11         4
                            14         5
                             4         6
                             1         7
                            10        10
                             1        12
                             2        15
                             4        20
                             2        25
                             1        30
                             1        40
                            51         .
                          1565        .a  .a. Did not receive service
                            97        .b  .b. Own car, walked, biked
                          1630        .g  .g. Gender skip pattern
                             2        .m  .m. Missing
                            20        .s  .s. Skipped by participant
                             5        .w  .w. Answered 6 mon survey at 6
                                          wk visit
                           699        .x  .x. No 6 week survey
```

```
--------------------------------------------------------------------------------------------
w6_514_f                                                   514: FP: Distance traveled (km)
--------------------------------------------------------------------------------------------

                  type:  numeric (int)
                 label:  hftkm, but 11 nonmissing values are not labeled

                 range:  [0,888]                      units:  1
         unique values:  13                       missing .:  51/4168
       unique mv codes:  7                       missing .*:  3966/4168

           tabulation:  Freq.   Numeric  Label
                           23         0  0. <1
                           14         1
                           13         2
                           15         3
                            7         4
                            7         5
                            2         6
                            2         7
                            1        23
                            1        30
                            1        40
                            1        50
                           64       888  888. Don't Know
                           51         .
                         1565        .a  .a. Did not receive service
                         1630        .g  .g. Gender skip pattern
                            2        .m  .m. Missing
                           65        .s  .s. Skipped by participant
                            5        .w  .w. Answered 6 mon survey at 6
                                         wk visit
                          699        .x  .x. No 6 week survey

--------------------------------------------------------------------------------------------
w6_515_f                                                514: FP: Source of transport money
--------------------------------------------------------------------------------------------

                  type:  numeric (byte)
                 label:  hftsource

                 range:  [1,6]                        units:  1
         unique values:  6                        missing .:  51/4168
       unique mv codes:  9                       missing .*:  4024/4168

           tabulation:  Freq.   Numeric  Label
                            8         1  1. Cutting down on other
                                         expenses
                           31         2  2. Savings
                            2         3  3. Borrowing
                            1         4  4. Selling assets
                           38         5  5. Donation
                           13         6  6. Other
                           51         .
                         1565        .a  .a. Did not receive service
                            6        .b  .b. Did not pay for transport
                           97        .c  .c. Own car, walked, biked
                         1630        .g  .g. Gender skip pattern
                            2        .m  .m. Missing
                           20        .s  .s. Skipped by participant
                            5        .w  .w. Answered 6 mon survey at 6
                                         wk visit
                          699        .x  .x. No 6 week survey

--------------------------------------------------------------------------------------------
w6_516_f                          516: FP: What respondent would be doing if not at health facility
--------------------------------------------------------------------------------------------

                  type:  numeric (byte)
```

```
                label:  hfnot

                range:  [1,6]                        units:  1
        unique values:  6                        missing .:  51/4168
      unique mv codes:  7                       missing .*:  3920/4168

           tabulation:  Freq.   Numeric  Label
                           22         1  1. Paid employment
                           41         2  2. Own business
                           83         3  3. Unpaid work/Housework
                            6         4  4. In school
                           39         5  5. Resting/No specific activity
                            6         6  6. Other
                           51         .
                         1565        .a  .a. Did not receive service
                         1630        .g  .g. Gender skip pattern
                            2        .m  .m. Missing
                           19        .s  .s. Skipped by participant
                            5        .w  .w. Answered 6 mon survey at 6
                                         wk visit
                          699        .x  .x. No 6 week survey

------------------------------------------------------------------------------------------------
w6_600_f          600: CCS: Respondent understands to only include services from last 6 weeks
------------------------------------------------------------------------------------------------

                 type:  numeric (byte)
                label:  yesnoserv

                range:  [1,1]                        units:  1
        unique values:  1                        missing .:  51/4168
      unique mv codes:  7                       missing .*:  3831/4168

           tabulation:  Freq.   Numeric  Label
                          286         1  1. Yes
                           51         .
                         1494        .a  .a. Did not receive service
                         1630        .g  .g. Gender skip pattern
                            2        .m  .m. Missing
                            1        .s  .s. Skipped by participant
                            5        .w  .w. Answered 6 mon survey at 6
                                         wk visit
                          699        .x  .x. No 6 week survey

------------------------------------------------------------------------------------------------
w6_601_f                                          601: CCS: numservber times to health facility
------------------------------------------------------------------------------------------------

                 type:  numeric (byte)
                label:  numserv, but 6 nonmissing values are not labeled

                range:  [1,6]                        units:  1
        unique values:  6                        missing .:  51/4168
      unique mv codes:  7                       missing .*:  3834/4168

           tabulation:  Freq.   Numeric  Label
                          234         1
                           38         2
                            6         3
                            2         4
                            2         5
                            1         6
                           51         .
                         1494        .a  .a. Did not receive service
                         1630        .g  .g. Gender skip pattern
                            2        .m  .m. Missing
                            4        .s  .s. Skipped by participant
                            5        .w  .w. Answered 6 mon survey at 6
                                         wk visit
                          699        .x  .x. No 6 week survey
```

```
-------------------------------------------------------------------------------------------
w6_602_f                                                  602: CCS: Health facility name
-------------------------------------------------------------------------------------------

                type:  string (str28)

       unique values:  64                      missing "":  755/4168

            examples:  "."
                       "."
                       "."
                       "."

             warning:  variable has embedded blanks

-------------------------------------------------------------------------------------------
w6_603_f                                                  603: CCS: Alone or accompanied
-------------------------------------------------------------------------------------------

                type:  numeric (byte)
               label:  hfpeople

               range:  [1,3]                         units:  1
       unique values:  3                         missing .:  51/4168
     unique mv codes:  7                        missing .*:  3835/4168

          tabulation:  Freq.   Numeric  Label
                         175         1  1. Alone
                          11         2  2. Spouse/Partner
                          96         3  3. Someone else
                          51         .
                        1494        .a  .a. Did not receive service
                        1630        .g  .g. Gender skip pattern
                           2        .m  .m. Missing
                           5        .s  .s. Skipped by participant
                           5        .w  .w. Answered 6 mon survey at 6
                                        wk visit
                         699        .x  .x. No 6 week survey

-------------------------------------------------------------------------------------------
w6_604_f                                                604: CCS: Satisfied with services
-------------------------------------------------------------------------------------------

                type:  numeric (byte)
               label:  hfsat

               range:  [1,3]                         units:  1
       unique values:  3                         missing .:  51/4168
     unique mv codes:  7                        missing .*:  3835/4168

          tabulation:  Freq.   Numeric  Label
                          14         1  1. Not satisfied
                           2         2  2. Neither
                         266         3  3. Satisfied
                          51         .
                        1494        .a  .a. Did not receive service
                        1630        .g  .g. Gender skip pattern
                           2        .m  .m. Missing
                           5        .s  .s. Skipped by participant
                           5        .w  .w. Answered 6 mon survey at 6
                                        wk visit
                         699        .x  .x. No 6 week survey

-------------------------------------------------------------------------------------------
w6_605_f                                605: CCS:  Waiting time at health facility (min)
-------------------------------------------------------------------------------------------

                type:  numeric (int)
               label:  numserv, but 42 nonmissing values are not labeled
```

```
             range:  [1,2400]                     units:  1
     unique values:  42                       missing .:  51/4168
   unique mv codes:  7                       missing .*:  3837/4168

          examples:  .a    .a. Did not receive service
                     .a    .a. Did not receive service
                     .g    .g. Gender skip pattern
                     .g    .g. Gender skip pattern

-------------------------------------------------------------------------------
w6_606_f                                606: CCS: Travel time to health facility (min)
-------------------------------------------------------------------------------

              type:  numeric (int)
             label:  hfttime, but 29 nonmissing values are not labeled

             range:  [1,888]                      units:  1
     unique values:  30                       missing .:  51/4168
   unique mv codes:  7                       missing .*:  3838/4168

        tabulation:  Freq.   Numeric  Label
                         2         1
                         6         5
                         1         7
                        27        10
                         1        12
                        35        15
                        21        20
                         3        25
                         1        28
                        49        30
                        11        40
                        22        45
                         5        50
                        39        60
                         2        62
                         1        63
                         1        67
                         1        69
                         1        70
                         1        75
                         1        81
                         1        85
                         3        90
                         2       100
                         2       118
                        20       120
                         1       130
                         1       190
                         2       240
                        16       888  888. Don't Know
                        51         .
                      1494        .a  .a. Did not receive service
                      1630        .g  .g. Gender skip pattern
                         3        .m  .m. Missing
                         7        .s  .s. Skipped by participant
                         5        .w  .w. Answered 6 mon survey at 6
                                      wk visit
                       699        .x  .x. No 6 week survey

-------------------------------------------------------------------------------
w6_607_f                                      607: CCS: Travel mode to health faciity
-------------------------------------------------------------------------------

              type:  numeric (byte)
             label:  hftmode

             range:  [1,6]                        units:  1
     unique values:  6                        missing .:  51/4168
```

```
      unique mv codes:  7                      missing .*:  3836/4168

          tabulation:  Freq.   Numeric  Label
                          84         1  1. Bus
                          69         2  2. Taxi
                           3         3  3. Someone drove in private car
                           1         4  4. Drove self
                         115         5  5. Walked
                           9         6  6. Bicycle
                          51         .
                        1494        .a  .a. Did not receive service
                        1630        .g  .g. Gender skip pattern
                           3        .m  .m. Missing
                           5        .s  .s. Skipped by participant
                           5        .w  .w. Answered 6 mon survey at 6
                                        wk visit
                         699        .x  .x. No 6 week survey

-----------------------------------------------------------------------------------------------
w6_608_f                                                            608: CCS: Transport cost
-----------------------------------------------------------------------------------------------

                type:  numeric (byte)
               label:  hftcost, but 15 nonmissing values are not labeled

               range:  [0,80]                       units:  1
       unique values:  16                       missing .:  51/4168
      unique mv codes:  8                      missing .*:  3964/4168

          tabulation:  Freq.   Numeric  Label
                          42         0  0. Nothing
                           3         1
                          15         2
                          15         3
                          19         4
                          18         5
                           3         6
                           3         8
                          14        10
                           5        15
                           8        20
                           2        25
                           1        28
                           2        35
                           2        60
                           1        80
                          51         .
                        1494        .a  .a. Did not receive service
                         125        .b  .b. Own car, walked, biked
                        1630        .g  .g. Gender skip pattern
                           3        .m  .m. Missing
                           8        .s  .s. Skipped by participant
                           5        .w  .w. Answered 6 mon survey at 6
                                        wk visit
                         699        .x  .x. No 6 week survey

-----------------------------------------------------------------------------------------------
w6_609_f                                                     609: CCS: Distance traveled (km)
-----------------------------------------------------------------------------------------------

                type:  numeric (int)
               label:  hftkm, but 14 nonmissing values are not labeled

               range:  [0,888]                      units:  1
       unique values:  16                       missing .:  51/4168
      unique mv codes:  7                      missing .*:  3861/4168

          tabulation:  Freq.   Numeric  Label
                          32         0  0. <1
                          21         1
```

```
                           37         2
                           18         3
                           15         4
                            5         5
                            2         6
                            2         8
                            2         9
                            1        10
                            1        14
                            1        16
                            1        19
                            1        30
                            1        60
                          116       888  888. Don't Know
                           51         .
                         1494        .a  .a. Did not receive service
                         1630        .g  .g. Gender skip pattern
                            3        .m  .m. Missing
                           30        .s  .s. Skipped by participant
                            5        .w  .w. Answered 6 mon survey at 6
                                         wk visit
                          699        .x  .x. No 6 week survey

-------------------------------------------------------------------------------
w6_610_f                                       610: CCS: Source of transport money
-------------------------------------------------------------------------------

                  type:  numeric (byte)
                 label:  hftsource

                 range:  [1,6]                        units:  1
         unique values:  6                        missing .:  51/4168
       unique mv codes:  9                       missing .*:  4007/4168

            tabulation:  Freq.   Numeric  Label
                           12         1  1. Cutting down on other
                                         expenses
                           30         2  2. Savings
                            2         3  3. Borrowing
                            2         4  4. Selling assets
                           43         5  5. Donation
                           21         6  6. Other
                           51         .
                         1494        .a  .a. Did not receive service
                           42        .b  .b. Did not pay for transport
                          125        .c  .c. Own car, walked, biked
                         1630        .g  .g. Gender skip pattern
                            3        .m  .m. Missing
                            9        .s  .s. Skipped by participant
                            5        .w  .w. Answered 6 mon survey at 6
                                         wk visit
                          699        .x  .x. No 6 week survey

-------------------------------------------------------------------------------
w6_611_f                   611: CCS: What respondent would be doing if not at health facility
-------------------------------------------------------------------------------

                  type:  numeric (byte)
                 label:  hfnot

                 range:  [1,6]                        units:  1
         unique values:  6                        missing .:  51/4168
       unique mv codes:  7                       missing .*:  3835/4168

            tabulation:  Freq.   Numeric  Label
                           22         1  1. Paid employment
                           58         2  2. Own business
                          140         3  3. Unpaid work/Housework
                            9         4  4. In school
                           41         5  5. Resting/No specific activity
```

```
                        12         6  6. Other
                        51         .
                      1494        .a  .a. Did not receive service
                      1630        .g  .g. Gender skip pattern
                         3        .m  .m. Missing
                         4        .s  .s. Skipped by participant
                         5        .w  .w. Answered 6 mon survey at 6
                                      wk visit
                       699        .x  .x. No 6 week survey

-------------------------------------------------------------------------------------------
w6_700_m          700: VMMC: Respondent understands to only include services from last 6 weeks
-------------------------------------------------------------------------------------------

                  type:  numeric (byte)
                 label:  yesnoserv

                 range:  [1,1]                        units:  1
         unique values:  1                        missing .:  51/4168
       unique mv codes:  7                       missing .*:  3821/4168

            tabulation:  Freq.   Numeric  Label
                           296         1  1. Yes
                            51         .
                          1325        .a  .a. Did not receive service
                          1783        .g  .g. Gender skip pattern
                             7        .m  .m. Missing
                             2        .s  .s. Skipped by participant
                             5        .w  .w. Answered 6 mon survey at 6
                                          wk visit
                           699        .x  .x. No 6 week survey

-------------------------------------------------------------------------------------------
w6_701_m                                    701: VMMC: numservber times to health facility
-------------------------------------------------------------------------------------------

                  type:  numeric (byte)
                 label:  numserv, but 6 nonmissing values are not labeled

                 range:  [1,6]                        units:  1
         unique values:  6                        missing .:  51/4168
       unique mv codes:  7                       missing .*:  3821/4168

            tabulation:  Freq.   Numeric  Label
                           153         1
                            62         2
                            62         3
                            15         4
                             2         5
                             2         6
                            51         .
                          1325        .a  .a. Did not receive service
                          1783        .g  .g. Gender skip pattern
                             6        .m  .m. Missing
                             3        .s  .s. Skipped by participant
                             5        .w  .w. Answered 6 mon survey at 6
                                          wk visit
                           699        .x  .x. No 6 week survey

-------------------------------------------------------------------------------------------
w6_702_m                                                 702: VMMC: Health facility name
-------------------------------------------------------------------------------------------

                  type:  string (str58)

         unique values:  56                       missing "":  755/4168

              examples:  "."
                         "."
                         "."
```

```
                      "."

             warning:  variable has embedded blanks

-----------------------------------------------------------------------------------------------
w6_703_m                                                     703: VMMC: Alone or accompanied
-----------------------------------------------------------------------------------------------

                type:  numeric (byte)
               label:  hfpeople

               range:  [1,3]                        units:  1
       unique values:  3                        missing .:  51/4168
     unique mv codes:  7                       missing .*:  3834/4168

          tabulation:  Freq.   Numeric  Label
                         196         1  1. Alone
                          23         2  2. Spouse/Partner
                          64         3  3. Someone else
                          51         .
                        1325        .a  .a. Did not receive service
                        1783        .g  .g. Gender skip pattern
                           6        .m  .m. Missing
                          16        .s  .s. Skipped by participant
                           5        .w  .w. Answered 6 mon survey at 6
                                        wk visit
                         699        .x  .x. No 6 week survey

-----------------------------------------------------------------------------------------------
w6_704_m                                                  704: VMMC: Satisfied with services
-----------------------------------------------------------------------------------------------

                type:  numeric (byte)
               label:  hfsat

               range:  [1,3]                        units:  1
       unique values:  3                        missing .:  51/4168
     unique mv codes:  7                       missing .*:  3835/4168

          tabulation:  Freq.   Numeric  Label
                           8         1  1. Not satisfied
                           9         2  2. Neither
                         265         3  3. Satisfied
                          51         .
                        1325        .a  .a. Did not receive service
                        1783        .g  .g. Gender skip pattern
                           6        .m  .m. Missing
                          17        .s  .s. Skipped by participant
                           5        .w  .w. Answered 6 mon survey at 6
                                        wk visit
                         699        .x  .x. No 6 week survey

-----------------------------------------------------------------------------------------------
w6_705_m                                    705: VMMC:  Waiting time at health facility (min)
-----------------------------------------------------------------------------------------------

                type:  numeric (int)
               label:  numserv, but 32 nonmissing values are not labeled

               range:  [1,480]                      units:  1
       unique values:  32                       missing .:  51/4168
     unique mv codes:  7                       missing .*:  3834/4168

            examples:  .a    .a. Did not receive service
                       .g    .g. Gender skip pattern
                       .g    .g. Gender skip pattern
                       .g    .g. Gender skip pattern

-----------------------------------------------------------------------------------------------
w6_706_m                                                   706: VMMC: Weeks since circumcision
```

```
                type:  numeric (byte)
               label:  w6_706

               range:  [0,6]                        units:  1
       unique values:  7                        missing .:  51/4168
     unique mv codes:  7                       missing .*:  3844/4168

          tabulation:  Freq.   Numeric  Label
                           3         0  0. Did not get circumcised
                           3         1  1. 1 week ago
                          10         2  2. 2 weeks ago
                           4         3  3. 3 weeks ago
                          16         4  4. 4 weeks ago
                          27         5  5. 5 weeks ago
                         210         6  6. 6 weeks ago
                          51         .
                        1325        .a  .a. Did not receive service
                        1783        .g  .g. Gender skip pattern
                           6        .m  .m. Missing
                          26        .s  .s. Skipped by participant
                           5        .w  .w. Answered 6 mon survey at 6
                                        wk visit
                         699        .x  .x. No 6 week survey
```

---

w6_707_m   707: VMMC: Resumed sexual activity since circumcision

---

```
                type:  numeric (byte)
               label:  w6_707

               range:  [0,1]                        units:  1
       unique values:  2                        missing .:  51/4168
     unique mv codes:  8                       missing .*:  3826/4168

          tabulation:  Freq.   Numeric  Label
                         200         0  0. No
                          91         1  1. Yes
                          51         .
                        1325        .a  .a. Did not receive service
                           3        .b  .b. Did not get circumcised
                        1783        .g  .g. Gender skip pattern
                           7        .m  .m. Missing
                           4        .s  .s. Skipped by participant
                           5        .w  .w. Answered 6 mon survey at 6
                                        wk visit
                         699        .x  .x. No 6 week survey
```

---

w6_708_m   708: VMMC: Weeks after circumcision resumed sexual activity

---

```
                type:  numeric (byte)
               label:  w6_708

               range:  [0,6]                        units:  1
       unique values:  7                        missing .:  51/4168
     unique mv codes:  9                       missing .*:  4035/4168

          tabulation:  Freq.   Numeric  Label
                           2         0  0. <1 week
                           2         1  1. 1 week
                           9         2  2. 2 weeks
                           4         3  3. 3 weeks
                          18         4  4. 4 weeks
                          15         5  5. 5 weeks
                          32         6  6. 6 weeks
                          51         .
```

```
                    1325        .a  .a. Did not receive service
                       3        .b  .b. Did not get circumcised
                     200        .c  .c. Have not resumed sexual
                                        activity
                    1783        .g  .g. Gender skip pattern
                       7        .m  .m. Missing
                      13        .s  .s. Skipped by participant
                       5        .w  .w. Answered 6 mon survey at 6
                                        wk visit
                     699        .x  .x. No 6 week survey

-------------------------------------------------------------------------------
w6_709_m                                709: VMMC: Travel time to health facility (min)
-------------------------------------------------------------------------------

                  type:  numeric (int)
                 label:  hfttime, but 26 nonmissing values are not labeled

                 range:  [2,888]                      units:  1
         unique values:  27                       missing .:  51/4168
       unique mv codes:  7                       missing .*:  3837/4168

           tabulation:  Freq.   Numeric  Label
                            1         2
                            2         4
                           15         5
                            1         6
                            1         7
                            1         8
                           21        10
                            3        14
                           22        15
                            1        18
                           24        20
                            6        25
                           59        30
                            7        35
                           14        40
                           19        45
                            3        50
                           47        60
                            1        65
                            1        75
                            2        80
                           13        90
                            1        95
                            2       100
                            8       120
                            1       180
                            4       888  888. Don't Know
                           51         .
                         1325        .a  .a. Did not receive service
                         1783        .g  .g. Gender skip pattern
                            7        .m  .m. Missing
                           18        .s  .s. Skipped by participant
                            5        .w  .w. Answered 6 mon survey at 6
                                             wk visit
                          699        .x  .x. No 6 week survey

-------------------------------------------------------------------------------
w6_710_m                                       710: VMMC: Travel mode to health facity
-------------------------------------------------------------------------------

                  type:  numeric (byte)
                 label:  hftmode

                 range:  [1,7]                        units:  1
         unique values:  7                        missing .:  51/4168
       unique mv codes:  7                       missing .*:  3835/4168
```

```
          tabulation:  Freq.   Numeric  Label
                          56         1  1. Bus
                          44         2  2. Taxi
                           7         3  3. Someone drove in private car
                           9         4  4. Drove self
                         146         5  5. Walked
                          18         6  6. Bicycle
                           2         7  7. Other
                          51         .  
                        1325        .a  .a. Did not receive service
                        1783        .g  .g. Gender skip pattern
                           7        .m  .m. Missing
                          16        .s  .s. Skipped by participant
                           5        .w  .w. Answered 6 mon survey at 6
                                        wk visit
                         699        .x  .x. No 6 week survey

--------------------------------------------------------------------------------
w6_711_m                                               711: VMMC: Transport cost
--------------------------------------------------------------------------------

                type:  numeric (byte)
               label:  hftcost, but 16 nonmissing values are not labeled

               range:  [0,50]                       units:  1
       unique values:  17                       missing .:  51/4168
     unique mv codes:  8                       missing .*:  4010/4168

          tabulation:  Freq.   Numeric  Label
                          16         0  0. Nothing
                           3         1  
                           5         2  
                          15         3  
                           7         4  
                          19         5  
                           2         6  
                           2         7  
                           2         8  
                          13        10  
                          10        15  
                           1        17  
                           6        20  
                           1        25  
                           2        30  
                           2        40  
                           1        50  
                          51         .  
                        1325        .a  .a. Did not receive service
                         173        .b  .b. Own car, walked, biked
                        1783        .g  .g. Gender skip pattern
                           7        .m  .m. Missing
                          18        .s  .s. Skipped by participant
                           5        .w  .w. Answered 6 mon survey at 6
                                        wk visit
                         699        .x  .x. No 6 week survey

--------------------------------------------------------------------------------
w6_712_m                                        712: VMMC: Distance traveled (km)
--------------------------------------------------------------------------------

                type:  numeric (int)
               label:  hftkm, but 16 nonmissing values are not labeled

               range:  [0,888]                      units:  1
       unique values:  18                       missing .:  51/4168
     unique mv codes:  7                       missing .*:  3837/4168

          tabulation:  Freq.   Numeric  Label
                          17         0  0. <1
                          18         1  
```

```
                            41         2
                            47         3
                            30         4
                            17         5
                            18         6
                             5         7
                             5         8
                             2         9
                            13        10
                             2        12
                             1        14
                            11        15
                             1        21
                             1        30
                             1        60
                            50       888  888. Don't Know
                            51         .
                          1325        .a  .a. Did not receive service
                          1783        .g  .g. Gender skip pattern
                             7        .m  .m. Missing
                            18        .s  .s. Skipped by participant
                             5        .w  .w. Answered 6 mon survey at 6
                                          wk visit
                           699        .x  .x. No 6 week survey
```

```
-----------------------------------------------------------------------------------------------
w6_713_m                                                  713: VMMC: Source of transport money
-----------------------------------------------------------------------------------------------

                  type:  numeric (byte)
                 label:  hftsource

                 range:  [1,6]                        units:  1
         unique values:  5                        missing .:  51/4168
       unique mv codes:  9                       missing .*:  4025/4168

           tabulation:  Freq.   Numeric  Label
                            17         1  1. Cutting down on other
                                          expenses
                            43         2  2. Savings
                             5         3  3. Borrowing
                            24         5  5. Donation
                             3         6  6. Other
                            51         .
                          1325        .a  .a. Did not receive service
                            16        .b  .b. Did not pay for transport
                           173        .c  .c. Own car, walked, biked
                          1783        .g  .g. Gender skip pattern
                             7        .m  .m. Missing
                            17        .s  .s. Skipped by participant
                             5        .w  .w. Answered 6 mon survey at 6
                                          wk visit
                           699        .x  .x. No 6 week survey
```

```
-----------------------------------------------------------------------------------------------
w6_714_m                    714: VMMC: What respondent would be doing if not at health facility
-----------------------------------------------------------------------------------------------

                  type:  numeric (byte)
                 label:  hfnot

                 range:  [1,6]                        units:  1
         unique values:  6                        missing .:  51/4168
       unique mv codes:  7                       missing .*:  3836/4168

           tabulation:  Freq.   Numeric  Label
                            77         1  1. Paid employment
                            45         2  2. Own business
                            13         3  3. Unpaid work/Housework
                            62         4  4. In school
```

```
                        66         5  5. Resting/No specific activity
                        18         6  6. Other
                        51         .
                      1325        .a  .a. Did not receive service
                      1783        .g  .g. Gender skip pattern
                         7        .m  .m. Missing
                        17        .s  .s. Skipped by participant
                         5        .w  .w. Answered 6 mon survey at 6
                                      wk visit
                       699        .x  .x. No 6 week survey

-----------------------------------------------------------------------------------------------
w6_800                      800: Self-rated health in past YEAR: 1 (very bad) - 10 (very good)
-----------------------------------------------------------------------------------------------

                  type:  numeric (byte)
                 label:  w6_num, but 10 nonmissing values are not labeled

                 range:  [1,10]                       units:  1
         unique values:  10                       missing .:  51/4168
       unique mv codes:  3                       missing .*:  708/4168

            tabulation:  Freq.   Numeric  Label
                            13         1
                            10         2
                            17         3
                           250         4
                           132         5
                           145         6
                           881         7
                           441         8
                           514         9
                          1006        10
                            51         .
                             9        .m  .m. Missing
                           699        .x  .x. No 6 week survey

-----------------------------------------------------------------------------------------------
w6_801                     801: Self-rated health in past MONTH: 1 (very bad) - 10 (very good)
-----------------------------------------------------------------------------------------------

                  type:  numeric (byte)
                 label:  w6_num, but 10 nonmissing values are not labeled

                 range:  [1,10]                       units:  1
         unique values:  10                       missing .:  51/4168
       unique mv codes:  3                       missing .*:  707/4168

            tabulation:  Freq.   Numeric  Label
                            11         1
                            12         2
                            25         3
                           201         4
                           109         5
                           137         6
                           770         7
                           431         8
                           519         9
                          1195        10
                            51         .
                             8        .m  .m. Missing
                           699        .x  .x. No 6 week survey

-----------------------------------------------------------------------------------------------
w6_802a                                                  802: Health problems past month: Fever
-----------------------------------------------------------------------------------------------

                  type:  numeric (byte)
                 label:  w6_yesno
```

```
           range:  [0,1]                        units:  1
   unique values:  2                        missing .:  51/4168
 unique mv codes:  4                       missing .*:  709/4168

     tabulation:  Freq.   Numeric  Label
                   2810         0  0. No
                    598         1  1. Yes
                     51         .
                      9        .m  .m. Missing
                      1        .s  .s. Skipped by participant
                    699        .x  .x. No 6 week survey
```

```
w6_802b                                       802: Health problems past month: Night sweat
```

```
            type:  numeric (byte)
           label:  w6_yesno

           range:  [0,1]                        units:  1
   unique values:  2                        missing .:  51/4168
 unique mv codes:  4                       missing .*:  709/4168

     tabulation:  Freq.   Numeric  Label
                   2980         0  0. No
                    428         1  1. Yes
                     51         .
                      9        .m  .m. Missing
                      1        .s  .s. Skipped by participant
                    699        .x  .x. No 6 week survey
```

```
w6_802c                                 802: Health problems past month: Rapid weight loss
```

```
            type:  numeric (byte)
           label:  w6_yesno

           range:  [0,1]                        units:  1
   unique values:  2                        missing .:  51/4168
 unique mv codes:  4                       missing .*:  709/4168

     tabulation:  Freq.   Numeric  Label
                   3157         0  0. No
                    251         1  1. Yes
                     51         .
                      9        .m  .m. Missing
                      1        .s  .s. Skipped by participant
                    699        .x  .x. No 6 week survey
```

```
w6_802d                                802: Health problems past month: Recurring diarrhea
```

```
            type:  numeric (byte)
           label:  w6_yesno

           range:  [0,1]                        units:  1
   unique values:  2                        missing .:  51/4168
 unique mv codes:  4                       missing .*:  709/4168

     tabulation:  Freq.   Numeric  Label
                   2956         0  0. No
                    452         1  1. Yes
                     51         .
                      9        .m  .m. Missing
                      1        .s  .s. Skipped by participant
                    699        .x  .x. No 6 week survey
```

```
w6_802e             802: Health problems past month: Recurring coughing or shortness of breath
-----------------------------------------------------------------------------------------------

                  type:  numeric (byte)
                 label:  w6_yesno

                 range:  [0,1]                        units:  1
         unique values:  2                        missing .:  51/4168
       unique mv codes:  4                       missing .*:  709/4168

            tabulation:  Freq.   Numeric  Label
                          2918         0  0. No
                           490         1  1. Yes
                            51         .  
                             9        .m  .m. Missing
                             1        .s  .s. Skipped by participant
                           699        .x  .x. No 6 week survey

-----------------------------------------------------------------------------------------------
w6_802f                                     802: Health problems past month: Recurring vomiting
-----------------------------------------------------------------------------------------------

                  type:  numeric (byte)
                 label:  w6_yesno

                 range:  [0,1]                        units:  1
         unique values:  2                        missing .:  51/4168
       unique mv codes:  4                       missing .*:  709/4168

            tabulation:  Freq.   Numeric  Label
                          3147         0  0. No
                           261         1  1. Yes
                            51         .  
                             9        .m  .m. Missing
                             1        .s  .s. Skipped by participant
                           699        .x  .x. No 6 week survey

-----------------------------------------------------------------------------------------------
w6_802g                                      802: Health problems past month: Recurring fatigue
-----------------------------------------------------------------------------------------------

                  type:  numeric (byte)
                 label:  w6_yesno

                 range:  [0,1]                        units:  1
         unique values:  2                        missing .:  51/4168
       unique mv codes:  4                       missing .*:  709/4168

            tabulation:  Freq.   Numeric  Label
                          3027         0  0. No
                           381         1  1. Yes
                            51         .  
                             9        .m  .m. Missing
                             1        .s  .s. Skipped by participant
                           699        .x  .x. No 6 week survey

-----------------------------------------------------------------------------------------------
w6_803                                                803: Covered by health insurance/scheme
-----------------------------------------------------------------------------------------------

                  type:  numeric (byte)
                 label:  w6_yesno

                 range:  [0,88]                       units:  1
         unique values:  3                        missing .:  51/4168
       unique mv codes:  3                       missing .*:  707/4168

            tabulation:  Freq.   Numeric  Label
                          3220         0  0. No
                           180         1  1. Yes
```

```
                         10        88  88. Don't Know
                         51         .
                          8        .m  .m. Missing
                        699        .x  .x. No 6 week survey

-------------------------------------------------------------------------------------------
w6_804                                                  804: Type of health insurance/scheme
-------------------------------------------------------------------------------------------

                  type:  numeric (byte)
                 label:  w6_804

                 range:  [1,96]                       units:  1
         unique values:  7                        missing .:  51/4168
       unique mv codes:  4                       missing .*:  3951/4168

            tabulation:  Freq.   Numeric  Label
                            18         1  1. MUTUAL HEALTH/COMMUNITY-BASED
                            84         2  2. EMPLOYER
                             3         3  3. SOCIAL SECURITY
                            14         4  4. OTHER PRIVATELY PURCHASED
                                          COMMERCIAL
                            26         5  5. LOW COST PRE-PAYMENT
                            11         6  6. HIGH COST PRE-PAYMENT
                            10        96  96. OTHER
                            51         .
                          3220        .a  .a. No insurance
                            32        .m  .m. Missing
                           699        .x  .x. No 6 week survey

-------------------------------------------------------------------------------------------
w6_804a                                      804a: If other, specify type of health insurance
-------------------------------------------------------------------------------------------

                  type:  string (str68)

         unique values:  10                       missing "":  4158/4168

            tabulation:  Freq.  Value
                          4158  ""
                             1  "AIRTEL INSURANCE"
                             1  "AIRTEL INSURANCE COVER"
                             1  "Chershiere Homes pays for  the client"
                             1  "CheshIre home society"
                             1  "Heath insurance through husbands
                                employer"
                             1  "Insured through family members
                                employer"
                             1  "Managed fund"
                             1  "Quick fit chipata"
                             1  "UnKnown by participant as it is an
                                extension of his mother's policy."
                             1  "through my father who works for a
                                hospital"

               warning:  variable has embedded blanks

-------------------------------------------------------------------------------------------
w6_805                                   805: FP: Currently doing something to avoid pregnancy
-------------------------------------------------------------------------------------------

                  type:  numeric (byte)
                 label:  w6_805

                 range:  [0,1]                        units:  1
         unique values:  2                        missing .:  51/4168
       unique mv codes:  6                       missing .*:  1056/4168

            tabulation:  Freq.   Numeric  Label
                          1106         0  0. No
```

```
                        1955         1  1. Yes
                          51         .
                         215        .a  .a. FP Client: see section 500
                         102        .e  .e. Programming skip error: Most
                                        males who answered 804 were
                                        skipped out of 805-807
                          10        .m  .m. Missing
                          30        .s  .s. Skipped by participant
                         699        .x  .x. No 6 week survey
```

w6_805:
  1. 12 cases made NO who originally reported YES to w6_805, but said NO to all methods in w6_807.

```
--------------------------------------------------------------------------------------------
w6_806                                         806: FP: Know place to get family planning
--------------------------------------------------------------------------------------------

                  type:  numeric (byte)
                 label:  w6_806

                 range:  [0,1]                        units:  1
         unique values:  2                        missing .:  51/4168
       unique mv codes:  7                       missing .*:  3005/4168

            tabulation:  Freq.   Numeric  Label
                           400         0  0. No
                           712         1  1. Yes
                            51         .
                           215        .a  .a. FP Client: see section 500
                          1955        .b  .b. Currently doing something to
                                          avoid pregnancy: 805 = 1
                           102        .e  .e. Programming skip error: Most
                                          males who answered 804 were
                                          skipped out of 805-807
                            22        .m  .m. Missing
                            12        .s  .s. Skipped by participant
                           699        .x  .x. No 6 week survey

--------------------------------------------------------------------------------------------
w6_807a                                             807: FP: Method: Female sterilization
--------------------------------------------------------------------------------------------

                  type:  numeric (byte)
                 label:  w6_807a

                 range:  [0,1]                        units:  1
         unique values:  2                        missing .:  51/4168
       unique mv codes:  7                       missing .*:  2165/4168

            tabulation:  Freq.   Numeric  Label
                          1948         0  0. No
                             4         1  1. Yes
                            51         .
                           215        .a  .a. FP Client: see section 500
                          1106        .b  .b. Not using a method: 805 = 0
                           102        .e  .e. Programming skip error: Most
                                          males who answered 804 were
                                          skipped out of 805-807
                            31        .m  .m. Missing
                            12        .s  .s. Skipped by participant
                           699        .x  .x. No 6 week survey

--------------------------------------------------------------------------------------------
w6_807b                                               807: FP: Method: Male sterilization
--------------------------------------------------------------------------------------------

                  type:  numeric (byte)
                 label:  w6_807b
```

```
          range:  [0,1]                        units:  1
  unique values:  2                        missing .:  51/4168
unique mv codes:  7                       missing .*:  2165/4168

    tabulation:  Freq.   Numeric  Label
                  1949         0  0. No
                     3         1  1. Yes
                    51         .
                   215        .a  .a. FP Client: see section 500
                  1106        .b  .b. Not using a method: 805 = 0
                   102        .e  .e. Programming skip error: Most
                                  males who answered 804 were
                                  skipped out of 805-807
                    31        .m  .m. Missing
                    12        .s  .s. Skipped by participant
                   699        .x  .x. No 6 week survey
```

---

w6_807c — 807: FP: Method: Pill

```
           type:  numeric (byte)
          label:  w6_807c

          range:  [0,1]                        units:  1
  unique values:  2                        missing .:  51/4168
unique mv codes:  7                       missing .*:  2165/4168

    tabulation:  Freq.   Numeric  Label
                  1825         0  0. No
                   127         1  1. Yes
                    51         .
                   215        .a  .a. FP Client: see section 500
                  1106        .b  .b. Not using a method: 805 = 0
                   102        .e  .e. Programming skip error: Most
                                  males who answered 804 were
                                  skipped out of 805-807
                    31        .m  .m. Missing
                    12        .s  .s. Skipped by participant
                   699        .x  .x. No 6 week survey
```

---

w6_807d — 807: FP: Method: IUD

```
           type:  numeric (byte)
          label:  w6_807d

          range:  [0,1]                        units:  1
  unique values:  2                        missing .:  51/4168
unique mv codes:  7                       missing .*:  2165/4168

    tabulation:  Freq.   Numeric  Label
                  1811         0  0. No
                   141         1  1. Yes
                    51         .
                   215        .a  .a. FP Client: see section 500
                  1106        .b  .b. Not using a method: 805 = 0
                   102        .e  .e. Programming skip error: Most
                                  males who answered 804 were
                                  skipped out of 805-807
                    31        .m  .m. Missing
                    12        .s  .s. Skipped by participant
                   699        .x  .x. No 6 week survey
```

---

w6_807e — 807: FP: Method: Injectables

```
           type:  numeric (byte)
```

```
              label:  w6_807e

              range:  [0,1]                        units:  1
      unique values:  2                        missing .:  51/4168
    unique mv codes:  7                       missing .*:  2165/4168

        tabulation:  Freq.   Numeric  Label
                      1761         0  0. No
                       191         1  1. Yes
                        51         .
                       215        .a  .a. FP Client: see section 500
                      1106        .b  .b. Not using a method: 805 = 0
                       102        .e  .e. Programming skip error: Most
                                      males who answered 804 were
                                      skipped out of 805-807
                        31        .m  .m. Missing
                        12        .s  .s. Skipped by participant
                       699        .x  .x. No 6 week survey

--------------------------------------------------------------------------------
w6_807f                                              807: FP: Method: Implants
--------------------------------------------------------------------------------

               type:  numeric (byte)
              label:  w6_807f

              range:  [0,1]                        units:  1
      unique values:  2                        missing .:  51/4168
    unique mv codes:  7                       missing .*:  2165/4168

        tabulation:  Freq.   Numeric  Label
                      1184         0  0. No
                       768         1  1. Yes
                        51         .
                       215        .a  .a. FP Client: see section 500
                      1106        .b  .b. Not using a method: 805 = 0
                       102        .e  .e. Programming skip error: Most
                                      males who answered 804 were
                                      skipped out of 805-807
                        31        .m  .m. Missing
                        12        .s  .s. Skipped by participant
                       699        .x  .x. No 6 week survey

--------------------------------------------------------------------------------
w6_807g                                            807: FP: Method: Male condom
--------------------------------------------------------------------------------

               type:  numeric (byte)
              label:  w6_807g

              range:  [0,1]                        units:  1
      unique values:  2                        missing .:  51/4168
    unique mv codes:  7                       missing .*:  2165/4168

        tabulation:  Freq.   Numeric  Label
                      1200         0  0. No
                       752         1  1. Yes
                        51         .
                       215        .a  .a. FP Client: see section 500
                      1106        .b  .b. Not using a method: 805 = 0
                       102        .e  .e. Programming skip error: Most
                                      males who answered 804 were
                                      skipped out of 805-807
                        31        .m  .m. Missing
                        12        .s  .s. Skipped by participant
                       699        .x  .x. No 6 week survey

--------------------------------------------------------------------------------
w6_807h                                          807: FP: Method: Female condom
--------------------------------------------------------------------------------
```

```
              type:  numeric (byte)
             label:  w6_807h

             range:  [0,1]                        units:  1
     unique values:  2                        missing .:  51/4168
   unique mv codes:  7                       missing .*:  2165/4168

        tabulation:  Freq.   Numeric  Label
                      1925         0  0. No
                        27         1  1. Yes
                        51         .
                       215        .a  .a. FP Client: see section 500
                      1106        .b  .b. Not using a method: 805 = 0
                       102        .e  .e. Programming skip error: Most
                                      males who answered 804 were
                                      skipped out of 805-807
                        31        .m  .m. Missing
                        12        .s  .s. Skipped by participant
                       699        .x  .x. No 6 week survey

-----------------------------------------------------------------------------------------------
w6_807i                                                          807: FP: Method: Diaphragm
-----------------------------------------------------------------------------------------------

              type:  numeric (byte)
             label:  w6_807i

             range:  [0,1]                        units:  1
     unique values:  2                        missing .:  51/4168
   unique mv codes:  7                       missing .*:  2165/4168

        tabulation:  Freq.   Numeric  Label
                      1948         0  0. No
                         4         1  1. Yes
                        51         .
                       215        .a  .a. FP Client: see section 500
                      1106        .b  .b. Not using a method: 805 = 0
                       102        .e  .e. Programming skip error: Most
                                      males who answered 804 were
                                      skipped out of 805-807
                        31        .m  .m. Missing
                        12        .s  .s. Skipped by participant
                       699        .x  .x. No 6 week survey

-----------------------------------------------------------------------------------------------
w6_807j                                                         807: FP: Method: Foam/Jelly
-----------------------------------------------------------------------------------------------

              type:  numeric (byte)
             label:  w6_807j

             range:  [0,1]                        units:  1
     unique values:  2                        missing .:  51/4168
   unique mv codes:  7                       missing .*:  2165/4168

        tabulation:  Freq.   Numeric  Label
                      1950         0  0. No
                         2         1  1. Yes
                        51         .
                       215        .a  .a. FP Client: see section 500
                      1106        .b  .b. Not using a method: 805 = 0
                       102        .e  .e. Programming skip error: Most
                                      males who answered 804 were
                                      skipped out of 805-807
                        31        .m  .m. Missing
                        12        .s  .s. Skipped by participant
                       699        .x  .x. No 6 week survey

-----------------------------------------------------------------------------------------------
```

```
w6_807k                                  807: FP: Method: Lactational amenorrhea method
---------------------------------------------------------------------------------------

                  type:  numeric (byte)
                 label:  w6_807k

                 range:  [0,1]                        units:  1
         unique values:  2                        missing .:  51/4168
       unique mv codes:  7                       missing .*:  2165/4168

            tabulation:  Freq.   Numeric  Label
                          1948         0  0. No
                             4         1  1. Yes
                            51         .  
                           215        .a  .a. FP Client: see section 500
                          1106        .b  .b. Not using a method: 805 = 0
                           102        .e  .e. Programming skip error: Most
                                          males who answered 804 were
                                          skipped out of 805-807
                            31        .m  .m. Missing
                            12        .s  .s. Skipped by participant
                           699        .x  .x. No 6 week survey

---------------------------------------------------------------------------------------
w6_807l                                               807: FP: Method: Rhythm method
---------------------------------------------------------------------------------------

                  type:  numeric (byte)
                 label:  w6_807l

                 range:  [0,1]                        units:  1
         unique values:  2                        missing .:  51/4168
       unique mv codes:  7                       missing .*:  2165/4168

            tabulation:  Freq.   Numeric  Label
                          1912         0  0. No
                            40         1  1. Yes
                            51         .  
                           215        .a  .a. FP Client: see section 500
                          1106        .b  .b. Not using a method: 805 = 0
                           102        .e  .e. Programming skip error: Most
                                          males who answered 804 were
                                          skipped out of 805-807
                            31        .m  .m. Missing
                            12        .s  .s. Skipped by participant
                           699        .x  .x. No 6 week survey

---------------------------------------------------------------------------------------
w6_807m                                                  807: FP: Method: Withdrawal
---------------------------------------------------------------------------------------

                  type:  numeric (byte)
                 label:  w6_807m

                 range:  [0,1]                        units:  1
         unique values:  2                        missing .:  51/4168
       unique mv codes:  7                       missing .*:  2165/4168

            tabulation:  Freq.   Numeric  Label
                          1912         0  0. No
                            40         1  1. Yes
                            51         .  
                           215        .a  .a. FP Client: see section 500
                          1106        .b  .b. Not using a method: 805 = 0
                           102        .e  .e. Programming skip error: Most
                                          males who answered 804 were
                                          skipped out of 805-807
                            31        .m  .m. Missing
                            12        .s  .s. Skipped by participant
                           699        .x  .x. No 6 week survey
```

```
--------------------------------------------------------------------------------------------
w6_807n                                                   807: FP: Method: Other method
--------------------------------------------------------------------------------------------

                  type:  numeric (byte)
                 label:  w6_807n

                 range:  [0,1]                        units:  1
         unique values:  2                        missing .:  51/4168
       unique mv codes:  7                       missing .*:  2165/4168

            tabulation:  Freq.   Numeric  Label
                          1920         0  0. No
                            32         1  1. Yes
                            51         .
                           215        .a  .a. FP Client: see section 500
                          1106        .b  .b. Not using a method: 805 = 0
                           102        .e  .e. Programming skip error: Most
                                          males who answered 804 were
                                          skipped out of 805-807
                            31        .m  .m. Missing
                            12        .s  .s. Skipped by participant
                           699        .x  .x. No 6 week survey

--------------------------------------------------------------------------------------------
w6_902                                            902: Last time had sexual intercourse
--------------------------------------------------------------------------------------------

                  type:  numeric (byte)
                 label:  w6_902

                 range:  [0,4]                        units:  1
         unique values:  5                        missing .:  51/4168
       unique mv codes:  4                       missing .*:  731/4168

            tabulation:  Freq.   Numeric  Label
                           467         0  0. Never had sex
                          1230         1  1. Today/Days ago
                           733         2  2. More than a week ago
                           737         3  3. More than a month ago
                           219         4  4. More than a year ago
                            51         .
                            10        .m  .m. Missing
                            22        .s  .s. Skipped by participant
                           699        .x  .x. No 6 week survey

--------------------------------------------------------------------------------------------
w6_903                                                903: Times had sex in last week
--------------------------------------------------------------------------------------------

                  type:  numeric (byte)
                 label:  w6_903, but 12 nonmissing values are not labeled

                 range:  [0,88]                       units:  1
         unique values:  12                       missing .:  51/4168
       unique mv codes:  6                       missing .*:  2881/4168

            tabulation:  Freq.   Numeric  Label
                            12         0
                           332         1
                           335         2
                           241         3
                            95         4
                            28         5
                            29         6
                            22         7
                            17         8
                             6         9
                             2        10
```

```
                        117        88
                         51         .
                        467        .a  .a. Never had sex
                       1689        .b  .b. Sex >1 week ago
                         10        .m  .m. Missing
                         16        .s  .s. Skipped by participant
                        699        .x  .x. No 6 week survey

-------------------------------------------------------------------------------------------
w6_904                                    904: Times used condom when had sex in last week
-------------------------------------------------------------------------------------------

                  type:  numeric (byte)
                 label:  w6_904, but 11 nonmissing values are not labeled

                 range:  [0,88]                       units:  1
         unique values:  11                       missing .:  51/4168
       unique mv codes:  6                       missing .*:  2877/4168

            tabulation:  Freq.   Numeric  Label
                           471         0
                           234         1
                           209         2
                            94         3
                            36         4
                            18         5
                            18         6
                             3         7
                             8         8
                             1         9
                           148        88
                            51         .
                           467        .a  .a. Never had sex
                          1689        .b  .b. Sex >1 week ago
                            10        .m  .m. Missing
                            12        .s  .s. Skipped by participant
                           699        .x  .x. No 6 week survey

-------------------------------------------------------------------------------------------
w6_905                     905: Relationship to LAST person with whom had sexual intercourse
-------------------------------------------------------------------------------------------

                  type:  numeric (byte)
                 label:  w6_905

                 range:  [1,6]                        units:  1
         unique values:  6                        missing .:  51/4168
       unique mv codes:  6                       missing .*:  1398/4168

            tabulation:  Freq.   Numeric  Label
                          1361         1  1. Husband/wife
                           360         2  2. Live-in partner
                           873         3  3. Girlfriend/boyfriend not
                                          living with you
                            53         4  4. Person you paid or who paid
                                          you for sex
                            49         5  5. Casual acquaintance
                            23         6  6. Other
                            51         .
                           467        .a  .a. Never had sex
                           219        .b  .b. Sex >1 year ago
                            10        .m  .m. Missing
                             3        .s  .s. Skipped by participant
                           699        .x  .x. No 6 week survey

-------------------------------------------------------------------------------------------
w6_906                                                  906: Condom used last time had sex
-------------------------------------------------------------------------------------------

                  type:  numeric (byte)
```

```
               label:  w6_906

               range:  [0,88]                       units:  1
       unique values:  3                        missing .:  51/4168
     unique mv codes:  6                       missing .*:  1398/4168

          tabulation:  Freq.   Numeric  Label
                        1342         0  0. No
                        1302         1  1. Yes
                          75        88  88. Don't Know
                          51         .  
                         467        .a  .a. Never had sex
                         219        .b  .b. Sex >1 year ago
                          10        .m  .m. Missing
                           3        .s  .s. Skipped by participant
                         699        .x  .x. No 6 week survey

-----------------------------------------------------------------------------------------------
w6_907                  907: Use condom every time had sex with LAST person in last 12 months
-----------------------------------------------------------------------------------------------

                type:  numeric (byte)
               label:  w6_907

               range:  [0,88]                       units:  1
       unique values:  3                        missing .:  51/4168
     unique mv codes:  6                       missing .*:  1401/4168

          tabulation:  Freq.   Numeric  Label
                        1412         0  0. No
                        1193         1  1. Yes
                         111        88  88. Don't Know
                          51         .  
                         467        .a  .a. Never had sex
                         219        .b  .b. Sex >1 year ago
                          10        .m  .m. Missing
                           6        .s  .s. Skipped by participant
                         699        .x  .x. No 6 week survey

-----------------------------------------------------------------------------------------------
w6_908               908: Having sex with other pople during time having sex with LAST person
-----------------------------------------------------------------------------------------------

                type:  numeric (byte)
               label:  w6_908

               range:  [0,88]                       units:  1
       unique values:  3                        missing .:  51/4168
     unique mv codes:  6                       missing .*:  1398/4168

          tabulation:  Freq.   Numeric  Label
                        1672         0  0. No
                         957         1  1. Yes
                          90        88  88. Don't Know
                          51         .  
                         467        .a  .a. Never had sex
                         219        .b  .b. Sex >1 year ago
                          10        .m  .m. Missing
                           3        .s  .s. Skipped by participant
                         699        .x  .x. No 6 week survey

-----------------------------------------------------------------------------------------------
w6_911                                          911: Number new sexual partners in last 6 weeks
-----------------------------------------------------------------------------------------------

                type:  numeric (byte)
               label:  w6_911, but 11 nonmissing values are not labeled

               range:  [0,10]                       units:  1
       unique values:  11                       missing .:  51/4168
```

```
      unique mv codes:  7                      missing .*:  1422/4168

          tabulation:  Freq.   Numeric  Label
                        1386         0
                         920         1
                         232         2
                          92         3
                          26         4
                           4         5
                          13         6
                           7         7
                           8         8
                           5         9
                           2        10
                          51         .
                         467        .a  .a. Never had sex
                         219        .b  .b. Sex >1 year ago
                          10        .m  .m. Missing
                          22        .s  .s. Skipped by participant
                           5        .w  .w. Answered 6 mon survey at 6
                                        wk visit
                         699        .x  .x. No 6 week survey

-----------------------------------------------------------------------------------------------
w6_currpart_an                          Analysis: Currently has spouse/primary sex partner
-----------------------------------------------------------------------------------------------

                type:  numeric (byte)
               label:  w6_yesno

               range:  [0,1]                        units:  1
       unique values:  2                        missing .:  51/4168
     unique mv codes:  3                       missing .*:  703/4168

          tabulation:  Freq.   Numeric  Label
                         563         0  0. No
                        2851         1  1. Yes
                          51         .
                         702        .m  .m. Missing
                           1        .s  .s. Skipped by participant

-----------------------------------------------------------------------------------------------
m6_time                                                                             Time Point
-----------------------------------------------------------------------------------------------

                type:  numeric (byte)
               label:  time

               range:  [1,2]                        units:  1
       unique values:  2                        missing .:  67/4168
     unique mv codes:  2                       missing .*:  755/4168

          tabulation:  Freq.   Numeric  Label
                           2         1  1. 6 Weeks
                        3344         2  2. 6 Months
                          67         .
                         755        .x

-----------------------------------------------------------------------------------------------
m6_ID                                                                6 Month Survey: Survey ID
-----------------------------------------------------------------------------------------------

                type:  numeric (int)
               label:  admin_m6, but 3132 nonmissing values are not labeled

               range:  [803,14899]                  units:  1
       unique values:  3132                     missing .:  67/4168
     unique mv codes:  2                       missing .*:  755/4168

            examples:  7315
```

```
                        10205
                        11888
                        14109

-------------------------------------------------------------------------------------------
m6_Date                              6 Month Survey: Interviewer entered date, System timestamp
-------------------------------------------------------------------------------------------

                  type:  numeric (double)
                 label:  admin_m6, but 3346 nonmissing values are not labeled

                 range:  [1.699e+12,1.749e+12]        units:  1000
         unique values:  3346                     missing .:  67/4168
       unique mv codes:  2                       missing .*:  755/4168

              examples:  1.729e+12
                         1.734e+12
                         1.739e+12
                         1.748e+12

-------------------------------------------------------------------------------------------
m6_SurveyStatus                                           6 Month Survey: Survey Status
-------------------------------------------------------------------------------------------

                  type:  numeric (byte)
                 label:  SurveyStatus_m6

                 range:  [0,5]                        units:  1
         unique values:  6                        missing .:  0/4168
       unique mv codes:  1                       missing .*:  787/4168

            tabulation:  Freq.   Numeric  Label
                             9         0  0. Interrupted
                          3331         1  1. Completed
                             4         2  2. Completed on resumption
                             2         3  3. Completed 6 wk survey at 6
                                          mon visit
                            28         4  4. Withdrew from study
                             7         5  5. Deceased
                           787        .x  .x. No 6 month survey

-------------------------------------------------------------------------------------------
m6_AdminAge                                      6 Month Survey: Age entered by interviewer
-------------------------------------------------------------------------------------------

                  type:  numeric (byte)
                 label:  admin_m6, but 31 nonmissing values are not labeled

                 range:  [18,48]                      units:  1
         unique values:  31                       missing .:  67/4168
       unique mv codes:  2                       missing .*:  755/4168

              examples:  22
                         25
                         31
                         48

m6_AdminAge:
  1.  Do not use this variable for analysis. Entered for administrative purposes. Should use
      bl_102a.

-------------------------------------------------------------------------------------------
m6_SurveyGender                                     6 Month Survey: Administrative variable
-------------------------------------------------------------------------------------------

                  type:  string (str6)

         unique values:  2                        missing "":  821/4168

            tabulation:  Freq.  Value
```

```
                           821  ""
                          1734  "Female"
                          1613  "Male"

m6_SurveyGender:
  1.  Survey gender does NOT always match true participant gender.

-------------------------------------------------------------------------------------------------
m6_InterviewerID                                              6 Month Survey: Interviewer ID
-------------------------------------------------------------------------------------------------

                  type:  numeric (int)
                 label:  admin_m6, but 25 nonmissing values are not labeled

                 range:  [100,241]                    units:  1
         unique values:  25                       missing .:  67/4168
       unique mv codes:  2                       missing .*:  755/4168

            tabulation:  Freq.   Numeric  Label
                            64       100
                            57       101
                           168       102
                           173       103
                           104       104
                            61       105
                           232       106
                             3       107
                            74       108
                           102       109
                             1       111
                            96       112
                            36       113
                            97       114
                           208       115
                           186       116
                           211       117
                           114       118
                           164       119
                           271       120
                           226       210
                           134       211
                           333       222
                           230       223
                             1       241
                            67         .
                           755        .x  .x. No 6 month survey

-------------------------------------------------------------------------------------------------
m6_GUID_sys                                         6 Month Survey: Unique survey identifier
-------------------------------------------------------------------------------------------------

                  type:  string (str36)

         unique values:  3347                     missing "":  821/4168

              examples:  "008e04e8-3455-4630-bded-3b8b7b333df4"
                         "4076eee3-ffef-461d-bcd8-e9c78fd4c48e"
                         "826d1f46-fc08-4b11-9ae4-993a3cd9fd6a"
                         "c09e823c-a4ff-4189-a54d-32192f8ef6e3"

-------------------------------------------------------------------------------------------------
m6_SiteID                                                     6 Month Survey: Survey Site ID
-------------------------------------------------------------------------------------------------

                  type:  numeric (byte)
                 label:  m6_SiteID

                 range:  [1,95]                       units:  1
         unique values:  11                       missing .:  67/4168
       unique mv codes:  2                       missing .*:  755/4168
```

```
            tabulation:  Freq.   Numeric  Label
                           444         1  1. SFH HCT - Cairo Road
                           365         2  2. Chawama Clinic - MCH
                           200         3  3. Chawama Clinic - Out Patient
                                          Ward (VMMC)
                           246         5  5. Kamwala Clinic - TB, STI &
                                          HIV Clinic
                           472         6  6. Kapata Urban Clinic, MCH
                           647         7  7. Kapata Urban Clinic, TB, STI
                                          & HIV
                           164         8  8. Chipata Gen Hosp - OP VMMC
                           608        11  11. SFH New Start
                           109        13  13. SFH VMMC
                            40        94  94. YWCA HTC Site
                            51        95  95. YWCA VMMC
                            67         .
                           755        .x  .x. No 6 month survey

m6_SiteID:
  1.  93, 94, & 95 are external evaluation sites

-----------------------------------------------------------------------------------------------
m6_SiteName                                                6 Month Survey: Survey Site String
-----------------------------------------------------------------------------------------------

                  type:  string (str40)

         unique values:  13                       missing "":  821/4168

            tabulation:  Freq.  Value
                           821  ""
                           365  "Chawama Clinic - Maternity Ward"
                           200  "Chawama Clinic - Out Patient Ward
                                (VMMC)"
                           164  "Chipata Gen Hosp - OP VMMC"
                           232  "Kamwala Clinic - TB, STI & HIV Clinic"
                            14  "Kamwala Clinic - TB, STI and HIV
                                Clinic"
                           472  "Kapata Urban Clinic, MCH"
                           615  "Kapata Urban Clinic, TB, STI & HIV"
                            33  "Kapata Urban Clinic, TB, STI and HIV"
                           444  "SFH HCT - Cairo Road"
                           608  "SFH New Start"
                           109  "SFH VMMC"
                            40  "YWCA HTC Site"
                            51  "YWCA VMMC"

               warning:  variable has embedded blanks

m6_SiteName:
  1.  YWCA sites are external evaluation sites

-----------------------------------------------------------------------------------------------
m6_SurveyType                                                       6 Month Survey: Survey Type
-----------------------------------------------------------------------------------------------

                  type:  numeric (byte)
                 label:  admin_m6, but 2 nonmissing values are not labeled

                 range:  [3,4]                        units:  1
         unique values:  2                        missing .:  67/4168
       unique mv codes:  2                       missing .*:  755/4168

            tabulation:  Freq.   Numeric  Label
                             2         3
                          3344         4
                            67         .
                           755        .x  .x. No 6 month survey
```

```
--------------------------------------------------------------------------------------------
m6_SurveyTypeName                                          6 Month Survey: Survey Type String
--------------------------------------------------------------------------------------------

                  type:  string (str21)

         unique values:  2                        missing "":  821/4168

           tabulation:  Freq.  Value
                          821  ""
                         3345  "Reach 6 MONTHS - Both"
                            2  "Reach 6 WEEKS - Both"

              warning:  variable has embedded blanks

--------------------------------------------------------------------------------------------
m6_SurveyDuration_sys                                           Minutes for survey completion
--------------------------------------------------------------------------------------------

                  type:  numeric (long)
                 label:  admin_m6, but 73 nonmissing values are not labeled

                 range:  [-717,142960]                units:  1
         unique values:  73                        missing .:  67/4168
       unique mv codes:  2                        missing .*:  755/4168

              examples:  6
                         9
                         13
                         53

m6_SurveyDuration_sys:
  1.  Large values may be due to completing survey on resumption (see SurveyStatus).

--------------------------------------------------------------------------------------------
m6_Weekday_sys                                                    6 Month Survey: Day of week
--------------------------------------------------------------------------------------------

                  type:  string (str3)

         unique values:  7                        missing "":  821/4168

           tabulation:  Freq.  Value
                          821  ""
                          516  "Fri"
                          821  "Mon"
                          154  "Sat"
                           37  "Sun"
                          513  "Thu"
                          720  "Tue"
                          586  "Wed"

m6_Weekday_sys:
  1.  Generated automatically based on m6_Date

--------------------------------------------------------------------------------------------
m6_Day_sys                                                       6 Month Survey: Day of month
--------------------------------------------------------------------------------------------

                  type:  numeric (byte)
                 label:  admin_m6, but 31 nonmissing values are not labeled

                 range:  [1,31]                       units:  1
         unique values:  31                        missing .:  67/4168
       unique mv codes:  2                        missing .*:  755/4168

              examples:  7
                         15
                         23
                         31
```

```
m6_Day_sys:
  1.  Generated automatically based on m6_Date

-------------------------------------------------------------------------------------------------
m6_Month_sys                                                       6 Month Survey: Day of month
-------------------------------------------------------------------------------------------------

                  type:  numeric (byte)
                 label:  admin_m6, but 12 nonmissing values are not labeled

                 range:  [1,12]                       units:  1
         unique values:  12                       missing .:  67/4168
       unique mv codes:  2                       missing .*:  755/4168

            tabulation:  Freq.   Numeric  Label
                           291         1
                           439         2
                           265         3
                           161         4
                           141         5
                            19         6
                            64         7
                           183         8
                           403         9
                           386        10
                           465        11
                           529        12
                            67         .
                           755        .x  .x. No 6 month survey

m6_Month_sys:
  1.  Generated automatically based on m6_Date

-------------------------------------------------------------------------------------------------
m6_DBVersion                                                                          DBVersion
-------------------------------------------------------------------------------------------------

                  type:  numeric (double)
                 label:  admin_m6, but 2 nonmissing values are not labeled

                 range:  [2.5,2.6]                    units:  .1
         unique values:  2                        missing .:  67/4168
       unique mv codes:  2                       missing .*:  755/4168

            tabulation:  Freq.   Numeric  Label
                           394       2.5
                          2952       2.6
                            67         .
                           755        .x  .x. No 6 month survey

-------------------------------------------------------------------------------------------------
m6_AppVersion                                                                        AppVersion
-------------------------------------------------------------------------------------------------

                  type:  numeric (double)
                 label:  admin_m6, but 2 nonmissing values are not labeled

                 range:  [2.5,2.6]                    units:  .1
         unique values:  2                        missing .:  67/4168
       unique mv codes:  2                       missing .*:  755/4168

            tabulation:  Freq.   Numeric  Label
                           394       2.5
                          2952       2.6
                            67         .
                           755        .x  .x. No 6 month survey

-------------------------------------------------------------------------------------------------
m6_100                                                                              100: Gender
```

```
-------------------------------------------------------------------------------------------

                 type:  numeric (byte)
                label:  m6_100

                range:  [0,1]                        units:  1
        unique values:  2                        missing .:  67/4168
      unique mv codes:  2                       missing .*:  755/4168

           tabulation:  Freq.   Numeric  Label
                         1613         0  0. Male
                         1733         1  1. Female
                           67         .
                          755        .x  .x. No 6 month survey

-------------------------------------------------------------------------------------------
m6_101                                                    101: Married or living with someone
-------------------------------------------------------------------------------------------

                 type:  numeric (byte)
                label:  m6_101

                range:  [0,2]                        units:  1
        unique values:  3                        missing .:  67/4168
      unique mv codes:  3                       missing .*:  758/4168

           tabulation:  Freq.   Numeric  Label
                         1630         0  0. No
                         1671         1  1. Yes, married
                           42         2  2. Yes, living with partner
                           67         .
                            3        .m  .m. Missing
                          755        .x  .x. No 6 month survey

-------------------------------------------------------------------------------------------
m6_102                                                               102: Primary sex partner
-------------------------------------------------------------------------------------------

                 type:  numeric (byte)
                label:  m6_102

                range:  [0,1]                        units:  1
        unique values:  2                        missing .:  67/4168
      unique mv codes:  4                       missing .*:  2472/4168

           tabulation:  Freq.   Numeric  Label
                          519         0  0. No
                         1110         1  1. Yes
                           67         .
                         1713        .a  .a. Married/Living with someone
                            4        .m  .m. Missing
                          755        .x  .x. No 6 month survey

-------------------------------------------------------------------------------------------
m6_103                                                             103: Children 15 and older
-------------------------------------------------------------------------------------------

                 type:  numeric (byte)
                label:  m6_yesno

                range:  [0,1]                        units:  1
        unique values:  2                        missing .:  67/4168
      unique mv codes:  3                       missing .*:  759/4168

           tabulation:  Freq.   Numeric  Label
                         2909         0  0. No
                          433         1  1. Yes
                           67         .
                            4        .m  .m. Missing
                          755        .x  .x. No 6 month survey
```

```
--------------------------------------------------------------------------------------------
m6_104                 104: Respondent understands to only include services from last 6 months
--------------------------------------------------------------------------------------------

                  type:  numeric (byte)
                 label:  m6_yesno

                 range:  [1,1]                        units:  1
         unique values:  1                        missing .:  67/4168
       unique mv codes:  4                       missing .*:  761/4168

            tabulation:  Freq.   Numeric  Label
                          3340         1  1. Yes
                            67         .
                             4        .m  .m. Missing
                             2        .w  .w. Answered 6 wk survey at 6
                                          mon visit
                           755        .x  .x. No 6 month survey

--------------------------------------------------------------------------------------------
m6_105a                               105: Respondent: HIV testing/counseling in last 6 months
--------------------------------------------------------------------------------------------

                  type:  numeric (byte)
                 label:  m6_yesno

                 range:  [0,1]                        units:  1
         unique values:  2                        missing .:  67/4168
       unique mv codes:  5                       missing .*:  763/4168

            tabulation:  Freq.   Numeric  Label
                          2299         0  0. No
                          1039         1  1. Yes
                            67         .
                             4        .m  .m. Missing
                             2        .s  .s. Skipped by participant
                             2        .w  .w. Answered 6 wk survey at 6
                                          mon visit
                           755        .x  .x. No 6 month survey

--------------------------------------------------------------------------------------------
m6_105b                               105: Respondent: HIV care and treatment in last 6 months
--------------------------------------------------------------------------------------------

                  type:  numeric (byte)
                 label:  m6_yesno

                 range:  [0,1]                        units:  1
         unique values:  2                        missing .:  67/4168
       unique mv codes:  4                       missing .*:  761/4168

            tabulation:  Freq.   Numeric  Label
                          3039         0  0. No
                           301         1  1. Yes
                            67         .
                             4        .m  .m. Missing
                             2        .w  .w. Answered 6 wk survey at 6
                                          mon visit
                           755        .x  .x. No 6 month survey

--------------------------------------------------------------------------------------------
m6_105c                               105: Respondent: STD care and treatment in last 6 months
--------------------------------------------------------------------------------------------

                  type:  numeric (byte)
                 label:  m6_yesno

                 range:  [0,1]                        units:  1
         unique values:  2                        missing .:  67/4168
```

```
      unique mv codes:  4                       missing .*:  761/4168

           tabulation:  Freq.   Numeric  Label
                         3197         0  0. No
                          143         1  1. Yes
                           67         .
                            4        .m  .m. Missing
                            2        .w  .w. Answered 6 wk survey at 6
                                         mon visit
                          755        .x  .x. No 6 month survey

-----------------------------------------------------------------------------------------------
m6_105d_f                                 105: Respondent: Family planning in last 6 months
-----------------------------------------------------------------------------------------------

                 type:  numeric (byte)
                label:  m6_yesno

                range:  [0,1]                        units:  1
        unique values:  2                        missing .:  67/4168
      unique mv codes:  5                       missing .*:  2376/4168

           tabulation:  Freq.   Numeric  Label
                         1514         0  0. No
                          211         1  1. Yes
                           67         .
                         1615        .g  .g. Gender skip pattern
                            4        .m  .m. Missing
                            2        .w  .w. Answered 6 wk survey at 6
                                         mon visit
                          755        .x  .x. No 6 month survey

-----------------------------------------------------------------------------------------------
m6_105e_f                                        105: Respondent: Cervical cancer screening
-----------------------------------------------------------------------------------------------

                 type:  numeric (byte)
                label:  m6_yesno

                range:  [0,1]                        units:  1
        unique values:  2                        missing .:  67/4168
      unique mv codes:  5                       missing .*:  2376/4168

           tabulation:  Freq.   Numeric  Label
                         1416         0  0. No
                          309         1  1. Yes
                           67         .
                         1615        .g  .g. Gender skip pattern
                            4        .m  .m. Missing
                            2        .w  .w. Answered 6 wk survey at 6
                                         mon visit
                          755        .x  .x. No 6 month survey

-----------------------------------------------------------------------------------------------
m6_105f_m                               105: Respondent: Male circumcision in last 6 months
-----------------------------------------------------------------------------------------------

                 type:  numeric (byte)
                label:  m6_yesno

                range:  [0,1]                        units:  1
        unique values:  2                        missing .:  67/4168
      unique mv codes:  6                       missing .*:  2496/4168

           tabulation:  Freq.   Numeric  Label
                         1314         0  0. No
                          291         1  1. Yes
                           67         .
                         1729        .g  .g. Gender skip pattern
                            9        .m  .m. Missing
```

```
                        1        .s  .s. Skipped by participant
                        2        .w  .w. Answered 6 wk survey at 6
                                         mon visit
                      755        .x  .x. No 6 month survey

-------------------------------------------------------------------------------------------
m6_106a                            106: Partner: HIV testing/counseling in last 6 months
-------------------------------------------------------------------------------------------

                 type:  numeric (byte)
                label:  m6_106

                range:  [0,88]                      units:  1
        unique values:  3                       missing .:  67/4168
      unique mv codes:  5                      missing .*:  1281/4168

          tabulation:  Freq.   Numeric  Label
                        1657         0  0. No
                         938         1  1. Yes
                         225        88  88. Don't Know
                          67         .
                         519        .a  .a. Married/Living with someone
                           5        .m  .m. Missing
                           2        .w  .w. Answered 6 wk survey at 6
                                         mon visit
                         755        .x  .x. No 6 month survey

-------------------------------------------------------------------------------------------
m6_106b                            106: Partner: HIV care and treatment in last 6 months
-------------------------------------------------------------------------------------------

                 type:  numeric (byte)
                label:  m6_106

                range:  [0,88]                      units:  1
        unique values:  3                       missing .:  67/4168
      unique mv codes:  5                      missing .*:  1281/4168

          tabulation:  Freq.   Numeric  Label
                        2445         0  0. No
                         207         1  1. Yes
                         168        88  88. Don't Know
                          67         .
                         519        .a  .a. Married/Living with someone
                           5        .m  .m. Missing
                           2        .w  .w. Answered 6 wk survey at 6
                                         mon visit
                         755        .x  .x. No 6 month survey

-------------------------------------------------------------------------------------------
m6_106c                            106: Partner: STD care and treatment in last 6 months
-------------------------------------------------------------------------------------------

                 type:  numeric (byte)
                label:  m6_106

                range:  [0,88]                      units:  1
        unique values:  3                       missing .:  67/4168
      unique mv codes:  5                      missing .*:  1281/4168

          tabulation:  Freq.   Numeric  Label
                        2575         0  0. No
                         103         1  1. Yes
                         142        88  88. Don't Know
                          67         .
                         519        .a  .a. Married/Living with someone
                           5        .m  .m. Missing
                           2        .w  .w. Answered 6 wk survey at 6
                                         mon visit
                         755        .x  .x. No 6 month survey
```

```
-------------------------------------------------------------------------------------------
m6_106d_m                                    106: Partner: Family planning in last 6 months
-------------------------------------------------------------------------------------------

                  type:  numeric (byte)
                 label:  m6_106

                 range:  [0,88]                       units:  1
         unique values:  3                        missing .:  67/4168
       unique mv codes:  6                       missing .*:  2841/4168

            tabulation:  Freq.   Numeric  Label
                           897         0  0. No
                           283         1  1. Yes
                            80        88  88. Don't Know
                            67         .
                           347        .a  .a. Married/Living with someone
                          1729        .g  .g. Gender skip pattern
                             8        .m  .m. Missing
                             2        .w  .w. Answered 6 wk survey at 6
                                          mon visit
                           755        .x  .x. No 6 month survey

-------------------------------------------------------------------------------------------
m6_106e_m                                           106: Partner: Cervical cancer screening
-------------------------------------------------------------------------------------------

                  type:  numeric (byte)
                 label:  m6_106

                 range:  [0,88]                       units:  1
         unique values:  3                        missing .:  67/4168
       unique mv codes:  6                       missing .*:  2841/4168

            tabulation:  Freq.   Numeric  Label
                           999         0  0. No
                           164         1  1. Yes
                            97        88  88. Don't Know
                            67         .
                           347        .a  .a. Married/Living with someone
                          1729        .g  .g. Gender skip pattern
                             8        .m  .m. Missing
                             2        .w  .w. Answered 6 wk survey at 6
                                          mon visit
                           755        .x  .x. No 6 month survey

-------------------------------------------------------------------------------------------
m6_106f_f                                  106: Partner: Male circumcision in last 6 months
-------------------------------------------------------------------------------------------

                  type:  numeric (byte)
                 label:  m6_106

                 range:  [0,88]                       units:  1
         unique values:  3                        missing .:  67/4168
       unique mv codes:  6                       missing .*:  2548/4168

            tabulation:  Freq.   Numeric  Label
                          1447         0  0. No
                            77         1  1. Yes
                            29        88  88. Don't Know
                            67         .
                           172        .a  .a. Married/Living with someone
                          1615        .g  .g. Gender skip pattern
                             4        .m  .m. Missing
                             2        .w  .w. Answered 6 wk survey at 6
                                          mon visit
                           755        .x  .x. No 6 month survey
```

```
--------------------------------------------------------------------------------------------
m6_107a                               107: Children: HIV testing/counseling in last 6 months
--------------------------------------------------------------------------------------------

                  type:  numeric (byte)
                 label:  m6_107

                 range:  [0,88]                       units:  1
         unique values:  3                        missing .:  67/4168
       unique mv codes:  5                       missing .*:  3668/4168

            tabulation:  Freq.   Numeric  Label
                           300         0  0. No
                            92         1  1. Yes
                            41        88  88. Don't Know
                            67         .  
                          2907        .a  .a. No children aged 15 or older
                             4        .m  .m. Missing
                             2        .w  .w. Answered 6 wk survey at 6
                                          mon visit
                           755        .x  .x. No 6 month survey

--------------------------------------------------------------------------------------------
m6_107b                               107: Children: HIV care and treatment in last 6 months
--------------------------------------------------------------------------------------------

                  type:  numeric (byte)
                 label:  m6_107

                 range:  [0,88]                       units:  1
         unique values:  3                        missing .:  67/4168
       unique mv codes:  5                       missing .*:  3668/4168

            tabulation:  Freq.   Numeric  Label
                           364         0  0. No
                            39         1  1. Yes
                            30        88  88. Don't Know
                            67         .  
                          2907        .a  .a. No children aged 15 or older
                             4        .m  .m. Missing
                             2        .w  .w. Answered 6 wk survey at 6
                                          mon visit
                           755        .x  .x. No 6 month survey

--------------------------------------------------------------------------------------------
m6_107c                               107: Children: STD care and treatment in last 6 months
--------------------------------------------------------------------------------------------

                  type:  numeric (byte)
                 label:  m6_107

                 range:  [0,88]                       units:  1
         unique values:  3                        missing .:  67/4168
       unique mv codes:  5                       missing .*:  3668/4168

            tabulation:  Freq.   Numeric  Label
                           393         0  0. No
                             9         1  1. Yes
                            31        88  88. Don't Know
                            67         .  
                          2907        .a  .a. No children aged 15 or older
                             4        .m  .m. Missing
                             2        .w  .w. Answered 6 wk survey at 6
                                          mon visit
                           755        .x  .x. No 6 month survey

--------------------------------------------------------------------------------------------
m6_107d                                      107: Children: Family planning in last 6 months
--------------------------------------------------------------------------------------------
```

```
                type:  numeric (byte)
               label:  m6_107

               range:  [0,88]                       units:  1
       unique values:  3                        missing .:  67/4168
     unique mv codes:  5                       missing .*:  3668/4168

          tabulation:  Freq.   Numeric  Label
                         385         0  0. No
                          19         1  1. Yes
                          29        88  88. Don't Know
                          67         .
                        2907        .a  .a. No children aged 15 or older
                           4        .m  .m. Missing
                           2        .w  .w. Answered 6 wk survey at 6
                                        mon visit
                         755        .x  .x. No 6 month survey

--------------------------------------------------------------------------------------------
m6_107e                                          107: Children: Cervical cancer screening
--------------------------------------------------------------------------------------------

                type:  numeric (byte)
               label:  m6_107

               range:  [0,88]                       units:  1
       unique values:  3                        missing .:  67/4168
     unique mv codes:  5                       missing .*:  3668/4168

          tabulation:  Freq.   Numeric  Label
                         379         0  0. No
                          31         1  1. Yes
                          23        88  88. Don't Know
                          67         .
                        2907        .a  .a. No children aged 15 or older
                           4        .m  .m. Missing
                           2        .w  .w. Answered 6 wk survey at 6
                                        mon visit
                         755        .x  .x. No 6 month survey

--------------------------------------------------------------------------------------------
m6_107f                                   107: Children: Male circumcision in last 6 months
--------------------------------------------------------------------------------------------

                type:  numeric (byte)
               label:  m6_107

               range:  [0,88]                       units:  1
       unique values:  3                        missing .:  67/4168
     unique mv codes:  5                       missing .*:  3668/4168

          tabulation:  Freq.   Numeric  Label
                         384         0  0. No
                          30         1  1. Yes
                          19        88  88. Don't Know
                          67         .
                        2907        .a  .a. No children aged 15 or older
                           4        .m  .m. Missing
                           2        .w  .w. Answered 6 wk survey at 6
                                        mon visit
                         755        .x  .x. No 6 month survey

--------------------------------------------------------------------------------------------
m6_200           200: HTC: Respondent understands to only include services from last 6 months
--------------------------------------------------------------------------------------------

                type:  numeric (byte)
               label:  yesnoserv

               range:  [1,1]                        units:  1
```

```
        unique values:  1                        missing .:  67/4168
      unique mv codes:  6                       missing .*:  3061/4168

          tabulation:  Freq.   Numeric  Label
                        1040         1  1. Yes
                          67         .
                        2299        .a  .a. Did not receive service
                           4        .m  .m. Missing
                           1        .s  .s. Skipped by participant
                           2        .w  .w. Answered 6 mon survey at 6
                                        wk visit
                         755        .x  .x. No 6 week survey

-----------------------------------------------------------------------------------------------
m6_201                                              201: HTC: Number times to health facility
-----------------------------------------------------------------------------------------------

                 type:  numeric (byte)
                label:  numserv, but 6 nonmissing values are not labeled

                range:  [1,6]                        units:  1
        unique values:  6                        missing .:  67/4168
      unique mv codes:  6                       missing .*:  3065/4168

          tabulation:  Freq.   Numeric  Label
                         635         1
                         280         2
                          80         3
                          23         4
                           8         5
                          10         6
                          67         .
                        2299        .a  .a. Did not receive service
                           4        .m  .m. Missing
                           5        .s  .s. Skipped by participant
                           2        .w  .w. Answered 6 mon survey at 6
                                        wk visit
                         755        .x  .x. No 6 week survey

-----------------------------------------------------------------------------------------------
m6_202                                                         202: HTC: Health facility name
-----------------------------------------------------------------------------------------------

                 type:  string (str41)

        unique values:  379                      missing "":  823/4168

             examples:  "."
                        "."
                        "."
                        "Chipata general Hospital"

              warning:  variable has embedded blanks

-----------------------------------------------------------------------------------------------
m6_203                                                         203: HTC: Alone or accompanied
-----------------------------------------------------------------------------------------------

                 type:  numeric (byte)
                label:  hfpeople

                range:  [1,3]                        units:  1
        unique values:  3                        missing .:  67/4168
      unique mv codes:  6                       missing .*:  3064/4168

          tabulation:  Freq.   Numeric  Label
                         647         1  1. Alone
                         299         2  2. Spouse/Partner
                          91         3  3. Someone else
                          67         .
```

```
                    2299        .a  .a. Did not receive service
                       4        .m  .m. Missing
                       4        .s  .s. Skipped by participant
                       2        .w  .w. Answered 6 mon survey at 6
                                    wk visit
                     755        .x  .x. No 6 week survey

-----------------------------------------------------------------------------------------------
m6_204                                                      204: HTC: Satisfied with services
-----------------------------------------------------------------------------------------------

                  type:  numeric (byte)
                 label:  hfsat

                 range:  [1,3]                        units:  1
         unique values:  3                        missing .:  67/4168
       unique mv codes:  6                       missing .*:  3063/4168

            tabulation:  Freq.   Numeric  Label
                            32         1  1. Not satisfied
                            21         2  2. Neither
                           985         3  3. Satisfied
                            67         .
                          2299        .a  .a. Did not receive service
                             4        .m  .m. Missing
                             3        .s  .s. Skipped by participant
                             2        .w  .w. Answered 6 mon survey at 6
                                          wk visit
                           755        .x  .x. No 6 week survey

-----------------------------------------------------------------------------------------------
m6_205                                         205: HTC: Waiting time at health facility (min)
-----------------------------------------------------------------------------------------------

                  type:  numeric (int)
                 label:  numserv, but 61 nonmissing values are not labeled

                 range:  [1,2400]                     units:  1
         unique values:  61                       missing .:  67/4168
       unique mv codes:  6                       missing .*:  3066/4168

              examples:  60
                         .a    .a. Did not receive service
                         .a    .a. Did not receive service
                         .a    .a. Did not receive service

-----------------------------------------------------------------------------------------------
m6_206                                          206: HTC: Travel time to health facility (min)
-----------------------------------------------------------------------------------------------

                  type:  numeric (int)
                 label:  hfttime, but 56 nonmissing values are not labeled

                 range:  [1,300]                      units:  1
         unique values:  56                       missing .:  67/4168
       unique mv codes:  6                       missing .*:  3072/4168

              examples:  60
                         .a    .a. Did not receive service
                         .a    .a. Did not receive service
                         .a    .a. Did not receive service

-----------------------------------------------------------------------------------------------
m6_207                                              207: HTC: Travel mode to health faciity
-----------------------------------------------------------------------------------------------

                  type:  numeric (byte)
                 label:  hftmode

                 range:  [1,7]                        units:  1
```

```
        unique values:  7                        missing .:  67/4168
      unique mv codes:  6                       missing .*:  3066/4168

          tabulation:  Freq.   Numeric  Label
                         322         1  1. Bus
                          75         2  2. Taxi
                          34         3  3. Someone drove in private car
                          38         4  4. Drove self
                         518         5  5. Walked
                          40         6  6. Bicycle
                           8         7  7. Other
                          67         .
                        2299        .a  .a. Did not receive service
                           4        .m  .m. Missing
                           6        .s  .s. Skipped by participant
                           2        .w  .w. Answered 6 mon survey at 6
                                        wk visit
                         755        .x  .x. No 6 week survey

-------------------------------------------------------------------------------------------
m6_208                                                            208: HTC: Transport cost
-------------------------------------------------------------------------------------------

                 type:  numeric (byte)
                label:  hftcost, but 31 nonmissing values are not labeled

                range:  [0,100]                      units:  1
        unique values:  32                       missing .:  67/4168
      unique mv codes:  7                       missing .*:  3661/4168

             examples:  .a    .a. Did not receive service
                        .a    .a. Did not receive service
                        .a    .a. Did not receive service
                        .b    .b. Own car, walked, biked

-------------------------------------------------------------------------------------------
m6_209                                                    209: HTC: Distance traveled (km)
-------------------------------------------------------------------------------------------

                 type:  numeric (int)
                label:  hftkm, but 29 nonmissing values are not labeled

                range:  [0,888]                      units:  1
        unique values:  31                       missing .:  67/4168
      unique mv codes:  6                       missing .*:  3153/4168

             examples:  888   888. Don't Know
                        .a    .a. Did not receive service
                        .a    .a. Did not receive service
                        .s    .s. Skipped by participant

-------------------------------------------------------------------------------------------
m6_210                                                 210: HTC: Source of transport money
-------------------------------------------------------------------------------------------

                 type:  numeric (byte)
                label:  hftsource

                range:  [1,6]                        units:  1
        unique values:  6                        missing .:  67/4168
      unique mv codes:  8                       missing .*:  3694/4168

          tabulation:  Freq.   Numeric  Label
                          16         1  1. Cutting down on other
                                        expenses
                         265         2  2. Savings
                           5         3  3. Borrowing
                           1         4  4. Selling assets
                          99         5  5. Donation
                          21         6  6. Other
```

Freq.	Numeric	Label
67	.	
2299	.a	.a. Did not receive service
31	.b	.b. Did not pay for transport
596	.c	.c. Own car, walked, biked
4	.m	.m. Missing
7	.s	.s. Skipped by participant
2	.w	.w. Answered 6 mon survey at 6 wk visit
755	.x	.x. No 6 week survey

---

m6_211 211: HTC: What respondent would be doing if not at health facility

---

type: numeric (byte)
label: hfnot

range: [1,6] units: 1
unique values: 6 missing .: 67/4168
unique mv codes: 6 missing .*: 3064/4168

tabulation:

Freq.	Numeric	Label
257	1	1. Paid employment
160	2	2. Own business
272	3	3. Unpaid work/Housework
81	4	4. In school
190	5	5. Resting/No specific activity
77	6	6. Other
67	.	
2299	.a	.a. Did not receive service
4	.m	.m. Missing
4	.s	.s. Skipped by participant
2	.w	.w. Answered 6 mon survey at 6 wk visit
755	.x	.x. No 6 week survey

---

m6_300 300: HIV Care/Trt: Respondent understands to only include services from last 6 m

---

type: numeric (byte)
label: yesnoserv

range: [1,1] units: 1
unique values: 1 missing .: 67/4168
unique mv codes: 6 missing .*: 3801/4168

tabulation:

Freq.	Numeric	Label
300	1	1. Yes
67	.	
3039	.a	.a. Did not receive service
4	.m	.m. Missing
1	.s	.s. Skipped by participant
2	.w	.w. Answered 6 mon survey at 6 wk visit
755	.x	.x. No 6 week survey

---

m6_301 301: HIV Care/Trt: Number times to health facility

---

type: numeric (byte)
label: numserv, but 6 nonmissing values are not labeled

range: [1,6] units: 1
unique values: 6 missing .: 67/4168
unique mv codes: 6 missing .*: 3814/4168

tabulation:

Freq.	Numeric	Label
106	1	

```
                          86         2
                          49         3
                          19         4
                          12         5
                          15         6
                          67         .
                        3039        .a  .a. Did not receive service
                           4        .m  .m. Missing
                          14        .s  .s. Skipped by participant
                           2        .w  .w. Answered 6 mon survey at 6
                                        wk visit
                         755        .x  .x. No 6 week survey

-----------------------------------------------------------------------------------------------
m6_302                                                 302: HIV Care/Trt: Health facility name
-----------------------------------------------------------------------------------------------

                  type:  string (str33)

         unique values:  102                      missing "":  823/4168

              examples:  "."
                         "."
                         "."
                         "."

               warning:  variable has embedded blanks

-----------------------------------------------------------------------------------------------
m6_303                                                 303: HIV Care/Trt: Alone or accompanied
-----------------------------------------------------------------------------------------------

                  type:  numeric (byte)
                 label:  hfpeople

                 range:  [1,3]                         units:  1
         unique values:  3                         missing .:  67/4168
       unique mv codes:  6                        missing .*:  3816/4168

            tabulation:  Freq.   Numeric  Label
                           210         1  1. Alone
                            55         2  2. Spouse/Partner
                            20         3  3. Someone else
                            67         .
                          3039        .a  .a. Did not receive service
                             4        .m  .m. Missing
                            16        .s  .s. Skipped by participant
                             2        .w  .w. Answered 6 mon survey at 6
                                          wk visit
                           755        .x  .x. No 6 week survey

-----------------------------------------------------------------------------------------------
m6_304                                              304: HIV Care/Trt: Satisfied with services
-----------------------------------------------------------------------------------------------

                  type:  numeric (byte)
                 label:  hfsat

                 range:  [1,3]                         units:  1
         unique values:  3                         missing .:  67/4168
       unique mv codes:  6                        missing .*:  3816/4168

            tabulation:  Freq.   Numeric  Label
                            12         1  1. Not satisfied
                             9         2  2. Neither
                           264         3  3. Satisfied
                            67         .
                          3039        .a  .a. Did not receive service
                             4        .m  .m. Missing
                            16        .s  .s. Skipped by participant
```

```
                            2         .w  .w. Answered 6 mon survey at 6
                                          wk visit
                          755         .x  .x. No 6 week survey

-------------------------------------------------------------------------------
m6_305                            305: HIV Care/Trt:  Waiting time at health facility (min)
-------------------------------------------------------------------------------

                  type:  numeric (int)
                 label:  numserv, but 46 nonmissing values are not labeled

                 range:  [1,840]                      units:  1
         unique values:  46                       missing .:  67/4168
       unique mv codes:  6                       missing .*:  3818/4168

              examples:  .a    .a. Did not receive service
                         .a    .a. Did not receive service
                         .a    .a. Did not receive service
                         .a    .a. Did not receive service

-------------------------------------------------------------------------------
m6_306                                             306: HIV Care/Trt: TB testing
-------------------------------------------------------------------------------

                  type:  numeric (byte)
                 label:  yesnoserv

                 range:  [0,1]                        units:  1
         unique values:  2                        missing .:  67/4168
       unique mv codes:  6                       missing .*:  3805/4168

            tabulation:  Freq.   Numeric  Label
                           215         0  0. No
                            81         1  1. Yes
                            67         .  
                          3039        .a  .a. Did not receive service
                             4        .m  .m. Missing
                             5        .s  .s. Skipped by participant
                             2        .w  .w. Answered 6 mon survey at 6
                                          wk visit
                           755        .x  .x. No 6 week survey

-------------------------------------------------------------------------------
m6_307                                            307: HIV Care/Trt: CD4 testing
-------------------------------------------------------------------------------

                  type:  numeric (byte)
                 label:  yesnoserv

                 range:  [0,88]                       units:  1
         unique values:  3                        missing .:  67/4168
       unique mv codes:  6                       missing .*:  3805/4168

            tabulation:  Freq.   Numeric  Label
                            89         0  0. No
                           206         1  1. Yes
                             1        88  88. Don't Know
                            67         .  
                          3039        .a  .a. Did not receive service
                             4        .m  .m. Missing
                             5        .s  .s. Skipped by participant
                             2        .w  .w. Answered 6 mon survey at 6
                                          wk visit
                           755        .x  .x. No 6 week survey

-------------------------------------------------------------------------------
m6_308                                       308: HIV Care/Trt: Eligible for ARV
-------------------------------------------------------------------------------

                  type:  numeric (byte)
```

```
                label:  m6_308

                range:  [0,1]                        units:  1
        unique values:  2                        missing .:  67/4168
      unique mv codes:  7                       missing .*:  3894/4168

           tabulation:  Freq.   Numeric  Label
                           50         0  0. No
                          157         1  1. Yes
                           67         .  
                         3039        .a  .a. Did not receive service
                           89        .b  .b. Have not received CD4
                                         testing
                            4        .m  .m. Missing
                            5        .s  .s. Skipped by participant
                            2        .w  .w. Answered 6 wk survey at 6
                                         mon visit
                          755        .x  .x. No 6 month survey

-----------------------------------------------------------------------------------------------
m6_309                                                       309: HIV Care/Trt: Initiated ARV
-----------------------------------------------------------------------------------------------

                 type:  numeric (byte)
                label:  m6_309

                range:  [0,1]                        units:  1
        unique values:  2                        missing .:  67/4168
      unique mv codes:  8                       missing .*:  3944/4168

           tabulation:  Freq.   Numeric  Label
                            4         0  0. No
                          153         1  1. Yes
                           67         .  
                         3039        .a  .a. Did not receive service
                           89        .b  .b. Have not received CD4
                                         testing
                           50        .c  .c. Not eligible for ARV
                            4        .m  .m. Missing
                            5        .s  .s. Skipped by participant
                            2        .w  .w. Answered 6 wk survey at 6
                                         mon visit
                          755        .x  .x. No 6 month survey

-----------------------------------------------------------------------------------------------
m6_310                             310: HIV Care/Trt: Number missed follow-ups since started ARV
-----------------------------------------------------------------------------------------------

                 type:  numeric (byte)
                label:  m6_310, but 6 nonmissing values are not labeled

                range:  [0,6]                        units:  1
        unique values:  7                        missing .:  67/4168
      unique mv codes:  9                       missing .*:  3948/4168

           tabulation:  Freq.   Numeric  Label
                          131         0  0. Never
                           10         1  
                            5         2  
                            3         3  
                            2         4  
                            1         5  
                            1         6  
                           67         .  
                         3039        .a  .a. Did not receive service
                           89        .b  .b. Have not received CD4
                                         testing
                           50        .c  .c. Not eligible for ARV
                            4        .d  .d. Did not initiate ARV
                            4        .m  .m. Missing
```

```
                     5        .s  .s. Skipped by participant
                     2        .w  .w. Answered 6 wk survey at 6
                                  mon visit
                   755        .x  .x. No 6 month survey

-----------------------------------------------------------------------------------------------
m6_311                                              311: HIV Care/Trt: Number missed doses ARV
-----------------------------------------------------------------------------------------------

                  type:  numeric (byte)
                 label:  m6_311, but 5 nonmissing values are not labeled

                 range:  [0,5]                        units:  1
         unique values:  6                        missing .:  67/4168
       unique mv codes:  9                       missing .*:  3948/4168

            tabulation:  Freq.   Numeric  Label
                           125         0  0. Never
                             6         1
                            10         2
                             5         3
                             4         4
                             3         5
                            67         .
                          3039        .a  .a. Did not receive service
                            89        .b  .b. Have not received CD4
                                          testing
                            50        .c  .c. Not eligible for ARV
                             4        .d  .d. Did not initiate ARV
                             4        .m  .m. Missing
                             5        .s  .s. Skipped by participant
                             2        .w  .w. Answered 6 wk survey at 6
                                          mon visit
                           755        .x  .x. No 6 month survey

-----------------------------------------------------------------------------------------------
m6_312                           312: HIV Care/Trt: Participated in psycho-social support groups
-----------------------------------------------------------------------------------------------

                  type:  numeric (byte)
                 label:  yesnoserv

                 range:  [0,1]                        units:  1
         unique values:  2                        missing .:  67/4168
       unique mv codes:  6                       missing .*:  3806/4168

            tabulation:  Freq.   Numeric  Label
                           252         0  0. No
                            43         1  1. Yes
                            67         .
                          3039        .a  .a. Did not receive service
                             4        .m  .m. Missing
                             6        .s  .s. Skipped by participant
                             2        .w  .w. Answered 6 mon survey at 6
                                          wk visit
                           755        .x  .x. No 6 week survey

-----------------------------------------------------------------------------------------------
m6_313                                  313: HIV Care/Trt: Travel time to health facility (min)
-----------------------------------------------------------------------------------------------

                  type:  numeric (int)
                 label:  hfttime, but 30 nonmissing values are not labeled

                 range:  [5,888]                      units:  1
         unique values:  31                       missing .:  67/4168
       unique mv codes:  6                       missing .*:  3820/4168

              examples:  .a    .a. Did not receive service
                         .a    .a. Did not receive service
```

```
                        .a    .a. Did not receive service
                        .a    .a. Did not receive service

-------------------------------------------------------------------------------------------------
m6_314                                       314: HIV Care/Trt: Travel mode to health faciity
-------------------------------------------------------------------------------------------------

                  type:  numeric (byte)
                 label:  hftmode

                 range:  [1,7]                        units:  1
         unique values:  7                        missing .:  67/4168
       unique mv codes:  6                       missing .*:  3817/4168

            tabulation:  Freq.   Numeric  Label
                           109         1  1. Bus
                            20         2  2. Taxi
                             4         3  3. Someone drove in private car
                             5         4  4. Drove self
                           133         5  5. Walked
                            12         6  6. Bicycle
                             1         7  7. Other
                            67         .
                          3039        .a  .a. Did not receive service
                             4        .m  .m. Missing
                            17        .s  .s. Skipped by participant
                             2        .w  .w. Answered 6 mon survey at 6
                                          wk visit
                           755        .x  .x. No 6 week survey

-------------------------------------------------------------------------------------------------
m6_315                                                      315: HIV Care/Trt: Transport cost
-------------------------------------------------------------------------------------------------

                  type:  numeric (byte)
                 label:  hftcost, but 17 nonmissing values are not labeled

                 range:  [0,60]                       units:  1
         unique values:  18                       missing .:  67/4168
       unique mv codes:  7                       missing .*:  3966/4168

            tabulation:  Freq.   Numeric  Label
                             6         0  0. Nothing
                             3         1
                            18         2
                            17         3
                            19         4
                            23         5
                             2         6
                             3         7
                             4         8
                             2         9
                            14        10
                             5        15
                             2        16
                            10        20
                             4        30
                             1        35
                             1        50
                             1        60
                            67         .
                          3039        .a  .a. Did not receive service
                           150        .b  .b. Own car, walked, biked
                             4        .m  .m. Missing
                            16        .s  .s. Skipped by participant
                             2        .w  .w. Answered 6 mon survey at 6
                                          wk visit
                           755        .x  .x. No 6 week survey

-------------------------------------------------------------------------------------------------
```

```
m6_316                                       316: HIV Care/Trt: Distance traveled (km)
-------------------------------------------------------------------------------------------

                  type:  numeric (int)
                 label:  hftkm, but 17 nonmissing values are not labeled

                 range:  [0,888]                      units:  1
         unique values:  19                       missing .:  67/4168
       unique mv codes:  6                       missing .*:  3846/4168

            tabulation:  Freq.   Numeric  Label
                            27         0  0. <1
                             9         1
                            20         2
                            18         3
                            13         4
                             9         5
                             8         6
                             4         7
                             5         8
                             3         9
                             3        10
                             1        12
                             1        15
                             1        25
                             1        40
                             2        45
                             1        90
                             1       100
                           128       888  888. Don't Know
                            67         .
                          3039        .a  .a. Did not receive service
                             4        .m  .m. Missing
                            46        .s  .s. Skipped by participant
                             2        .w  .w. Answered 6 mon survey at 6
                                          wk visit
                           755        .x  .x. No 6 week survey

-------------------------------------------------------------------------------------------
m6_317                                    317: HIV Care/Trt: Source of transport money
-------------------------------------------------------------------------------------------

                  type:  numeric (byte)
                 label:  hftsource

                 range:  [1,6]                        units:  1
         unique values:  5                        missing .:  67/4168
       unique mv codes:  8                       missing .*:  3973/4168

            tabulation:  Freq.   Numeric  Label
                             8         1  1. Cutting down on other
                                          expenses
                            66         2  2. Savings
                             4         3  3. Borrowing
                            40         5  5. Donation
                            10         6  6. Other
                            67         .
                          3039        .a  .a. Did not receive service
                             6        .b  .b. Did not pay for transport
                           150        .c  .c. Own car, walked, biked
                             4        .m  .m. Missing
                            17        .s  .s. Skipped by participant
                             2        .w  .w. Answered 6 mon survey at 6
                                          wk visit
                           755        .x  .x. No 6 week survey

-------------------------------------------------------------------------------------------
m6_318            318: HIV Care/Trt: What respondent would be doing if not at health facility
-------------------------------------------------------------------------------------------
```

```
                  type:  numeric (byte)
                 label:  hfnot

                 range:  [1,6]                        units:  1
         unique values:  6                        missing .:  67/4168
       unique mv codes:  6                       missing .*:  3818/4168

            tabulation:  Freq.   Numeric  Label
                            49         1  1. Paid employment
                            55         2  2. Own business
                            99         3  3. Unpaid work/Housework
                             4         4  4. In school
                            53         5  5. Resting/No specific activity
                            23         6  6. Other
                            67         .
                          3039        .a  .a. Did not receive service
                             4        .m  .m. Missing
                            18        .s  .s. Skipped by participant
                             2        .w  .w. Answered 6 mon survey at 6
                                          wk visit
                           755        .x  .x. No 6 week survey

-----------------------------------------------------------------------------------------------
m6_400        400: STI Care/Trt: Respondent understands to only include services from last 6 m
-----------------------------------------------------------------------------------------------

                  type:  numeric (byte)
                 label:  yesnoserv

                 range:  [1,1]                        units:  1
         unique values:  1                        missing .:  67/4168
       unique mv codes:  5                       missing .*:  3958/4168

            tabulation:  Freq.   Numeric  Label
                           143         1  1. Yes
                            67         .
                          3197        .a  .a. Did not receive service
                             4        .m  .m. Missing
                             2        .w  .w. Answered 6 mon survey at 6
                                          wk visit
                           755        .x  .x. No 6 week survey

-----------------------------------------------------------------------------------------------
m6_401                                    401: STI Care/Trt: Number times to health facility
-----------------------------------------------------------------------------------------------

                  type:  numeric (byte)
                 label:  numserv, but 6 nonmissing values are not labeled

                 range:  [1,6]                        units:  1
         unique values:  6                        missing .:  67/4168
       unique mv codes:  6                       missing .*:  3963/4168

            tabulation:  Freq.   Numeric  Label
                            80         1
                            28         2
                            21         3
                             2         4
                             2         5
                             5         6
                            67         .
                          3197        .a  .a. Did not receive service
                             4        .m  .m. Missing
                             5        .s  .s. Skipped by participant
                             2        .w  .w. Answered 6 mon survey at 6
                                          wk visit
                           755        .x  .x. No 6 week survey

-----------------------------------------------------------------------------------------------
m6_402                                               402: STI Care/Trt: Health facility name
```

```
------------------------------------------------------------------------------------------

                  type:  string (str57)

         unique values:  86                       missing "":  823/4168

              examples:  "."
                         "."
                         "."
                         "."

               warning:  variable has embedded blanks

------------------------------------------------------------------------------------------
m6_403                                           403: STI Care/Trt: Alone or accompanied
------------------------------------------------------------------------------------------

                  type:  numeric (byte)
                 label:  hfpeople

                 range:  [1,3]                        units:  1
         unique values:  3                        missing .:  67/4168
       unique mv codes:  6                       missing .*:  3961/4168

            tabulation:  Freq.   Numeric  Label
                            95         1  1. Alone
                            32         2  2. Spouse/Partner
                            13         3  3. Someone else
                            67         .
                          3197        .a  .a. Did not receive service
                             4        .m  .m. Missing
                             3        .s  .s. Skipped by participant
                             2        .w  .w. Answered 6 mon survey at 6
                                          wk visit
                           755        .x  .x. No 6 week survey

------------------------------------------------------------------------------------------
m6_404                                        404: STI Care/Trt: Satisfied with services
------------------------------------------------------------------------------------------

                  type:  numeric (byte)
                 label:  hfsat

                 range:  [1,3]                        units:  1
         unique values:  3                        missing .:  67/4168
       unique mv codes:  6                       missing .*:  3961/4168

            tabulation:  Freq.   Numeric  Label
                             7         1  1. Not satisfied
                             2         2  2. Neither
                           131         3  3. Satisfied
                            67         .
                          3197        .a  .a. Did not receive service
                             4        .m  .m. Missing
                             3        .s  .s. Skipped by participant
                             2        .w  .w. Answered 6 mon survey at 6
                                          wk visit
                           755        .x  .x. No 6 week survey

------------------------------------------------------------------------------------------
m6_405                            405: STI Care/Trt:  Waiting time at health facility (min)
------------------------------------------------------------------------------------------

                  type:  numeric (int)
                 label:  numserv, but 35 nonmissing values are not labeled

                 range:  [1,360]                      units:  1
         unique values:  35                       missing .:  67/4168
       unique mv codes:  6                       missing .*:  3964/4168
```

```
           examples:  .a    .a. Did not receive service
                      .a    .a. Did not receive service
                      .a    .a. Did not receive service
                      .a    .a. Did not receive service

-----------------------------------------------------------------------------------------------
m6_406                                              406: STI Care/Trt: Need medicine for STD
-----------------------------------------------------------------------------------------------

                type:  numeric (byte)
               label:  yesnoserv

               range:  [0,1]                        units:  1
       unique values:  2                        missing .:  67/4168
     unique mv codes:  6                       missing .*:  3962/4168

          tabulation:  Freq.   Numeric  Label
                          77         0  0. No
                          62         1  1. Yes
                          67         .
                        3197        .a  .a. Did not receive service
                           4        .m  .m. Missing
                           4        .s  .s. Skipped by participant
                           2        .w  .w. Answered 6 mon survey at 6
                                        wk visit
                         755        .x  .x. No 6 week survey

-----------------------------------------------------------------------------------------------
m6_407                                            407: STI Care/Trt: Place obtained medicine
-----------------------------------------------------------------------------------------------

                type:  numeric (byte)
               label:  m6_407

               range:  [1,3]                        units:  1
       unique values:  3                        missing .:  67/4168
     unique mv codes:  7                       missing .*:  4039/4168

          tabulation:  Freq.   Numeric  Label
                          53         1  1. At health facility
                           7         2  2. Somewhere else
                           2         3  3. Did not get medicine
                          67         .
                        3197        .a  .a. Did not receive service
                          77        .b  .b. Did not need medicine
                           4        .m  .m. Missing
                           4        .s  .s. Skipped by participant
                           2        .w  .w. Answered 6 wk survey at 6
                                        mon visit
                         755        .x  .x. No 6 month survey

-----------------------------------------------------------------------------------------------
m6_408                                                 408: STI Care/Trt: Cost of medicine
-----------------------------------------------------------------------------------------------

                type:  numeric (byte)
               label:  m6_408, but 9 nonmissing values are not labeled

               range:  [0,100]                      units:  1
       unique values:  10                       missing .:  67/4168
     unique mv codes:  8                       missing .*:  4041/4168

          tabulation:  Freq.   Numeric  Label
                          44         0  0. Nothing
                           1        10
                           1        11
                           1        15
                           3        20
                           3        25
                           1        30
```

```
                          1        61
                          1        85
                          4       100
                         67         .
                       3197        .a  .a. Did not receive service
                         77        .b  .b. Did not need medicine
                          2        .c  .c. Did not get medicine
                          4        .m  .m. Missing
                          4        .s  .s. Skipped by participant
                          2        .w  .w. Answered 6 wk survey at 6
                                       mon visit
                        755        .x  .x. No 6 month survey

-----------------------------------------------------------------------------------------------
m6_409                                  409: STI Care/Trt: Travel time to health facility (min)
-----------------------------------------------------------------------------------------------

                  type:  numeric (int)
                 label:  hfttime, but 28 nonmissing values are not labeled

                 range:  [1,888]                      units:  1
         unique values:  29                       missing .:  67/4168
       unique mv codes:  6                       missing .*:  3965/4168

            tabulation:  Freq.   Numeric  Label
                             1         1
                             1         4
                             7         5
                             1         9
                            14        10
                             2        13
                             9        15
                             1        16
                             1        18
                            13        20
                             3        25
                             1        28
                            22        30
                             2        35
                             6        40
                             1        44
                             6        45
                             4        50
                             2        55
                            19        60
                             2        61
                             1        65
                             1        67
                             2        70
                             1        80
                             5       120
                             1       140
                             1       240
                             6       888  888. Don't Know
                            67         .
                          3197        .a  .a. Did not receive service
                             4        .m  .m. Missing
                             7        .s  .s. Skipped by participant
                             2        .w  .w. Answered 6 mon survey at 6
                                          wk visit
                           755        .x  .x. No 6 week survey

-----------------------------------------------------------------------------------------------
m6_410                                         410: STI Care/Trt: Travel mode to health faciity
-----------------------------------------------------------------------------------------------

                  type:  numeric (byte)
                 label:  hftmode

                 range:  [1,6]                        units:  1
```

```
        unique values:  6                        missing .:  67/4168
      unique mv codes:  6                       missing .*:  3964/4168

          tabulation:  Freq.   Numeric  Label
                          50         1  1. Bus
                           7         2  2. Taxi
                           1         3  3. Someone drove in private car
                           4         4  4. Drove self
                          69         5  5. Walked
                           6         6  6. Bicycle
                          67         .
                        3197        .a  .a. Did not receive service
                           4        .m  .m. Missing
                           6        .s  .s. Skipped by participant
                           2        .w  .w. Answered 6 mon survey at 6
                                        wk visit
                         755        .x  .x. No 6 week survey

-----------------------------------------------------------------------------------------------
m6_411                                                       411: STI Care/Trt: Transport cost
-----------------------------------------------------------------------------------------------

                 type:  numeric (byte)
                label:  hftcost, but 15 nonmissing values are not labeled

                range:  [0,70]                       units:  1
        unique values:  16                       missing .:  67/4168
      unique mv codes:  7                       missing .*:  4041/4168

          tabulation:  Freq.   Numeric  Label
                           4         0  0. Nothing
                           6         2
                          10         3
                          10         4
                           6         5
                           2         6
                           2         7
                           2         8
                          10        10
                           1        14
                           1        15
                           1        16
                           2        20
                           1        25
                           1        50
                           1        70
                          67         .
                        3197        .a  .a. Did not receive service
                          79        .b  .b. Own car, walked, biked
                           4        .m  .m. Missing
                           4        .s  .s. Skipped by participant
                           2        .w  .w. Answered 6 mon survey at 6
                                        wk visit
                         755        .x  .x. No 6 week survey

-----------------------------------------------------------------------------------------------
m6_412                                                412: STI Care/Trt: Distance traveled (km)
-----------------------------------------------------------------------------------------------

                 type:  numeric (byte)
                label:  hftkm, but 14 nonmissing values are not labeled

                range:  [0,88]                       units:  1
        unique values:  15                       missing .:  67/4168
      unique mv codes:  6                       missing .*:  3970/4168

          tabulation:  Freq.   Numeric  Label
                           8         0  0. <1
                           8         1
                           9         2
```

```
                        21         3
                        12         4
                         9         5
                         5         6
                         2         7
                         1         8
                         1         9
                         1        10
                         2        12
                         1        15
                         1        40
                        50        88
                        67         .
                      3197        .a  .a. Did not receive service
                         4        .m  .m. Missing
                        12        .s  .s. Skipped by participant
                         2        .w  .w. Answered 6 mon survey at 6
                                      wk visit
                       755        .x  .x. No 6 week survey

-----------------------------------------------------------------------------------------------
m6_413                                           413: STI Care/Trt: Source of transport money
-----------------------------------------------------------------------------------------------

                  type:  numeric (byte)
                 label:  hftsource

                 range:  [1,6]                        units:  1
         unique values:  5                        missing .:  67/4168
       unique mv codes:  8                       missing .*:  4045/4168

            tabulation:  Freq.   Numeric  Label
                             1         1  1. Cutting down on other
                                          expenses
                            40         2  2. Savings
                             2         3  3. Borrowing
                            11         5  5. Donation
                             2         6  6. Other
                            67         .
                          3197        .a  .a. Did not receive service
                             4        .b  .b. Did not pay for transport
                            79        .c  .c. Own car, walked, biked
                             4        .m  .m. Missing
                             4        .s  .s. Skipped by participant
                             2        .w  .w. Answered 6 mon survey at 6
                                          wk visit
                           755        .x  .x. No 6 week survey

-----------------------------------------------------------------------------------------------
m6_414             414: STI Care/Trt: What respondent would be doing if not at health facility
-----------------------------------------------------------------------------------------------

                  type:  numeric (byte)
                 label:  hfnot

                 range:  [1,6]                        units:  1
         unique values:  6                        missing .:  67/4168
       unique mv codes:  6                       missing .*:  3965/4168

            tabulation:  Freq.   Numeric  Label
                            28         1  1. Paid employment
                            25         2  2. Own business
                            36         3  3. Unpaid work/Housework
                             3         4  4. In school
                            31         5  5. Resting/No specific activity
                            13         6  6. Other
                            67         .
                          3197        .a  .a. Did not receive service
                             4        .m  .m. Missing
                             7        .s  .s. Skipped by participant
```

```
                          2        .w  .w. Answered 6 mon survey at 6
                                       wk visit
                        755        .x  .x. No 6 week survey

-------------------------------------------------------------------------------------------------
m6_500_f           500: FP: Respondent understands to only include services from last 6 months
-------------------------------------------------------------------------------------------------

                  type:  numeric (byte)
                 label:  yesnoserv

                 range:  [1,1]                        units:  1
         unique values:  1                        missing .:  67/4168
       unique mv codes:  6                       missing .*:  3890/4168

            tabulation:  Freq.   Numeric  Label
                           211         1  1. Yes
                            67         .
                          1514        .a  .a. Did not receive service
                          1615        .g  .g. Gender skip pattern
                             4        .m  .m. Missing
                             2        .w  .w. Answered 6 mon survey at 6
                                          wk visit
                           755        .x  .x. No 6 week survey

-------------------------------------------------------------------------------------------------
m6_501_f                                            501: FP: Number times to health facility
-------------------------------------------------------------------------------------------------

                  type:  numeric (byte)
                 label:  numserv, but 6 nonmissing values are not labeled

                 range:  [1,6]                        units:  1
         unique values:  6                        missing .:  67/4168
       unique mv codes:  7                       missing .*:  3893/4168

            tabulation:  Freq.   Numeric  Label
                           107         1
                            68         2
                            25         3
                             2         4
                             2         5
                             4         6
                            67         .
                          1514        .a  .a. Did not receive service
                          1615        .g  .g. Gender skip pattern
                             4        .m  .m. Missing
                             3        .s  .s. Skipped by participant
                             2        .w  .w. Answered 6 mon survey at 6
                                          wk visit
                           755        .x  .x. No 6 week survey

-------------------------------------------------------------------------------------------------
m6_502_f                                                      502: FP: Health facility name
-------------------------------------------------------------------------------------------------

                  type:  string (str33)

         unique values:  86                       missing "":  823/4168

              examples:  "."
                         "."
                         "."
                         "."

               warning:  variable has embedded blanks

-------------------------------------------------------------------------------------------------
m6_503_f                                                      503: FP: Alone or accompanied
-------------------------------------------------------------------------------------------------
```

```
                  type:  numeric (byte)
                 label:  hfpeople

                 range:  [1,3]                        units:  1
         unique values:  3                        missing .:  67/4168
       unique mv codes:  7                       missing .*:  3893/4168

            tabulation:  Freq.   Numeric  Label
                           181         1  1. Alone
                            19         2  2. Spouse/Partner
                             8         3  3. Someone else
                            67         .
                          1514        .a  .a. Did not receive service
                          1615        .g  .g. Gender skip pattern
                             4        .m  .m. Missing
                             3        .s  .s. Skipped by participant
                             2        .w  .w. Answered 6 mon survey at 6
                                          wk visit
                           755        .x  .x. No 6 week survey

-----------------------------------------------------------------------------------------------
m6_504_f                                                          504: FP: Satisfied with services
-----------------------------------------------------------------------------------------------

                  type:  numeric (byte)
                 label:  hfsat

                 range:  [1,3]                        units:  1
         unique values:  3                        missing .:  67/4168
       unique mv codes:  7                       missing .*:  3893/4168

            tabulation:  Freq.   Numeric  Label
                             6         1  1. Not satisfied
                             6         2  2. Neither
                           196         3  3. Satisfied
                            67         .
                          1514        .a  .a. Did not receive service
                          1615        .g  .g. Gender skip pattern
                             4        .m  .m. Missing
                             3        .s  .s. Skipped by participant
                             2        .w  .w. Answered 6 mon survey at 6
                                          wk visit
                           755        .x  .x. No 6 week survey

-----------------------------------------------------------------------------------------------
m6_505_f                                         505: FP:  Waiting time at health facility (min)
-----------------------------------------------------------------------------------------------

                  type:  numeric (int)
                 label:  numserv, but 34 nonmissing values are not labeled

                 range:  [1,240]                      units:  1
         unique values:  34                       missing .:  67/4168
       unique mv codes:  7                       missing .*:  3898/4168

              examples:  .a    .a. Did not receive service
                         .a    .a. Did not receive service
                         .g    .g. Gender skip pattern
                         .g    .g. Gender skip pattern

-----------------------------------------------------------------------------------------------
m6_506_f                                                               506: FP: Currently pregnant
-----------------------------------------------------------------------------------------------

                  type:  numeric (byte)
                 label:  yesnoserv

                 range:  [0,1]                        units:  1
         unique values:  2                        missing .:  67/4168
```

```
      unique mv codes:  7                      missing .*:  3891/4168

          tabulation:  Freq.   Numeric  Label
                         209         0  0. No
                           1         1  1. Yes
                          67         .
                        1514        .a  .a. Did not receive service
                        1615        .g  .g. Gender skip pattern
                           4        .m  .m. Missing
                           1        .s  .s. Skipped by participant
                           2        .w  .w. Answered 6 mon survey at 6
                                        wk visit
                         755        .x  .x. No 6 week survey

-------------------------------------------------------------------------------
m6_507_f                                             507: FP: Currently using FP
-------------------------------------------------------------------------------

                 type:  numeric (byte)
                label:  m6_507

                range:  [0,1]                        units:  1
        unique values:  2                        missing .:  67/4168
      unique mv codes:  8                       missing .*:  3892/4168

          tabulation:  Freq.   Numeric  Label
                           9         0  0. No
                         200         1  1. Yes
                          67         .
                        1514        .a  .a. Did not receive service
                           1        .b  .b. Currently pregnant
                        1615        .g  .g. Gender skip pattern
                           4        .m  .m. Missing
                           1        .s  .s. Skipped by participant
                           2        .w  .w. Answered 6 wk survey at 6
                                        mon visit
                         755        .x  .x. No 6 month survey

-------------------------------------------------------------------------------
m6_508a_f                                  508: FP: Method: Female sterilization
-------------------------------------------------------------------------------

                 type:  numeric (byte)
                label:  m6_508a

                range:  [0,0]                        units:  1
        unique values:  1                        missing .:  67/4168
      unique mv codes:  8                       missing .*:  3900/4168

          tabulation:  Freq.   Numeric  Label
                         201         0  0. No
                          67         .
                        1514        .a  .a. Did not receive service
                           1        .b  .b. Currently pregnant
                           9        .c  .c. Not using any method
                        1615        .g  .g. Gender skip pattern
                           4        .m  .m. Missing
                           2        .w  .w. Answered 6 wk survey at 6
                                        mon visit
                         755        .x  .x. No 6 month survey

-------------------------------------------------------------------------------
m6_508b_f                                    508: FP: Method: Male sterilization
-------------------------------------------------------------------------------

                 type:  numeric (byte)
                label:  m6_508b

                range:  [0,0]                        units:  1
        unique values:  1                        missing .:  67/4168
```

```
      unique mv codes:  8                       missing .*:  3900/4168

          tabulation:  Freq.   Numeric  Label
                         201         0  0. No
                          67         .
                        1514        .a  .a. Did not receive service
                           1        .b  .b. Currently pregnant
                           9        .c  .c. Not using any method
                        1615        .g  .g. Gender skip pattern
                           4        .m  .m. Missing
                           2        .w  .w. Answered 6 wk survey at 6
                                        mon visit
                         755        .x  .x. No 6 month survey

------------------------------------------------------------------------------------------
m6_508c_f                                                          508: FP: Method: Pill
------------------------------------------------------------------------------------------

                type:  numeric (byte)
               label:  m6_508c

               range:  [0,1]                        units:  1
       unique values:  2                        missing .:  67/4168
      unique mv codes:  8                       missing .*:  3900/4168

          tabulation:  Freq.   Numeric  Label
                         157         0  0. No
                          44         1  1. Yes
                          67         .
                        1514        .a  .a. Did not receive service
                           1        .b  .b. Currently pregnant
                           9        .c  .c. Not using any method
                        1615        .g  .g. Gender skip pattern
                           4        .m  .m. Missing
                           2        .w  .w. Answered 6 wk survey at 6
                                        mon visit
                         755        .x  .x. No 6 month survey

------------------------------------------------------------------------------------------
m6_508d_f                                                           508: FP: Method: IUD
------------------------------------------------------------------------------------------

                type:  numeric (byte)
               label:  m6_508d

               range:  [0,1]                        units:  1
       unique values:  2                        missing .:  67/4168
      unique mv codes:  8                       missing .*:  3900/4168

          tabulation:  Freq.   Numeric  Label
                         193         0  0. No
                           8         1  1. Yes
                          67         .
                        1514        .a  .a. Did not receive service
                           1        .b  .b. Currently pregnant
                           9        .c  .c. Not using any method
                        1615        .g  .g. Gender skip pattern
                           4        .m  .m. Missing
                           2        .w  .w. Answered 6 wk survey at 6
                                        mon visit
                         755        .x  .x. No 6 month survey

------------------------------------------------------------------------------------------
m6_508e_f                                                   508: FP: Method: Injectables
------------------------------------------------------------------------------------------

                type:  numeric (byte)
               label:  m6_508e

               range:  [0,1]                        units:  1
```

```
        unique values:  2                         missing .:  67/4168
      unique mv codes:  8                        missing .*:  3900/4168

          tabulation:  Freq.   Numeric  Label
                          90         0  0. No
                         111         1  1. Yes
                          67         .
                        1514        .a  .a. Did not receive service
                           1        .b  .b. Currently pregnant
                           9        .c  .c. Not using any method
                        1615        .g  .g. Gender skip pattern
                           4        .m  .m. Missing
                           2        .w  .w. Answered 6 wk survey at 6
                                        mon visit
                         755        .x  .x. No 6 month survey

-------------------------------------------------------------------------------
m6_508f_f                                          508: FP: Method: Implants
-------------------------------------------------------------------------------

                 type:  numeric (byte)
                label:  m6_508f

                range:  [0,1]                         units:  1
        unique values:  2                         missing .:  67/4168
      unique mv codes:  8                        missing .*:  3900/4168

          tabulation:  Freq.   Numeric  Label
                         174         0  0. No
                          27         1  1. Yes
                          67         .
                        1514        .a  .a. Did not receive service
                           1        .b  .b. Currently pregnant
                           9        .c  .c. Not using any method
                        1615        .g  .g. Gender skip pattern
                           4        .m  .m. Missing
                           2        .w  .w. Answered 6 wk survey at 6
                                        mon visit
                         755        .x  .x. No 6 month survey

-------------------------------------------------------------------------------
m6_508g_f                                       508: FP: Method: Male condom
-------------------------------------------------------------------------------

                 type:  numeric (byte)
                label:  m6_508g

                range:  [0,1]                         units:  1
        unique values:  2                         missing .:  67/4168
      unique mv codes:  8                        missing .*:  3900/4168

          tabulation:  Freq.   Numeric  Label
                         182         0  0. No
                          19         1  1. Yes
                          67         .
                        1514        .a  .a. Did not receive service
                           1        .b  .b. Currently pregnant
                           9        .c  .c. Not using any method
                        1615        .g  .g. Gender skip pattern
                           4        .m  .m. Missing
                           2        .w  .w. Answered 6 wk survey at 6
                                        mon visit
                         755        .x  .x. No 6 month survey

-------------------------------------------------------------------------------
m6_508h_f                                     508: FP: Method: Female condom
-------------------------------------------------------------------------------

                 type:  numeric (byte)
                label:  m6_508h
```

```
          range:  [0,0]                        units:  1
  unique values:  1                        missing .:  67/4168
unique mv codes:  8                       missing .*:  3900/4168

    tabulation:  Freq.   Numeric  Label
                   201         0  0. No
                    67         .  
                  1514        .a  .a. Did not receive service
                     1        .b  .b. Currently pregnant
                     9        .c  .c. Not using any method
                  1615        .g  .g. Gender skip pattern
                     4        .m  .m. Missing
                     2        .w  .w. Answered 6 wk survey at 6
                                  mon visit
                   755        .x  .x. No 6 month survey
```

```
------------------------------------------------------------------------------------------
m6_508i_f                                                    508: FP: Method: Diaphragm
------------------------------------------------------------------------------------------

           type:  numeric (byte)
          label:  m6_508i

          range:  [0,0]                        units:  1
  unique values:  1                        missing .:  67/4168
unique mv codes:  8                       missing .*:  3900/4168

    tabulation:  Freq.   Numeric  Label
                   201         0  0. No
                    67         .  
                  1514        .a  .a. Did not receive service
                     1        .b  .b. Currently pregnant
                     9        .c  .c. Not using any method
                  1615        .g  .g. Gender skip pattern
                     4        .m  .m. Missing
                     2        .w  .w. Answered 6 wk survey at 6
                                  mon visit
                   755        .x  .x. No 6 month survey
```

```
------------------------------------------------------------------------------------------
m6_508j_f                                                   508: FP: Method: Foam/Jelly
------------------------------------------------------------------------------------------

           type:  numeric (byte)
          label:  m6_508j

          range:  [0,0]                        units:  1
  unique values:  1                        missing .:  67/4168
unique mv codes:  8                       missing .*:  3900/4168

    tabulation:  Freq.   Numeric  Label
                   201         0  0. No
                    67         .  
                  1514        .a  .a. Did not receive service
                     1        .b  .b. Currently pregnant
                     9        .c  .c. Not using any method
                  1615        .g  .g. Gender skip pattern
                     4        .m  .m. Missing
                     2        .w  .w. Answered 6 wk survey at 6
                                  mon visit
                   755        .x  .x. No 6 month survey
```

```
------------------------------------------------------------------------------------------
m6_508k_f                                 508: FP: Method: Lactational amenorrhea method
------------------------------------------------------------------------------------------

           type:  numeric (byte)
          label:  m6_508k
```

```
                 range:  [0,0]                        units:  1
         unique values:  1                        missing .:  67/4168
       unique mv codes:  8                       missing .*:  3900/4168

            tabulation:  Freq.   Numeric  Label
                           201         0  0. No
                            67         .
                          1514        .a  .a. Did not receive service
                             1        .b  .b. Currently pregnant
                             9        .c  .c. Not using any method
                          1615        .g  .g. Gender skip pattern
                             4        .m  .m. Missing
                             2        .w  .w. Answered 6 wk survey at 6
                                          mon visit
                           755        .x  .x. No 6 month survey

--------------------------------------------------------------------------------------------
m6_508l_f                                                  508: FP: Method: Rhythm method
--------------------------------------------------------------------------------------------

                  type:  numeric (byte)
                 label:  m6_508l

                 range:  [0,0]                        units:  1
         unique values:  1                        missing .:  67/4168
       unique mv codes:  8                       missing .*:  3900/4168

            tabulation:  Freq.   Numeric  Label
                           201         0  0. No
                            67         .
                          1514        .a  .a. Did not receive service
                             1        .b  .b. Currently pregnant
                             9        .c  .c. Not using any method
                          1615        .g  .g. Gender skip pattern
                             4        .m  .m. Missing
                             2        .w  .w. Answered 6 wk survey at 6
                                          mon visit
                           755        .x  .x. No 6 month survey

--------------------------------------------------------------------------------------------
m6_508m_f                                                     508: FP: Method: Withdrawal
--------------------------------------------------------------------------------------------

                  type:  numeric (byte)
                 label:  m6_508m

                 range:  [0,1]                        units:  1
         unique values:  2                        missing .:  67/4168
       unique mv codes:  8                       missing .*:  3900/4168

            tabulation:  Freq.   Numeric  Label
                           200         0  0. No
                             1         1  1. Yes
                            67         .
                          1514        .a  .a. Did not receive service
                             1        .b  .b. Currently pregnant
                             9        .c  .c. Not using any method
                          1615        .g  .g. Gender skip pattern
                             4        .m  .m. Missing
                             2        .w  .w. Answered 6 wk survey at 6
                                          mon visit
                           755        .x  .x. No 6 month survey

--------------------------------------------------------------------------------------------
m6_508n_f                                                   508: FP: Method: Other method
--------------------------------------------------------------------------------------------

                  type:  numeric (byte)
                 label:  m6_508n
```

```
                 range:  [0,1]                        units:  1
         unique values:  2                        missing .:  67/4168
       unique mv codes:  8                       missing .*:  3900/4168

            tabulation:  Freq.   Numeric  Label
                           200         0  0. No
                             1         1  1. Yes
                            67         .  
                          1514        .a  .a. Did not receive service
                             1        .b  .b. Currently pregnant
                             9        .c  .c. Not using any method
                          1615        .g  .g. Gender skip pattern
                             4        .m  .m. Missing
                             2        .w  .w. Answered 6 wk survey at 6
                                          mon visit
                           755        .x  .x. No 6 month survey

-----------------------------------------------------------------------------------------------
m6_509_f                                          509: FP: Number days forgot pill in past month
-----------------------------------------------------------------------------------------------

                  type:  numeric (byte)
                 label:  m6_509, but 6 nonmissing values are not labeled

                 range:  [0,6]                        units:  1
         unique values:  7                        missing .:  67/4168
       unique mv codes:  9                       missing .*:  4057/4168

            tabulation:  Freq.   Numeric  Label
                            26         0  0. Never
                             4         1  
                            10         2  
                             1         3  
                             1         4  
                             1         5  
                             1         6  
                            67         .  
                          1514        .a  .a. Did not receive service
                             1        .b  .b. Currently pregnant
                             9        .c  .c. Not using any method
                           157        .d  .d. Not taking pill
                          1615        .g  .g. Gender skip pattern
                             4        .m  .m. Missing
                             2        .w  .w. Answered 6 wk survey at 6
                                          mon visit
                           755        .x  .x. No 6 month survey

-----------------------------------------------------------------------------------------------
m6_510_f                                            510: FP: Number times had sex without condom
-----------------------------------------------------------------------------------------------

                  type:  numeric (byte)
                 label:  m6_510

                 range:  [.,.]                        units:  .
         unique values:  0                        missing .:  67/4168
       unique mv codes:  10                      missing .*:  4101/4168

            tabulation:  Freq.   Numeric  Label
                            67         .  
                          1514        .a  .a. Did not receive service
                             1        .b  .b. Currently pregnant
                             9        .c  .c. Not using any method
                           182        .d  .d. Not currently using condoms
                            19        .e  .e. Programming skip error: Had
                                          to say yes to male and female
                                          condom to answer this question
                          1615        .g  .g. Gender skip pattern
                             4        .m  .m. Missing
                             2        .w  .w. Answered 6 wk survey at 6
```

```
                                          mon visit
                            755        .x  .x. No 6 month survey

------------------------------------------------------------------------------------
m6_511_f                                     511: FP: Travel time to health facility (min)
------------------------------------------------------------------------------------

                  type:  numeric (int)
                 label:  hfttime, but 28 nonmissing values are not labeled

                 range:  [0,888]                      units:  1
         unique values:  29                       missing .:  67/4168
       unique mv codes:  7                       missing .*:  3895/4168

            tabulation:  Freq.   Numeric  Label
                             1         0
                             1         3
                             1         4
                             9         5
                             1         6
                             2         7
                             1         8
                            23        10
                            17        15
                             1        16
                             1        19
                            29        20
                             1        21
                             4        25
                             1        27
                            47        30
                             1        34
                             2        35
                            12        40
                             2        43
                            14        45
                             1        50
                             1        59
                            19        60
                             1        70
                             3        90
                             1       120
                             2       180
                             7       888  888. Don't Know
                            67         .
                          1514        .a  .a. Did not receive service
                          1615        .g  .g. Gender skip pattern
                             4        .m  .m. Missing
                             5        .s  .s. Skipped by participant
                             2        .w  .w. Answered 6 mon survey at 6
                                          wk visit
                           755        .x  .x. No 6 week survey

------------------------------------------------------------------------------------
m6_512_f                                           512: FP: Travel mode to health faciity
------------------------------------------------------------------------------------

                  type:  numeric (byte)
                 label:  hftmode

                 range:  [1,7]                        units:  1
         unique values:  7                        missing .:  67/4168
       unique mv codes:  7                       missing .*:  3895/4168

            tabulation:  Freq.   Numeric  Label
                            51         1  1. Bus
                             4         2  2. Taxi
                             3         3  3. Someone drove in private car
                             3         4  4. Drove self
                           142         5  5. Walked
```

```
                          2         6  6. Bicycle
                          1         7  7. Other
                         67         .
                       1514        .a  .a. Did not receive service
                       1615        .g  .g. Gender skip pattern
                          4        .m  .m. Missing
                          5        .s  .s. Skipped by participant
                          2        .w  .w. Answered 6 mon survey at 6
                                       wk visit
                        755        .x  .x. No 6 week survey
```

```
-----------------------------------------------------------------------------------------------
m6_513_f                                                                513: FP: Transport cost
-----------------------------------------------------------------------------------------------

                  type:  numeric (byte)
                 label:  hftcost, but 12 nonmissing values are not labeled

                 range:  [0,100]                      units:  1
         unique values:  13                       missing .:  67/4168
       unique mv codes:  8                       missing .*:  4041/4168

            tabulation:  Freq.   Numeric  Label
                             3         0  0. Nothing
                            11         2
                             9         3
                             6         4
                            10         5
                             3         6
                             1         8
                             1         9
                            11        10
                             1        15
                             1        20
                             1        50
                             2       100
                            67         .
                          1514        .a  .a. Did not receive service
                           147        .b  .b. Own car, walked, biked
                          1615        .g  .g. Gender skip pattern
                             4        .m  .m. Missing
                             4        .s  .s. Skipped by participant
                             2        .w  .w. Answered 6 mon survey at 6
                                          wk visit
                           755        .x  .x. No 6 week survey
```

```
-----------------------------------------------------------------------------------------------
m6_514_f                                                        514: FP: Distance traveled (km)
-----------------------------------------------------------------------------------------------

                  type:  numeric (int)
                 label:  hftkm, but 8 nonmissing values are not labeled

                 range:  [0,888]                      units:  1
         unique values:  10                       missing .:  67/4168
       unique mv codes:  7                       missing .*:  3915/4168

            tabulation:  Freq.   Numeric  Label
                            28         0  0. <1
                            10         1
                             9         2
                            21         3
                            17         4
                            13         5
                             4         6
                             3         7
                             1        30
                            80       888  888. Don't Know
                            67         .
                          1514        .a  .a. Did not receive service
```

```
                         1615        .g  .g. Gender skip pattern
                            4        .m  .m. Missing
                           25        .s  .s. Skipped by participant
                            2        .w  .w. Answered 6 mon survey at 6
                                         wk visit
                          755        .x  .x. No 6 week survey
```

```
m6_515_f                                             515: FP: Source of transport money
```

```
                 type:  numeric (byte)
                label:  hftsource

                range:  [1,6]                        units:  1
        unique values:  4                        missing .:  67/4168
      unique mv codes:  9                       missing .*:  4046/4168

           tabulation:  Freq.   Numeric  Label
                            3         1  1. Cutting down on other
                                         expenses
                           36         2  2. Savings
                           13         5  5. Donation
                            3         6  6. Other
                           67         .
                         1514        .a  .a. Did not receive service
                            3        .b  .b. Did not pay for transport
                          147        .c  .c. Own car, walked, biked
                         1615        .g  .g. Gender skip pattern
                            4        .m  .m. Missing
                            6        .s  .s. Skipped by participant
                            2        .w  .w. Answered 6 mon survey at 6
                                         wk visit
                          755        .x  .x. No 6 week survey
```

```
m6_516_f                       516: FP: What respondent would be doing if not at health facility
```

```
                 type:  numeric (byte)
                label:  hfnot

                range:  [1,6]                        units:  1
        unique values:  6                        missing .:  67/4168
      unique mv codes:  7                       missing .*:  3895/4168

           tabulation:  Freq.   Numeric  Label
                           29         1  1. Paid employment
                           32         2  2. Own business
                           83         3  3. Unpaid work/Housework
                            8         4  4. In school
                           49         5  5. Resting/No specific activity
                            5         6  6. Other
                           67         .
                         1514        .a  .a. Did not receive service
                         1615        .g  .g. Gender skip pattern
                            4        .m  .m. Missing
                            5        .s  .s. Skipped by participant
                            2        .w  .w. Answered 6 mon survey at 6
                                         wk visit
                          755        .x  .x. No 6 week survey
```

```
m6_600_f          600: CCS: Respondent understands to only include services from last 6 months
```

```
                 type:  numeric (byte)
                label:  yesnoserv

                range:  [1,1]                        units:  1
```

```
        unique values:  1                        missing .:  67/4168
      unique mv codes:  6                       missing .*:  3792/4168

          tabulation:  Freq.   Numeric  Label
                         309         1  1. Yes
                          67         .
                        1416        .a  .a. Did not receive service
                        1615        .g  .g. Gender skip pattern
                           4        .m  .m. Missing
                           2        .w  .w. Answered 6 mon survey at 6
                                        wk visit
                         755        .x  .x. No 6 week survey

-----------------------------------------------------------------------------------------------
m6_601_f                                            601: CCS: Number times to health facility
-----------------------------------------------------------------------------------------------

                type:  numeric (byte)
               label:  numserv, but 5 nonmissing values are not labeled

               range:  [1,6]                        units:  1
        unique values:  5                        missing .:  67/4168
      unique mv codes:  7                       missing .*:  3796/4168

          tabulation:  Freq.   Numeric  Label
                         269         1
                          31         2
                           3         3
                           1         5
                           1         6
                          67         .
                        1416        .a  .a. Did not receive service
                        1615        .g  .g. Gender skip pattern
                           4        .m  .m. Missing
                           4        .s  .s. Skipped by participant
                           2        .w  .w. Answered 6 mon survey at 6
                                        wk visit
                         755        .x  .x. No 6 week survey

-----------------------------------------------------------------------------------------------
m6_602_f                                                       602: CCS: Health facility name
-----------------------------------------------------------------------------------------------

                type:  string (str40)

        unique values:  69                       missing "":  823/4168

            examples:  "."
                       "."
                       "."
                       "."

             warning:  variable has embedded blanks

-----------------------------------------------------------------------------------------------
m6_603_f                                                       603: CCS: Alone or accompanied
-----------------------------------------------------------------------------------------------

                type:  numeric (byte)
               label:  hfpeople

               range:  [1,3]                        units:  1
        unique values:  3                        missing .:  67/4168
      unique mv codes:  7                       missing .*:  3794/4168

          tabulation:  Freq.   Numeric  Label
                         205         1  1. Alone
                          17         2  2. Spouse/Partner
                          85         3  3. Someone else
                          67         .
```

```
                         1416        .a  .a. Did not receive service
                         1615        .g  .g. Gender skip pattern
                            4        .m  .m. Missing
                            2        .s  .s. Skipped by participant
                            2        .w  .w. Answered 6 mon survey at 6
                                         wk visit
                          755        .x  .x. No 6 week survey

-----------------------------------------------------------------------------------------------
m6_604_f                                                      604: CCS: Satisfied with services
-----------------------------------------------------------------------------------------------

                  type:  numeric (byte)
                 label:  hfsat

                 range:  [1,3]                        units:  1
         unique values:  3                        missing .:  67/4168
       unique mv codes:  7                       missing .*:  3794/4168

            tabulation:  Freq.   Numeric  Label
                             4         1  1. Not satisfied
                             5         2  2. Neither
                           298         3  3. Satisfied
                            67         .  
                          1416        .a  .a. Did not receive service
                          1615        .g  .g. Gender skip pattern
                             4        .m  .m. Missing
                             2        .s  .s. Skipped by participant
                             2        .w  .w. Answered 6 mon survey at 6
                                          wk visit
                           755        .x  .x. No 6 week survey

-----------------------------------------------------------------------------------------------
m6_605_f                                       605: CCS:  Waiting time at health facility (min)
-----------------------------------------------------------------------------------------------

                  type:  numeric (int)
                 label:  numserv, but 39 nonmissing values are not labeled

                 range:  [2,740]                      units:  1
         unique values:  39                       missing .:  67/4168
       unique mv codes:  7                       missing .*:  3794/4168

              examples:  .a    .a. Did not receive service
                         .a    .a. Did not receive service
                         .g    .g. Gender skip pattern
                         .g    .g. Gender skip pattern

-----------------------------------------------------------------------------------------------
m6_606_f                                         606: CCS: Travel time to health facility (min)
-----------------------------------------------------------------------------------------------

                  type:  numeric (int)
                 label:  hfttime, but 31 nonmissing values are not labeled

                 range:  [2,888]                      units:  1
         unique values:  32                       missing .:  67/4168
       unique mv codes:  7                       missing .*:  3795/4168

              examples:  .a    .a. Did not receive service
                         .a    .a. Did not receive service
                         .g    .g. Gender skip pattern
                         .g    .g. Gender skip pattern

-----------------------------------------------------------------------------------------------
m6_607_f                                                607: CCS: Travel mode to health faciity
-----------------------------------------------------------------------------------------------

                  type:  numeric (byte)
                 label:  hftmode
```

```
                range:  [1,6]                        units:  1
        unique values:  5                        missing .:  67/4168
      unique mv codes:  7                       missing .*:  3795/4168

           tabulation:  Freq.   Numeric  Label
                          104         1  1. Bus
                           62         2  2. Taxi
                            6         3  3. Someone drove in private car
                          122         5  5. Walked
                           12         6  6. Bicycle
                           67         .  
                         1416        .a  .a. Did not receive service
                         1615        .g  .g. Gender skip pattern
                            4        .m  .m. Missing
                            3        .s  .s. Skipped by participant
                            2        .w  .w. Answered 6 mon survey at 6
                                         wk visit
                          755        .x  .x. No 6 week survey

-----------------------------------------------------------------------------------------------
m6_608_f                                                               608: CCS: Transport cost
-----------------------------------------------------------------------------------------------

                 type:  numeric (byte)
                label:  hftcost, but 17 nonmissing values are not labeled

                range:  [0,60]                       units:  1
        unique values:  18                       missing .:  67/4168
      unique mv codes:  8                       missing .*:  3928/4168

           tabulation:  Freq.   Numeric  Label
                           39         0  0. Nothing
                            6         1  
                           20         2  
                           18         3  
                           20         4  
                           20         5  
                            6         6  
                            1         8  
                           13        10  
                            6        15  
                           11        20  
                            2        25  
                            4        30  
                            1        32  
                            1        34  
                            2        40  
                            1        50  
                            2        60  
                           67         .  
                         1416        .a  .a. Did not receive service
                          134        .b  .b. Own car, walked, biked
                         1615        .g  .g. Gender skip pattern
                            4        .m  .m. Missing
                            2        .s  .s. Skipped by participant
                            2        .w  .w. Answered 6 mon survey at 6
                                         wk visit
                          755        .x  .x. No 6 week survey

-----------------------------------------------------------------------------------------------
m6_609_f                                                        609: CCS: Distance traveled (km)
-----------------------------------------------------------------------------------------------

                 type:  numeric (int)
                label:  hftkm, but 8 nonmissing values are not labeled

                range:  [0,888]                      units:  1
        unique values:  10                       missing .:  67/4168
      unique mv codes:  7                       missing .*:  3832/4168
```

```
          tabulation:  Freq.   Numeric  Label
                          19         0  0. <1
                           8         1
                          18         2
                          23         3
                          23         4
                          16         5
                           3         6
                           1         8
                           2       100
                         156       888  888. Don't Know
                          67         .
                        1416        .a  .a. Did not receive service
                        1615        .g  .g. Gender skip pattern
                           4        .m  .m. Missing
                          40        .s  .s. Skipped by participant
                           2        .w  .w. Answered 6 mon survey at 6
                                        wk visit
                         755        .x  .x. No 6 week survey

-----------------------------------------------------------------------------------------------
m6_610_f                                                    610: CCS: Source of transport money
-----------------------------------------------------------------------------------------------

                type:  numeric (byte)
               label:  hftsource

               range:  [1,6]                        units:  1
       unique values:  6                        missing .:  67/4168
     unique mv codes:  9                       missing .*:  3970/4168

          tabulation:  Freq.   Numeric  Label
                           6         1  1. Cutting down on other
                                        expenses
                          70         2  2. Savings
                           1         3  3. Borrowing
                           2         4  4. Selling assets
                          40         5  5. Donation
                          12         6  6. Other
                          67         .
                        1416        .a  .a. Did not receive service
                          39        .b  .b. Did not pay for transport
                         134        .c  .c. Own car, walked, biked
                        1615        .g  .g. Gender skip pattern
                           4        .m  .m. Missing
                           5        .s  .s. Skipped by participant
                           2        .w  .w. Answered 6 mon survey at 6
                                        wk visit
                         755        .x  .x. No 6 week survey

-----------------------------------------------------------------------------------------------
m6_611_f                       611: CCS: What respondent would be doing if not at health facility
-----------------------------------------------------------------------------------------------

                type:  numeric (byte)
               label:  hfnot

               range:  [1,6]                        units:  1
       unique values:  6                        missing .:  67/4168
     unique mv codes:  7                       missing .*:  3794/4168

          tabulation:  Freq.   Numeric  Label
                          26         1  1. Paid employment
                          59         2  2. Own business
                         157         3  3. Unpaid work/Housework
                          12         4  4. In school
                          39         5  5. Resting/No specific activity
                          14         6  6. Other
                          67         .
```

```
                        1416        .a  .a. Did not receive service
                        1615        .g  .g. Gender skip pattern
                           4        .m  .m. Missing
                           2        .s  .s. Skipped by participant
                           2        .w  .w. Answered 6 mon survey at 6
                                        wk visit
                         755        .x  .x. No 6 week survey

-------------------------------------------------------------------------------
m6_700_m         700: VMMC: Respondent understands to only include services from last 6 months
-------------------------------------------------------------------------------

                  type:  numeric (byte)
                 label:  yesnoserv

                 range:  [1,1]                        units:  1
         unique values:  1                        missing .:  67/4168
       unique mv codes:  7                       missing .*:  3810/4168

            tabulation:  Freq.   Numeric  Label
                          291         1  1. Yes
                           67         .
                         1314        .a  .a. Did not receive service
                         1729        .g  .g. Gender skip pattern
                            9        .m  .m. Missing
                            1        .s  .s. Skipped by participant
                            2        .w  .w. Answered 6 mon survey at 6
                                         wk visit
                          755        .x  .x. No 6 week survey

-------------------------------------------------------------------------------
m6_701_m                                   701: VMMC: Number times to health facility
-------------------------------------------------------------------------------

                  type:  numeric (byte)
                 label:  numserv, but 6 nonmissing values are not labeled

                 range:  [1,6]                        units:  1
         unique values:  6                        missing .:  67/4168
       unique mv codes:  7                       missing .*:  3814/4168

            tabulation:  Freq.   Numeric  Label
                          146         1
                           43         2
                           70         3
                           21         4
                            5         5
                            2         6
                           67         .
                         1314        .a  .a. Did not receive service
                         1729        .g  .g. Gender skip pattern
                            9        .m  .m. Missing
                            5        .s  .s. Skipped by participant
                            2        .w  .w. Answered 6 mon survey at 6
                                         wk visit
                          755        .x  .x. No 6 week survey

-------------------------------------------------------------------------------
m6_702_m                                          702: VMMC: Health facility name
-------------------------------------------------------------------------------

                  type:  string (str31)

         unique values:  59                       missing "":  823/4168

              examples:  "."
                         "."
                         "."
                         "."
```

```
              warning:  variable has embedded blanks

-----------------------------------------------------------------------------------------------
m6_703_m                                                      703: VMMC: Alone or accompanied
-----------------------------------------------------------------------------------------------

                 type:  numeric (byte)
                label:  hfpeople

                range:  [1,3]                        units:  1
        unique values:  3                        missing .:  67/4168
      unique mv codes:  7                       missing .*:  3828/4168

           tabulation:  Freq.   Numeric  Label
                          209         1  1. Alone
                           21         2  2. Spouse/Partner
                           43         3  3. Someone else
                           67         .
                         1314        .a  .a. Did not receive service
                         1729        .g  .g. Gender skip pattern
                            9        .m  .m. Missing
                           19        .s  .s. Skipped by participant
                            2        .w  .w. Answered 6 mon survey at 6
                                         wk visit
                          755        .x  .x. No 6 week survey

-----------------------------------------------------------------------------------------------
m6_704_m                                                   704: VMMC: Satisfied with services
-----------------------------------------------------------------------------------------------

                 type:  numeric (byte)
                label:  hfsat

                range:  [1,3]                        units:  1
        unique values:  3                        missing .:  67/4168
      unique mv codes:  7                       missing .*:  3827/4168

           tabulation:  Freq.   Numeric  Label
                            4         1  1. Not satisfied
                            7         2  2. Neither
                          263         3  3. Satisfied
                           67         .
                         1314        .a  .a. Did not receive service
                         1729        .g  .g. Gender skip pattern
                            9        .m  .m. Missing
                           18        .s  .s. Skipped by participant
                            2        .w  .w. Answered 6 mon survey at 6
                                         wk visit
                          755        .x  .x. No 6 week survey

-----------------------------------------------------------------------------------------------
m6_705_m                                    705: VMMC:  Waiting time at health facility (min)
-----------------------------------------------------------------------------------------------

                 type:  numeric (int)
                label:  numserv, but 32 nonmissing values are not labeled

                range:  [1,420]                      units:  1
        unique values:  32                       missing .:  67/4168
      unique mv codes:  7                       missing .*:  3832/4168

             examples:  .a    .a. Did not receive service
                        .g    .g. Gender skip pattern
                        .g    .g. Gender skip pattern
                        .g    .g. Gender skip pattern

-----------------------------------------------------------------------------------------------
m6_706_m                                                     706: VMMC: Weeks since circumcision
-----------------------------------------------------------------------------------------------
```

```
                type:  numeric (byte)
               label:  m6_706

               range:  [3,7]                        units:  1
       unique values:  5                        missing .:  67/4168
     unique mv codes:  7                       missing .*:  3821/4168

          tabulation:  Freq.   Numeric  Label
                           1         3  3. 3 weeks ago
                           4         4  4. 4 weeks ago
                           2         5  5. 5 weeks ago
                           5         6  6. 6 weeks ago
                         268         7  7. More than 6 weeks ago
                          67         .
                        1314        .a  .a. Did not receive service
                        1729        .g  .g. Gender skip pattern
                           9        .m  .m. Missing
                          12        .s  .s. Skipped by participant
                           2        .w  .w. Answered 6 wk survey at 6
                                        mon visit
                         755        .x  .x. No 6 month survey

-----------------------------------------------------------------------------------------------
m6_707_m                                 707: VMMC: Resumed sexual activity since circumcision
-----------------------------------------------------------------------------------------------

                type:  numeric (byte)
               label:  m6_707

               range:  [0,1]                        units:  1
       unique values:  2                        missing .:  67/4168
     unique mv codes:  7                       missing .*:  3812/4168

          tabulation:  Freq.   Numeric  Label
                          85         0  0. No
                         204         1  1. Yes
                          67         .
                        1314        .a  .a. Did not receive service
                        1729        .g  .g. Gender skip pattern
                           9        .m  .m. Missing
                           3        .s  .s. Skipped by participant
                           2        .w  .w. Answered 6 wk survey at 6
                                        mon visit
                         755        .x  .x. No 6 month survey

-----------------------------------------------------------------------------------------------
m6_708_m                           708: VMMC: Weeks after circumcision resumed sexual activity
-----------------------------------------------------------------------------------------------

                type:  numeric (byte)
               label:  m6_708

               range:  [0,7]                        units:  1
       unique values:  7                        missing .:  67/4168
     unique mv codes:  8                       missing .*:  3899/4168

          tabulation:  Freq.   Numeric  Label
                           1         0  0. <1 week
                           2         2  2. 2 weeks
                           4         3  3. 3 weeks
                          11         4  4. 4 weeks
                           7         5  5. 5 weeks
                          39         6  6. 6 weeks
                         138         7  7. More than 6 weeks ago
                          67         .
                        1314        .a  .a. Did not receive service
                          85        .c  .c. Have not resumed sexual
                                        activity
                        1729        .g  .g. Gender skip pattern
                           9        .m  .m. Missing
```

```
                        5        .s  .s. Skipped by participant
                        2        .w  .w. Answered 6 wk survey at 6
                                     mon visit
                      755        .x  .x. No 6 month survey

-------------------------------------------------------------------------------
m6_709_m                                709: VMMC: Travel time to health facility (min)
-------------------------------------------------------------------------------

                  type:  numeric (int)
                 label:  hfttime, but 27 nonmissing values are not labeled

                 range:  [2,180]                      units:  1
         unique values:  27                       missing .:  67/4168
       unique mv codes:  7                       missing .*:  3834/4168

            tabulation:  Freq.   Numeric  Label
                             2         2
                             1         3
                             7         5
                             2         8
                            17        10
                             1        14
                            24        15
                             1        17
                             2        18
                             1        19
                            19        20
                            11        25
                            54        30
                             1        31
                             1        33
                             4        35
                            14        40
                            20        45
                             6        50
                            50        60
                             2        70
                             1        80
                            11        90
                             9       120
                             1       130
                             3       140
                             2       180
                            67         .
                          1314        .a  .a. Did not receive service
                          1729        .g  .g. Gender skip pattern
                             9        .m  .m. Missing
                            25        .s  .s. Skipped by participant
                             2        .w  .w. Answered 6 mon survey at 6
                                          wk visit
                           755        .x  .x. No 6 week survey

-------------------------------------------------------------------------------
m6_710_m                                       710: VMMC: Travel mode to health faciity
-------------------------------------------------------------------------------

                  type:  numeric (byte)
                 label:  hftmode

                 range:  [1,7]                        units:  1
         unique values:  7                        missing .:  67/4168
       unique mv codes:  7                       missing .*:  3832/4168

            tabulation:  Freq.   Numeric  Label
                            76         1  1. Bus
                            24         2  2. Taxi
                             4         3  3. Someone drove in private car
                             9         4  4. Drove self
                           137         5  5. Walked
```

```
                         18         6  6. Bicycle
                          1         7  7. Other
                         67         .
                       1314        .a  .a. Did not receive service
                       1729        .g  .g. Gender skip pattern
                          9        .m  .m. Missing
                         23        .s  .s. Skipped by participant
                          2        .w  .w. Answered 6 mon survey at 6
                                       wk visit
                        755        .x  .x. No 6 week survey

-----------------------------------------------------------------------------------------------
m6_711_m                                                                711: VMMC: Transport cost
-----------------------------------------------------------------------------------------------

                  type:  numeric (byte)
                 label:  hftcost, but 17 nonmissing values are not labeled

                 range:  [0,100]                      units:  1
         unique values:  18                       missing .:  67/4168
       unique mv codes:  8                       missing .*:  3993/4168

            tabulation:  Freq.   Numeric  Label
                            17         0  0. Nothing
                             2         1
                             9         2
                            15         3
                             8         4
                            26         5
                             2         6
                             1         7
                            12        10
                             1        12
                             4        15
                             5        20
                             1        25
                             1        30
                             1        31
                             1        40
                             1        45
                             1       100
                            67         .
                          1314        .a  .a. Did not receive service
                           164        .b  .b. Own car, walked, biked
                          1729        .g  .g. Gender skip pattern
                             9        .m  .m. Missing
                            20        .s  .s. Skipped by participant
                             2        .w  .w. Answered 6 mon survey at 6
                                          wk visit
                           755        .x  .x. No 6 week survey

-----------------------------------------------------------------------------------------------
m6_712_m                                                          712: VMMC: Distance traveled (km)
-----------------------------------------------------------------------------------------------

                  type:  numeric (int)
                 label:  hftkm, but 16 nonmissing values are not labeled

                 range:  [0,888]                      units:  1
         unique values:  18                       missing .:  67/4168
       unique mv codes:  7                       missing .*:  3835/4168

            tabulation:  Freq.   Numeric  Label
                            20         0  0. <1
                            11         1
                            48         2
                            42         3
                            32         4
                            17         5
                            10         6
```

```
                          7         7
                          6         8
                          2         9
                          5        10
                          1        12
                          1        13
                          5        15
                          1        20
                          2        35
                          1        40
                         55       888  888. Don't Know
                         67         .
                       1314        .a  .a. Did not receive service
                       1729        .g  .g. Gender skip pattern
                          9        .m  .m. Missing
                         26        .s  .s. Skipped by participant
                          2        .w  .w. Answered 6 mon survey at 6
                                       wk visit
                        755        .x  .x. No 6 week survey

-------------------------------------------------------------------------------------------------
m6_713_m                                                   713: VMMC: Source of transport money
-------------------------------------------------------------------------------------------------

                 type:  numeric (byte)
                label:  hftsource

                range:  [1,6]                        units:  1
        unique values:  5                        missing .:  67/4168
      unique mv codes:  9                       missing .*:  4011/4168

           tabulation:  Freq.   Numeric  Label
                           22         1  1. Cutting down on other
                                         expenses
                           39         2  2. Savings
                            4         3  3. Borrowing
                           23         5  5. Donation
                            2         6  6. Other
                           67         .
                         1314        .a  .a. Did not receive service
                           17        .b  .b. Did not pay for transport
                          164        .c  .c. Own car, walked, biked
                         1729        .g  .g. Gender skip pattern
                            9        .m  .m. Missing
                           21        .s  .s. Skipped by participant
                            2        .w  .w. Answered 6 mon survey at 6
                                         wk visit
                          755        .x  .x. No 6 week survey

-------------------------------------------------------------------------------------------------
m6_714_m                  714: VMMC: What respondent would be doing if not at health facility
-------------------------------------------------------------------------------------------------

                 type:  numeric (byte)
                label:  hfnot

                range:  [1,6]                        units:  1
        unique values:  6                        missing .:  67/4168
      unique mv codes:  7                       missing .*:  3833/4168

           tabulation:  Freq.   Numeric  Label
                           87         1  1. Paid employment
                           42         2  2. Own business
                           19         3  3. Unpaid work/Housework
                           59         4  4. In school
                           51         5  5. Resting/No specific activity
                           10         6  6. Other
                           67         .
                         1314        .a  .a. Did not receive service
                         1729        .g  .g. Gender skip pattern
```

```
                          9        .m  .m. Missing
                         24        .s  .s. Skipped by participant
                          2        .w  .w. Answered 6 mon survey at 6
                                       wk visit
                        755        .x  .x. No 6 week survey

-----------------------------------------------------------------------------------------------
m6_800                     800: Self-rated health in past YEAR: 1 (very bad) - 10 (very good)
-----------------------------------------------------------------------------------------------

                  type:  numeric (byte)
                 label:  m6_num, but 10 nonmissing values are not labeled

                 range:  [1,10]                       units:  1
         unique values:  10                       missing .:  67/4168
       unique mv codes:  3                       missing .*:  760/4168

            tabulation:  Freq.   Numeric  Label
                            15         1
                            10         2
                            11         3
                           268         4
                           117         5
                           106         6
                           849         7
                           474         8
                           528         9
                           963        10
                            67         .
                             5        .m  .m. Missing
                           755        .x  .x. No 6 month survey

-----------------------------------------------------------------------------------------------
m6_801                    801: Self-rated health in past MONTH: 1 (very bad) - 10 (very good)
-----------------------------------------------------------------------------------------------

                  type:  numeric (byte)
                 label:  m6_num, but 10 nonmissing values are not labeled

                 range:  [1,10]                       units:  1
         unique values:  10                       missing .:  67/4168
       unique mv codes:  3                       missing .*:  760/4168

            tabulation:  Freq.   Numeric  Label
                            13         1
                             7         2
                             7         3
                           185         4
                            93         5
                           116         6
                           724         7
                           416         8
                           524         9
                          1256        10
                            67         .
                             5        .m  .m. Missing
                           755        .x  .x. No 6 month survey

-----------------------------------------------------------------------------------------------
m6_802a                                               802: Health problems past month: Fever
-----------------------------------------------------------------------------------------------

                  type:  numeric (byte)
                 label:  m6_yesno

                 range:  [0,1]                        units:  1
         unique values:  2                        missing .:  67/4168
       unique mv codes:  4                       missing .*:  762/4168

            tabulation:  Freq.   Numeric  Label
```

```
                        2755         0  0. No
                         584         1  1. Yes
                          67         .
                           5        .m  .m. Missing
                           2        .s  .s. Skipped by participant
                         755        .x  .x. No 6 month survey

-------------------------------------------------------------------------------
m6_802b                                   802: Health problems past month: Night sweat
-------------------------------------------------------------------------------

                 type:  numeric (byte)
                label:  m6_yesno

                range:  [0,1]                        units:  1
        unique values:  2                        missing .:  67/4168
      unique mv codes:  4                       missing .*:  762/4168

           tabulation:  Freq.   Numeric  Label
                        2950         0  0. No
                         389         1  1. Yes
                          67         .
                           5        .m  .m. Missing
                           2        .s  .s. Skipped by participant
                         755        .x  .x. No 6 month survey

-------------------------------------------------------------------------------
m6_802c                              802: Health problems past month: Rapid weight loss
-------------------------------------------------------------------------------

                 type:  numeric (byte)
                label:  m6_yesno

                range:  [0,1]                        units:  1
        unique values:  2                        missing .:  67/4168
      unique mv codes:  4                       missing .*:  762/4168

           tabulation:  Freq.   Numeric  Label
                        3077         0  0. No
                         262         1  1. Yes
                          67         .
                           5        .m  .m. Missing
                           2        .s  .s. Skipped by participant
                         755        .x  .x. No 6 month survey

-------------------------------------------------------------------------------
m6_802d                             802: Health problems past month: Recurring diarrhea
-------------------------------------------------------------------------------

                 type:  numeric (byte)
                label:  m6_yesno

                range:  [0,1]                        units:  1
        unique values:  2                        missing .:  67/4168
      unique mv codes:  4                       missing .*:  762/4168

           tabulation:  Freq.   Numeric  Label
                        2868         0  0. No
                         471         1  1. Yes
                          67         .
                           5        .m  .m. Missing
                           2        .s  .s. Skipped by participant
                         755        .x  .x. No 6 month survey

-------------------------------------------------------------------------------
m6_802e            802: Health problems past month: Recurring coughing or shortness of breath
-------------------------------------------------------------------------------

                 type:  numeric (byte)
                label:  m6_yesno
```

```
           range:  [0,1]                        units:  1
   unique values:  2                        missing .:  67/4168
 unique mv codes:  4                       missing .*:  762/4168

      tabulation:  Freq.   Numeric  Label
                    2860         0  0. No
                     479         1  1. Yes
                      67         .
                       5        .m  .m. Missing
                       2        .s  .s. Skipped by participant
                     755        .x  .x. No 6 month survey

-----------------------------------------------------------------------------------------------
m6_802f                                   802: Health problems past month: Recurring vomiting
-----------------------------------------------------------------------------------------------

            type:  numeric (byte)
           label:  m6_yesno

           range:  [0,1]                        units:  1
   unique values:  2                        missing .:  67/4168
 unique mv codes:  4                       missing .*:  762/4168

      tabulation:  Freq.   Numeric  Label
                    3089         0  0. No
                     250         1  1. Yes
                      67         .
                       5        .m  .m. Missing
                       2        .s  .s. Skipped by participant
                     755        .x  .x. No 6 month survey

-----------------------------------------------------------------------------------------------
m6_802g                                    802: Health problems past month: Recurring fatigue
-----------------------------------------------------------------------------------------------

            type:  numeric (byte)
           label:  m6_yesno

           range:  [0,1]                        units:  1
   unique values:  2                        missing .:  67/4168
 unique mv codes:  4                       missing .*:  762/4168

      tabulation:  Freq.   Numeric  Label
                    2958         0  0. No
                     381         1  1. Yes
                      67         .
                       5        .m  .m. Missing
                       2        .s  .s. Skipped by participant
                     755        .x  .x. No 6 month survey

-----------------------------------------------------------------------------------------------
m6_803                                               803: Covered by health insurance/scheme
-----------------------------------------------------------------------------------------------

            type:  numeric (byte)
           label:  m6_yesno

           range:  [0,88]                       units:  1
   unique values:  3                        missing .:  67/4168
 unique mv codes:  3                       missing .*:  760/4168

      tabulation:  Freq.   Numeric  Label
                    3148         0  0. No
                     191         1  1. Yes
                       2        88  88. Don't Know
                      67         .
                       5        .m  .m. Missing
                     755        .x  .x. No 6 month survey
```

```
--------------------------------------------------------------------------------------------
m6_804                                             804: Type of health insurance/scheme
--------------------------------------------------------------------------------------------

                  type:  numeric (byte)
                 label:  m6_804

                 range:  [1,96]                       units:  1
         unique values:  7                        missing .:  67/4168
       unique mv codes:  4                       missing .*:  3910/4168

            tabulation:  Freq.   Numeric  Label
                            31         1  1. MUTUAL HEALTH/COMMUNITY-BASED
                           103         2  2. EMPLOYER
                             4         3  3. SOCIAL SECURITY
                            21         4  4. OTHER PRIVATELY PURCHASED
                                          COMMERCIAL
                             8         5  5. LOW COST PRE-PAYMENT
                            12         6  6. HIGH COST PRE-PAYMENT
                            12        96  96. OTHER
                            67         .
                          3148        .a  .a. No insurance
                             7        .m  .m. Missing
                           755        .x  .x. No 6 month survey

--------------------------------------------------------------------------------------------
m6_804a                                 804a: If other, specify type of health insurance
--------------------------------------------------------------------------------------------

                  type:  string (str60)

         unique values:  11                       missing "":  4156/4168

            tabulation:  Freq.  Value
                          4156  ""
                             1  "African life"
                             1  "Eastern water and sewerage"
                             1  "Health care"
                             1  "M life insurance"
                             1  "Medical scheme through husband"
                             2  "Metropolitan"
                             1  "Napsa"
                             1  "Participant unsure as it is extension
                                of his mother's policy"
                             1  "Through Husbands Work place"
                             1  "Uncertain?  as he is covered under
                                mother's work scheme."
                             1  "ppt unsure but related to work"

               warning:  variable has embedded blanks

--------------------------------------------------------------------------------------------
m6_805                               805: FP: Currently doing something to avoid pregnancy
--------------------------------------------------------------------------------------------

                  type:  numeric (byte)
                 label:  m6_805

                 range:  [0,1]                        units:  1
         unique values:  2                        missing .:  67/4168
       unique mv codes:  6                       missing .*:  1085/4168

            tabulation:  Freq.   Numeric  Label
                           985         0  0. No
                          2031         1  1. Yes
                            67         .
                           214        .a  .a. FP Client: see section 500
                           110        .e  .e. Programming skip error: Most
                                          males who answered 804 were
                                          skipped out of 805-807
```

```
                          5        .m  .m. Missing
                          1        .s  .s. Skipped by participant
                        755        .x  .x. No 6 month survey

m6_805:
  1.  10 cases made NO who originally reported YES to m6_805, but said NO to all methods in
      m6_807.

-----------------------------------------------------------------------------------------------
m6_806                                              806: FP: Know place to get family planning
-----------------------------------------------------------------------------------------------

                  type:  numeric (byte)
                 label:  m6_806

                 range:  [0,1]                        units:  1
         unique values:  2                        missing .:  67/4168
       unique mv codes:  7                       missing .*:  3126/4168

            tabulation:  Freq.   Numeric  Label
                           328         0  0. No
                           647         1  1. Yes
                            67         .
                           214        .a  .a. FP Client: see section 500
                          2031        .b  .b. Currently doing something to
                                              avoid pregnancy: 805 = 1
                           110        .e  .e. Programming skip error: Most
                                              males who answered 804 were
                                              skipped out of 805-807
                            15        .m  .m. Missing
                             1        .s  .s. Skipped by participant
                           755        .x  .x. No 6 month survey

-----------------------------------------------------------------------------------------------
m6_807a                                                 807: FP: Method: Female sterilization
-----------------------------------------------------------------------------------------------

                  type:  numeric (byte)
                 label:  m6_807a

                 range:  [0,1]                        units:  1
         unique values:  2                        missing .:  67/4168
       unique mv codes:  7                       missing .*:  2071/4168

            tabulation:  Freq.   Numeric  Label
                          2027         0  0. No
                             3         1  1. Yes
                            67         .
                           214        .a  .a. FP Client: see section 500
                           985        .b  .b. Not using a method: 805 = 0
                           110        .e  .e. Programming skip error: Most
                                              males who answered 804 were
                                              skipped out of 805-807
                             5        .m  .m. Missing
                             2        .s  .s. Skipped by participant
                           755        .x  .x. No 6 month survey

-----------------------------------------------------------------------------------------------
m6_807b                                                   807: FP: Method: Male sterilization
-----------------------------------------------------------------------------------------------

                  type:  numeric (byte)
                 label:  m6_807b

                 range:  [0,1]                        units:  1
         unique values:  2                        missing .:  67/4168
       unique mv codes:  7                       missing .*:  2071/4168

            tabulation:  Freq.   Numeric  Label
                          2029         0  0. No
```

```
                    1         1  1. Yes
                   67         .
                  214        .a  .a. FP Client: see section 500
                  985        .b  .b. Not using a method: 805 = 0
                  110        .e  .e. Programming skip error: Most
                                 males who answered 804 were
                                 skipped out of 805-807
                    5        .m  .m. Missing
                    2        .s  .s. Skipped by participant
                  755        .x  .x. No 6 month survey

-------------------------------------------------------------------------------
m6_807c                                                   807: FP: Method: Pill
-------------------------------------------------------------------------------

                  type:  numeric (byte)
                 label:  m6_807c

                 range:  [0,1]                        units:  1
         unique values:  2                        missing .:  67/4168
       unique mv codes:  7                       missing .*:  2071/4168

            tabulation:  Freq.   Numeric  Label
                          1880         0  0. No
                           150         1  1. Yes
                            67         .
                           214        .a  .a. FP Client: see section 500
                           985        .b  .b. Not using a method: 805 = 0
                           110        .e  .e. Programming skip error: Most
                                          males who answered 804 were
                                          skipped out of 805-807
                             5        .m  .m. Missing
                             2        .s  .s. Skipped by participant
                           755        .x  .x. No 6 month survey

-------------------------------------------------------------------------------
m6_807d                                                    807: FP: Method: IUD
-------------------------------------------------------------------------------

                  type:  numeric (byte)
                 label:  m6_807d

                 range:  [0,1]                        units:  1
         unique values:  2                        missing .:  67/4168
       unique mv codes:  7                       missing .*:  2071/4168

            tabulation:  Freq.   Numeric  Label
                          1890         0  0. No
                           140         1  1. Yes
                            67         .
                           214        .a  .a. FP Client: see section 500
                           985        .b  .b. Not using a method: 805 = 0
                           110        .e  .e. Programming skip error: Most
                                          males who answered 804 were
                                          skipped out of 805-807
                             5        .m  .m. Missing
                             2        .s  .s. Skipped by participant
                           755        .x  .x. No 6 month survey

-------------------------------------------------------------------------------
m6_807e                                            807: FP: Method: Injectables
-------------------------------------------------------------------------------

                  type:  numeric (byte)
                 label:  m6_807e

                 range:  [0,1]                        units:  1
         unique values:  2                        missing .:  67/4168
       unique mv codes:  7                       missing .*:  2071/4168
```

```
          tabulation:  Freq.   Numeric  Label
                        1844         0  0. No
                         186         1  1. Yes
                          67         .  
                         214        .a  .a. FP Client: see section 500
                         985        .b  .b. Not using a method: 805 = 0
                         110        .e  .e. Programming skip error: Most
                                        males who answered 804 were
                                        skipped out of 805-807
                           5        .m  .m. Missing
                           2        .s  .s. Skipped by participant
                         755        .x  .x. No 6 month survey
```

```
m6_807f                                                   807: FP: Method: Implants
```

```
                type:  numeric (byte)
               label:  m6_807f

               range:  [0,1]                        units:  1
       unique values:  2                        missing .:  67/4168
     unique mv codes:  7                       missing .*:  2071/4168

          tabulation:  Freq.   Numeric  Label
                        1247         0  0. No
                         783         1  1. Yes
                          67         .  
                         214        .a  .a. FP Client: see section 500
                         985        .b  .b. Not using a method: 805 = 0
                         110        .e  .e. Programming skip error: Most
                                        males who answered 804 were
                                        skipped out of 805-807
                           5        .m  .m. Missing
                           2        .s  .s. Skipped by participant
                         755        .x  .x. No 6 month survey
```

```
m6_807g                                                807: FP: Method: Male condom
```

```
                type:  numeric (byte)
               label:  m6_807g

               range:  [0,1]                        units:  1
       unique values:  2                        missing .:  67/4168
     unique mv codes:  7                       missing .*:  2071/4168

          tabulation:  Freq.   Numeric  Label
                        1225         0  0. No
                         805         1  1. Yes
                          67         .  
                         214        .a  .a. FP Client: see section 500
                         985        .b  .b. Not using a method: 805 = 0
                         110        .e  .e. Programming skip error: Most
                                        males who answered 804 were
                                        skipped out of 805-807
                           5        .m  .m. Missing
                           2        .s  .s. Skipped by participant
                         755        .x  .x. No 6 month survey
```

```
m6_807h                                              807: FP: Method: Female condom
```

```
                type:  numeric (byte)
               label:  m6_807h

               range:  [0,1]                        units:  1
       unique values:  2                        missing .:  67/4168
```

```
      unique mv codes:  7                      missing .*:  2071/4168

          tabulation:  Freq.   Numeric  Label
                        1988         0  0. No
                          42         1  1. Yes
                          67         .
                         214        .a  .a. FP Client: see section 500
                         985        .b  .b. Not using a method: 805 = 0
                         110        .e  .e. Programming skip error: Most
                                        males who answered 804 were
                                        skipped out of 805-807
                           5        .m  .m. Missing
                           2        .s  .s. Skipped by participant
                         755        .x  .x. No 6 month survey

------------------------------------------------------------------------------
m6_807i                                             807: FP: Method: Diaphragm
------------------------------------------------------------------------------

                type:  numeric (byte)
               label:  m6_807i

               range:  [0,1]                        units:  1
       unique values:  2                        missing .:  67/4168
      unique mv codes:  7                      missing .*:  2071/4168

          tabulation:  Freq.   Numeric  Label
                        2025         0  0. No
                           5         1  1. Yes
                          67         .
                         214        .a  .a. FP Client: see section 500
                         985        .b  .b. Not using a method: 805 = 0
                         110        .e  .e. Programming skip error: Most
                                        males who answered 804 were
                                        skipped out of 805-807
                           5        .m  .m. Missing
                           2        .s  .s. Skipped by participant
                         755        .x  .x. No 6 month survey

------------------------------------------------------------------------------
m6_807j                                            807: FP: Method: Foam/Jelly
------------------------------------------------------------------------------

                type:  numeric (byte)
               label:  m6_807j

               range:  [0,0]                        units:  1
       unique values:  1                        missing .:  67/4168
      unique mv codes:  7                      missing .*:  2071/4168

          tabulation:  Freq.   Numeric  Label
                        2030         0  0. No
                          67         .
                         214        .a  .a. FP Client: see section 500
                         985        .b  .b. Not using a method: 805 = 0
                         110        .e  .e. Programming skip error: Most
                                        males who answered 804 were
                                        skipped out of 805-807
                           5        .m  .m. Missing
                           2        .s  .s. Skipped by participant
                         755        .x  .x. No 6 month survey

------------------------------------------------------------------------------
m6_807k                        807: FP: Method: Lactational amenorrhea method
------------------------------------------------------------------------------

                type:  numeric (byte)
               label:  m6_807k

               range:  [0,1]                        units:  1
```

```
        unique values:  2                        missing .:  67/4168
      unique mv codes:  7                       missing .*:  2071/4168

           tabulation:  Freq.   Numeric  Label
                         2029         0  0. No
                            1         1  1. Yes
                           67         .
                          214        .a  .a. FP Client: see section 500
                          985        .b  .b. Not using a method: 805 = 0
                          110        .e  .e. Programming skip error: Most
                                         males who answered 804 were
                                         skipped out of 805-807
                            5        .m  .m. Missing
                            2        .s  .s. Skipped by participant
                          755        .x  .x. No 6 month survey

-------------------------------------------------------------------------------------------------
m6_807l                                                      807: FP: Method: Rhythm method
-------------------------------------------------------------------------------------------------

                 type:  numeric (byte)
                label:  m6_807l

                range:  [0,1]                        units:  1
        unique values:  2                        missing .:  67/4168
      unique mv codes:  7                       missing .*:  2071/4168

           tabulation:  Freq.   Numeric  Label
                         1989         0  0. No
                           41         1  1. Yes
                           67         .
                          214        .a  .a. FP Client: see section 500
                          985        .b  .b. Not using a method: 805 = 0
                          110        .e  .e. Programming skip error: Most
                                         males who answered 804 were
                                         skipped out of 805-807
                            5        .m  .m. Missing
                            2        .s  .s. Skipped by participant
                          755        .x  .x. No 6 month survey

-------------------------------------------------------------------------------------------------
m6_807m                                                         807: FP: Method: Withdrawal
-------------------------------------------------------------------------------------------------

                 type:  numeric (byte)
                label:  m6_807m

                range:  [0,1]                        units:  1
        unique values:  2                        missing .:  67/4168
      unique mv codes:  7                       missing .*:  2071/4168

           tabulation:  Freq.   Numeric  Label
                         1987         0  0. No
                           43         1  1. Yes
                           67         .
                          214        .a  .a. FP Client: see section 500
                          985        .b  .b. Not using a method: 805 = 0
                          110        .e  .e. Programming skip error: Most
                                         males who answered 804 were
                                         skipped out of 805-807
                            5        .m  .m. Missing
                            2        .s  .s. Skipped by participant
                          755        .x  .x. No 6 month survey

-------------------------------------------------------------------------------------------------
m6_807n                                                       807: FP: Method: Other method
-------------------------------------------------------------------------------------------------

                 type:  numeric (byte)
                label:  m6_807n
```

```
            range:  [0,1]                        units:  1
    unique values:  2                        missing .:  67/4168
  unique mv codes:  7                       missing .*:  2071/4168

       tabulation:  Freq.   Numeric  Label
                     2010         0  0. No
                       20         1  1. Yes
                       67         .
                      214        .a  .a. FP Client: see section 500
                      985        .b  .b. Not using a method: 805 = 0
                      110        .e  .e. Programming skip error: Most
                                     males who answered 804 were
                                     skipped out of 805-807
                        5        .m  .m. Missing
                        2        .s  .s. Skipped by participant
                      755        .x  .x. No 6 month survey

-----------------------------------------------------------------------------------------------
m6_902                                                  902: Last time had sexual intercourse
-----------------------------------------------------------------------------------------------

             type:  numeric (byte)
            label:  m6_902

            range:  [0,4]                        units:  1
    unique values:  5                        missing .:  67/4168
  unique mv codes:  4                       missing .*:  772/4168

       tabulation:  Freq.   Numeric  Label
                      336         0  0. Never had sex
                     1207         1  1. Today/Days ago
                      822         2  2. More than a week ago
                      766         3  3. More than a month ago
                      198         4  4. More than a year ago
                       67         .
                        6        .m  .m. Missing
                       11        .s  .s. Skipped by participant
                      755        .x  .x. No 6 month survey

-----------------------------------------------------------------------------------------------
m6_903                                                        903: Times had sex in last week
-----------------------------------------------------------------------------------------------

             type:  numeric (byte)
            label:  m6_903, but 12 nonmissing values are not labeled

            range:  [0,88]                       units:  1
    unique values:  12                       missing .:  67/4168
  unique mv codes:  6                       missing .*:  2887/4168

       tabulation:  Freq.   Numeric  Label
                       64         0
                      296         1
                      313         2
                      249         3
                      101         4
                       31         5
                       38         6
                       24         7
                       16         8
                        6         9
                        1        10
                       75        88
                       67         .
                      336        .a  .a. Never had sex
                     1786        .b  .b. Sex >1 week ago
                        6        .m  .m. Missing
                        4        .s  .s. Skipped by participant
                      755        .x  .x. No 6 month survey
```

```
--------------------------------------------------------------------------------------------
m6_904                                    904: Times used condom when had sex in last week
--------------------------------------------------------------------------------------------

                  type:  numeric (byte)
                 label:  m6_904, but 11 nonmissing values are not labeled

                 range:  [0,88]                       units:  1
         unique values:  11                       missing .:  67/4168
       unique mv codes:  6                       missing .*:  2884/4168

            tabulation:  Freq.   Numeric  Label
                           565         0
                           201         1
                           158         2
                            94         3
                            39         4
                            16         5
                            14         6
                             8         7
                            16         8
                             1         9
                           105        88
                            67         .
                           336        .a  .a. Never had sex
                          1786        .b  .b. Sex >1 week ago
                             6        .m  .m. Missing
                             1        .s  .s. Skipped by participant
                           755        .x  .x. No 6 month survey

--------------------------------------------------------------------------------------------
m6_905                       905: Relationship to LAST person with whom had sexual intercourse
--------------------------------------------------------------------------------------------

                  type:  numeric (byte)
                 label:  m6_905

                 range:  [1,6]                        units:  1
         unique values:  6                        missing .:  67/4168
       unique mv codes:  5                       missing .*:  1295/4168

            tabulation:  Freq.   Numeric  Label
                          1460         1  1. Husband/wife
                           316         2  2. Live-in partner
                           886         3  3. Girlfriend/boyfriend not
                                          living with you
                            50         4  4. Person you paid or who paid
                                          you for sex
                            68         5  5. Casual acquaintance
                            26         6  6. Other
                            67         .
                           336        .a  .a. Never had sex
                           198        .b  .b. Sex >1 year ago
                             6        .m  .m. Missing
                           755        .x  .x. No 6 month survey

--------------------------------------------------------------------------------------------
m6_906                                                906: Condom used last time had sex
--------------------------------------------------------------------------------------------

                  type:  numeric (byte)
                 label:  m6_906

                 range:  [0,88]                       units:  1
         unique values:  3                        missing .:  67/4168
       unique mv codes:  5                       missing .*:  1295/4168

            tabulation:  Freq.   Numeric  Label
                          1566         0  0. No
```

```
                      1198         1  1. Yes
                        42        88  88. Don't Know
                        67         .
                       336        .a  .a. Never had sex
                       198        .b  .b. Sex >1 year ago
                         6        .m  .m. Missing
                       755        .x  .x. No 6 month survey
```

```
-------------------------------------------------------------------------------
m6_907                  907: Use condom every time had sex with LAST person in last 12 months
-------------------------------------------------------------------------------

                  type:  numeric (byte)
                 label:  m6_907

                 range:  [0,88]                       units:  1
         unique values:  3                        missing .:  67/4168
       unique mv codes:  6                       missing .*:  1296/4168

            tabulation:  Freq.   Numeric  Label
                          1728         0  0. No
                          1016         1  1. Yes
                            61        88  88. Don't Know
                            67         .
                           336        .a  .a. Never had sex
                           198        .b  .b. Sex >1 year ago
                             6        .m  .m. Missing
                             1        .s  .s. Skipped by participant
                           755        .x  .x. No 6 month survey
```

```
-------------------------------------------------------------------------------
m6_908               908: Having sex with other pople during time having sex with LAST person
-------------------------------------------------------------------------------

                  type:  numeric (byte)
                 label:  m6_908

                 range:  [0,88]                       units:  1
         unique values:  3                        missing .:  67/4168
       unique mv codes:  5                       missing .*:  1295/4168

            tabulation:  Freq.   Numeric  Label
                          2248         0  0. No
                           500         1  1. Yes
                            58        88  88. Don't Know
                            67         .
                           336        .a  .a. Never had sex
                           198        .b  .b. Sex >1 year ago
                             6        .m  .m. Missing
                           755        .x  .x. No 6 month survey
```

```
-------------------------------------------------------------------------------
m6_911                                 911: Number new sexual partners in last 6 months
-------------------------------------------------------------------------------

                  type:  numeric (byte)
                 label:  m6_911, but 11 nonmissing values are not labeled

                 range:  [0,10]                       units:  1
         unique values:  11                       missing .:  67/4168
       unique mv codes:  7                       missing .*:  1303/4168

            tabulation:  Freq.   Numeric  Label
                          1332         0
                          1013         1
                           265         2
                           107         3
                            39         4
                            12         5
                            15         6
```

```
                            4         7
                            3         8
                            6         9
                            2        10
                           67         .
                          335        .a  .a. Never had sex
                          198        .b  .b. Sex >1 year ago
                            6        .m  .m. Missing
                            7        .s  .s. Skipped by participant
                            2        .w  .w. Answered 6 wk survey at 6
                                         mon visit
                          755        .x  .x. No 6 month survey

-----------------------------------------------------------------------------------------------
m6_currpart_an                            Analysis: Currently has spouse/primary sex partner
-----------------------------------------------------------------------------------------------

                  type:  numeric (byte)
                 label:  m6_yesno

                 range:  [0,1]                        units:  1
         unique values:  2                        missing .:  67/4168
       unique mv codes:  2                       missing .*:  758/4168

           tabulation:  Freq.   Numeric  Label
                          519         0  0. No
                         2824         1  1. Yes
                           67         .
                          758        .m  .m. Missing

-----------------------------------------------------------------------------------------------
ch_Site                                                                            CHAMP: Site
-----------------------------------------------------------------------------------------------

                  type:  string (str40)

         unique values:  10                       missing "":  2644/4168

           tabulation:  Freq.  Value
                         2644  ""
                           97  "99: Missing from CHAMP database"
                          278  "Chawama Clinic - Maternity Ward"
                            4  "Chawama Clinic - Out Patient Ward
                               (VMMC)"
                            3  "Chipata Gen Hosp - OP VMMC"
                          132  "Kamwala Clinic - TB, STI & HIV Clinic"
                          257  "Kapata Urban Clinic, MCH"
                          350  "Kapata Urban Clinic, TB, STI & HIV"
                          150  "SFH HCT - Cairo Road"
                          252  "SFH New Start"
                            1  "SFH VMMC"

               warning:  variable has embedded blanks

-----------------------------------------------------------------------------------------------
ch_Gender                                                                        CHAMP: Gender
-----------------------------------------------------------------------------------------------

                  type:  numeric (byte)

                 range:  [0,1]                        units:  1
         unique values:  2                        missing .:  1141/4168
       unique mv codes:  3                       missing .*:  1524/4168

           tabulation:  Freq.  Value
                          479  0
                         1024  1
                         1141  .
                          205  .j
                         1319  .k
```

```
-------------------------------------------------------------------------------------------
ch_Referrals                                                              CHAMP: Referral
-------------------------------------------------------------------------------------------

                  type:  string (str244)

         unique values:  942                      missing "":  2644/4168

              examples:  ""
                         ""
                         ""
                         "Referred for : CCS: CERVICAL CANCER SCREENING on May
                         8 2014  to Chawama Clinic - Maternity WardReferred
                         for : HIV: HTC - COUPLES on May  8 2014  to Chawama
                         Clinic - Out Patient Ward (VMMC)"

               warning:  variable has embedded and trailing blanks

-------------------------------------------------------------------------------------------
ch_Visit                                                               CHAMP: Visit notes
-------------------------------------------------------------------------------------------

                  type:  string (str48)

         unique values:  3                        missing "":  2644/4168

            tabulation:  Freq.  Value
                          2644  ""
                          1422  "No visit  recorded at any site for this
                                service"
                           101  "99: Missing from CHAMP database"
                             1  "Client had no number "

               warning:  variable has leading, embedded, and trailing blanks

-------------------------------------------------------------------------------------------
ch_Result                                                              CHAMP: Result code
-------------------------------------------------------------------------------------------

                  type:  numeric (byte)
                 label:  ch_Result

                 range:  [1,5]                        units:  1
         unique values:  5                        missing .:  1141/4168
       unique mv codes:  3                       missing .*:  1524/4168

            tabulation:  Freq.   Numeric  Label
                           661         1  1. Successful
                           404         2  2. Unsuccessful
                           210         3  3. Computer didn't generate
                           143         4  4. Omitted
                            85         5  5. Client had no number
                          1141         .
                           205        .j  .j. Ineligible: External site
                                          client
                          1319        .k  .k. Ineligible: SOC arm

-------------------------------------------------------------------------------------------
ch_RefDate                                                           CHAMP: Referral date
-------------------------------------------------------------------------------------------

                  type:  numeric daily date (int)

                 range:  [19711,20037]                units:  1
       or equivalently:  [19dec2013,10nov2014]        units:  days
         unique values:  210                      missing .:  1218/4168
       unique mv codes:  3                       missing .*:  1524/4168

                  mean:   19864.3 = 21may2014 (+ 8 hours)
```

```
              std. dev:   72.5984

           percentiles:        10%       25%       50%       75%       90%
                             19774     19807     19859     19919     19969
                         20feb2014 25mar2014 16may2014 15jul2014 03sep2014

.
.
end of do-file

.
.
.
end of do-file

. do "C:\Users\JDIGIT~1\AppData\Local\Temp\STD0f000000.tmp"

. *** INPUT:    REacH_StudyData
. *** OUTPUT:   REacH_AnalysisData
. *** CREATED:  1 MAY 2015
. *** UPDATED:  19 OCT 2015
. *** AUTHORS:  JD, PH
.
. *** SETTINGS AND PATHS
.     clear

.         clear matrix

.         clear mata

.         set more off

.         set maxvar 25000


.         set matsize 800

.     getcurrdate       /* PERSONAL ADO - GETS DATE FORMATS */


Format of currdate1 is 28Oct2015
Format of currdate2 is 2015-10-28
Format of currdate3 is 10-28-2015



.
.     capture log close
```
